# Targeting secreted PLA_2_ interactions with EGFR and vimentin to arrest prostate tumour growth

**DOI:** 10.1038/s41419-025-08280-x

**Published:** 2025-12-20

**Authors:** Timothy J. Mann, Ryung Rae Kim, Mila Sajinovic, Shadma Fatima, Vinod Kumar, Abdel Qader Al Bawab, Hiba Bahidh, Edwin Huang, Anya Salih, Enrico Gratton, Nathan Main, Maria George Elias, Isabelle Meyer-Carrive, Peter Galettis, Russell Pickford, David G. Harman, Jun Zeng, Winston Liauw, Albert S. Mellick, Gregory James Cooney, Qihan Dong, Garry G. Graham, W. Bret Church, Pamela J. Russell, Paul de Souza, Kieran F. Scott

**Affiliations:** 1 School of Medicine, Western Sydney University, Campbelltown, NSW Australia; 2 Ingham Institute of Applied Medical Research, Liverpool, NSW Australia; 3 School of Pharmacy, Faculty of Medicine and Health, University of Sydney, Camperdown, NSW Australia; 4 St Vincent’s Hospital Clinical School, The University of New South Wales, Darlinghurst, NSW Australia; 5 Faculty of Health, Medicine and Behavioural Sciences. The University of Queensland, Brisbane, QLD Australia; 6 Faculty of Pharmacy, Al-Zaytoonah University of Jordan, Amman, Jordan; 7 Australian Institute of Bioengineering and Nanotechnology, The University of Queensland, Brisbane, QLD Australia; 8 School of Life Science, Faculty of Science, University of Technology Sydney, Ultimo, NSW Australia; 9 Fluoresci Research, Cogra Bay, NSW Australia; 10 Laboratory of Fluorescence Dynamics, University of California, Irvine, CA USA; 11 School of Science, Western Sydney University, Campbelltown, NSW Australia; 12 ICP Firefly Pty Ltd., Alexandria, NSW Australia; 13 St George Hospital Clinical School, The University of New South Wales, Kogarah, NSW Australia; 14 Mark Wainwright Analytical Centre, The University of New South Wales, Kensington, NSW Australia; 15 MedChemSoft Solutions. 42-44 Lakeview Dr, Scoresby, VIC Australia; 16 Graduate School of Biomedical Engineering, The University of New South Wales, Kensington, NSW Australia; 17 Charles Perkins Centre, University of Sydney, Camperdown, NSW Australia; 18 Central Clinical School, Faculty of Medicine and Health, University of Sydney, Camperdown, NSW Australia; 19 Queensland University of Technology, Brisbane, QLD Australia; 20 Nepean Clinical School, Faculty of Medicine and Health, University of Sydney, Kingswood, NSW Australia

**Keywords:** Prostate cancer, Drug development, Intermediate filaments, Peptides, Preclinical research

## Abstract

The secreted phospholipase A_2_ human group IIA (hGIIA) is overexpressed in prostate cancer (PCa), where its expression is closely aligned with malignancy. While its enzymatic activity is important in mediating innate immunity, here we highlight that hGIIA contributes to PCa pathology primarily through specific protein-protein interactions. We have developed cyclic peptides cF and c2, derived from the structure of hGIIA, that selectively inhibit these interactions and inhibit PCa growth. hGIIA interacts directly with epidermal growth factor receptor (EGFR), resulting in increased cytosolic PLA_2_-α activation and prostaglandin E_2_ production, which is suppressed by c2. Further, vimentin was identified to bind hGIIA in PCa cells, modulating hGIIA intracellular trafficking. c2 binds vimentin, blocking this interaction and initiating vimentin-mediated aggresome formation and apoptosis even in the absence of hGIIA. cF and c2 suppress androgen-sensitive, castrate-resistant and androgen-independent models of tumour growth in vivo at doses as low as 0.1 mg/kg, are non-toxic, orally bioavailable and cell-permeable. Critically, as with hGIIA, EGFR and vimentin are also increasingly expressed as PCa develops, cF and c2 may represent a novel therapeutic option for incurable metastatic castrate resistant PCa. Our findings identify hGIIA as an innate immune effector that regulates both inflammation and PCa progression and describe a novel class of hGIIA protein-protein interaction inhibitor with therapeutic potential in PCa.

## Introduction

In prostate cancer (PCa), the development of resistance to standard androgen-directed therapies is inevitable and leads to incurable metastatic castrate resistant PCa (mCRPC). It is therefore critical to develop therapies that target novel mCRPC-promoting mechanisms. Aberrant inflammation is an enabling characteristic of cancer, increasing metastatic potential by shaping the tumour microenvironment and promoting angiogenesis, cell plasticity, invasion, and tissue remodelling [1–3]. Here, we identify a secreted human phospholipase A_2_ (PLA_2_) known as hGIIA (human group IIA PLA_2,_ human sPLA_2_-IIA) as a key molecular mediator of pro-tumorigenic inflammation and present the characterisation of a new class of anti-inflammatory cyclic peptides; cF (cyclo-(Phe-Leu Ser-Tyr-Arg)) and c2 (cyclo-((2-Nal)-Leu-Ser-(2-Nal)-Arg)), that selectively inhibit the catalysis-independent function of hGIIA with potential to impact cancer treatment.

The primary physiological role of hGIIA is in host defence [4], with strong bactericidal action driven by its phospholipase enzyme activity that cleaves phospholipids at the *sn*-2 position. However, it is also a known amplifier of cytokine-mediated inflammation in the innate immune response, being upregulated in multiple inflammatory disorders and cancers [5]. Able to modulate its own expression through multiple positive feedback loops [6], hGIIA expression is up to 22-fold higher in PCa tissue than in healthy prostate, where its expression is confined to luminal epithelial cells, and can cause PCa cell proliferation [7]. hGIIA plays a critical role in the amplification of inflammation through indirect upregulation of enzymes of the eicosanoid pathway [8], a key driver of inflammation and cancer [9]. PLA_2_ activity is a rate-limiting step of the eicosanoid pathway, with higher hGIIA expression correlating with increased eicosanoid production [10, 11]. Therefore, hGIIA inhibition is an attractive strategy for the treatment of inflammatory diseases, including cancer [5, 12]. This has led to the development of potent inhibitors of hGIIA enzymatic activity such as Varespladib^®^ which entered clinical trials for the treatment of sepsis, rheumatoid arthritis, asthma and cardiovascular disease [12, 13]. Despite the initial promise of this approach, there are no hGIIA inhibitors approved for clinical use largely due to safety concerns, with Phase III trials in patients with acute coronary syndrome being terminated early due to futility and possible harm [12, 13].

Recent studies have shown that while hGIIA catalytic activity is crucial for its physiological roles, such as microbial defence, hGIIA also drives eicosanoid overproduction and inflammation in human rheumatoid arthritis through a catalysis-independent mechanism, acting as an activator of cytosolic PLA_2_-α (cPLA_2_-α) [8, 11, 14]. Further, hGIIA has been identified as an activator of epidermal growth factor receptor (EGFR) [6, 15, 16], which leads to extracellular signal-related kinase (ERK) phosphorylation and potentially cPLA_2_-α activation. In rheumatoid synoviocytes, hGIIA induces ERK in the absence of cytokine stimulation [8] and activation of cells was accompanied by its rapid internalisation and colocalisation with the intermediate filament protein vimentin [14]. In these cells, we have discovered that, unlike other inhibitors, cF and c2 selectively inhibit the previously untargeted catalysis-independent mechanism of hGIIA [8] with ~1000-fold less potency in the inhibition of hGIIA catalytic activity than other hGIIA inhibitors. Inhibition results in suppression of eicosanoid production coinciding with a loss of hGIIA colocalisation with vimentin [14].

The present study investigated this catalysis-independent mechanism and the effect of its pharmacological inhibition in the context of PCa. We show that hGIIA directly binds to vimentin both in vitro and in PCa cells, a novel interaction that facilitates hGIIA intracellular trafficking. We further demonstrate that c2 has a multi-modal mechanism of action inhibiting: (i) hGIIA binding to EGFR and subsequent eicosanoid production, and (ii) modulating vimentin-led trafficking of hGIIA, leading to aggresome build-up and apoptosis. Furthermore, we show that cF and c2 are non-toxic, orally active, cell-permeable and effective inhibitors of three different xenograft models encompassing androgen-dependent early-stage PCa, castrate-resistant PCa and androgen-independent PCa. Taken together, these findings demonstrate for the first time the clinical potential of a powerful new class of hGIIA-selective protein-protein interaction inhibitors, thereby providing a new avenue for future anticancer drug development.

## Results

### cF and c2 are orally-bioavailable and non-toxic

While hGIIA has a known role in inflammation [5], it was validated as a target for inhibition specifically in PCa using a publicly available dataset. Across a database of 9736 tumours of all tissue types [17], the gene encoding hGIIA (*PLA2G2A*) was most transcribed in PCa and was significantly higher in prostate tumours than healthy tissue (Fig. S1). Previous analysis of the Gene Expression Omnibus (GEO) database found *PLA2G2A* was one of the top five most over-expressed genes in enzalutamide-resistant cell lines compared to enzalutamide-sensitive tumour cell lines [18]. Together, these findings provide correlative evidence that aberrant hGIIA expression may be associated with PCa progression and resistance to therapy.

**Fig. 1:**
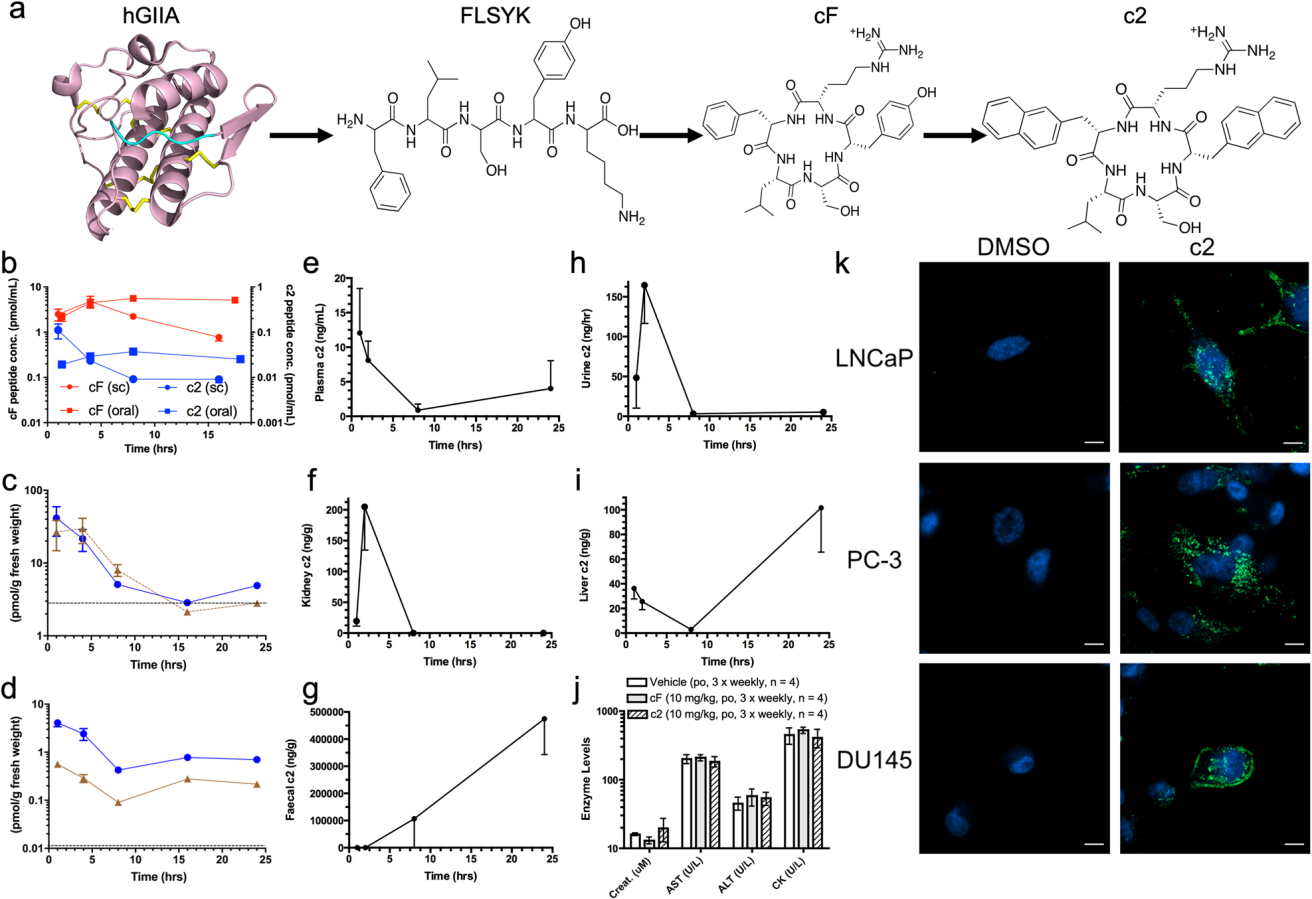
Derived from the structure of hGIIA, cF and c2 are orally absorbed, cell permeable and non-toxic. **a** Structure of hGIIA showing disulphide bonds (yellow) and the location of the peptide sequence ^70^FLSYK^74^ (cyan), which was cyclised to produce pentapeptide cF, then modified to produce c2. **b** Plasma concentrations of tritiated cF (red) and c2 (blue) following subcutaneous (sc) administration (circles) or oral (po) administration (squares) at 5 mg/kg to BALB/c mice (mean ± SE, *n* = 4 per time point). Tissue concentrations of **c** cF and **d** c2 following sc administration determined in the liver (blue) and kidney (brown) (mean ± SE). Dashed lines indicate estimated background levels of tritium in tissues due to tissue blood content (10% of peak plasma concentration). **e**–**i** Following po administration of c2 at 100 mg/kg to male rats (*n* = 5 per group), levels of c2 were measured in **e** plasma, **f** kidney, **g** faeces, **h** urine and **i** liver at timepoints indicated *via* LCMS/MS (mean ± SE). **j** Serum enzyme levels of markers of kidney function (creatinine), liver damage (AST and ALT) and heart damage (CK) in PCa xenograft model mice in response to treatment with vehicle (po, 3 x weekly for 6.1 ± 1.6 weeks), cF (10 mg/kg, po, 3 x weekly for 5.4 ± 0.9 weeks) or c2 (10 mg/kg, po, 3 x weekly for 4.8 ± 0.8 weeks) (*n* = 4, mean ± SE). Data were obtained by the South Eastern Area Laboratory Services Pathology Laboratory at Prince of Wales Hospital, Randwick, Australia. **k** PCa cell lines LNCaP, PC-3, and DU145 cells incubated with 100 µM c2 (green) for 24 hours with DAPI (blue), scale bar 10 µm (representative cells of *n* = 3 biological replicates, 5 cells per replicate).

Through structure-based drug design approaches, we developed novel peptides cF and c2, derived from the primary sequence of hGIIA, (^70^FLSYK^74^, Fig. 1a) [19, 20]. The linear peptide FLSYK was initially shown to modestly inhibit hGIIA enzyme activity. The potency of inhibition was increased through cyclisation first to cFLSYK and to its analogue cFLSYR (cF) followed by amino acid substitution experiments to produce c((2-Nal)LS(2-Nal)R) (c2). Importantly, both c2 and cF suppress the synthesis of exogenous hGIIA-mediated prostaglandin (PG) production in fibroblast-like synoviocytes derived from patients with rheumatoid arthritis by selectively inhibiting the catalysis-independent function of hGIIA, confirming their anti-inflammatory effect [14].

Pharmacokinetic studies following both subcutaneous (sc) and oral (po) administration of tritiated cF and c2 to BALB/c mice found both compounds were orally bioavailable. cF was detectable in plasma at ~10-fold higher concentration than c2 on administration by either route. Area under the curve (AUC) analysis indicated that c2 and cF had ~10% and 20% oral bioavailability at 16 hours respectively relative to sc delivery (Fig. 1b). Administration of cF and c2 resulted in systemic uptake of both compounds in the liver and kidney, with cF at ~10-fold higher concentrations than c2 (Fig. 1c and d, respectively) and to a lesser extent in heart, lung and muscle (Fig S2a, b). Integrity of ^3^H-peptides in vivo was validated by thin-layer chromatography of liver extracts from treated animals (Fig. S2c). Subsequent pharmacokinetic investigation of c2 oral bioavailability in Sprague Dawley rats identified its presence in the plasma, kidney and liver, peaking in the urine after 2 h with the majority excreted in faeces as expected (Fig. 1e–i).

A key failing of previous enzymatic inhibitors of hGIIA was toxicity, resulting in increased incidence of myocardial infarction in a phase III clinical trial [13]. Thus, the toxicity profile of novel hGIIA inhibitors is critically important in evaluating new inhibitors. No gross toxicity of c2 and cF was identified at max dosages of 10 mg/kg thrice weekly for 10 weeks in mice, with no observable damage to organs and no significant changes in serum creatinine, aspartate aminotransferase, alanine aminotransferase or, importantly, creatine kinase enzyme levels which indicate heart tissue damage (Fig. 1j). A comprehensive 28-day repeat-dose oral toxicity study in Sprague Dawley rats treated with vehicle, or c2 at either 1 mg/kg (low dose) or 20 mg/kg (high dose) every three days and analysed according to the OECD 407 protocol (Appendix 1), established no observable toxicity by any measure (mortality, daily clinical observations, weekly body weights, weekly food consumption, weekly detailed clinical examinations, functional observations, haematology, biochemistry, urinalysis, organ weights, gross necropsy on day of sacrifice). Extensive histopathology was performed on all animals in the control and high dose groups sacrificed on day 29, with no difference observed between the treated groups and the vehicle control group by any measure. These data further support the safety of oral administration of c2. In light of the toxicity associated with the myocardial infarction study, data from this c2 toxicity study relevant to heart toxicity are shown in Table 1. Heart wet weight was not affected by either low or high dose relative to vehicle, indicating the compound has no significant pro-hypertrophy effect at these doses. The lack of effect of c2 on gross necropsy or histopathology scores also supports the preclinical safety of the compound in the heart. Importantly, c2 has no effect on serum potassium levels or the Na/K ratio, since hypokalemia (low potassium) is highly arrythmogenic [21]. As seen in the mouse study (Fig. 1j), serum creatine kinase levels are not affected by c2 on repeat dosing, confirming no evidence of heart muscle damage by this measure. It is of interest also that serum alanine transaminase is unaffected by c2 at the doses tested, since this marker was raised three-fold over the normal range in 6.3-fold more patients on varespladib than placebo in the VISTA acute coronary syndrome trial of this sPLA_2_ inhibitor [13].

**Table 1:** Indicative measures of 28-day heart toxicity of c2 on oral delivery to Sprague Dawley rats.

Parameter	Vehicle (*n* = 10)	c2 (1 mg/kg, *n* = 10)	c2 (20 mg/kg) *n* = 10
Heart weight on Necropsy (g)	1.278 ± 0.1020*	1.211 ± 0.1348	1.322 ± 0.1050
Heart weight (% body weight)	0.32 ± 0.018	0.31 ± 0.026	0.34 ± 0.025
Gross necropsy^&^	NA^#^	NA	NA
Histopathology^%^	NA	NA	NA
**Serum biochemistry**			
K (mM)	3.83 ± 0.177	3.59 ± 0.137	3.66 ± 0.295
Na/K ratio	37.9 ± 1.78	40.5 ± 1.59	39.9 ± 3.16
CK (IU/L)	169 ± 91.4	164 ± 47.4	140 ± 34.4
ALT (IU/L)^$^	69.8 ± 21.83	61.1 ± 15.53	64.7 ± 19.01

^*^Data are mean ± SD. ^&^detailed inspection of the thoracic cavity and heart for macroscopic changes. ^#^NA: No observable abnormalities attributable to treatment. ^%^All tissue sections (*n* = 3 per specimen) were stained with hematoxilin and eosin, examined by light microscopy, scored and findings recorded by a qualified pathologist. ^$^While ALT is regarded as a measure of liver toxicity, it is included here since ALT levels were raised 3-fold over the normal range in 6.3-fold more patients on varespladib than placebo in the VISTA acute coronary syndrome trial of the sPLA_2_ inhibitor [13]. *K* serum potassium, *Na/K* ratio, serum sodium/potassium ratio, *CK* serum creatine kinase, *ALT* serum alanine transaminase.

### c2 is fluorescent and cell permeable

The naphthyl groups in the structure of c2 give the compound a unique autofluorescence emission spectral signature, which was characterised (Fig. S3a, b), allowing the direct imaging of c2 internalisation and localisation. To test cell permeability, c2 was added to PCa cell lines LNCaP, PC-3 and DU145 for 24 hours and live cells were confocally imaged using 488 nm excitation and emission captured at 490-620 nm. c2 was detected in all cell lines with a punctate localisation pattern, while DU145 also showed increased c2 localisation on the cell surface (Fig. 1k). Internalisation confirmed *via* z-stack (Fig. S3c).

While we attempted to demonstrate that c2 was internalised in tumours, these experiments were inconclusive due to interference by the broad autofluorescence profile of human tissue (typically spanning 500 nm–650 nm) coupled with the relatively low concentrations of c2 administered in vivo. Our earlier work [7], localising hGIIA in human prostate tumours, showing widespread staining of hGIIA in tumour cells, coupled with our demonstration that c2 and hGIIA are internalised by the same mechanism in this work, supports our conclusion that c2 is cell-permeable and further supports its potential for intracellular activity.

### cF and c2 reduce tumour volume and increase animal survival in xenograft models

**Fig. 2:**
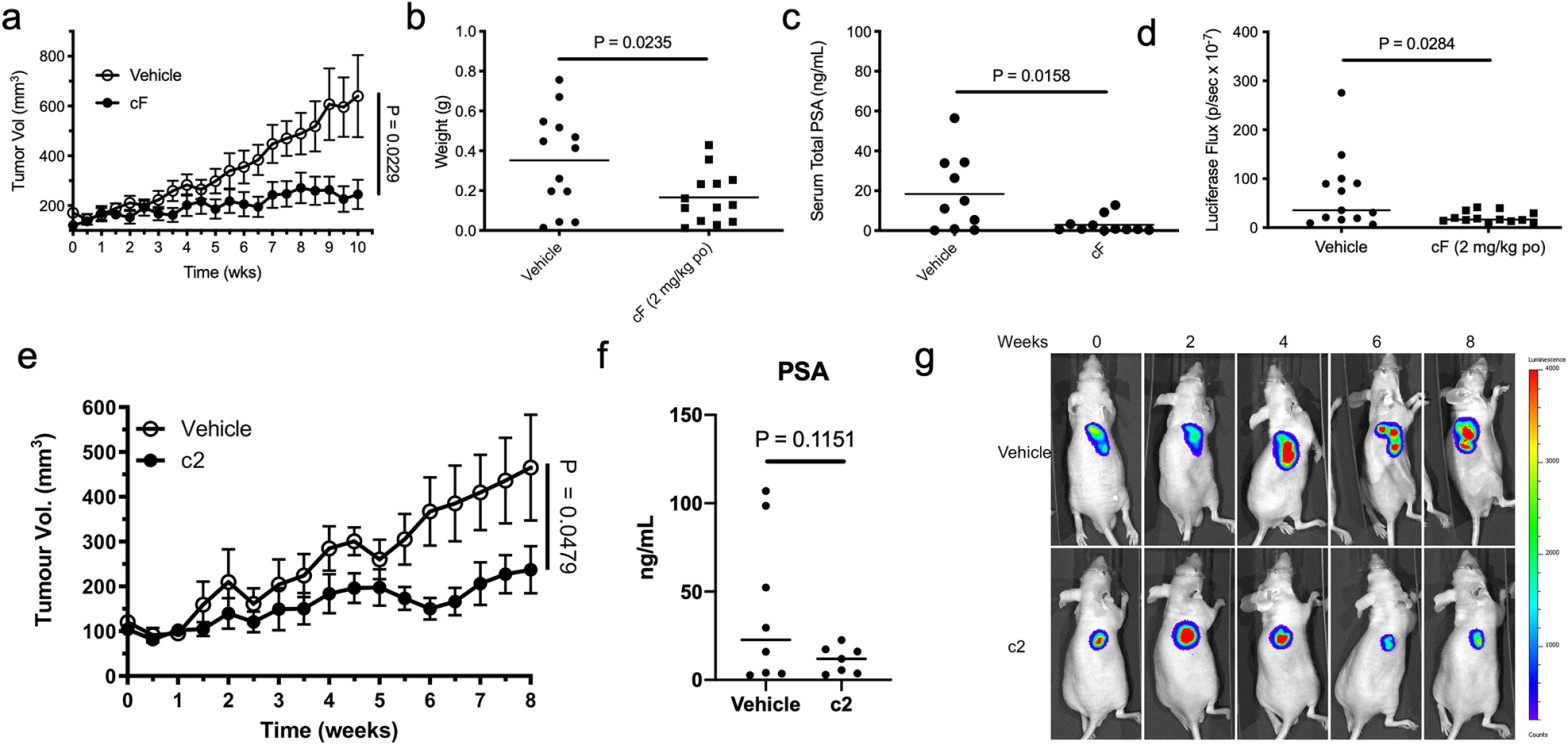
Oral administration of cF and c2 reduces tumour volume in an androgen sensitive xenograft model of PCa. Following sc injection of 1 × 10^6^ androgen-sensitive LNCaP-luc cells, mice with established tumours (>65.5 mm^3^) were treated with **a**–**d** either cF (2 mg/kg, po, daily, *n* = 13) or vehicle (*n* = 13), or **e**–**g** either c2 (0.1 mg/kg, po, thrice weekly, *n* = 7) or vehicle (*n* = 8) by oral gavage. **a**, **e** Tumour volume (mean ±SE), **b** tumour weight and **c**, **f** PSA levels (mean, end of study), **d** luciferase signal (mean, end of study) and **g** representative luciferase signal of tumours.

The efficacy of cF was first evaluated in a luciferase-tagged LNCaP-luc xenograft model, a known model of androgen-dependent PCa [22]. Once tumours were established, animals were treated po daily for 10 weeks with either vehicle or cF (2 mg/kg, daily). cF administration resulted in significant suppression of tumour volume (Fig. 2a, *p* = 0.0229; two-way ANOVA) and tumour weight (Fig. 2b, p = 0.0235, Student’s unpaired two-tailed *t*-test) relative to vehicle-treated animals over ten weeks. Prostate-specific antigen (PSA) levels were significantly reduced with cF treatment (*p* = 0.0158, Student’s unpaired two-tailed *t*-test) (Fig. 2c). Luciferase signal was also reduced (*p* = 0.0284, Student’s unpaired two-tailed *t*-test) (Fig. 2d).

The efficacy of c2 was also investigated in the same androgen-sensitive model with animals treated po thrice weekly with vehicle control or c2 (0.1 mg/kg, thrice weekly). c2 significantly reduced tumour volume, measured over eight weeks (Fig. 2e, *P* = 0.0479, mixed effects analysis) which coincided with a non-significant trend to reduction in PSA, (Fig. 2f, *p* = 0.1151, two tailed Student’s *t* test), as well as a non-significant trend to reduction in luciferase signal (Fig. 2g). Tumour tissues sections evaluated for apoptotic cells using the TUNEL assay showed a non-significant trend toward increased apoptosis. Immunohistochemical tissue staining showed non-significant trends to reduction in proliferation (Ki67) and increase in blood vessel number (CD31 staining), while vimentin and hGIIA staining was unchanged (Fig. S4a).

**Fig. 3:**
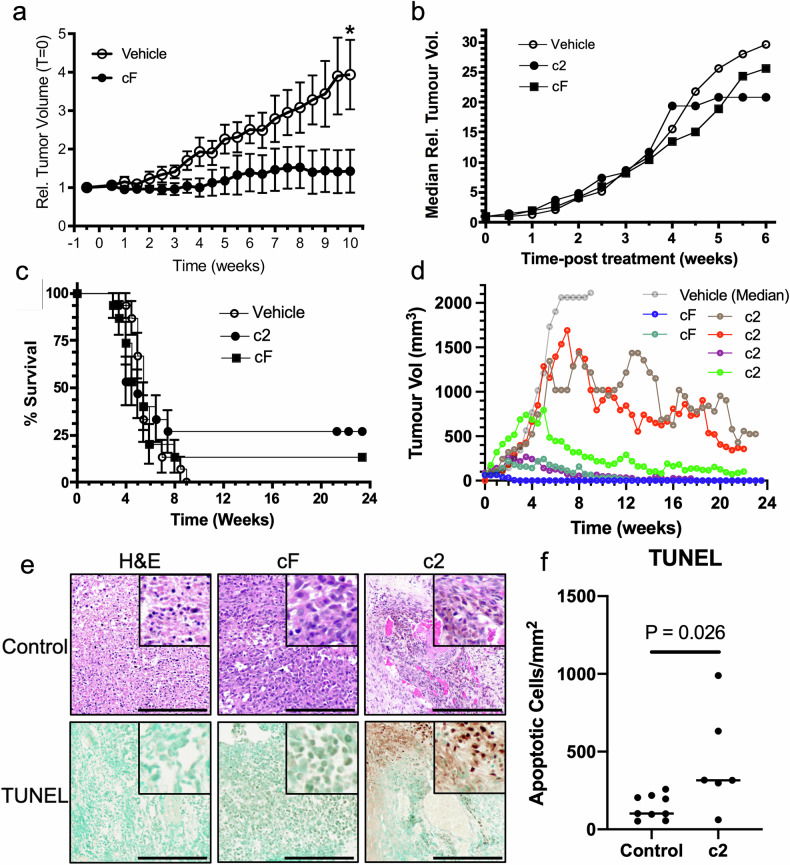
Oral administration of cF and c2 increases animal survival and tumour apoptosis in an androgen-insensitive model of PCa. **a** Castrate-resistant model of PCa: Mice with established tumours (>65.5 mm^3^) were castrated 2-6 weeks following sc injection of androgen-sensitive LNCaP-luc cells (tumour vol. median 164.6 mm^3^, range 74.6 mm^3^–378.4 mm^3^), treated by oral gavage (po) daily with vehicle (open circles, *n* = 10) or cF (closed circles, 2 mg/kg, *n* = 9) and tumour volume was measured over 10 weeks. Tumour volume on treatment commencement (T = 0); vehicle, median 106.5 mm^3^, range 67.5 mm^3^–623.9 mm^3^; cF, median 165.3 mm^3^, range, 60.7 mm^3^–526.3 mm^3^. Data are expressed relative to T = 0 and are mean ± SE. *, *p* < 0.05, Student’s unpaired two-tailed *t*-test of 10-week time point. **b**–**f** Androgen-receptor independent model of PCa. Following sc injection of androgen-receptor independent aggressive cell line PC-3M-luc (1 × 106 cells), mice with established tumours (>65.5 mm^3^) were treated with c2 (1 mg/kg, po, thrice weekly, *n* = 15) cF (2 mg/kg, po, daily, *n* = 15) or vehicle (*n* = 15). **b** Median tumour volume relative to T = 0, **c** survival curve of c2 (*n* = 4 survivors) and cF (*n* = 2 survivors) and **d** time course of tumour volume of animals that survived relative to median tumour volume of the vehicle-treated group (c2 *n* = 4, cF *n* = 2). **e** Randomly chosen representative IHC staining of tumours with H&E and TUNEL staining. (full data set in Fig. S4b). Scale bar 250 µm, inset 100 µm^2^. **f** TUNEL staining of cells showing an increase in apoptotic cells with c2 treatment (*n* = 6) compared to control (*n* = 9, student’s unpaired t-test).

The therapeutic effect of cF was also tested in a castrate-resistant model of PCa, where animals were injected with androgen-sensitive LNCaP-luc cells until tumours formed (>65.5 mm^3^), then castrated 2-6.6 weeks post tumour establishment. Treatment with vehicle or cF (2 mg/kg, po, daily) commenced five weeks post-castration and continued over 10 weeks. At the end of the 10 - week period, cF treatment significantly reduced relative tumour volume from time 0 at 10 weeks (*p* = 0.0419, Fig. 3a). c2 and cF efficacy was also investigated in an aggressive androgen-independent model of PCa. Mice bearing an established tumour derived from an androgen-receptor independent cell line PC-3M-luc were orally administered either vehicle control, c2 (1 mg/kg, thrice weekly) or cF (5 mg/kg, daily) for up to 24 weeks. Both c2 (*p* < 0.001, one-way ANOVA, Bonferroni’s multiple comparison test) and cF (*p* < 0.05), treatment significantly slowed the growth of tumours relative to vehicle controls, improving animal survival (Fig. 3b and c). Several animals (c2, *n* = 4 cF, *n* = 2, 27% and 13% of animals, respectively) survived until study termination at 6 months (Fig. 3c), while all vehicle-treated animals were euthanised due to excessive tumour size by nine weeks (Fig. 3c, d). Of six survivors, three showed complete tumour regression by three months, with the remaining three survivors showing a decline from peak tumour volume over six months of treatment (Fig. 3d). Furthermore, while vimentin and hGIIA expression showed no significant change (Fig. S4b), c2-treated mice had significantly increased TUNEL signal (*p* = 0.026, unpaired t-test), indicating an increase in tumour cell apoptosis (Fig. 3f). Interestingly, this was not seen with cF treatment (Fig S4b). cF treatment resulted in a significant increase in the proliferative marker Ki67 relative to control, an unexpected result, that warrants further study. Increased Ki67 expression in the androgen-independent treated tumour may indicate potential synergistic effects if used in combination with standard of care chemotherapy. Ki67 is a prognostic marker in many cancers, including prostate and a predictive marker of response to chemotherapy in some, notably breast cancer [23].

Taken together, these data indicate that both cF and c2 were therapeutically effective in suppressing tumour growth in multiple xenograft models, representing early stage androgen-dependent, castrated and androgen-independent PCa. Subsequent experiments to determine the mechanism of action used c2 as it reduced tumour volume at concentrations up to 20 times lower than cF.

### Both autocrine and paracrine provision of hGIIA results in internalisation and is inhibited by c2

**Fig. 4:**
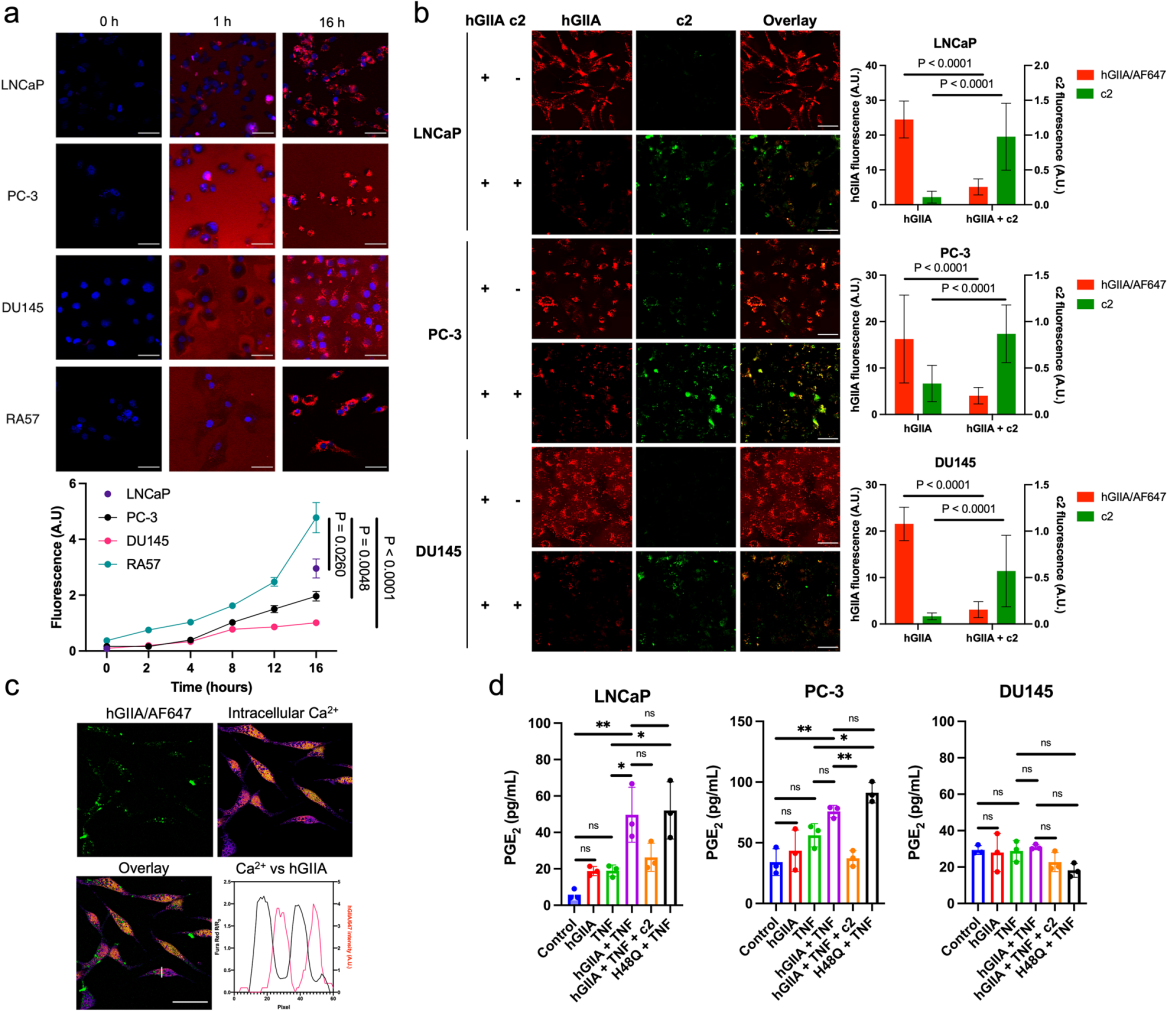
hGIIA internalisation follows both autocrine and paracrine pathways, promotes eicosanoid production via a catalysis-independent mechanism and is inhibited by c2. **a** PCa cell lines (LNCaP, PC-3, and DU145) and fibroblast-like synoviocyte cells (RA57) were incubated with 200 ng/mL hGIIA/AF647 (red) and live cells imaged over 16 hours (*n* = 3, 5 cells per replicate, scale bar 50 µm). Statistical significance using 16 hour data was determined by one-way ANOVA; *p*-values as indicated. **b** PC-3, LNCaP, and DU145 cell lines incubated with hGIIA/AF647 (red) at 2 µg/mL with and without c2 (green) at 100 µM for 24 hours before live cell imaging (*n* = 3 biological replicates, 15 cells per replicate, scale bar 50 µm) with changes in fluorescence quantified (Student’s unpaired *t*-test). **c** Live LNCaP cells incubated with hGIIA/AF647 (200 ng/mL, 24 hours) and intracellular Ca 2^+^ concentration imaged with Fura Red AM (*n* = 3 biological replicates, 20 cells per replicate, scale bar 50 µm). **d** PGE_2_ production by PCa cell lines PC-3, LNCaP and DU145 in response to treatment with TNF at 10 ng/mL, hGIIA at 2 µg/mL, catalytically inactive hGIIA mutant H48Q at 2 µg/mL, or c2 at 50 µM for 16 hours (*n* = 3, one-way ANOVA).

To determine whether c2 functions as a hGIIA inhibitor, we first investigated hGIIA in PCa cells. hGIIA is known to be expressed by innate immune cells such as macrophages and can induce its own expression *via* positive feedback loops [6, 24, 25]. As a model of paracrine effects of extracellular secreted hGIIA on PCa cells, hGIIA tagged with Alexa Fluor 647 (hGIIA/AF647), was incubated with PCa cell lines androgen-sensitive LNCaP, androgen-insensitive PC-3, androgen-insensitive DU145 or, as a positive control, rheumatoid arthritis patient-derived fibroblast-like synoviocyte RA57 cells which have previously been observed to sequester hGIIA [14]. Live cells were imaged over 16 hours. hGIIA/AF647 was detectable on the surface of cells as aggregates in LNCaP and PC-3 cell lines after 1 hour, with a more diffuse surface localisation observed on DU145 cells (Fig. 4a). After 24-hour incubation, hGIIA/AF647 signal was detected in both the hGIIA-positive cell lines LNCaP and PC-3, as well as the hGIIA-negative cell line DU145 ^7^, implying that hGIIA can be internalised on secretion by cancer cells (autocrine recycling) or on secretion by tumour microenvironment cells (paracrine uptake). Quantification of the fluorescent signal over time demonstrated continual accumulation of hGIIA/AF647 over a 16-hour period with sequestration rates as follows: RA57 (4.7 A.U.) > LNCaP (2.96 A.U.) > PC-3 (1.96 A.U.) > DU145 (1.03 A.U.) (Fig. 4a). RA57 cells showed statistically significant accumulation relative to all three PCa cell lines, while differences in accumulation between PCa lines were non-significant (LNCaP v.PC-3, *p* = 0.6230; LNCaP v DU145, *p* = 0.0581; PC-3 v DU145, *p* = 0.7007.

Incubation of PC-3, LNCaP and DU145 cells with both hGIIA/AF647 and c2 followed by quantification of hGIIA and c2 uptake, demonstrated that both protein and inhibitor are internalised. c2 colocalises with hGIIA, thus competitively reducing hGIIA sequestration in all cell lines (Fig. 4b, *P* < 0.0001, student’s unpaired t-test). This is the first evidence that c2 acts to inhibit hGIIA internalisation by entering the cell *via* the same pathway. Furthermore, hGIIA colocalises with caveolin-1 protein (a marker of caveolae) in PC-3 and LNCaP cells (Fig. S5a) and enters the cell *via* a charge-dependent interaction suppressed by protamine sulfate (Fig. S5b), indicating both hGIIA and c2 interact with heparan sulfate proteoglycans (HSPGs) before internalisation *via* caveolae. These studies support the view that the hGIIA internalisation mechanism, binding to heparan sulfate proteoglycans and being internalized via caveolae, first described by Murakami et al. [26] also occurs in prostate cancer cells.

As hGIIA requires millimolar calcium concentration for catalytic activity [11] and previous reports have suggested that hGIIA is able to contribute to eicosanoid production through intracellular catalytic activity in Ca^2+^ rich compartments [26], we next determined the Ca^2+^ concentration in caveolae using the ratiometric dye FURA Red AM, to evaluate whether hGIIA could be catalytically active in PCa cells. We found that in all three PCa cell lines, hGIIA/AF647 signal in the caveolae coincided with a negligible Ca^2+^ concentration (Fig. 4c). Our finding that hGIIA localises in regions of low calcium support our hypothesis that hGIIA effects are a result of a catalytically-independent mechanism.

### hGIIA contributes to eicosanoid production via a catalysis-independent mechanism

hGIIA’s primary contribution to inflammation-driven pathologies is through its increase of cytokine-dependent production of eicosanoids such as PGE_2_ [5]. To investigate hGIIA’s contribution to PGE_2_ production in PCa, cell lines LNCaP, PC-3 and DU145 were grown in serum-free media with hGIIA (2 µg/mL) and inflammatory cytokine tumour necrosis factor (TNF) (10 ng/mL) for 16 hours, before the media was harvested and PGE_2_ concentration detected *via* ELISA. TNF or hGIIA alone did not yield a significant increase in any cell line (Fig. 4d), although a trend to an increase is seen in LNCaP cells. However, in comparison to TNF, co-incubation of hGIIA with TNF resulted in a significant increase in PGE_2_ in LNCaP (*p* = 0.0198, one-way ANOVA), and a non-significant increase in PC-3 cells (*p* = 0.2582). Furthermore, in comparison to TNF treatment, cells co-incubated with the catalytically-inactive mutant of hGIIA known as H48Q (2 µg/mL) and TNF resulted in significant increases in PGE_2_ in both LNCaP (*p* = 0.0120) and PC-3 cells (*p* = 0.0131), indicating that hGIIA upregulates eicosanoid production *via* a catalysis-independent mechanism in these cells (Fig. 4d). Co-incubation of c2 (50 µM) with hGIIA and TNF significantly reduced PGE_2_ levels in PC-3 (*p* = 0.0065) with a trend to decrease in LNCaP (*p* = 0.0940). DU145 did not respond to any treatments (Fig. 4d). DU145’s lack of response may be due to decreased hGIIA and c2 internalisation seen in Fig. 4a and b, however this requires further investigation.

### hGIIA binds to epidermal growth factor receptor (EGFR) and binding is inhibited by c2

**Fig. 5:**
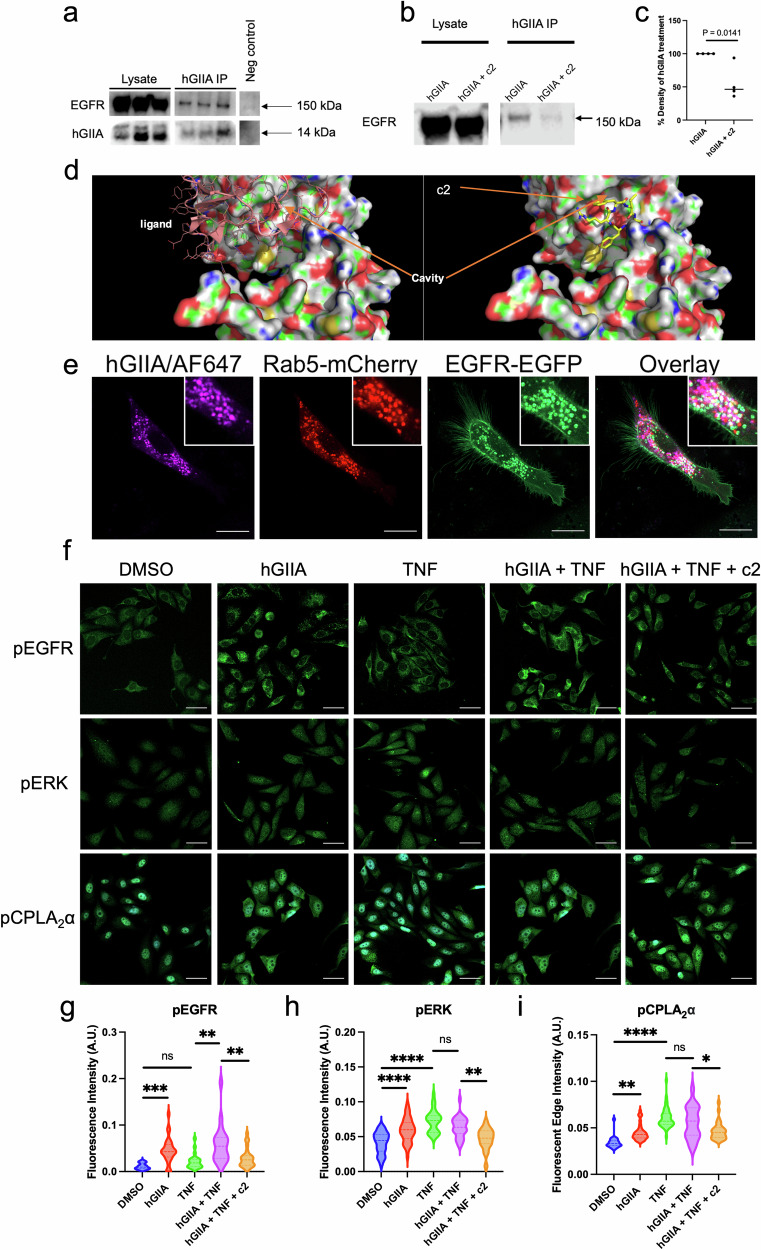
hGIIA binds to EGFR, initiating signalling *via* a catalytically independent mechanism, with binding inhibited by c2. **a** PC-3 cells incubated with hGIIA (2 µg/mL) for two hours and washed before immunoprecipitation of lysates with anti-hGIIA beads (*n* = 3 biological replicates), anti-EGFR beads (*n* = 2 biological replicates, see uncropped Western blots online) or negative control (beads only) overnight, followed by detection of hGIIA and EGFR levels in lysate and Co-IP samples. **b** PC-3 cells were treated with or without c2 (100 µM) for two hours, washed, then incubated with hGIIA (2 µg/mL) for two hours, before immunoprecipitating the lysates with anti-hGIIA antibody and detecting EGFR levels in lysate and Co-IP samples. **c** % of EGFR signal from hGIIA IP treated with hGIIA and with hGIIA + c2 (*n* = 4, student’s unpaired *t*-test). **d** The c2 peptide analogue cFLSFR was docked onto the crystal structure of EGFR (PDB 1nq1) at the ligand binding site using quCBit software. EGFR is depicted as a surface model (red, basic surfaces; blue, acidic surfaces, yellow; cysteine residues; green, hydrophobic surfaces; white, neutral surfaces), while cFLSFR is depicted as sticks. **e** Representative live cell image of a PC-3 cell treated with 200 ng/mL of hGIIA/AF647 (purple) for 24 hours and transfected to express EGFR-EGFP (green) and Rab5-mCherry (red), with colocalization between all three proteins appearing in overlay as white (scale bar 20 µm, inset 20 µm^2^). **f** PC-3 cells treated with combinations of DMSO (0.5%), hGIIA (2 µg/mL), TNF (10 ng/mL), and c2 (100 µM) for 10 mins before fixing and staining with antibodies for either **g** p-EGFR, **h** p-ERK or **i** p-cPLA_2_-α (scale bar 50 µm, n > 20 cells, one-way ANOVA).

hGIIA has been recently identified as a ligand for the epidermal growth factor receptor (EGFR) [6, 15, 16], providing a potential mechanism for hGIIA to initiate ERK phosphorylation, activation of cPLA_2_-α and subsequent eicosanoid production. As c2 is derived from the structure of hGIIA, its ability to potentially competitively inhibit the hGIIA / EGFR interaction was investigated. Lysates from PC-3 cells pre-incubated with c2 (100 µM, 2 hours) followed by hGIIA (2 µg/mL, 2 hours) were immunoprecipitated with anti-hGIIA and anti-EGFR antibody and coimmunoprecipitated EGFR (Fig. 5a. *n* = 3, full blot, Uncropped Western blots) and hGIIA (full blot Uncropped Western blots. *n* = 2)) was detected using western blot, respectively. c2 reduced EGFR signal (*p* = 0.0141, Student’s unpaired t-test, (Fig. 5b, c). Modelling of the c2 analogue cFLSFR binding to EGFR revealed that the lowest energy binding conformation is located close to the ligand binding domain of EGFR (Fig. 5d).

To determine whether an EGFR / hGIIA interaction could be demonstrated in live cells, PC-3 cells were transfected with EGFR tagged with EGFP and the EGFR internalisation regulator Rab5 tagged with mCherry. Exogenous hGIIA / AF647 was added for 24 hours. Imaging of each protein demonstrated colocalisation among all three proteins, shown in white in the overlay (Fig. 5e).

These results raise the possibility that c2 may inhibit hGIIA’s catalysis-independent mechanism of PGE_2_ production through blocking hGIIA binding to EGFR, thereby inhibiting downstream signalling and resulting in decreased PGE_2_ production. To investigate this, PC-3 cells were incubated with DMSO (0.5%), TNF (10 ng/mL), hGIIA (2 mg/mL) and c2 (100 mM), alone or in combination, for 10mins before fixing cells immunofluorescently labelled with either pEGFR, pERK or pcPLA_2_-a antibodies (Fig. 5f–i). hGIIA alone resulted in a significant increase in pEGFR fluorescence (*p* = 0.0003, one-way ANOVA), pERK fluorescence (*p* < 0.0001) and pcPLA_2_-a (*p* = 0.0031). In contrast, TNF alone did not increase pEGFR fluorescence over control (*p* = 0.6714), while significantly increasing pERK (*p* < 0.0001) and pcPLA_2_-a (*p* < 0.0001) fluorescence. In comparison to TNF alone, co-incubation of TNF with hGIIA significantly increased pEGFR signal (*p* = 0.002), with non-significant changes for hGIIA + TNF in pERK and pcPLA_2_-a (*p* = 0.2475 and *p* = 0.3505 respectively). With the addition of c2 to TNF + hGIIA, pEGFR, pERK and pcPLA_2_-a signal was reduced (*p* = 0.0016, *p* = 0.0039 and *p* = 0.0478 respectively). Collectively, these results indicate that hGIIA’s ability to increase PGE_2_ production *via* a catalytically-independent mechanism is through binding EGFR and initiating signalling, which can be inhibited by c2.

### hGIIA binds to vimentin in prostate cancer cells

**Fig. 6:**
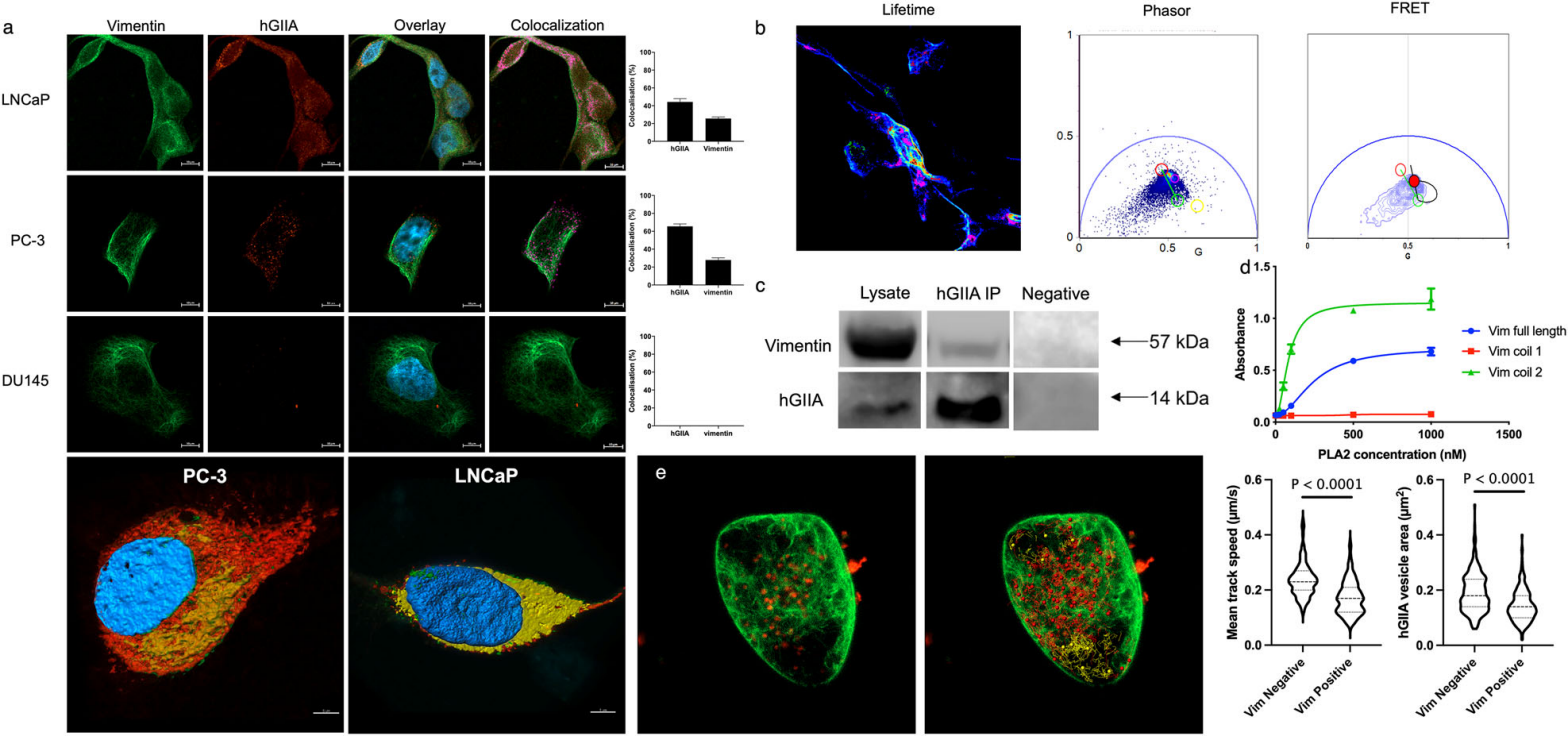
hGIIA binds to and is trafficked by vimentin in PCa cells. **a** Immunofluorescence images of PCa cell lines LNCaP, PC-3, and DU145 cells with vimentin (green), endogenous hGIIA (red), and colocalisation of vimentin and hGIIA (pink) (*n* > 30 cells per cell line). Projections of example cells generated in Imaris from a Z-stack of PC-3 and LNCaP cells, with colocalisation calculated using the Costes et al. (2004) [54] method shown in yellow. **b** Fluorescent lifetime images (left) and phasor plot of fluorescence lifetime (middle) of LNCaP cells immunostained with primary conjugated antibody RV202 / AF488 for vimentin (donor) and 4A1 / AF568 for hGIIA (acceptor), showing areas of FRET (right) (*n* = 3). Circles on the graph show areas for donor signal (red), autofluorescence (green), FRET (purple) and c2 (yellow). **c** LNCaP cell lysate was incubated with anti-vimentin antibody beads overnight, before lysate and immunoprecipitated bead samples were electrophoresed on SDS-PAGE. Vimentin and hGIIA signal was detected by western blot relative to a negative control immunoprecipitation (beads alone). **d** in vitro enzyme immuno-assay of hGIIA binding to coil 1 (red), coil 2 (green), or full length (blue) vimentin fragments (representative of *n* = 3 experiments, standard deviation). **e** Live cell imaging of PC-3 cells following transfection with vimentin-EGFP and incubation with hGIIA / AF568 (200 ng/mL) for 24 hours (left panel), followed by identification of hGIIA-positive vesicles as either vimentin positive (red, *n* = 405 vesicles) or vimentin negative (yellow, *n* = 123 vesicles) (middle panel). Dynamics were tracked over two minutes and mean speed calculated (right panel) with mean track speed and vesicle (spot) area calculated using Imaris 8.2 (Mann-Whitney t-test).

hGIIA has been previously identified to bind to the rod domain of vimentin in apoptotic T-cells [27]. In addition, our previous study in rheumatoid arthritis cells identified colocalisation between the two proteins, raising the possibility that a protein-protein interaction between hGIIA and vimentin may contribute to its catalysis-independent amplification of inflammation since the two proteins colocalise in these cells [14]. To investigate this potential interaction in PCa cells, preliminary immunofluorescence analysis (Fig. 6a) confirmed expression and colocalisation of endogenous hGIIA and vimentin in LNCaP and PC-3 cells, providing further evidence of autocrine internalisation of hGIIA. No expression of hGIIA was detected in DU145 cells, confirming previous results measuring hGIIA mRNA expression [7]. Next, direct binding between vimentin and hGIIA was demonstrated using fluorescence lifetime imaging microscopy (FLIM) to observe Förster resonance energy transfer (FRET), which, unlike immunofluorescence, can determine whether proteins are within 10 nm of each other (Fig. 6b). LNCaP cells show a shift in the lifetime of the donor, indicating FRET with the acceptor, thus establishing localisation of hGIIA and vimentin within 10 nm. This novel interaction between hGIIA and vimentin in PCa cells was confirmed by co-immunoprecipitation of vimentin with hGIIA in LNCaP cells (Fig. 6c). To further characterise this interaction, hGIIA binding to full-length vimentin and specifically, to vimentin’s coil 2, was demonstrated using an in vitro binding assay, with hGIIA showing no affinity for coil 1 (Fig. 6d).

hGIIA-vimentin dynamics were next examined through live cell imaging of PC-3 cells transiently transfected with vimentin-EGFP and incubated with hGIIA/AF568 for 24 hours. Cells were imaged over a two-minute period with a representative cell shown in Fig. 6e. Analysis of hGIIA dynamics using imaging software Imaris (Bitplane) identified two distinct classes of hGIIA-positive vesicles based on colocalisation with vimentin; (i) those tightly associated with vimentin (vimentin positive, red), and (ii) those moving freely in the cytoplasm (vimentin free, yellow). Fluorescent dynamics showed that vimentin-associated hGIIA-positive vesicles exhibit restricted movement, with slower average speed and smaller vesicle size than vimentin-free hGIIA-positive vesicles (Fig. 6e), indicating vimentin acts as a cage. (Movie S1). Further live cell imaging highlights that vimentin also facilitates the movement, fusion, and division of hGIIA-positive vesicles (Movie S2, S3). This novel interaction indicates vimentin modulates intracellular hGIIA movement and localisation, confirming its involvement in hGIIA’s intracellular interactions.

### c2 blocks hGIIA/vimentin interaction, modulating vimentin cellular architecture and hGIIA trafficking

**Fig. 7:**
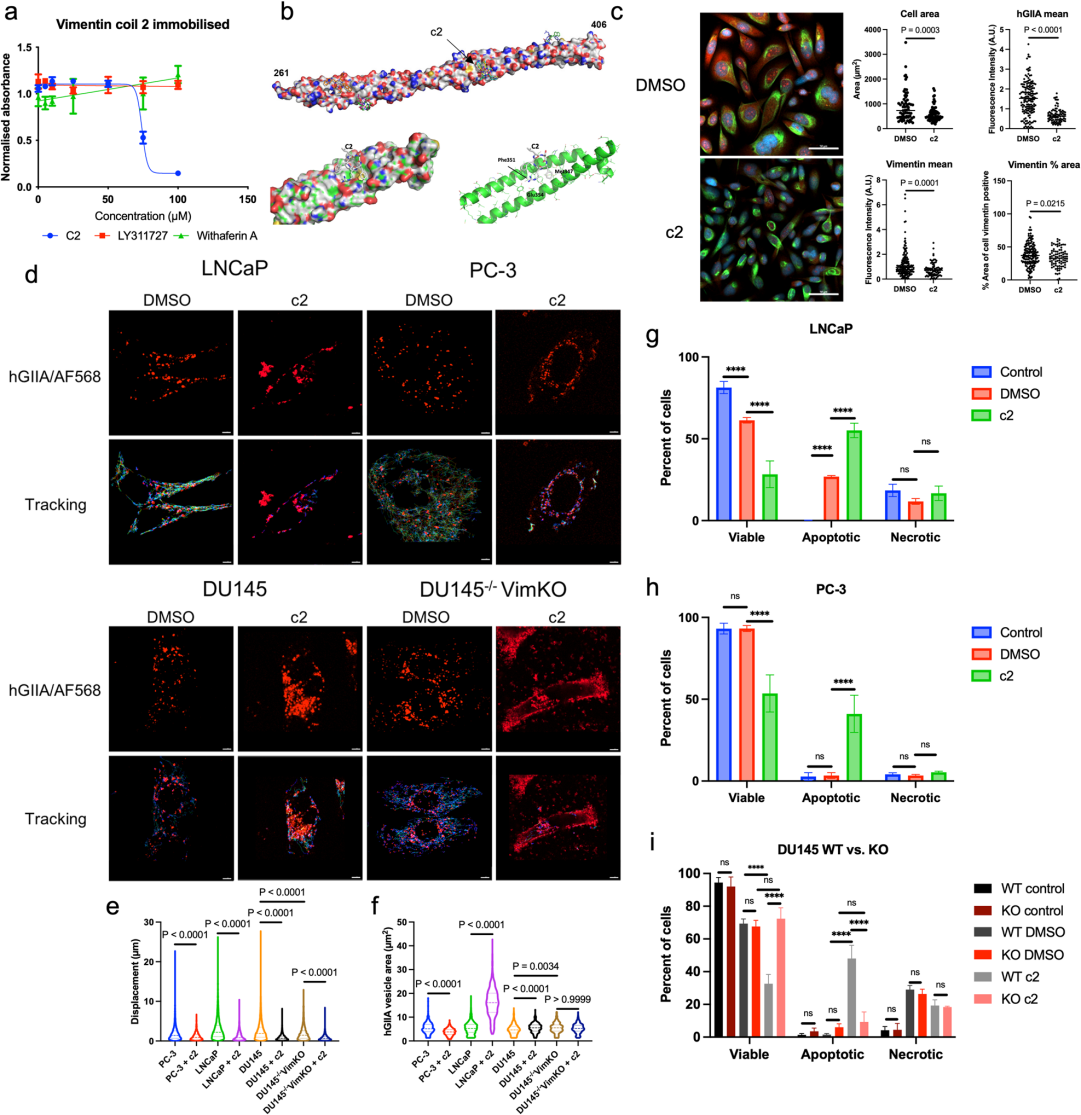
c2 blocks hGIIA interaction with vimentin in vitro and in vivo, modulating hGIIA trafficking and vesicle size. **a** In vitro enzyme imunoassay of hGIIA binding to coil 2 of vimentin, showing inhibition of binding in the presence of c2 (blue), but not hGIIA inhibitor LY31127 (red) or vimentin inhibitor Withaferin A (green). **b** Molecular docking studies identifying binding confirmation of c2 to coil 2 of vimentin. Vimentin coil 2 structures are shown as space-filling or ribbon diagrams. The lowest energy c2 binding mode is indicated and shown as sticks. Docking of c2 on to full vimentin coil 2 structure is shown (top image). Space-filling and ribbon diagram model of the location of the lowest energy conformation of the c2 analogue cFLSFR bound to coil 2 (bottom images). **c** Representative images of PC-3 cells treated with DMSO or c2 (10 μM) for 1 hour and stained with hGIIA (red), vimentin (green) and DAPI (blue). Graphs show quantitation of total cell area, hGIIA mean fluorescence intensity, vimentin mean intensity and vimentin area as % of total cell area (*n* = 3, > 15 cells per replicate, scale bars 50 μm). **d** Imaging of LNCaP, PC-3, DU145 and DU145 vimentin knockout cells (63 x objective) incubated with 200 ng/mL of hGIIA/AF568 with either DMSO or c2 at 100 μM for 24 hours (top panel). Cells were live-imaged for 3 minutes (bottom panel) with dynamics of hGIIA positive vesicles tracked and analysed using Imaris 8.2. Vesicle movement colour coded from 0 μm/s (blue) to 1.5 μm/s (red). Graphs show **e** track displacement, and **f** vesicle area (*n* = 3, 20 cells per replicate, scale bar is 10 μm). **g** FACS analysis of PC-3, **h** LNCaP, **i** DU145 WT vs DU145 vimKO treated with either control (nothing), DMSO (0.5%), c2 (100 µM) for 72 hours stained with Annexin V-FITC and propidium iodide (PI), before FACS analysis. The Annexin V/PI data was measured on FL1-H versus FL2-H scatter plot using FlowJo^TM^ v10.9 software (*n* = 3, 2-way ANOVA).

Given the above findings, we next investigated whether c2 perturbs the hGIIA/vimentin interaction as a potential mechanism of action for inhibiting tumour growth and hGIIA catalysis-independent activity. Firstly, we used an enzyme immunoassay to confirm that c2 inhibits the interaction between hGIIA and vimentin in vitro, with an IC_50_ of 56.5 µM. hGIIA inhibitor LY311727 and vimentin-binding compound withaferin A did not inhibit this interaction (Fig. 7a), indicating that this effect was unique to c2. Next, molecular docking studies predicted that c2 binds to the second coil of vimentin, with the c2 analogue cFLSFR forming well-defined hydrophobic interactions with Met347 and Phe351, respectively, as well as charge-charge interactions with Glu354, indicating that c2 may sterically hinder the hGIIA interaction with vimentin (Fig. 7b). To investigate c2’s effect on cell size and vimentin organisation in the presence of hGIIA, c2 (10 µM) was incubated with PC-3 cells for one hour and endogenous hGIIA and vimentin were imaged *via* immunofluorescence (Fig. 7c). c2 reduced cell size (*p* = 0.0003, Student’s two-tailed unpaired t-test), while also reducing endogenous hGIIA mean fluorescence intensity (*p* < 0.0001) and vimentin mean fluorescence intensity (*p* = 0.0001). However, the vimentin-positive area as a percentage of total cell area was also reduced (*p* = 0.0215) despite a reduction in mean fluorescence intensity. This reduction in vimentin network size indicates that c2 addition results in a change in the organisational state of vimentin known as vimentin bundling.

c2 also greatly inhibited hGIIA trafficking, as co-incubation of hGIIA/AF568 at 2 µg/mL with c2 at 100 µM for 24 hours significantly reduced hGIIA displacement and mean speed in all tested cell lines (*p* < 0.0001, Kruskal-Wallis test) (Fig. 7d–f, Movie S4, S5). The cell-wide hGIIA-positive vesicle movement and fusion and division events under vehicle treatment were almost completely stagnated by c2, with hGIIA localisation identified as predominantly perinuclear. c2 treatment also reduces size of hGIIA-positive vesicles in PC-3 cells, while increasing them in LNCaP and DU145 (Fig. 7d).

Since vimentin is involved in hGIIA trafficking (Fig. 7e) and c2 inhibits the hGIIA/vimentin interaction in vitro (Figs. 7a, b), vimentin targeting by c2 was investigated as the potential mechanism of action for impeding hGIIA trafficking. A vimentin knockout DU145 cell line was constructed using CRISPR-Cas9 (Fig. S6). DU145 cells were selected for CRISPR-mediated vimentin knockout due to their markedly high vimentin expression, which we anticipated would yield a more pronounced phenotype upon knockout. In addition, DU145 cells do not express endogenous hGIIA, which provides a model that could examine the role of vimentin in exogenous addition of hGIIA without the potential confounding influence of endogenous enzyme expression. hGIIA displacement was significantly lower in vimentin knockout DU145 than wtDU145 confirming that vimentin is a major facilitator of exogenous hGIIA trafficking. However, c2 further reduced hGIIA movement in the vimentin knockout to similar levels seen in wt-DU145 cells, indicating that despite the absence of vimentin, c2 is capable of further reducing hGIIA trafficking by a so far unidentified mechanism (Fig. 7e). c2 action is thus likely to be multimodal, suppressing both vimentin-mediated and vimentin-independent trafficking. It is possible that this c2 effect may be mediated through targeting a protein other than vimentin, such as EGFR. EGFR activation is known to trigger its endocytosis, as well as increased trafficking by Rab proteins such as Rab 5 [28].

In contrast to trafficking, hGIIA-positive vesicle area was significantly larger in the vimentin knockout than wtDU145 (*p* = 0.0034, one-way ANOVA), and unlike wtDU145, was unaffected by c2 treatment (*p* > 0.9999) (Fig. 7f). The loss of c2 function in vimentin knockout DU145 confirms that c2 modulates vesicle size through targeting vimentin. c2’s ability to increase vesicle size suggests the induction of aggresome formation, a process that is tightly coordinated by vimentin under stress conditions and associated with the disposal of misfolded proteins [29]. The build-up of aggresomes leads to apoptosis, which was observed in c2-treated tumours (Fig. 3f).

### c2 initiates PCa apoptosis via a vimentin-mediated pathway

To confirm that c2 initiates apoptosis, PCa cell lines PC-3, LNCaP, DU145 WT and vimentin knockout DU145 were treated with either nothing (control), DMSO (0.5%) or c2 (100 µM) for 72 hours. Cells were then analysed following staining with Annexin V-FITC and propidium iodide, markers of apoptosis and necrosis, respectively. In comparison to DMSO, c2 significantly increased the population of apoptotic cells in PC-3, LNCaP and DU145 WT cells (all P < 0.0001, two-way ANOVA) (Fig. 7g–i, scatter plots Fig. S7). However, in DU145 vimentin knockout (KO) cells, there was no significant difference in the number of apoptotic cells between DMSO and c2-treated cells (*P* = 0.7055, 2-way ANOVA) (Fig. 7i). This loss of function of c2 in DU145 KO confirms that c2 causes apoptosis of cells *via* a vimentin-mediated pathway, confirming c2’s multimodal function.

## Discussion

This research identifies a novel molecular pathway for hGIIA in PCa, highlighting that hGIIA’s interactions with EGFR and vimentin contribute to pathology. Furthermore, we present a novel class of inhibitors that selectively inhibit these interactions, providing therapeutic benefit in in vivo models of both androgen-sensitive and androgen-insensitive models of PCa. Extensive in vivo safety testing and pharmacokinetic studies also confirm these compounds are orally effective, non-toxic and cell permeable.

hGIIA is a secreted protein and a known ‘amplifier of inflammation’ that is overexpressed in PCa, and correlates with poorer disease outcome [5, 11]. This study has identified that hGIIA’s role in promoting PCa tumour progression is due to its non-catalytic function rather than its enzymatic activity, which was the primary target of previous failed clinical trials [13, 30]. This non-catalytic function involves increasing the production of eicosanoids such as PGE_2_ as confirmed in PCa cells with the use of a catalytically-inactive hGIIA mutant H48Q (Fig. 4c). We provide plausible evidence that increased PGE_2_ production in these cells is likely through hGIIA binding and activating the EGFR signalling pathway (Fig. 5). In addition, the calcium/hGIIA imaging data established that exogenously added hGIIA is only found in low calcium intracellular environments where it is has no enzymatic activity (Fig. 4c), further supporting a hGIIA catalysis-independent intracellular role. While we have shown the importance of hGIIA’s non-catalytic role, hGIIA may still contribute indirectly to PCa pathology through enzymatic activity, as its metabolite lysophosphatidic acid (LPA), a product of hGIIA-mediated hydrolysis of phosphatidic acid, can stimulate proliferation of PCa cells, and recently, hGIIA’s bactericidal role in the gut has been identified to alter the lipidome, indirectly affecting inflammation and cancer [31, 32].

hGIIA is expressed by macrophages and activated epithelial cells in PCa and can control its own expression *via* multiple feedback loops, through activating and becoming induced by TNF and NF-κB signalling [6, 24, 25, 33]. In our studies, both exogenous and endogenous hGIIA have been observed to enter and activate PCa cells, supporting the view that hGIIA derived from both cancer cells and stromal cells may regulate tumour cell function. Tumour hGIIA may also amplify stromal cell activation in the presence of inflammatory cytokines [33–35], thereby contributing to the cytokine and eicosanoid storm that drives tumorigenesis [36]. Our findings suggest that further exploration of the role of hGIIA in mediating the interplay between stromal and tumour cells within the tumour microenvironment is warranted and demonstrate that hGIIA may provide a molecular link between the innate immune response and PCa.

We also characterise for the first time the anti-cancer effects of novel hGIIA inhibitors, cF and c2. Unlike previous structure-based designed inhibitors, including those trialled in the clinic, c2 and cF are selective for the catalysis-independent function of hGIIA relative to inhibition of catalytic mechanisms [14]. In addition, pre-clinical testing indicates that, unlike most cyclic peptides [37], cF and c2 are orally absorbed, bioavailable, orally effective and cell permeable (Figs. 1–3). c2 is extremely well tolerated with no observable toxicity at high dosage (Fig. 1j, Table 1, Appendix 1), another key difference from other clinically tested hGIIA inhibitors such as Varespladib methyl [13]. Through characterisation of c2’s unique autofluorescence, the compound was observed to colocalise with hGIIA in vesicles (Fig. 4b) in real time, in situ, in living cells. These vesicles stain positively for caveolin-1 a marker of caveolae (Fig. S5a), which is consistent with findings by Murakami and Kudo that demonstrate hGIIA binding to the glycosylphosphatidylinositol-anchored HSPG protein glypican-1, in caveolae or rafts [26, 38]. Furthermore, unlike previous inhibitors [39], c2 was able to enter cells in the absence of hGIIA. c2 inhibited hGIIA sequestration into cells, indicating that c2 enters the cell *via* the same pathway as hGII,A identified to be through a charge interaction with heparan sulfate proteoglycans (HSPG) (Fig. S5b).

Both cF and c2 reduced tumour volume in the androgen-dependent xenograft model (Fig. 2a, e). cF was further tested in the castration-resistant model and demonstrated efficacy (Fig. 3a). In the androgen-independent xenograft model, cF and c2 increased survival and in some cases, resulted in complete tumour regression (Fig. 3b–d). cF and c2 caused tumour regression and increased survival only in some animals (13% and 27%, respectively), notably without measurable side effects. However, the response rate in this study is higher than the response rate for docetaxel, the current standard of care for men with castrate-resistant PCa, (12% tumour response [40]). This tumour response rate reflects the molecular heterogeneity of advanced prostate tumours. These findings indicate that the peptides may be effective in both early-stage and advanced PCa. While both cF and c2 were effective, c2 was far more potent, and effective at concentrations of 0.1 mg/kg thrice weekly, 20 times less than the tested concentration of cF (2 mg/kg), and 400 times less than the maximum observed dosage with no toxicity (40 mg/kg, Appendix 1), highlighting the potential usefulness of c2 as a therapeutic for PCa. While this research highlights two key mechanisms of action for c2, off target effects cannot be ruled out. However, as c2 is confirmed to be non-toxic (Fig. 1j, Table 1, Appendix 1), it is unlikely that any further interactions will cause harm. c2 was also observed to inhibit hGIIA-mediated catalysis-independent eicosanoid production (Fig. 4d), likely through its ability to inhibit hGIIA binding to EGFR (Fig. 5a–c), and subsequent observed activation of pEGFR signalling (Fig. 5f–i).

The suppression of apoptosis by PCa cells is a key mechanism that mediates progression from androgen-dependence to castrate resistance and androgen independence [41–43]. The observation that c2 increases apoptosis in the androgen independent in vivo model and in the two genetically diverse androgen-independent cell lines, confirms that c2 is able to relieve this suppression. In contrast, the androgen-dependent LNCaP xenograft TUNEL assay showed a non-significant trend to increase in apoptosis (Fig S4a). However, the observation that c2 induces apoptosis in the androgen-dependent cell line LNCaP in vitro suggests that induction of apoptosis by c2 may also be possible in early stage (androgen-dependent) tumours. Further in vivo work with higher animal numbers, higher doses of c2 or more sensitive assays will be required to confirm this. Alternatively, androgen is known to suppress apoptosis by suppression of anti-apoptotic genes [44] This may explain why c2 did not show a significant increase in apoptosis in LNCaP androgen dependent tumours as apoptosis may have been already suppressed *via* an androgen-dependent pathway. This pathway is not operable in androgen-independent cells. They do, however, retain machinery that can activate apoptosis by exogenous agents such as c2. Alternatively, LNCaP cells express low levels of vimentin relative to PC-3 cells which mediates c2-induced apoptosis in these cells. It is possible that the signal for induction of apoptosis by c2 is dependent on the level of expression of vimentin and that LNCaP cells fail to reach this threshold in vivo at the dose administered.

A novel interaction for hGIIA in PCa was also elucidated. Through FLIM/FRET analysis, coimmunoprecipitation and in vitro enzyme immunoassay studies, we have discovered that hGIIA directly binds to vimentin in PCa cells, and that this binding has been further localized to coil 2 of vimentin (Fig. 6b–d), further characterising previous reports that hGIIA binds to the rod domain of vimentin in apoptotic T cells [27]. Vimentin, a marker of the epithelial to mesenchymal transition (EMT) is also overexpressed in PCa, however the specific interactions and pro-tumorigenic contributions of the intermediate filament remain largely undefined [45, 46]. The hGIIA / vimentin interaction is a physical link between this tumour cytoskeletal protein and the innate immune response, of which hGIIA is a mediator.

Through live cell imaging, we have identified that vimentin acts bifunctionally to modulate hGIIA intracellular dynamics. Firstly, vimentin acts as a cage (Fig. 6e), binding hGIIA and thereby decreasing its vesicle speed and size. This finding, together with the finding that hGIIA colocalises with caveolae (Fig. S5a) is consistent with previous reports that vimentin inhibits caveolae movement [47, 48]. However, vimentin was also found to be involved with the active transport of hGIIA around the cell (Movie S2, S3), acting as a scaffold protein for hGIIA-positive vesicles to fuse and divide.

The novel mechanism of action of c2 as a ‘vimentin blocker’ was confirmed by our in vitro assays and molecular modelling approaches, which showed that c2 binds to coil 2, blocking interaction with hGIIA (Fig. 7a, b). In PC-3 cells, c2 caused vimentin bundling, demonstrated by a reduction in the % of cell area occupied by vimentin, despite reductions in cell size and in both endogenous hGIIA and vimentin mean fluorescence intensity (Fig. 7c). c2 also greatly inhibited intracellular trafficking of exogenous hGIIA reducing displacement and altering hGIIA vesicle size in all cell lines (Fig. 7d–f). Vimentin knockout DU145 cells were used to determine vimentin’s involvement in the c2-mediated reduction in hGIIA trafficking. Vimentin knockout had no significant effect on hGIIA vesicle displacement with c2 treatment, however unlike wt DU145 there was no increase in hGIIA-vesicle area (Fig. 7e, f) in the absence of vimentin. As vimentin has a well-established role in aggresome formation, c2 may exploit this process, binding to vimentin, causing bundling and aggresome formation resulting in the accumulation of misfolded proteins and subsequent apoptosis of PCa cells [29]. c2’s induction of apoptosis in PCa cells was confirmed via FACS (Fig. 7g-i), and a loss of function in the DU145 knockout confirmed this function is facilitated by vimentin (Fig. 7i). This mechanism is supported by the increased TUNEL staining in the androgen-independent xenograft model, as well as c2’s inability to modulate vesicle size in vimentin knockout DU145 (Fig. 3e, Fig. 7f). This result indicates that vimentin expression, as is commonly seen in metastatic cells that have undergone EMT, is an important requirement for c2 function in prostate cancer cells.

**Fig. 8:**
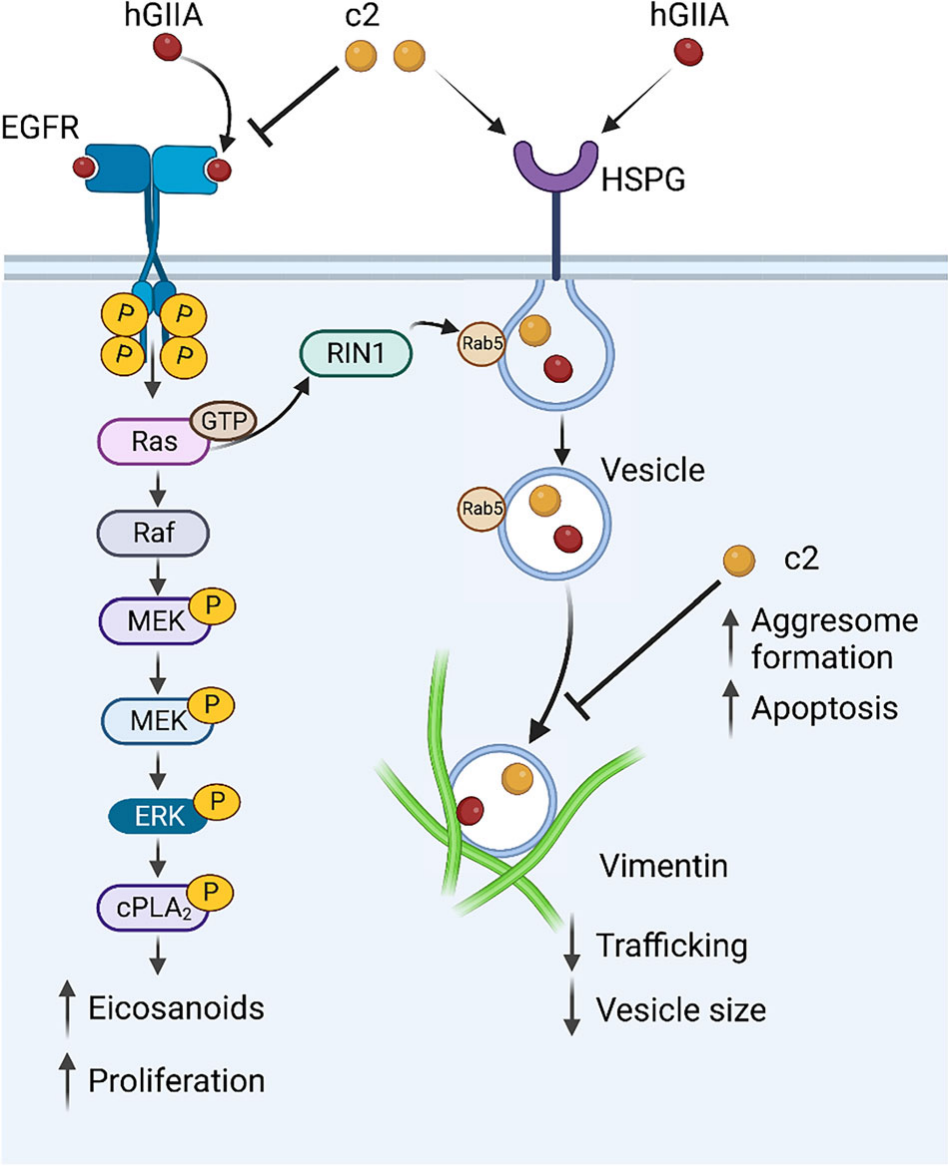
Model of the catalysis-independent function of hGIIA in PCa cells and its multimodal inhibition by c2. hGIIA present in the tumour microenvironment, through either autocrine secretion by cancer cells or by paracrine release from innate immune cells binds tumour cell EGFR, contributing to activation of cPLA_2_-α, thus amplifying cytokine-mediated prostaglandin production and cancer cell proliferation. Concomitantly, hGIIA is internalised *via* caveolae and these vesicles are trafficked intracellularly through binding to vimentin, resulting in decreased vesicle tracking speed and particle size relative to vimentin-unbound vesicles. c2 inhibition of the hGIIA/EGFR interaction reduces cPLA_2_-α activation and eicosanoid production, while slowing hGIIA trafficking within the cell. c2 also binds coil 2 of vimentin, resulting in aggresome formation and apoptosis.

Based on our findings, we propose the following model of the non-catalytic function of hGIIA in PCa and its multimodal inhibition by c2 (Fig. 8). hGIIA binds to EGFR, stimulating cPLA_2_-α activation and eicosanoid production through the Ras/MEK/ERK pathway. Furthermore, hGIIA binding to EGFR may also activate guanine nuclear exchange factor RIN1, a co-activator of Rab5, which promotes endocytosis, including *via* caveolae [28], facilitating hGIIA entry and trafficking. c2 inhibits EGFR activation, thereby reducing both eicosanoid production and intracellular trafficking of hGIIA. On internalisation via caveolae, c2 binds to the second coil of vimentin, resulting in aggresome formation, thereby inducing vimentin-dependent apoptosis. While this research identifies two key mechanisms of action for c2, off-target effects cannot be ruled out. However, as c2 is confirmed to be non-toxic, any further so-far unidentified interactions are unlikely to impact the translational potential of the peptide.

This research highlights that the pro-tumorigenic role of hGIIA in prostate cancer cells is a function primarily of its catalysis-independent mechanism mediated *via* specific protein-protein interactions. Here, we have begun to elucidate a novel function for vimentin in inflammation-driven malignancy in PCa. Finally, we present a novel, first-in-class inhibitor of hGIIA’s non-catalytic function, c2, which exhibits in vivo effectiveness at nanomolar concentrations. Its lack of toxicity and oral effectiveness demonstrate its potential as an anticancer therapeutic, justifying progression to clinical trials.

## Materials and Methods

### Peptides

Peptides were obtained following custom synthesis from either Auspep Pty Ltd., (Melbourne VIC, Australia) or Bachem Ltd., (Bubendorf, Switzerland). Peptides were supplied at > 95% purity.

### Pharmacokinetics

All animal studies were performed according to the guidelines for the ethical use of animals published by the National Health and Medical Research Council of Australia. Mouse pharmacokinetic studies were conducted with approval from the Garvan Institute Animal Ethics Committee (approval #02/21). cF and c2 were radioactively labelled with tritium by catalytic saturation of peptide analogues containing dehydroleucine (Sibtech Inc.), yielding high specific activity (50–60 Ci/mmol) peptides. Their identity was confirmed by three separate analytical methods prior to delivery. Mice (BALB/c, *n* = 4 per timepoint) were administered a single dose of peptide by the routes and times indicated. Animals were then exsanguinated by cardiac puncture under halothane anaesthesia followed by cervical dislocation. Tissues and plasma were stored at −80 °C prior to analysis. For plasma, a known volume of plasma was added to liquid scintillant (Ultima Gold, Perkin Elmer). For tissues, a known amount of tissue (50–100 mg) was incubated in NaOH (1 mL, 1 M) overnight at room temperature. The solutions were then bleached with disodium EDTA (100 µl, 0.1%) and hydrogen peroxide (300 µl, 30%) and incubated at 50 °C for one hour. The peptide content of plasma and tissues was determined by liquid scintillation counting. The stability of the peptides following administration to animals was evaluated by thin-layer chromatography of tissue-extracted peptide relative to peptide prior to injection (Fig. S2c).

Rat studies were performed under contract to the accredited animal research establishment ICP Firefly Pty Ltd. (Alexandria, NSW, Australia; Ref. No AW96/042, NSW Dep’t Primary Industries) with ethics approval from the ICP Firefly animal ethics committee. Briefly, male rats (Sprague Dawley, *n* = 5 / group) were treated with c2 (single dose 100 mg/kg suspended in 0.1% carboxymethylcellulose, po) and housed in metabolic cages. Animals were sacrificed at 1, 2, 8 or 24 h, blood and tissue samples taken for further analysis, with urine and faeces collected for 1, 6 and 16 hours respectively. Following extraction, c2 concentration in plasma was measured using a validated LC MS/MS assay (limit of detection 7.5 ng/mL and lower limit of quantitation 15 ng/mL in plasma, accuracy 98% and imprecision 12.7% at 10 ng/mL).

### Xenograft models

Xenograft model studies were conducted with the approval of the University of New South Wales Animal Ethics Committee (approval 17/92 A).

#### Androgen-sensitive xenograft model

BALB/c *nu/nu* mice (which express murine hGIIA) (n = 13 per group) were injected subcutaneously with 1 × 10^6^ luciferase-tagged LNCaP cells (LNCaP-luc) in RPMI 5% FBS/Matrigel (1:1). Subcutaneous tumours (> 65.5 mm^3^), in mice randomised to two groups, were treated by gavage with either vehicle (saline, 1% dimethylformamide (DMF), po, daily) cF (2 mg/kg po, daily) or c2 (0.1 mg/kg, thrice weekly). Mouse weight and tumour diameter, ((d1) and at 90 degrees to d1 (d2) measured with calipers) were recorded twice weekly. Diameter measurements were used to calculate tumour volume; Vol. = π/6 (d1.d2)^3/2^, with treatments blinded to experimentalists. Animals were treated for 8-10 weeks unless culled due to tumour volume reaching 15 x 15 mm (1768 mm^3^). End of study plasma PSA levels were measured by ELISA. Bioluminescence images were obtained weekly using the IVIS® Lumina (Perkin-Elmer). D-Luciferin, Firefly, potassium salt (150 mg/kg), (Gold Biotechnology Inc.) substrate was administered intraperitoneally prior to imaging. Images were acquired after 10 min with an image time of 60 s, medium binning, F/Stop=1. Images were quantified using the Living Image software (Perkin-Elmer).

#### Castrate-resistant model of PCa

Mice (BALB/c *nu/nu*) carrying LNCaP-luc xenograft tumours ( > 65.5 mm^3^) were surgically castrated 2.0 - 6.6 weeks post-tumour establishment. Treatment was commenced five weeks post-castration and the study terminated 10 weeks post-treatment commencement.

#### Androgen-receptor independent xenograft model

Mice (BALB/c *nu/nu*) carrying luciferase-tagged PC-3M cells (PC-3M-luc) were each injected sc with 1 × 10^6^ cells suspended in RPMI culture medium. Once tumours were established (> 65.5 mm^3^), animals were treated with ether cF (2 mg/kg, po, daily), c2 (1 mg/kg, po, thrice weekly) or vehicle (saline, 2.5% DMF, po, thrice weekly) administered over a period of 8-24 weeks. Tumour volume and animal weights were measured twice weekly.

### Immunohistochemistry

Tumours were fixed with 10% neutral buffered formalin and stored in 70% ethanol, then paraffin-embedded and cut into 5 µm consecutive sections. Samples were deparaffinised with xylene and dehydrated with ethanol using standard protocols, with antigens retrieved by incubating samples in citrate buffer (pH 6) at 95 °C for 20 min. Samples were then incubated with 3% H_2_O_2_ in methanol for 30 min to remove endogenous peroxidases, then blocked with 10% goat serum in PBS for 30 min. Samples were then incubated overnight at 4 °C with a range of antibodies: Ki67 (Abcam, ab15580, 1:100), vimentin (1:333), CD31 (Abcam, ab28364, 1:50) and hGIIA (1:100) (Cayman Chemical, Ann Arbour, MI). TUNEL assay (Abcam) was also used to identify apoptosis. Samples were then washed, stained with Rabbit IHC secondary antibody (1:100) (Dako, Glostrup, Denmark) and counterstained with Gill's haematoxylin (1:5) (Dako). Samples were imaged using an M8 digital microscope (PreciPoint GmbH, Freising, Germany) and DAB staining was scored in a blind manner using ImageJ as described previously [49].

### Preclinical safety studies

Potential toxicity of c2 was evaluated with a 28-day repeated dose oral toxicity study (OECD 407) [50] in male Sprague Dawley rats, with a recovery period. Animals (*n* = 10 per group) were treated with vehicle (1% carboxymethylcellulose in distilled water), c2 at 1 mg/kg or at 20 mg/kg every three days for 28 days. Animals were sacrificed on day 29 and blood and tissues were taken for further analysis. Two recovery groups treated concomitantly with the main study (*n* = 5, vehicle of 20 mg/kg c2) were monitored for a further 14-day treatment-free period, sacrificed on Day 43 and blood and tissues taken for analysis. (Data available on request).

### Cell lines

PCa cell lines PC-3, LNCaP, DU145 were a gift from Professor P. Russell (Queensland University of Technology, Brisbane, Australia). Patient-derived fibroblast-like synoviocyte cell line RA57 was used as a control cell lines [51]. PC-3 was grown in RPMI (Lonza, Basel, Switzerland), LNCaP in DMEM (Lonza), DU145 in MEM (Sigma-Aldrich, St. Louis, MO), and RA57 in DMEM-F12 (Lonza) in complete growth medium, all with 10% FCS, 4 mM L-glutamine (Sigma-Aldrich) and 1% penicillin and streptomycin (Sigma-Aldrich). Cells were incubated at 37 °C and 5% CO_2_, and sub-cultured when the culture neared confluency. Identity of cancer cell lines was confirmed by short tandem repeat (STR) analysis from the Australian Genomic Research Facility (AGRF). Cells were used between passages 2 and 30. All cell lines were mycoplasma negative following regular routine testing using the mycoplasma detection kit (Lonza) as per the manufacturer’s instructions.

### Vimentin knockout

gRNA oligos designed for the insertion into exon 1 of VIM gene using the selection tool CRISPOR [52]. ‘CACCGGAGGACGAGGACACGGACC’ and ‘AAACGGTCCGTGTCCTCGTCCTCC’ were annealed, phosphorylated and then ligated to pSpCas9(BB)-2A-GFP (PX458) (Addgene, Watertown, MA) for the creation of sgRNA. DU145 cells were grown to 80% confluency in a 6-well plate, then transfected with 0.2% Lipofectamine 3000 reagent. After 72 hours, cells were trypsinised, resuspended in PBS and GFP-positive cells were sorted using the FACS Melody (BD Biosciences), with single cells isolated and added to a 96-well plate with conditioned MEM media. Remaining positive cells were analysed against wild-type DU145 cells in a nuclease mismatch assay (Fig. S6a). Single cells were grown to confluency before transfer to a 6-well plate, where cell colonies were analysed and confirmed as vimentin knockout *via* western blot and immunofluorescence (Fig. S6b-d).

### hGIIA tagging

hGIIA was purified from conditioned media obtained from a 25-litre culture grown on alginate beads of hGIIA-expressing CHO cell line 2B1 [51] using AKTA Smart FPLC (GE Healthcare). Aliquots of hGIIA (100 µL, 1 mg/mL) in PBS were added to 15 µL of 1 M NaHCO_3_ and conjugated to 20 µg of Alexa Fluor 568 or 647 NHS ester. The solution was then put into 5000–8000 MWCO Spectra/Por dialysis tubing (Spectrum) and dialysed overnight in 3 × 1000 mL PBS on a magnetic stirrer to remove unconjugated dye. The degree of labelling (DOL) was < 1 for live cell imaging and was validated via isoelectric focusing (Fig. S8a) and immunofluorescence comparison with untagged hGIIA. hGIIA/AF647 functionality was validated relative to untagged hGIIA via i) isoelectric focusing and ii) comparative fluorescence imaging of hGIIA localisation with immunostained LNCaP and fibroblast-like synoviocyte RA57 cells, confirming no changes in charge or localisation, respectively (Fig. S8b).

### Immunofluorescence

Samples were fixed with 4% paraformaldehyde for 10 min and permeabilized with 0.4% Triton X-100 in PBS for 10 min. Samples were then blocked with 10% goat serum for 20 min before incubation with primary conjugated antibodies for vimentin/Alexa Fluor 488 (BD Biosciences, 1:100) and hGIIA/Alexa Fluor 568 (in-house, 1:50) for 1 hour at room temperature. Samples were imaged with the Zeiss LSM 800 confocal microscope using Airyscan detector for high-resolution images. Z-stacks were acquired with a 0.2 µm step-size. Weighted colocalisation coefficients were calculated using the Zen Blue colocalisation module (Zeiss), with thresholds set for each cell line using a single antibody control group. Analysis of fluorescence mean and % positive area was quantified using ImageJ.

### Live cell imaging

For all live cell imaging, cells were seeded on a 24-well glass-bottom Senso plates (Greiner Bio-one) and imaged at 37 °C and 5% CO_2_ with the LSM 800 (Zeiss). For c2 imaging, cells were incubated with c2 100 µM for 24 hours before excitation with 488 nm and emission 500-700 nm detected. For hGIIA entry, PC-3, LNCaP, DU145 and RA57 hGIIA/AF647 in cells were stained with 30 nM DAPI for 10 mins, cells were washed with PBS and then stained with 1 μg/mL of propidium iodide for 10 mins to confirm cell viability. Cells were washed three times with PBS and then cells were incubated with 200 ng/mL hGIIA/AF647, with entry imaged over 16 hours. Imaging parameters were kept consistent across all time points and cell lines.

For vimentin-hGIIA interactions, PC-3 cells were transfected with a plasmid encoding fusion protein EGFP-Vim7 (Addgene plasmid #56439, a gift from Michael Davidson) using Lipofectamine 3000 (Invitrogen) as per the manufacturer’s instructions. Three days after transfection, cells were incubated with 200 ng/mL hGIIA/AF568. For hGIIA dynamics, PC-3, LNCaP, DU145 or DU145 vimentin knockout cells were incubated with 200 ng/mL hGIIA/AF568 and with and without inhibitor c2 at 100 μM for 24 hours. Cells were then live imaged at a frame rate of 633 ms for 3 min and analysed using Imaris 8.2.0 (Bitplane) using the spot creation wizard. Briefly, the average hGIIA punctate diameter was measured at 0.8 μm, which was used along with fluorescent intensity to calibrate spot creation for each hGIIA punctate and vesicle speed, total displacement and vesicle size were measured.

For calcium imaging, PC-3 LNCaP and DU145 cell lines were seeded overnight. Media was removed and cells were washed with 500 μL of calcium recording buffer (125 mM NaCl, 2 mM MgCl_2_, 4.5 mM KCl, 10 mM Glucose, 2 mM CaCl_2_, 20 mM HEPES, pH 7.4) then incubated with calcium buffer including 5 µM Fura Red AM (Thermo Fisher Scientific) dye for 30 mins. Cells were excited using the 405 nm laser at 20%, and the 488 nm laser at 2.5%, and emission was captured from 495-700 nm. All settings remained constant for all measurements. Baseline intracellular calcium levels were measured for 60 sec before cells were incubated with hGIIA/AF647 (200 ng/mL) with calcium measured for another 300 sec. Ratiometric images were created using the Ratio Plus plugin for ImageJ.

### Fluorescence Lifetime Imaging

LNCaP cells were fixed, permeabilized, blocked and stained as described above with anti-vimentin/AF488 and anti-hGIIA/AF568. Samples were imaged with the Zeiss LSM 880, excited with 2- photon excitation tuned to 960 nm at 80 MhZ and the fluorescence lifetime of the donor only was captured and collected with the 520-535 nm filter. Fluorescence lifetime was processed using the PicoQuant system and captured until fluorescence lifetime had decayed to background levels (~1 min). For calibration, the lifetime of cells stained with only donor fluorescence (anti-vimentin/AF488) was captured (0% FRET point), as well as unstained cells (100% FRET point). All captured fluorescence lifetime analysed, and FRET was calculated analysed using SimFCS (Globals) for FLIM as described previously [53].

### PGE_2_ ELISA

PC-3, LNCaP and DU145 cells were seeded in a 6-well plate at 300,000 cells/well and incubated overnight. Complete media was removed and replaced with serum-free medium was supplemented with L-glutamine and 0.1% fatty-acid-free BSA (Sigma-Aldrich) for 1 hour. Cells were then incubated with combinations of TNF (10 ng/mL), hGIIA (2 μg/mL) H48Q (2 μg/mL) and c2 (50 µM) for 24 hours at 37 °C and 5% CO_2_. Each treatment was conducted in triplicate. Media was harvested then acidified to pH 2 with 150 μL of formic acid prior to solid phase extraction (SPE) using Oasis HLB 30 mg SPE cartridges (Waters, Eschborn, Germany). Cartridges were conditioned with 1 mL of methanol and equilibrated with 1 mL of water. Cell media was loaded onto columns and passed through using a 20-position cartridge manifold (Waters). Columns were washed with 1 mL aqueous 0.1% formic acid, then 1 mL 5% MeOH + 0.1% formic acid, then eluted with 1 mL 100% methanol. Loaded samples were then dried down for 4 hours in a Savant SPD131DDA Speedivac (Thermo Fisher Scientific). Samples were resuspended in 100 μL ELISA buffer and PGE_2_ concentration quantified using a commercial EIA kit (Cayman Chemical) according to the manufacturer’s instructions.

### Western Blot

PC-3, LNCaP and DU145 cells were treated for the time and with hGIIA, TNF and c2 as indicated in the figure legends. After treatment, cells were lysed in lysis buffer (150 mM NaCl, Triton X-100 0.5%, EDTA 2 mM, EGTA 2 mM, NaF 25 mM, b-glycerophosphate 25 mM, glycerol 10%, 50 mM Tris-HCl, pH 7.5) and incubated at 4 °C for 30 min. Lysates were then processed for Western blot analysis. Phospho-cPLA_2_-α (Ser505; 2831), cPLA_2_-α (2832) and EGFR (2232) were purchased from Cell Signaling. Vimentin (V9) antibody was purchased from Abcam. hGIIA (9C1) antiserum was prepared in-house (Scott unpublished). All antibodies were used at 1:1000, except for hGIIA antiserum at 1:500.

### Co-immunoprecipitation

After cell lysis, the lysate was incubated with monoclonal anti-hGIIA SCACC353 (Cayman Chemical) for two hours, then with 20 μL protein G agarose beads (Abcam) were added to the lysate and incubated overnight. Beads were centrifuged, washed three times with lysis buffer, then run on a gel and detected via western blot with either EGFR or vimentin antibodies as described above.

### Enzyme immunoassay

Prior to assay, hGIIA antibody 4A1 was conjugated to alkaline phosphatase (4A1-AP), produced using the lightning-link conjugation kit (ab102850, Abcam) as per the manufacturer’s instructions.

Purified vimentin fragments (full length or coil 2) were seeded to a 96-well microplate at 15 μg/mL with a 100 μL/well volume. After an overnight incubation at 4 °C, the plate was emptied. Each well was then blocked overnight at 4 °C with 200 μL of 1% BSA in PBS. The plate was emptied and washed with PBST (PBS + 0.05% Tween20). hGIIA solution of 100 ng/mL in PBS with 0.1% BSA was prepared with and without inhibitors c2, Withaferin A and LY311727 in DMSO with concentrations 0.1-100 µM. hGIIA working solution with 0.4% DMSO served as a positive control and PBS with 0.1% BSA served as a negative control. A 100 μL volume of each prepared hGIIA-inhibitor mixture was added to wells in triplicate and incubated at 37 °C for two hours. The plate was washed twice with PBST, then incubated with the 4A1-AP conjugate at 1 μg/mL 0.1% BSA in PBS and incubated for one hour at 37 °C. Plates were then emptied and washed three times with PBST, followed by washing twice with carbonate buffer (8 mM Na_2_CO_3_, 36 mM NaHCO_3_, 2 mM MgCl_2_, pH 9.8), before incubation with AP substrate *p*-nitro-phenyl phosphate (PNPP) powder at 1 mg/mL concentration in carbonate buffer. The plate was incubated at 37 °C for 60 mins then absorbance was read at 405 nm by a Spectramax M2 plate reader. All washes were a 200 µL/well volume.

### Molecular docking studies

Docking of the c2 ligand on the second domain of vimentin was conducted with Glide XP flexible docking as part of the Maestro software suite 10.2 (Schrödinger, LLC). The X-ray crystal structure 3TRT of vimentin was imported from the Protein Data Bank (PDB). The docking on vimentin was performed on five grids to allow for the inclusion of the entire surface area. The dimensions of the grid were 36 Å × 36 Å × 36 Å. The c2 ligand structure was processed by the LigPrep module as per default settings under the OPLS 2005 force field and was ionized by generating possible states at pH 7.4 using the Epik module of Maestro. In addition, specific docking of c2 to the vimentin coil 2 dimer was also performed using quCBit software (www.medchemsoft.com). c2 was similarly docked to the extracellular ligand binding domain of EGFR using crystal structure PDB 1nq1.

### Flow cytometry

AnnexinV-fluorescein isothiocyante (AnnexinV-FITC) and Propidium Iodide (PI) staining (Abcam, Cambridge, USA), was used to analyse cell death at 72-hours post-treatment of c2. DU145, DU145 vimentin KO, PC3 and LNCAP cells were seeded in a 6-well plate (10^5^ cells/mL) and treated with 100 µM concentration of c2 and incubated at 37 °C and 5% CO2. After 72 hours, cells were trypsinized with 300 μL trypsin/well, and 200,000 cells at a final concentration of 500 cells / μL were transferred into Eppendorf tubes, which was centrifuged at 1500 rpm (Heraeus Multifuge X3R Refrigerated Benchtop Centrifuge, TX-1000 rotor, ThermoScientific) for 5 minutes at 4 °C. The supernatant was removed, and the pellet was suspended in 100 µL of 1X Annexin V Binding Buffer. Each sample was moved to polystyrene round-bottom tubes (Interpath Services, Australia). To each tube, 2 μL of Annexin V-FITC and 2 uL of PI were added and incubated in the dark for 10 min. Cells were analysed using the BD FACSCanto II Benchtop Flow Cytometer (Biosciences, CA). The AnnexinV/PI data was measured on FL1-H versus FL2-H scatter plot using FlowJo^TM^ v10.9 software (Fig. S7).

### Statistical analysis

All graphs and statistical analysis, including student’s *t*-tests, one-way and two-way analysis of variance (ANOVA), and regression of linear and non-linear functions, were performed with GraphPad Prism 8.

## Supplementary information

Clean Revised Supplementary text summary

Figure S1

Figure S2

Figure S3

Figure S4

Figure S5

Figure S6

Figure S7

Figure S8

Uncropped Western Blots

Movie S1: Vimentin acts as a cage to hGIIA.

Movie S2: Vimentin is involved in the trafficking of hGIIA..

Movie S3: Vimentin is involved in the trafficking of hGIIA.

Movie S4: hGIIA dynamics with DMSO treatment in PC-3 cell.

Movie S5: hGIIA dynamics with c2 (100 μM) treatment in PC-3 cell.

Datasets for Manuscript Figures

Appendix

## Data Availability

Data are available from the corresponding author on request.
