# Supplementary material for: Targeting secreted PLA2 interactions with EGFR and vimentin to arrest prostate tumour growth
Source: Cell Death Dis. 2025 Dec 20;17(1):183. doi: 10.1038/s41419-025-08280-x (PMC12876990; doi:10.1038/s41419-025-08280-x)
Supplement: Supplementary file 1 — Clean Revised Supplementary text summary [file 41419_2025_8280_MOESM1_ESM.docx]

Supplementary Text Summary

**Figure S1: Abundance of hGIIA gene (*PLA2G2A*) mRNA expression in prostate cancer tissues (PRAD) relative to healthy prostate tissue.** (a). Transcripts per million (TPM) of *PLA2G2A* across all tissue and tumour types. Figure derived from analysis of publicly available TCGA Data, accessed through the GEPIA portal^17^. ACC–Adrenocortical carcinoma; BLCA–Bladder Urothelial Carcinoma; BRCA–breast invasive carcinoma; CESC–Cervical squamous cell carcinoma & endocervical adenocarcinoma; CHOL– Cholangiocarcinoma; COAD-Colon adenocarcinoma; DLBC–Lymphoid Neoplasm Diffuse Large B-cell Lymphoma; ESCA–Esophageal carcinoma; GBM–Glioblastoma multiforme; HNSC–Head & Neck SCC; KICH–Kidney Chromophobe; KIRC–Kidney renal clear cell carcinoma; KIRP–Kidney renal papillary cell carcinoma; LAML–Acute Myeloid Leukemia; LGG– Brain Lower Grade Glioma; LIHC–Liver hepatocellular carcinoma; LUAD–Lung adenocarcinoma; LUSC–Lung squamous cell carcinoma; MESO–Mesothelioma; OV– Ovarian serous cystadenocarcinoma; PAAD–Pancreatic adenocarcinoma; PCPG– Pheochromocytoma & Paraganglioma; PRAD– Prostate adenocarcinoma; READ–Rectum adenocarcinoma; SARC–Sarcoma; SKCM–Skin Cutaneous Melanoma; STAD–Stomach adenocarcinoma; TGCT–Testicular Germ Cell Tumours; THCA–Thyroid carcinoma; THYM–Thymoma; UCEC–Uterine Corpus Endometrial Carcinoma; UCS–Uterine Carcinosarcoma; UVM–Uveal Melanoma. (b) *PLA2G2A* expression in prostate adenocarcinoma tissue [Left, N (tumour tissue) = 492 and N (healthy tissue) = 152 showing significantly higher expression of *PLA2G2A* in PRAD compared with healthy tissues (one-way ANOVA, a = 0.01, * p < 0.01). Data (TPM transcripts per million) are transformed [log2 (TPM+1)], and represented as box plots, showing median values and quartiles.

**Figure S2: Pharmacokinetics of cF and c2 after oral administration**. Concentration of cF (a) or c2 (b) in liver, kidney, muscle, heart and lung from 1-24 hours after oral administration of cF or c2 (5 mg/kg; both n = 4), respectively. Data are mean ± SE (c) Identity and validation of cF and c2 radioactively-labelled with tritium. Mice (male, seven weeks old, BALB/c, n = 4 per timepoint) were administered with 5 mg/kg of tritiated cF or c2, by the routes and times indicated. Peptide content of tissues were determined by liquid scintillation counting. Stability of peptides following administration to animals was evaluated by thin layer chromatography of tissue-extracted peptide (red and blue lines) relative to peptide prior to injection (purple and green lines).

**Figure S3: Characterisation of c2 autofluorescence.** a) Fluorescent lifetime images (left) and phasor plot of fluorescence lifetime (right) of LNCaP cells immunostained with primary conjugated anti-hGIIA antibody 4A1/AF568 and vimentin antibody RV202/AF488 identified a distinct lifetime at 1.3 ns in cells incubated with c2 (100 μM) shown in yellow cursor. Images taken with 63 x objective, n = 3 biological replicates. (b) Emission spectrum of c2 in response to 405 nm excitation showing a broad emission maximum from 450 nm -500 nm. (c) Representative z-stack of PC-3, LNCaP and DU145 cell lines incubated with c2 at 100 μM (green) for 24 hours, with DAPI (blue). Scale bar 20 μm, step size 1 μm, showing planes xy, xz and yz.

**Figure S4: Quantification of IHC staining following c2 treatment.** Tumours (a) androgen dependent LNCaP-luc and (b) androgen independent PC-3M treated with either vehicle control (DMF) or c2 (0.1 mg/kg, thrice weekly) were stained with TUNEL or Ki67, CD31, vimentin or hGIIA antibodies. Data are mean ± SE. Significant differences were determined by two-tailed Student’s unpaired t-test. Representative images are shown. Scale bar 250 µm, inset 100 µm^2^.

**Figure S5: hGIIA and c2 entry and localisation**. (a) Immunofluorescent staining of PC-3, and LNCaP cell lines with hGIIA (red), caveolin-1 (green) and DAPI (blue) shown in overlay. Colocalisation (pink) between hGIIA and cav-1 staining (white arrows) as calculated by Costes *et al.* (2004) method^54^. Cells with 63 x objective, n = 3 biological replicates, 5 cells per replicate, scale bar is 10 μm. (b) PC-3 cells incubated without (control) or with 200 μg/mL of protamine sulfate for 60 min prior to washing with PBS and incubation with 200 ng/mL of hGIIA/647 for 24 hours. Scale bar 50 μm, n = 3, >15 cells per replicate, error bars SEM. ***, p<0.0001; statistical significance determined by Student’s unpaired two-tailed t test.

**Figure S6: Vimentin knockout in DU145 cells**. (a) Nuclease mismatch assay of wtDU145 and DU145 vimentin knockout. Single bands in wtDU145 indicate homoduplexes (no editing), while multiple bands (indicated by arrows) in the DU145 vimentin knockout lane confirm the formation of heteroduplexes and correct editing at the desired site. (b) Immunofluorescence staining of vimentin (green) and DAPI in wtDU145 and (c) vimentin knockout. (d) Western blot of SDS-size-fractionated cell lysates from wtDU145 (WT) and vimentin knockout mutant clonal colonies detected for vimentin and GAPDH. Arrow indicates clone chosen for further studies.

**Figure S7: c2 initiates apoptosis via a vimentin-mediated mechanism**. PC-3, LNCaP, DU145 WT and DU145 vimKO were treated with either control (nothing), DMSO (0.5%), c2 100 μM for 72 hours or positive control of all cell lines incubated at 42 ºC for 5 min. Cells were then stained with Annexin V-FITC and propidium iodide (PI), before FACS analysis). The Annexin V/PI data was measured on FL1-H versus FL2-H scatter plot using FlowJoTM v10.9 software (n=3 biological replicates).

**Figure S8: Validation of hGIIA tagged with Alexa Fluor 647 (hGIIA/AF647)**. (a) Iso-electric focusing (IEF) of hGIIA and hGIIA/AF647 yields pI (both at ~10.5). (b) Representative images of immunofluorescence of exogenous untagged hGIIA, detected with primary conjugated anti-hGIIA antibody 4A1/AF568 (red) entry compared to live cell imaging of hGIIA/AF647 (green) entry over 24 hours in RA57 and LNCaP cell lines. hGIIA or hGIIA/AF647 protein (200 ng/mL) was added and fluorescence intensity was measured at 0, 2 min on ice, or 1 h, 16 h and 24 h at 37 degrees. Scale bar 50 μm. Graphs show quantitation of fluorescence, n =1, 20 cells per replicate, error bars SE.

**Movie S1: Vimentin acts as a cage to hGIIA**. PC-3 cells transfected with Vim-EGFP (green) were treated with hGIIA/AF568 (200 ng/mL) for 24 hours. Vimentin-positive (red) and vimentin-negative (yellow) populations of hGIIA spots were sorted based on green, fluorescent intensity. Vimentin-positive spots had reduced speed and size. Representative cell of n = 3, 5 cells per replicate.

**Movie S2: Vimentin is involved in the trafficking of hGIIA**. LNCaP cells transfected with Vim-EGFP (green) were treated with hGIIA/AF568 (200 ng/mL) (red) for 24 hours. hGIIA/AF568 vesicles move freely around the cell and can also dock directly to vimentin filaments, where they fuse and divide. hGIIA/AF568 colocalised with vimentin can also remain stationary or be actively moving while remaining colocalised to filaments. Scale bar is 10 μm.

**Movie S3: Vimentin is involved in the trafficking of hGIIA**. A closeup of Movie S2. **Movie S4: hGIIA dynamics with DMSO treatment in PC-3 cell**. Live PC-3 cells were imaged with 63 x objective and treated with hGIIA/AF568 (200 ng/mL) and DMSO (0.5%) for 24 hours. Quantification of hGIIA/AF568 fluorescent dynamics allowed for particle tracking and spot size, with tracks colour coded with speed (μm/sec). Representative cell of n = 3.

**Movie S5: hGIIA dynamics with c2 (100 μM) treatment in PC-3 cell**. Live PC-3 cells were imaged with 63 x objective and treated with hGIIA/AF568 (200 ng/mL) and c2 (100 μM) for 24 hours. Quantification of hGIIA/AF568 fluorescent dynamics allowed for particle tracking and spot size, with tracks colour coded with speed (μm/sec). Representative cell of n = 3.
