# Supplementary figures and images for: Targeting secreted PLA2 interactions with EGFR and vimentin to arrest prostate tumour growth
Source: Cell Death Dis. 2025 Dec 20;17(1):183. doi: 10.1038/s41419-025-08280-x (PMC12876990; doi:10.1038/s41419-025-08280-x)

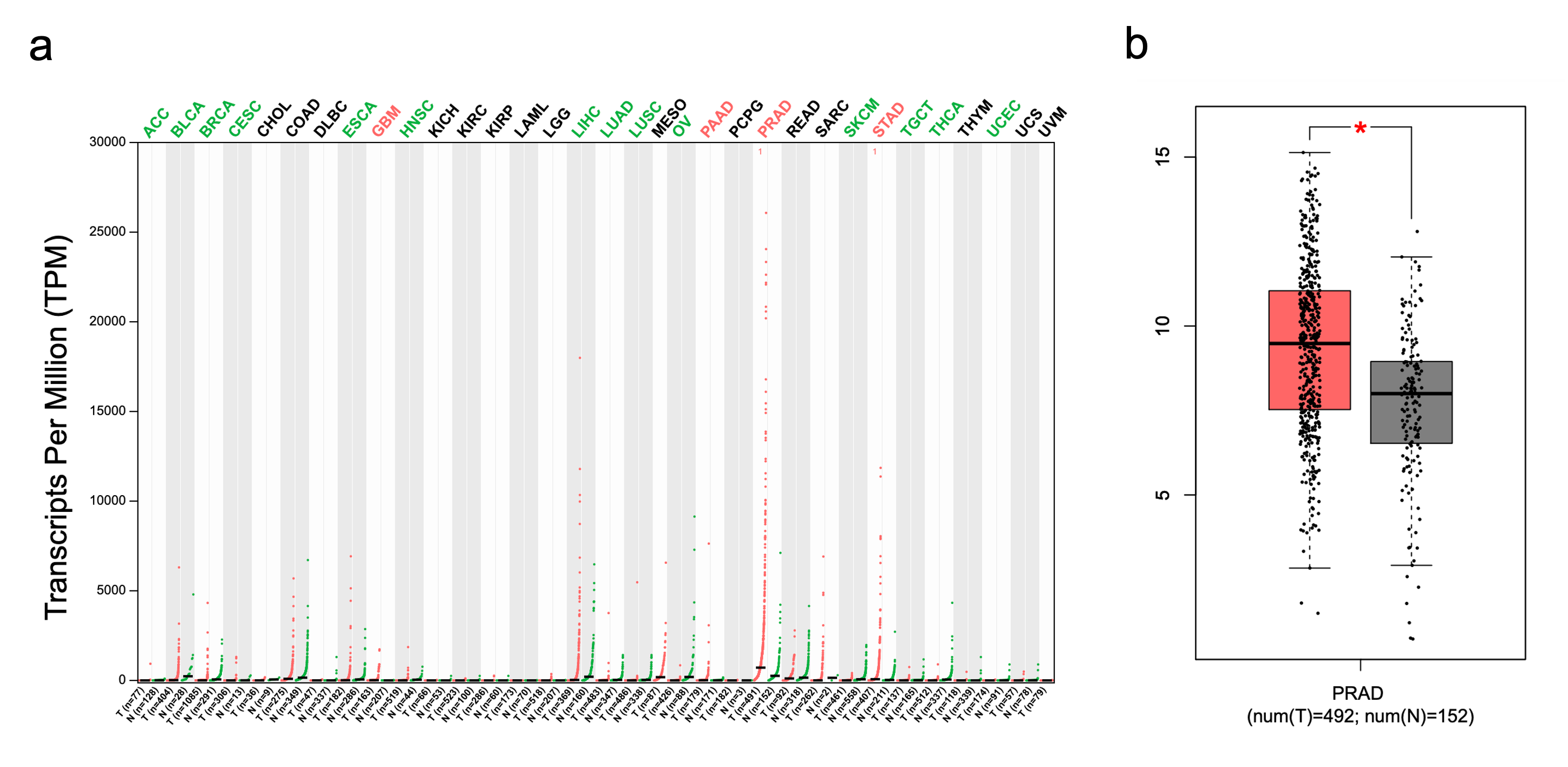

Supplement: Supplementary file 2 — Figure S1 [file 41419_2025_8280_MOESM2_ESM.png]

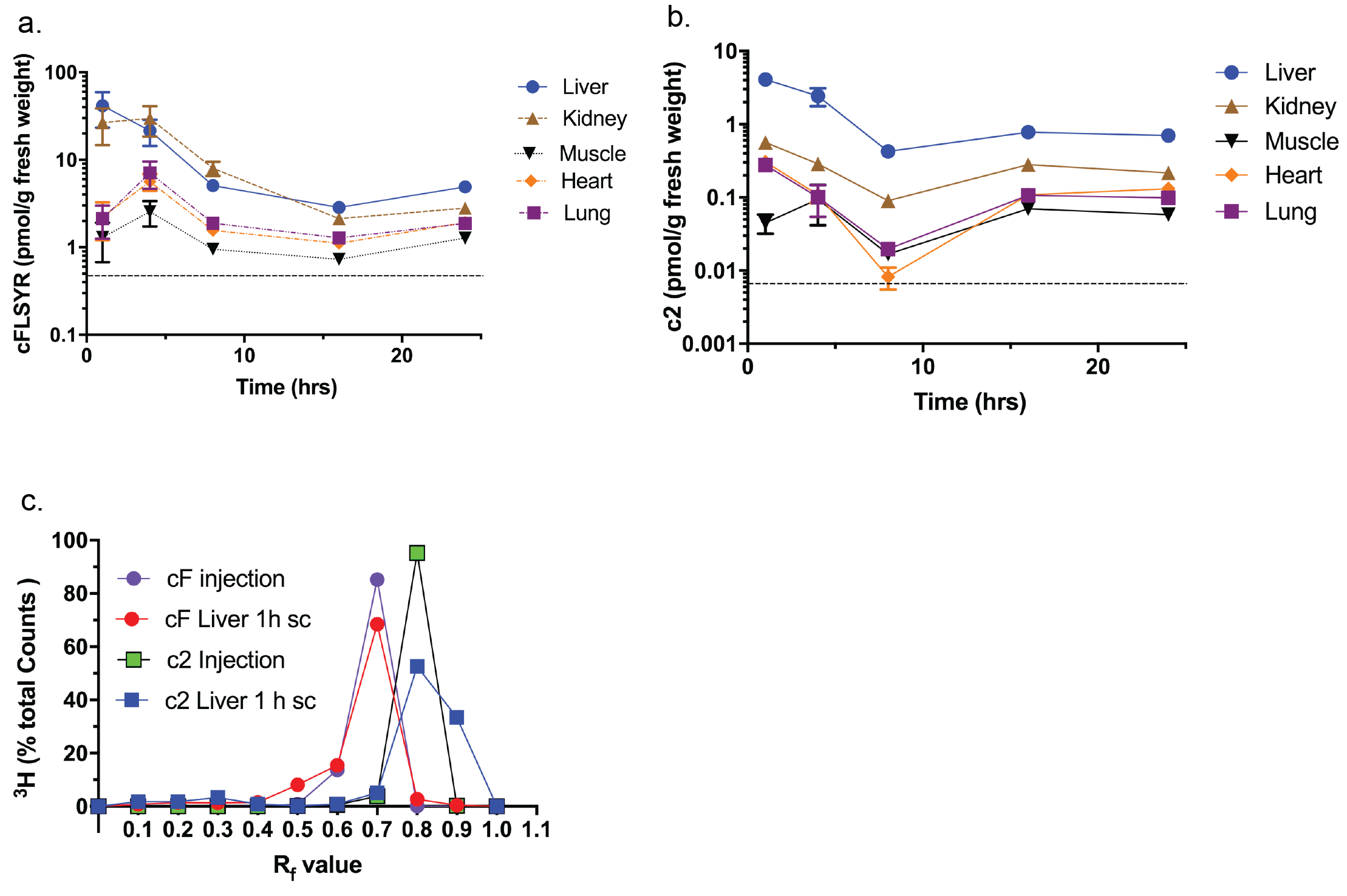

Supplement: Supplementary file 3 — Figure S2 [file 41419_2025_8280_MOESM3_ESM.png]

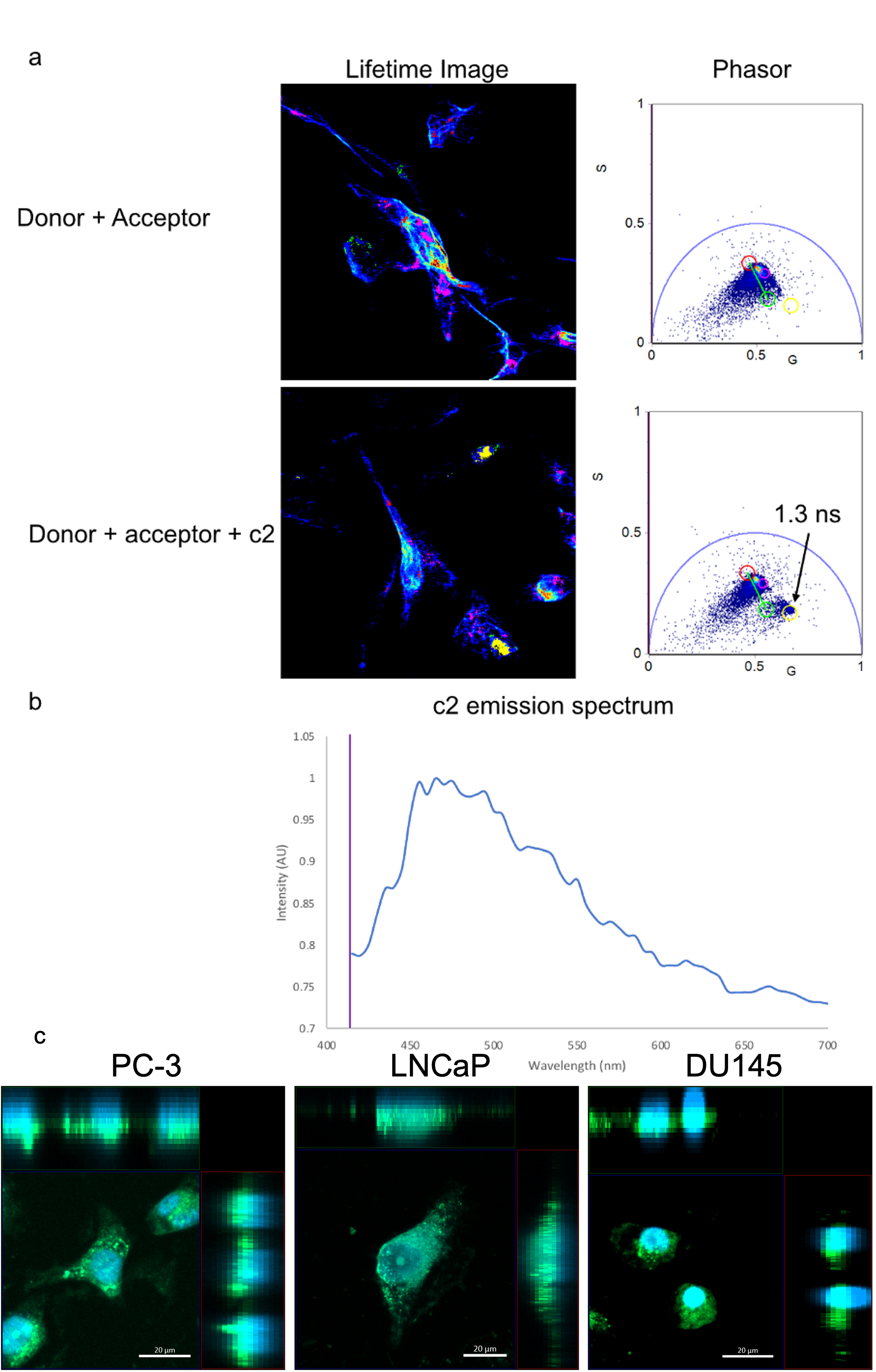

Supplement: Supplementary file 4 — Figure S3 [file 41419_2025_8280_MOESM4_ESM.png]

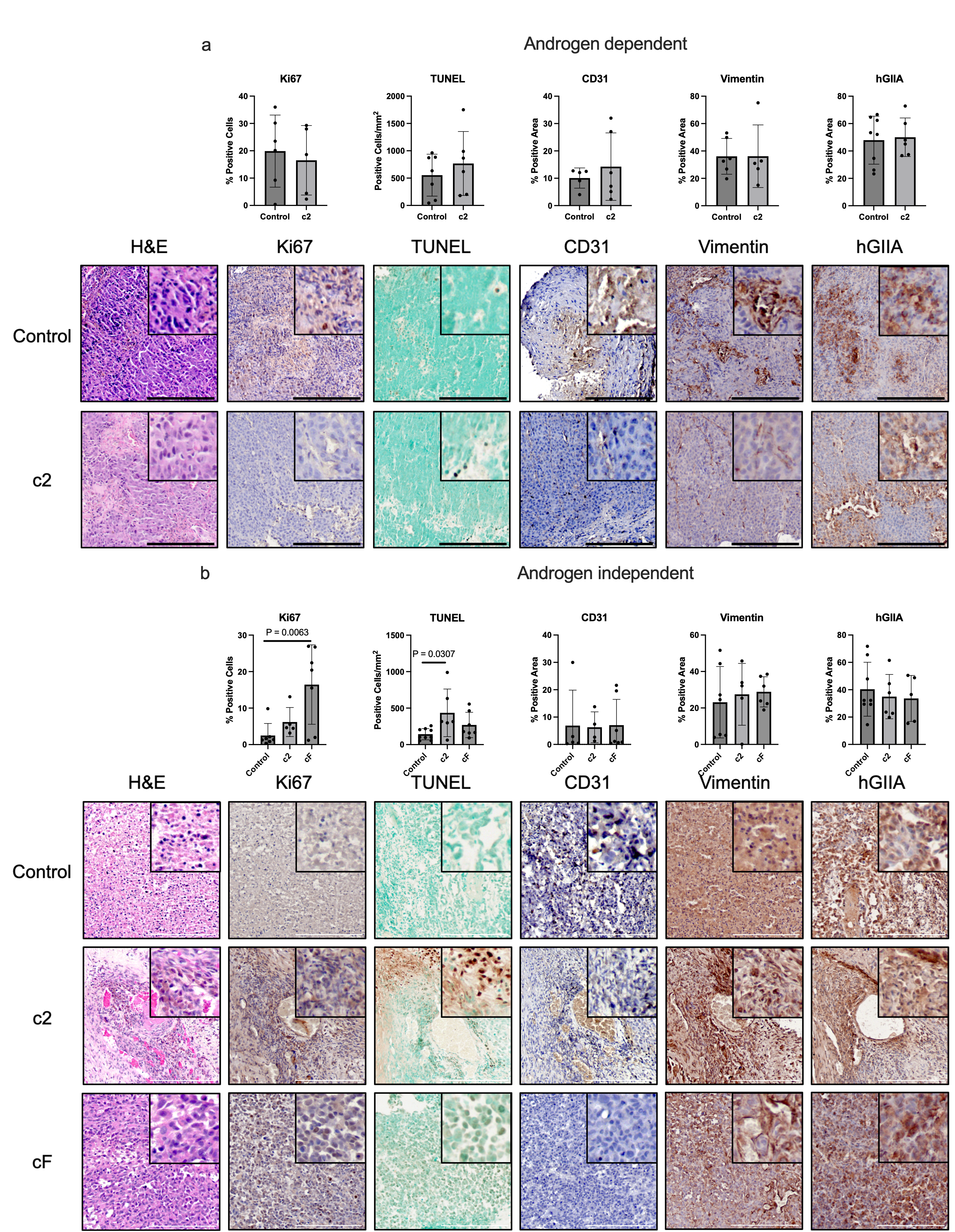

Supplement: Supplementary file 5 — Figure S4 [file 41419_2025_8280_MOESM5_ESM.png]

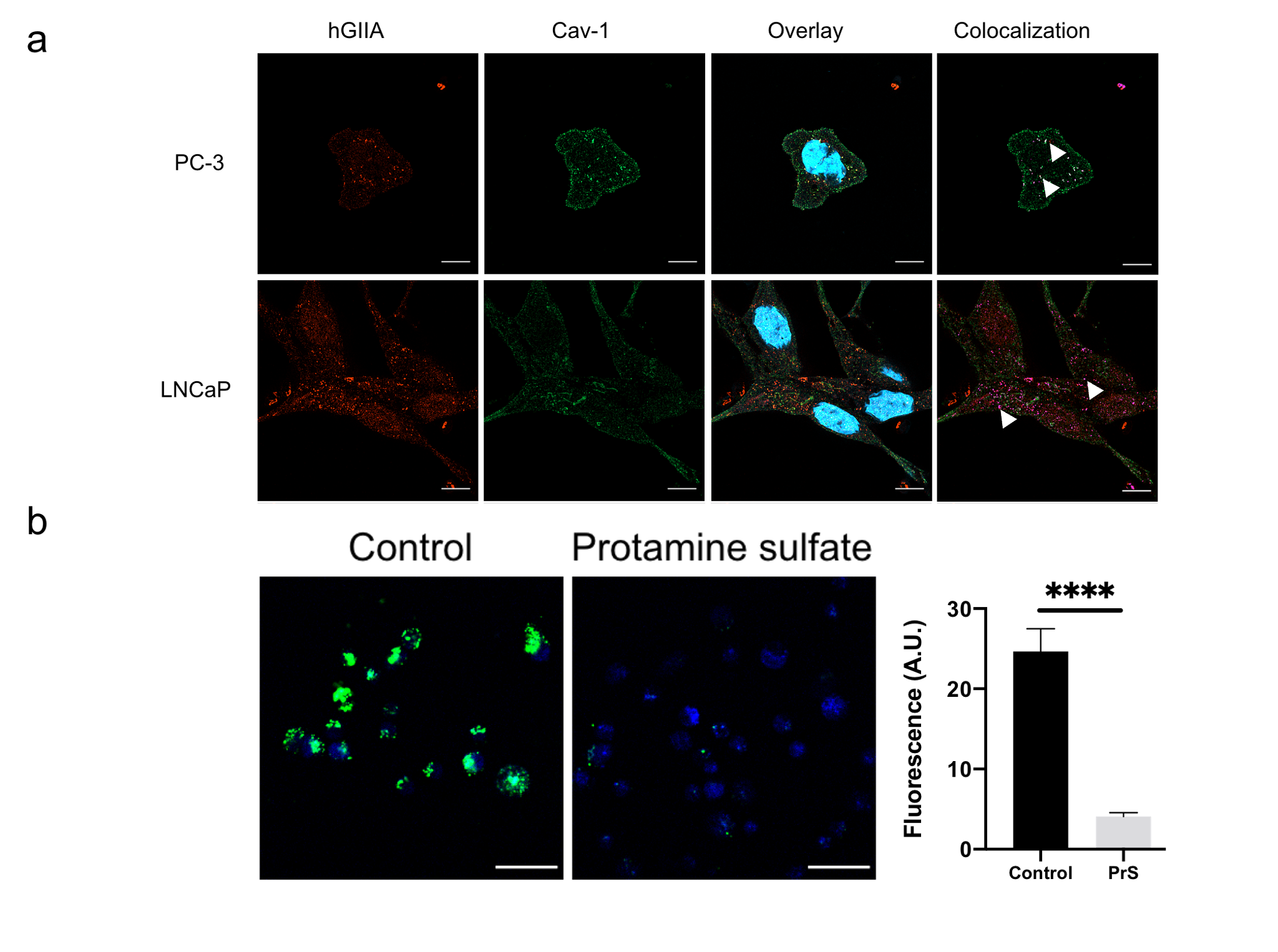

Supplement: Supplementary file 6 — Figure S5 [file 41419_2025_8280_MOESM6_ESM.png]

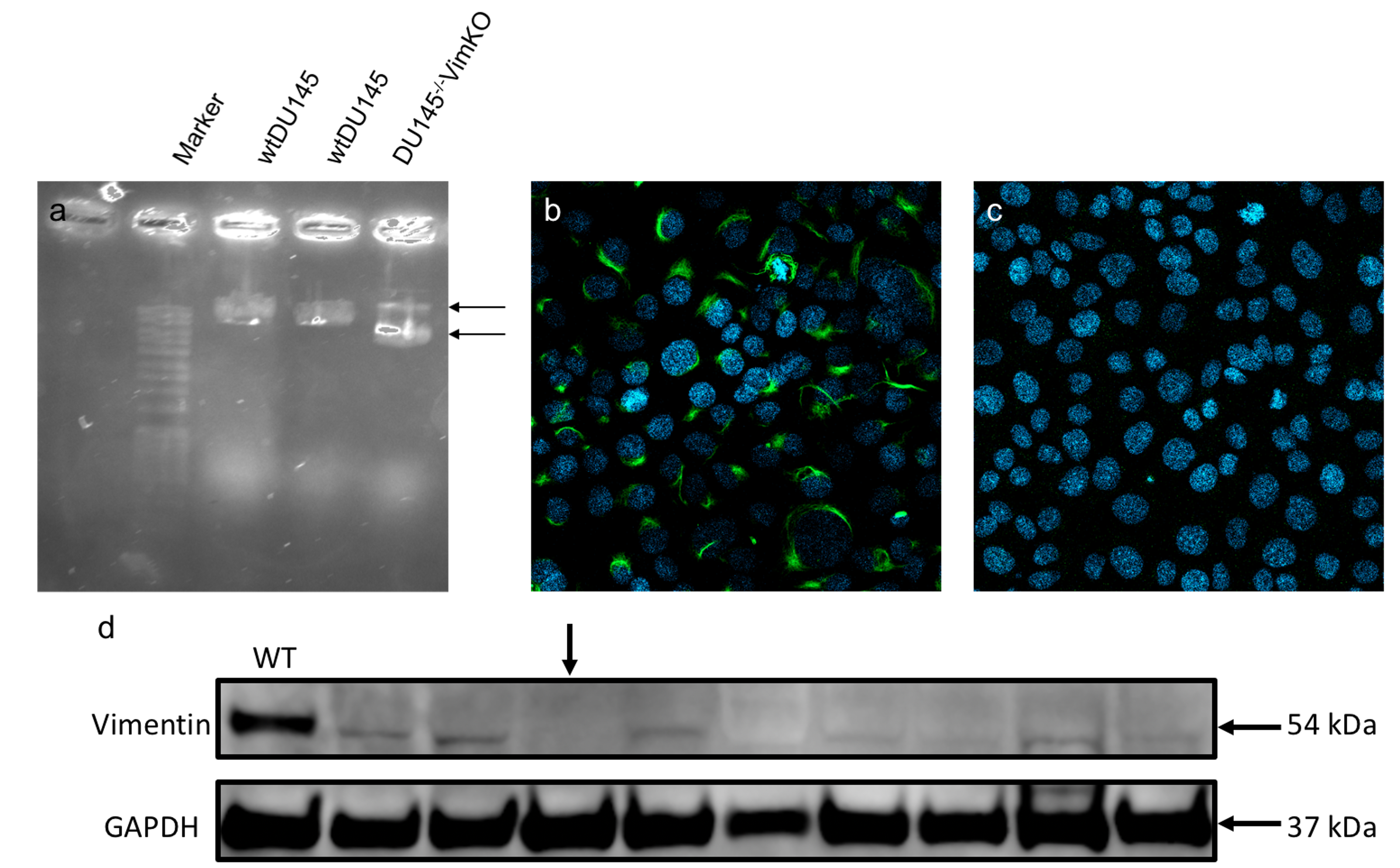

Supplement: Supplementary file 7 — Figure S6 [file 41419_2025_8280_MOESM7_ESM.png]

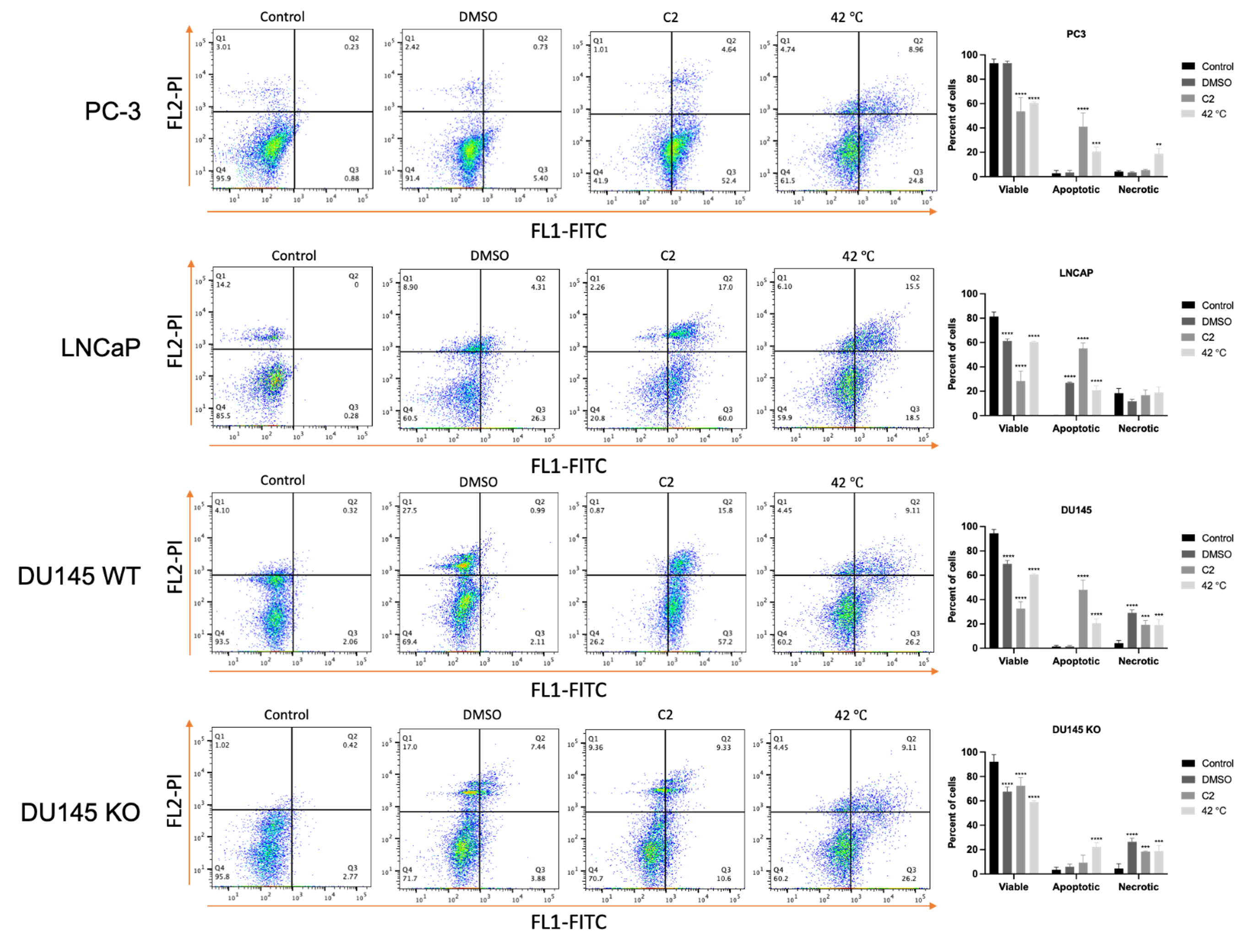

Supplement: Supplementary file 8 — Figure S7 [file 41419_2025_8280_MOESM8_ESM.png]

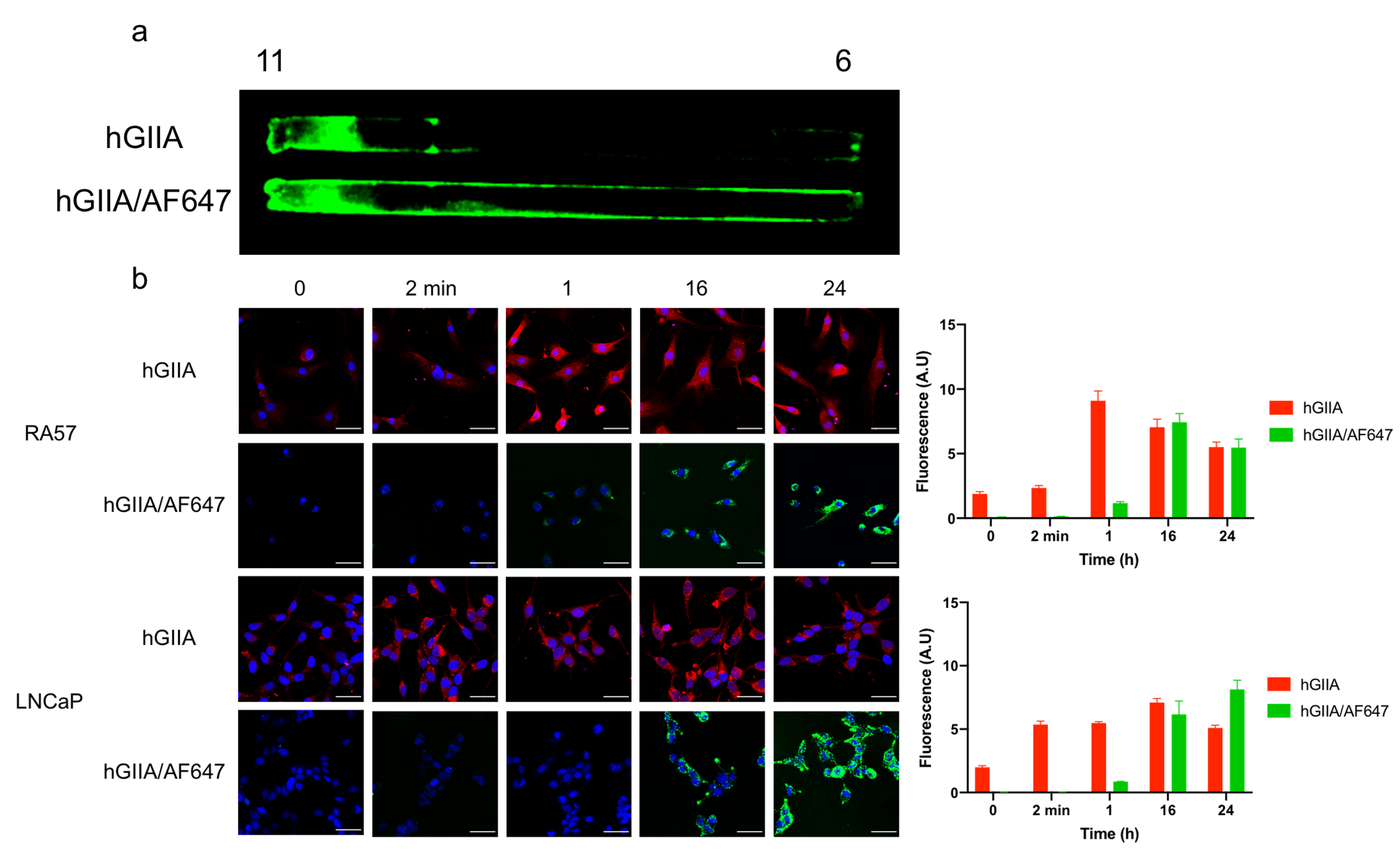

Supplement: Supplementary file 9 — Figure S8 [file 41419_2025_8280_MOESM9_ESM.png]

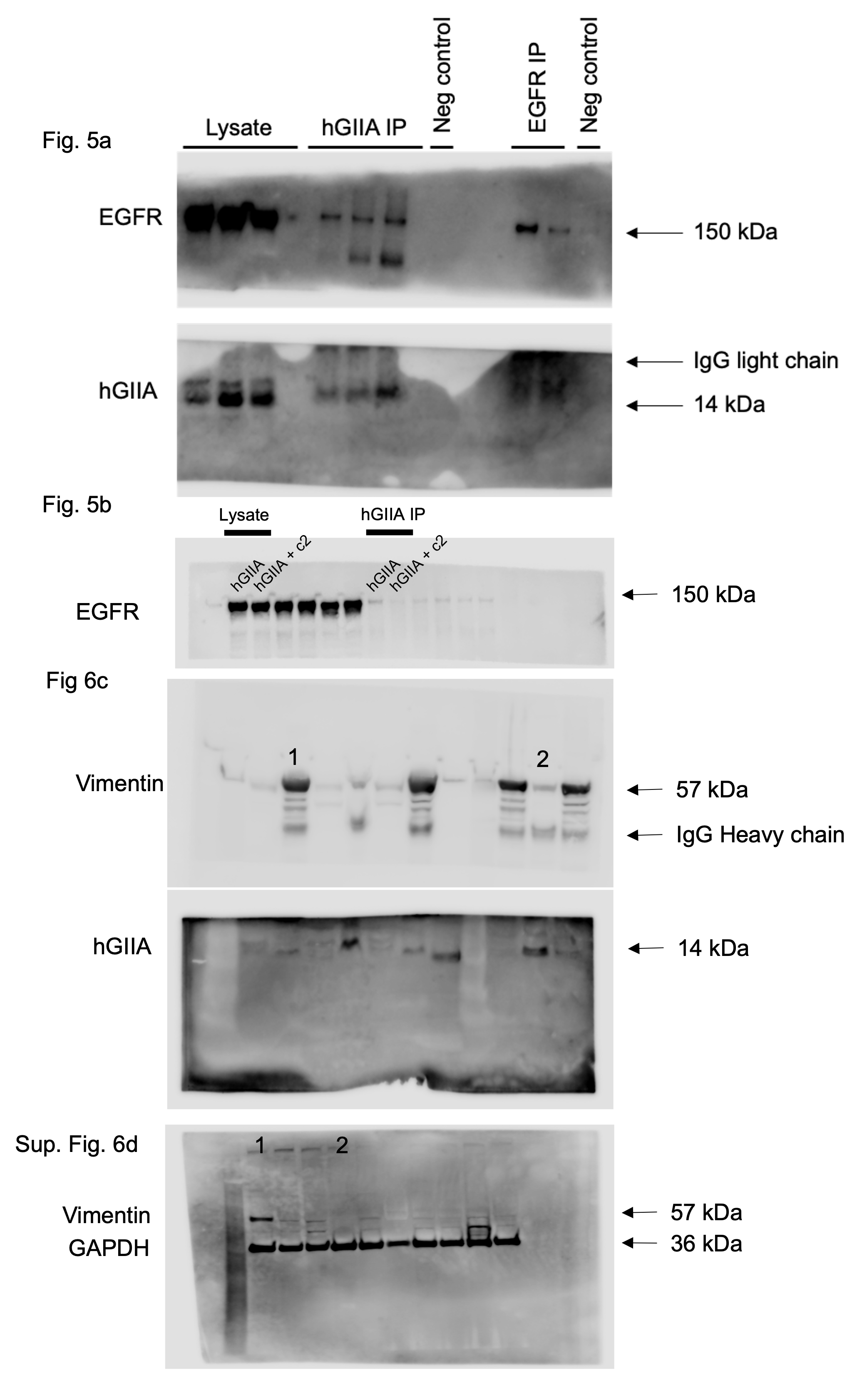

Supplement: Supplementary file 10 — Uncropped Western Blots [file 41419_2025_8280_MOESM10_ESM.png]
