# Supplementary material for: Targeting secreted PLA2 interactions with EGFR and vimentin to arrest prostate tumour growth
Source: Cell Death Dis. 2025 Dec 20;17(1):183. doi: 10.1038/s41419-025-08280-x (PMC12876990; doi:10.1038/s41419-025-08280-x)
Supplement: Supplementary file 17 — Appendix [file 41419_2025_8280_MOESM17_ESM.pdf]

Appendix 1.

The bright light of
certainty

I C P Firefly Pty Ltd

ACN 071 626 358

PO Box 6198, Alexandria NSW 2015 Australia
TEL: 61 2 9310 3899 FAX: 61 2 9310 4889 EMAIL: info@icpfirefly.com.au WEBSITE: www.icpfirefly.com.au

28-DAY REPEATED DOSE ORAL TOXICITY STUDY (OECD 407) OF c2 IN SPRAGUE DAWLEY RATS WITH A RECOVERY PERIOD

ICPQN1035.B

FINAL REPORT

Date: 14 March 2012

Submitted to: Prof. Paul De Souza
St George Clinical School
Clinical Sciences (WR Pitney) Building
Short Street
St George Hospital
Kogarah NSW 2217
AUSTRALIA

Page number: 1 of 50

TABLE OF CONTENTS

	Page No
**GOOD LABORATORY PRACTICE COMPLIANCE**	**7**
**STUDY DIRECTOR'S STATEMENT**	**8**
**QUALITY ASSURANCE STATEMENT**	**9**
**REGULATORY STATEMENT**	**10**
**ARCHIVES STATEMENT**	**11**
**PERSONNEL INVOLVED IN THE STUDY**	**12**
**1.0. SUMMARY**	**13**
**2.0. INTRODUCTION**	**14**
**2.1. Sponsor**	14
**2.2. Study Number**	14
**2.3. Study Director**	14
**2.4. Rationale for the study**	14
**2.5. Study timetable**	14
**2.6. Study integrity**	14
**2.7. Study Facility**	15
2.7.1. Test site 1	*15*
2.7.1.1. Phase of study	*15*
2.7.2. Test site 2	*15*
2.7.2.1. Phase of study	*15*
2.7.2.2. Principal Investigator	*15*
2.7.2.3. Test Site QA	*15*
**2.8. Definitions**	15
2.8.1. Dose	*15*
2.8.2. Dosage	*15*
2.8.3. Evident toxicity	*15*
2.8.4. Repeated dose toxicity	*16*
2.8.5. Test item	*16*
2.8.6. Vehicle	*16*
**2.9. Principle of the test method**	16
**3.0. MATERIALS AND METHODS**	**17**
**3.1. Regulatory references**	17
3.1.1. Test guidelines	*17*
3.1.2. Good Laboratory Practice	*17*
3.1.3. Animal Welfare Act compliance	*17*
**3.2. Test items**	17
**3.3. Vehicle**	17
**3.4. Animals**	17
3.4.1. Justification for species selection	*17*
3.4.2. Species, strain source and housing	*17*
3.4.3. Treatment allocation	*18*
3.4.4. Feed and water	*18*

TABLE OF CONTENTS (cont)

Page No

3.4.5. Environment *18*

3.5. Preparations 18

3.5.1. Test item *18*

3.5.2. Vehicle *18*

3.6. Procedure for test item administration 19

3.7. Observation of animals 19

3.7.1. Body weights *19*

3.7.2. Feed consumption *19*

3.7.3. Clinical observations *19*

3.7.4. Detailed clinical examinations *19*

3.7.5. Functional observation *19*

3.8. Blood sampling 20

3.9. Blood analyses 20

3.9.1. Haematology analysis *20*

3.9.2. Biochemistry analysis *20*

3.10. Urinalysis 20

3.11. Gross necropsy 20

3.12. Tissue processing / Slide preparation 21

3.13. Histopathology 21

3.14. Statistical analyses 21

4.0. RESULTS AND DISCUSSION **22**

4.1. Mortality 22

4.2. Body weights 22

4.2.1. Main and Recovery Groups: Week 1-4 *22*

4.2.1.1. Treated (Gr 2 - 1 mg/kg) vs. Vehicle control (Grs 1 & 4) *22*

4.2.1.2. Treated (Grs 3 & 5 - 20 mg/kg) vs. Vehicle control (Grs 1 & 4) *22*

4.2.2. Recovery period: Week 5-6 *22*

4.2.2.1. Treated (Gr 5 - 20 mg/kg) vs. Vehicle control (Gr 4)22

4.2.3. Discussion *22*

4.3. Feed intake 22

4.3.1. Main and Recovery Groups: Week 1–4 *23*

4.3.1.1. Treated (Gr 2 - 1 mg/kg) vs. Vehicle control (Grs 1 & 4) *23*

4.3.1.1. Treated (Grs 3 & 5 - 20 mg/kg) vs. Vehicle control (Grs 1 & 4) *23*

4.3.1.1. Recovery period: Week 5-6 *23*

4.3.2.1. Treated (Gr 5 - 20 mg/kg) vs Vehicle control (Gr 4)23

4.3.3. Discussion *23*

4.4. Clinical observations 23

4.4.1. Main and Recovery Groups: Week 1-4 *23*

4.4.1.1. Vehicle control (Gr 1 & 4) *23*

4.4.1.2. Treated (Gr 2 - 1 mg/kg) *23*

4.4.1.3. Treated (Gr 3 & 5 - 20 mg/kg) *23*

4.4.2. Recovery Groups: Week 5-6 *23*

4.4.2.1. Vehicle control (Gr 4) *23*

TABLE OF CONTENTS (cont)

Page No

4.4.2.2. Treated (Gr 5 - 20 mg/kg) 23
4.4.3. Discussion 23

4.5. Detailed clinical observations 23
4.5.1. Vehicle control (Grs 1 & 4) 24
4.5.2. Treated (Gr 2 - 1 mg/kg) 24
4.5.3. Treated (Grs 3 & 5 - 20 mg/kg) 24
4.5.4. Discussion 24

4.6. Functional observations 24
4.6.1 Vehicle control (Grs 1 & 4) 24
4.6.2 Treated (Gr 2 - 1 mg/kg) 24
4.6.3 Treated (Grs 3 & 5 - 20 mg/kg) 24
4.6.4 Discussion 24

4.7. Blood haematology analysis 24
4.7.1. Main Groups – Day 29 24
4.7.1.1. Vehicle control (Gr 1) 24
4.7.1.2. Treated (Gr 2 - 1 mg/kg) vs Vehicle control (Gr 1) 24
*4.7.1.3. Treated (Gr 3 - 20 mg/kg) vs Vehicle control (Gr 1)*24
4.7.2. Recovery Groups – Day 43 24
*4.7.2.1. Treated (Gr 5 - 20 mg/kg) vs Vehicle control (Gr 4)*24
4.7.3. Discussion 25

4.8. Blood biochemistry analysis 25
4.8.1. Main Groups – Day 29 25
4.8.1.1. Treated (Gr 2 - 1 mg/kg) vs Vehicle control (Gr 1) 25
*4.8.1.2. Treated (Gr 3 - 20 mg/kg) vs Vehicle control (Gr 1)*25
4.8.2. Recovery groups – Day 43 25
*4.8.2.1. Treated (Gr 5 - 20 mg/kg) vs Vehicle control (Gr 4)*25
4.8.3. Discussion 25

4.9. Urinalysis 25
4.9.1. Main Groups – Day 29 25
4.9.1.1. Treated (Gr 2 - 1 mg/kg) vs Vehicle control (Gr 1) 25
*4.9.1.2. Treated (Gr 3 - 20 mg/kg) vs Vehicle control (Gr 1)*25
4.9.2. Recovery Groups – Day 43 25
*4.9.2.1. Treated (Gr 5 - 20 mg/kg) vs Vehicle control (Gr 4)*25
4.9.3. Discussion 25

4.10. Gross necropsy 25
4.10.1. Main Groups: sacrificed - Day 29 26
4.10.1.1. Vehicle control (Gr 1) 26
4.10.1.2. Treated (Gr 2 - 1 mg/kg) vs Vehicle control (Gr 1) 26
*4.10.1.3. Treated (Gr 3 - 20 mg/kg) vs Vehicle control (Gr 1)*26
4.10.2. Recovery Groups – sacrificed - Day 43: 26
4.10.2.1. Vehicle control (Gr 4) 26
*4.10.2.2. Treated (Gr 5 - 20 mg/kg) vs Vehicle control (Gr 4))*26
4.10.3. Discussion 26

4.11. Histopathology 26
4.11.1. Alimentary and associated tissues Gr 1 and Gr 3 26
4.11.1.1. Stomach 26
4.11.1.2. Small intestine: ileum 26
4.11.1.3. Large intestine: colon 26
4.11.1.4. Liver 26

TABLE OF CONTENTS (cont)

		**Page No**
4.11.2.	*Cardiovascular and respiratory Gr 1 and Gr 3*	*27*
	4.11.2.1. Heart	*27*
	4.11.2.2. Trachea	*27*
	4.11.2.3. Lung	*27*
4.11.3.	*Endocrine Gr 1 and Gr 3*	*27*
	4.11.3.1. Adrenal glands	*27*
	4.11.3.2. Thyroid gland	*27*
4.11.4.	*Genito-urinary Gr 1 and Gr 3*	*27*
	4.11.4.1. Kidney	*27*
	4.11.4.2. Epididymis	*27*
	4.11.4.3. Prostate	*27*
	4.11.4.4. Seminal vesicle with coagulating glands	*27*
	4.11.4.5. Urinary bladder	*27*
	4.11.4.6. Testes	*27*
4.11.5.	*Lymphoid and haematopoietic tissues Gr 1 and Gr 3*	*28*
	4.11.5.1. Bone marrow	*28*
	4.11.5.2. Spleen	*28*
	4.11.5.3. Peyer's Patch	*28*
	4.11.5.4. Thymus	*28*
	4.11.5.5. Lymph node: mesenteric	*28*
4.11.6.	*Nervous and special tissues Gr 1 and Gr 3*	*28*
	4.11.6.1. Brain stem	*28*
	4.11.6.2. Cerebrum	*28*
	4.11.6.3. Cerebellum	*28*
	4.11.6.4. Spinal cord	*28*
**5.0.**	**CONCLUSION**	**29**

LIST OF TABLES

**Table 1 -** Deviations	**14**
**Table 2 -** Test item	**17**
**Table 3 -** Vehicle	**17**
**Table 4 -** Animal allocation	**18**
**Table 5 -** Mean Body Weights: Main and Recovery Groups Combined – Week 1-4	**30**
**Table 6 -** Mean Body Weights: Recovery Groups – Week 5- 6	**30**
**Table 7 -** Percentage Body Weight Gain: Main and Recovery Groups Combined – Week 1-4	**31**
**Table 8 -** Percentage Body Weight Gain: Recovery Groups – Week 5-6	**31**
**Table 9 -** Mean Feed Intake/Cage#/Week: Main and Recovery Groups Combined – Week 1-4	**32**
**Table 10 -** Mean Feed Intake/Cage#/Week: Recovery Groups – Week 5-6	**32**
**Table 11 -** Summarised Clinical Observations: Main Group and Recovery Groups	**33**
**Table 12 -** Detailed Clinical Examination Criteria	**34**
**Table 13 -** Summarised Detailed Clinical Examinations: Main and Recovery Groups*	**36**
**Table 14 -** Summarised Functional Observations*: Main and Recovery Groups	**37**
**Table 15 -** Haematology Summary: Main Groups - Day 29	**38**
**Table 16 -** Haematology Summary: Recovery Groups - Day 43	**39**
**Table 17 -** Biochemistry Summary: Main Groups - Day 29	**40**
**Table 18 -** Biochemistry Summary: Recovery Groups - Day 43	**41**
**Table 19 -** Urinalysis Summary: Main Groups - Day 29	**42**
**Table 20 -** Urinalysis Summary: Recovery Groups - Day 43	**43**
**Table 21 -** Organ Weights Summary: Main Groups - Day 29*	**44**
**Table 22 -** Organ Weights Summary: Recovery Groups – Day 43*	**45**
**Table 23 - O**rgan Weights (% Body Weight): Main Groups –Day 29#	**46**
**Table 24 -** Organ Weight (% Body Weight): Recovery Groups – Day 43#	**47**
**Table 25 -** Gross Necropsy Summary: Main Groups – Day 29	**48**
**Table 26 -** Gross Necropsy Summary: Recovery Groups – Day 43	**49**
**Table 27 -** Histopathology Summary – Main Groups and Recovery	**50**

LIST OF APPENDICES

**Appendix A**	**Certificate of Analysis**	**1-2**
**Appendix B**	**Test Item Preparations**	**1-9**
**Appendix C**	**Study Data Spreadsheets**	**1-45**
**Appendix D**	**Haematology and Biochemistry**	**1-2**
**Appendix E**	**Histopathology**	**1-25**
**Appendix F**	**Protocol/Study Plan**	**1-29**

GOOD LABORATORY PRACTICE COMPLIANCE

This document is issued in
Accordance with NATA's
GLP requirements.
Recognised for compliance with the
OECD Principles of Good Laboratory Practice

STUDY DIRECTOR'S STATEMENT

28-Day Repeated Dose Oral Toxicity Study (OECD 407)
of c2
in Sprague Dawley Rats with a Recovery Period

I, the undersigned, hereby declare that the work was performed by me or under my supervision and that the findings provide a full and true record of the results obtained.

The study was performed in accordance with the agreed protocol and with ICP Firefly Pty Ltd Standard Operating Procedures, unless otherwise stated, and the study objectives were achieved.

The histopathological evaluation, interpretation and reporting of tissue sections was performed by Dr Kevin Isaacs in compliance with the OECD Principles of Good Laboratory Practice (Last inspected 08.08.11, The Department of Health of the Government of the United Kingdom).

The study has been conducted in compliance with the OECD Principles of Good Laboratory Practice (GLP) (revised 1997, issued Jan 1998, ENV/MC/CHEM (98)17).

______________________________________ _________________________

Fiona Brook Date
Study Director
ICP Firefly Pty Ltd
129 Queen Street
Beaconsfield NSW 2015
AUSTRALIA

QUALITY ASSURANCE STATEMENT

28-Day Repeated Dose Oral Toxicity Study (OECD 407)
of c2
in Sprague Dawley Rats with a Recovery Period

The study described in this report was subject to audit by the independent Quality Assurance Department. The findings of each audit were reported to the Study Director and Management as prescribed by the ICP Firefly Quality System.

The conduct of the audit serves to confirm that the methods, procedures, and observations are accurately described, and that the reported results accurately reflect the raw data of the study.

Type of Inspection	Inspection dates	Date reported to Study Director and Management	Auditor
**Study plan/ Protocol review**	-	29 April 2011	E. Ho
**Facility audit** (Report No. IA1237)	30 January 2012	30 January 2012	E. Ho
**Data audit report** (Report No. IA1240)	05 March 2012	14 March 2012	E. Ho
**Final report audit** (Report No. IA1240)	05 March 2012	14 March 2012	E. Ho

______________________________ ______________________

Edmund Ho Date
QA Manager
ICP Firefly Pty Ltd

REGULATORY STATEMENT

28-Day Repeated Dose Oral Toxicity Study (OECD 407)
of c2
in Sprague Dawley Rats with a Recovery Period

ICP Firefly Pty Ltd is accredited as an Animal Research Establishment (Ref. No: AW96/042) by the New South Wales Department of Primary Industries. Operation of the test facility complies with the OECD Principles of GLP (NATA accreditation no: 14320), and AS/NZS ISO 9001:2000 (NCSI Certification No: 8116). ICP Firefly Pty Ltd holds and Animal Suppliers License in accordance with the Animal Research Act 1985, the Animal Research Regulation 2005 and the Code of Practice under that regulation (Ref no. AW2007/010, Trim no 7/2288).

The experimental protocol was approved by the ICP Firefly Animal Ethics Committee, and was found to be in compliance with all appropriate regulations.

________________________________ ____________________

Dr Isabelle Meyer-Carrive Date
Managing Director
ICP Firefly Pty Ltd

ARCHIVES STATEMENT

28-Day Repeated Dose Oral Toxicity Study (OECD 407)
of c2
in Sprague Dawley Rats with a Recovery Period

All primary raw data, study records, histopathology blocks and slides and a copy of the final report will be retained in the ICP Firefly Pty Ltd archives for six years after the completion of the study. Wet tissues will be retained for six months from experimental completion date.

Access to archives is restricted to authorised personnel.

PERSONNEL INVOLVED IN THE STUDY

The following personnel are aware of, and have adhered to GLP requirements and ICP Firefly Standard Operating Procedures.

Name	Job Title
**Dr E. Rozinova**<br>BSc, PhD	Research Manager
**Ms F. Brook**<br>BSc	Senior Research Officer/<br>Study Director
**Dr R. Miller**<br>BVSc, PhD, MACVSc, Dip ACVP	Specialist Veterinary Pathologist
**Dr K. Isaacs**<br>MA, VetMB, MRCVS, FRCPath	Consultant in Toxicological Pathology/<br>Principal Investigator
**Dr T. Rothwell**<br>BVSc, PhD, DVSc, MACVSc	Veterinary Pathologist
**Mr C. Clarke**<br>BAppSc	Senior Research Scientist
**Mr E. Ho**<br>BAppSc, MBus	QA Manager
**Dr I. Meyer-Carrive**<br>BSc, MSc, PhD	Managing Director

1.0. SUMMARY

The objective of this study was to investigate the potential oral toxicity of the test item **c2** at two dose levels following repeated oral administration every three days over 28 days in the male Sprague Dawley (SD) rat.

Groups of male SD rats were administered the test item over 28 days as a suspension at doses of 1 and 20 mg/kg/every three days. A vehicle control group was administered the vehicle only (1% Carboxymethylcellulose in distilled water). The dose levels were selected based on the results of the dose range finding study (ICPQN1035.A).

Three groups of 10 rats (Vehicle, Low dose and High dose) were treated over a period of 28 days and sacrificed on Day 29 [Main study]. Two additional groups of 5 rats from the vehicle and high dose treated group were observed for a treatment-free period of 14 days and sacrificed on Day 43 to assess reversibility of any toxicity observed by Day 29 [Recovery study].

The following parameters were evaluated in all animals: mortality, daily clinical observations, weekly body weights, weekly food consumption, weekly detailed clinical examinations, functional observations, haematology, biochemistry, urinalysis, organ weights and gross necropsy on day of sacrifice. Extensive histopathology was performed on all animals in control and high dose groups sacrificed on Day 29.

The findings of all parameters except blood and urine analysis, organ weights were combined for all groups of animals until Day 29.

No mortalities related to the treatment were observed in either the vehicle or treated groups in both the main and recovery study.

The test item produced no clinical abnormalities related to treatment in any animal during the 28 -day and 42-day experimental period.

Treated and vehicle control groups displayed comparable body weight gains over the 28-day and 42-day experimental period .

Feed intake was similar in control and treated groups for the 28-day experimental period and 42-day experimental period.

Urinalysis and blood analysis revealed no test item related effects.

No macroscopic abnormalities related to treatment were identified during necropsy of the surviving animals.

There was no evidence of any test item related effect on all the tissues examined histopathologically.

Under the conditions of the study, the test item, **C2**, administered 10 times over a period of 28 Days at doses of 1 and 20 mg/kg in the Sprague Dawley rat, produced no toxic effects.

2.0. INTRODUCTION

2.1. Sponsor

St George Clinical School
Clinical Sciences (WR Pitney) Building
Short Street
St George Hospital
Kogarah NSW 2217
AUSTRALIA.

2.2. Study Number

ICP Firefly Study No: ICPQN1035.B

2.3. Study Director

Fiona Brook, BSc.

2.4. Rationale for the study

The aim of this study was to evaluate the potential toxicity of test item **c2** following repeated oral administration every three days over 28 days at two (2) dose levels: 1 and 20 mg/kg in male SD rats, when compared with a vehicle treated control group. The dose levels were selected by the Sponsor following a 28-day sighting study (ICPQN1035.A).

2.5. Study timetable

Protocol acceptance date	29 April 2011
Experimental start date	06 January 2012
Experimental (in-life) completion date	22 February 2012
Experimental completion date	12 March 2012
Study completion date	16 March 2012

2.6. Study integrity

The following deviations from the study plan were made:

Table 1 - Deviations

No	Date	Deviation
1	16.2.2012	• Histopathology processing: Initial sections from block Nos 10083, 10120, 10126, 10140 and 10210 were not considered to be of good quality and re-cuts were made. New slide numbers allocated were 10083-1, 10120-1, 10126-1, 10140-1 and 10210-1, respectively.<br>• Histopathology processing: Cassette Nos 10185 and 10257 did not process satisfactorily. New fixed tissue was prepared: Cassette Nos 10325 and 10326, respectively. Cassette No 10326 did not include brain stem section, there was no remaining tissue.
2	17.2.2012	▪ Histopathology processing: Initial sections from block Nos 10266, 10279, 10280, 10294, 10308, 10321 and 10322 were not considered to be of good quality and re-cuts were made. New slide numbers allocated were 10266-1, 10279-1, 10280-1, 10294-1, 10308-1, 10321-1 and 10322-1, respectively.<br>▪ Cassette Number 10132 – Thyroid tissue missing after embedding. Slide number 10327 prepared from new fixed tissue.

No other deviations were made during the study.

There were no other known circumstances that might have affected the quality and integrity of the study.

2.7. Study Facility

ICP Firefly Pty Ltd
129 Queen Street
Beaconsfield NSW 2015
AUSTRALIA.

2.7.1. Test site 1

IDEXX-Laboratories
The Metro Centre
Unit 20, 38-46 South Street
Rydalmere NSW 2116
AUSTRALIA

2.7.1.1. Phase of study

Blood analysis, submission of raw data for inclusion in the final report.

2.7.2. Test site 2

Toxicological Histopathologist
14 Rossett Park Road
Harrogate
North Yorkshire HG2 9NP
UNITED KINGDOM

2.7.2.1. Phase of study

Histopathology slide examination and GLP compliant report (Project No: K12/002) for inclusion in final report.

2.7.2.2. Principal Investigator

Dr Kevin Isaacs.

2.7.2.3. Test Site QA

Mrs Hazel Isaacs.

2.8. Definitions

2.8.1. Dose

Dose is the amount of the test item administered. Dose is expressed as weight (g, mg) or as weight/weight of test animal (e.g. mg/kg).

2.8.2. Dosage

Dosage is a general term comprising dose, its frequency and the duration of dosing.

2.8.3. Evident toxicity

Evident toxicity is a general term describing clear signs of toxicity following administration of a test item. These should be sufficient for hazard assessment and should be such that an increase in the dose administered can be expected to result in the development of severe toxic signs and probable mortality.

2.8.4. Repeated dose toxicity

Repeated dose toxicity is the adverse effects occurring within a short time of several repeated administrations of a test item over a defined period.

2.8.5. Test item

The test item is the article that is the subject of the study.

2.8.6. Vehicle

Vehicle is any agent, which serves as a carrier used to mix, disperse, or solubilize the test item or reference item to facilitate the administration/application to the test system.

2.9. Principle of the test method

The test item suspension or vehicle was administered orally by gavage every three days for 28 days to three groups of male rats at two dose levels (low - 1 and high - 20 mg/kg) to assess toxicity. Two vehicle control groups were dosed with the vehicle. A recovery group was included to investigate reversibility of any toxicity and persistence or delayed occurrence of toxicity.

Daily clinical observations were performed. Weekly detailed clinical examinations were performed on all animals. Functional observations were performed in the last week of treatment. Body weights were monitored weekly throughout the study. Feed intake was measured weekly.

Gross necropsy was conducted in all animals that died or were killed during the study. Full blood analysis and detailed histopathology was performed on conclusion of the main study.

All tissues and carcasses were placed in 10% formalin and retained for a period of 6 months.

3.0. MATERIALS AND METHODS

3.1. Regulatory references

3.1.1. Test guidelines

The study was conducted according to the OECD Guidelines for the Testing of Chemicals, No.407 - Repeated Dose 28-Day Oral Toxicity Study in Rodents. [Adopted: 3 October 2008].

3.1.2. Good Laboratory Practice

This study was conducted in accordance with the Organization of Economic Cooperation and Development (OECD) Principles of Good Laboratory Practice (as revised in 1997) ENV/MC/CHEM (98) 17. The study was performed in accordance with the agreed protocol and with ICP Firefly Pty Ltd Standard Operating Procedures (SOPs).

3.1.3. Animal Welfare Act compliance

The experimental protocol was approved by the ICP Firefly Animal Ethics Committee, and was found to be in compliance with the Animal Research Act 1985, the Animal Research Regulation 2005 and the Code of Practice under that Regulation. It was granted Approval Number ICPE134.

3.2. Test items

Table 2 - Test item

ICP Firefly No	Identification	Batch No	Physical Description	Storage
TI/458	c2 Cyclo(2-Nal-leu-Ser-2-Nal-Arg) acetate salt	1037619	White powder	-20ºC

The Sponsor provided a Certificate of Analysis for the test item, which is attached as an appendix to the report (**Appendix A** Certificate of Analysis).

3.3. Vehicle

Table 3 - Vehicle

ICP Firefly No	Identification	Batch No	Storage	Manufacturer
	1% carboxymethylcellulose (CMC)	102K0113	2 - 8˚C	Sigma

3.4. Animals

3.4.1. Justification for species selection

The Specific Pathogen Free [SPF] male Sprague Dawley rat was selected since it is widely accepted and recognised by international guidelines as an appropriate experimental model for this type of study.

3.4.2. Species, strain source and housing

A total of 40 young adult Specific Pathogen Free Male Sprague Dawley rats weighing between 189 and 212 grams the beginning of the experiment were used for the study. The animals were obtained from Animal Resources Centre (ARC), WA, Australia.

The animals were acclimatised to the laboratory conditions for at least 5 days before commencement of the study. All animals were examined during the acclimatisation period.

Acclimatisation period observations were documented and included as spreadsheets in an appendix to the report (**Appendix C** – Study Data Spreadsheets). The rats were housed on

bedding of recycled paper pellets in groups of five in polyethylene boxes with stainless steel lids.

Animals were identified with a unique identification number by marking their tails with permanent marker pens. Each box was labelled with the study number, animal numbers, gender, animal supplier, Study Director's name and Animal Ethics Committee approval number.

3.4.3. Treatment allocation

The animals were assigned to the treatment groups without bias according to the schedule presented in **Table 4** below:

Table 4 - Animal allocation

Group No	Number of animals/sex	Unique animal number	Dose (mg/kg)	Concentration	Days of treatment	Days of observation
**Main Groups**						
1	10 males	R2409 – R2418	Vehicle	-	28	28
2	10 males	R2419 – R2428	Low<br>1 mg/kg	0.01 % w/w	28	28
3	10 males	R2429 – R2438	High<br>20 mg/kg	0.2% w/w	28	28
**Recovery Groups**						
4	5 males	R2439 – R2443	Vehicle	-	28	42
5	5 males	R2444 – R2448	High<br>20 mg/kg	0.2% w/w	28	42

3.4.4. Feed and water

The rats were fed on a diet of Rat & Mouse pellets (Gordon's Specialty Stock Feeds, DOM: 02/11/2011) and provided with tap water *ad libitum.*

Water is analysed regularly for bacterial contaminants. Water quality was within acceptable quality range (last reported 07/10/2011).

3.4.5. Environment

Environmental controls for the animal room were set to maintain a temperature of 22 ± 3°C and the relative humidity ranges from 30-70%. The automated light/dark cycle was 12 hours light/12 hours dark.

3.5. Preparations

3.5.1. Test item

The test item was prepared as a suspension at the required concentration immediately prior to administration and the weight/volume equivalence was calculated. The test item preparation procedures were recorded and are included as an appendix to the report (**Appendix B** Test Item Preparation).

3.5.2. Vehicle

1% carboxymethylcellulose (CMC) in distilled water was administered at a volume of 10.23 mL/kg. The vehicle preparation procedures were documented and included as an appendix to the report (**Appendix B** Test Item Preparation).

3.6. Procedure for test item administration

The calculated volumetric dose was administered to each rat orally by gavage, using a plastic intragastric cannula attached to a 3 mL syringe.

Two dose levels of 1 and 20 mg/kg of the test item suspension were administered to each rat every 3 days (total 10 doses) for 28 days. This corresponded to volumetric dose of 10.09 and 10.23 mL/kg. The volumetric dose administered to each animal was calculated on Days 1, 8, 15 and 22 and is presented in a spreadsheet as an appendix to the report (**Appendix C** Study Data Spreadsheets).

3.7. Observation of animals

3.7.1. Body weights

Body weights were determined immediately prior to the first test item administration (Day 1), and weekly thereafter (Day 8, 15, 22 and 28 for all groups; and Days 36 and 42 for recovery groups). All animals were weighed after fasting i.e. on the day of sacrifice (main groups: Day 29; recovery groups: Day 43).

The body weights were recorded and included as spreadsheets in an appendix to the report (**Appendix C** Study Data Spreadsheets).

3.7.2. Feed consumption

Feed consumption was monitored weekly over the 28-day experimental period in all groups and was continued for a further 2 weeks in the recovery groups (42-day experimental period). Feed intake was monitored by providing a known mass of feed to each cage and subtracting the mass remaining at the end of the weekly period. Animals were fasted overnight on either Day 28 (main groups) or Day 42 (recovery groups).

Feed intake was recorded for each group and included as a spreadsheet in an appendix to the report (**Appendix C** Study Data Spreadsheets).

3.7.3. Clinical observations

The animals were observed daily at approximately the same time each day, between 1 and 3 hours after dosing, for a period of 28 days for the main groups and 42 days for the recovery groups and on the day of sacrifice. Particular care was taken to look for signs of toxicity and abnormal behaviour. Skin and fur, eyes and mucous membranes, respiratory, circulatory, autonomic and central nervous systems, somatomotor activity and behaviour patterns were monitored. Particular attention was paid to observation of tremor, convulsion, salivation, diarrhoea, lethargy, sleep and coma.

3.7.4. Detailed clinical examinations

Detailed clinical examinations were performed prior to commencement of the study and weekly thereafter (Weeks 1 to 4) of the study in the main groups and the recovery groups. The following parameters were assessed: posture, gait, clonic convulsion, tonic convulsions, biting, removing rat from cage, handing rat in hand, lacrimation, fur appearance, salivation, defecation, urination, grooming, palpebral closure, pupil response, eye, respiration, behaviour and rearing.

The examinations were recorded in the form of a spreadsheet and attached as an appendix to the report (**Appendix C** Study Data Spreadsheets).

3.7.5. Functional observation

Functional observation was performed in the last week of the 28-day experimental period for each animal. The following parameters are assessed: arousal, finger snap, approach response, touch response, tail pinch, extensor thrust, limb rotation, catalepsy, righting reflex, supine drop, grip strength test.

The observations were recorded in the form of a spreadsheet and attached as an appendix to the report (**Appendix C** Study Data Spreadsheets).

3.8. Blood sampling

Blood analyses (haematology and biochemistry) were performed on all animals on Day 29 (main groups) or Day 43 (recovery groups). Approximately 4 mL of blood was taken from each animal by cardiac puncture using a 10 mL syringe and 21-gauge needle. The procedure involved anaesthetising by intraperitoneal injection of sodium pentobarbitone using a 2 mL sterile plastic syringe attached to a 26-gauge needle (Terumo).

For each blood sampling, the blood was divided into two aliquots: (i) one was placed into a tube containing EDTA (for whole blood and EDTA plasma), (ii) one aliquot into a Li-heparin tube.

3.9. Blood analyses

3.9.1. Haematology analysis

The following parameters were analysed: Red blood cell (RBC), haemoglobin (Hb), haematocrit (Hct), mean corpuscular volume (MCV), mean corpuscular haemoglobin (MCH), mean corpuscular haemoglobin concentration (MCHC), platelet count (PLT), mean platelet volume (MPV), white blood count (WBC), neutrophil (Neut), lymphocyte (Lymph), monocytes (Mono), eosinophil (Eos), basophil (Baso).

Results and methods of analysis were attached in an appendix to the report (**Appendix D** Haematology and Biochemistry). Blood smears for cell morphology were prepared. By convention only abnormal findings on smear evaluation were reported.

3.9.2. Biochemistry analysis

The following parameters were analysed: glucose (Glu), urea, creatinine (Creat), total protein (TP), albumin (ALB), albumin/globulin ratio (A:G), total bilirubin (TBil), alkaline phosphatase (ALP), aspartate aminotransferase (AST), alanine aminotransferase (ALT), creatine kinase (CK), cholesterol (Chol), calcium (Ca), phosphate (Phos), Ca:P ratio, sodium (Na), potassium (K), Na:K ratio, chloride (Cl), globulin (Glob), gamma glutamyl transferase (GGT) and triglycerides (Trig).

Results and methods of analysis were attached in an appendix to the report (**Appendix D** Haematology and Biochemistry).

3.10. Urinalysis

Urine was collected at the end of the study (on Days 29 and 43 for main and recovery groups, respectively) by placing a urine collection tube at the opening of the urethra and any urine expressed by the animal was collected. If no urine came out, pressure was gently applied on the bladder area of the abdomen.

Urine was also collected by bladder puncture at the time of death to maximise the volume collected. The animal was placed in lateral recumbency and the abdominal cavity opened. A needle attached to the syringe was inserted into the bladder and urine withdrawn with the plunger. The urine sample was expelled from the syringe into the collection tube.

The urine samples were analysed, individual results are presented as an appendix to the report (**Appendix C** Study Data Spreadsheets).

3.11. Gross necropsy

Following sacrifice by intraperitoneal injection of sodium pentobarbitone a gross necropsy was performed. This included determination of the wet weight of the liver, kidneys, adrenals, testes, spleen, thymus, heart, epididymis, prostate + seminal vesicles with coagulating glands as whole and brain. Observations were recorded of the abdominal, thoracic and cranial cavities and major

organs. Tissues and carcasses were placed in 10% formalin. The tissues to be processed for histopathological examinations were fixed for at least 48 hours prior to cassette preparation.
The organ weights and gross pathology changes are included in a spreadsheet as an appendix to the report (**Appendix C** Study Data Spreadsheets).

3.12. Tissue processing / Slide preparation

The tissues were placed in labelled cassettes and placed in an automated tissue processor and taken through several stages of alcohol dehydration, solvent clearing and wax infiltration. Infiltrated tissues were embedded in wax blocks, which were sectioned on a microtome at between 4 and 6 μm. Sections were placed on pre-labelled glass slides and stained with haematoxylin and eosin (H&E). Stained sections were dehydrated, then solvent cleared and cover slipped.

3.13. Histopathology

Histopathological examinations were performed on all animals in the vehicle control and high dose treated groups (main study) on the following tissues/organs: adrenal glands, bone marrow, brain, epididymis, heart, kidney, liver, lung, lymph node: mesenteric, prostate, seminal vesicles with coagulating glands, small and large intestine (including Peyer's patches), spinal cord, spleen, stomach, thymus, thyroid gland, trachea, testes and urinary bladder. Slides were evaluated for histology changes under light microscopy at a magnification of 100 to 1000 times. Individual histopathology results and a summary are attached as an appendix to the report (**Appendix E** Histopathology).

3.14. Statistical analyses

Clinical observations, detailed clinical observations, functional observations, blood and urine analyses and histopathology were summarised by descriptive analysis.
Statistical analyses, consisting of unpaired t-tests, were performed on measured variables: percentage body weight gains, food consumption and organ weights as a percentage of body weight. A significance level of $P < 0.05$ was used for all tests.

4.0. RESULTS AND DISCUSSION

Results are presented as a 28-day experimental period (Main study groups): **vehicle control - Group 1** and **treated** [**Group 2, low dose -** 1 mg/kg**,** and **Group 3, high dose -** 20 mg/kg**,**] and as a 42-day experimental period (Recovery groups): **vehicle control - Group 4 and high dose - Group 5.**

Combined analyses of Main study and Recovery groups were made during the 28-day observation period for the following parameters: mortality, body weight, feed intake, clinical observation, detailed and functional clinical observations.

4.1. Mortality

No mortalities related to the treatment were observed during the study period in either the main or recovery groups.

4.2. Body weights

Individual animal data are presented in a spreadsheet as an appendix to the report (**Appendix C** Study Data Spreadsheets). Summarised data are presented in **Table 5** – Mean Body Weights: Main and Recovery Groups Combined (Week 1 - 4), **Table 6** - Mean Body Weights: Recovery Groups (Week 5 - 6), **Table 7** - Percentage Body Weight Gain: Main and Recovery Groups Combined (Week 1 - 4) and **Table 8** – Percentage Body Weight Gain: Recovery Groups (Week 5 - 6).

4.2.1. Main and Recovery Groups: Week 1-4

4.2.1.1. Treated (Gr 2 - 1 mg/kg) vs. Vehicle control (Grs 1 & 4)

The treated males had a lower trend in body weight gain in Week 1, when compared to vehicle control males (19.6 ± 2.49 vs 22.4 ± 2.50%).

Over the 4-week observation period the treated animals had a statistically significantly lower percentage body weight gain when compared with vehicle (Groups 1 and 4) control rats (55.4 ± 8.84 vs 63.8 ± 8.65%)

4.2.1.2. Treated (Grs 3 & 5 - 20 mg/kg) vs. Vehicle control (Grs 1 & 4)

Over the 4-week observation period no significant difference was found between treated animals and vehicle control animals.

4.2.2. Recovery period: Week 5-6

4.2.2.1. Treated (Gr 5 - 20 mg/kg) vs. Vehicle control (Gr 4)

Over the 6-week observation period no significant difference between treated animals and vehicle control animals was found during the recovery period.

4.2.3. Discussion

Over the 28-day observation period, a lower body weight gain was observed in the low dose treated animals when compared to the vehicle control animals. This observation was not considered to be of any clinical significance.

After the recovery period, no statistical significant difference between test item and vehicle treated animals could be observed.

4.3. Feed intake

Individual animal data are presented in a spreadsheet as an appendix to the report (**Appendix C** Study Data Spreadsheets) and summarised data are presented in **Table 9** – Mean Feed Intake/ Cage/Week: Main and Recovery Groups Combined – Week 1-4 and **Table 10** – Mean Feed Intake/Cage/Week: Recovery Groups – Week 5-6.

4.3.1. Main and Recovery Groups: Week 1–4

4.3.1.1. Treated (Gr 2 - 1 mg/kg) vs. Vehicle control (Grs 1 & 4)

There were no statistically significant differences between groups in feed intake over the 28-day observation period.

4.3.1.1. Treated (Grs 3 & 5 - 20 mg/kg) vs. Vehicle control (Grs 1 & 4)

There were no statistically significant differences between groups in feed intake over the 28-day observation period.

4.3.1.1. Recovery period: Week 5-6

4.3.2.1. Treated (Gr 5 - 20 mg/kg) vs Vehicle control (Gr 4)

There were no statistically significant differences between groups in feed intake during the recovery period.

4.3.3. Discussion

No reduction in feed intake was observed during the 28-day (4-week) observation period and the recovery period (6-week) when compared to the vehicle control animals.

4.4. Clinical observations

Individual animal data are presented in a spreadsheet as an appendix to the report (**Appendix C** Study Data Spreadsheets) and summarised data are presented in **Table 11** -Summarised Clinical Observations: Main and Recovery Groups.

4.4.1. Main and Recovery Groups: Week 1-4

4.4.1.1. Vehicle control (Gr 1 & 4)

On Day 21-22, one control rat had porphyrin discharge around right eye. No other clinical signs were observed in any of the animals in the control groups over the 28-day observation period.

4.4.1.2. Treated (Gr 2 - 1 mg/kg)

No clinical signs were observed in any of the animals in the control groups over the 28-day observation period.

4.4.1.3. Treated (Gr 3 & 5 - 20 mg/kg)

No clinical signs were observed in any of the animals in the control group over the 28-day observation period.

4.4.2. Recovery Groups: Week 5-6

4.4.2.1. Vehicle control (Gr 4)

No clinical signs were observed in any of the animals in the control group during the recovery period.

4.4.2.2. Treated (Gr 5 - 20 mg/kg)

No clinical signs were observed in any of the animals in the treated group during the recovery period.

4.4.3. Discussion

They were no remarkable differences in clinical observations between vehicle and test item treated animals. The test item did not have an adverse effect on clinical signs.

4.5. Detailed clinical observations

Individual animal data are presented in a spreadsheet as an appendix to the report (**Appendix C** Raw Data Spreadsheets). The Detailed Clinical Examination Criteria are described in **Table 12** - Summarised data are presented in **Table 13** – Summarised Detailed Clinical Examinations: Main and Recovery Groups.

4.5.1. Vehicle control (Grs 1 & 4)

The vehicle did not affect any of the parameters measured in the detailed clinical examinations performed during Weeks 1 to 4.

4.5.2. Treated (Gr 2 - 1 mg/kg)

In treated animals the test item had no effect on any of the detailed clinical examinations parameters measured during Weeks 1 to 4.

4.5.3. Treated (Grs 3 & 5 - 20 mg/kg)

In treated animals the test item had no effect on any of the detailed clinical examinations parameters measured during Weeks 1 to 4.

4.5.4. Discussion

The test item had no obvious effect on any of the parameters measured in the detailed clinical examinations performed during Weeks 1 to 4.

4.6. Functional observations

Individual animal data are presented in a spreadsheet as an appendix to the report (**Appendix C** Study Data Spreadsheets) and in **Table 14** – Summarised Functional Observations: Main and Recovery Groups.

4.6.1 Vehicle control (Grs 1 & 4)

No abnormalities were detected in the vehicle control rats in any of the functional observation parameters measured during Week 4.

4.6.2 Treated (Gr 2 - 1 mg/kg)

No abnormalities were detected in the treated rats in any of the functional observation parameters measured during Week 4.

4.6.3 Treated (Grs 3 & 5 - 20 mg/kg)

No abnormalities were detected in the treated rats in any of the functional observation parameters measured during Week 4.

4.6.4 Discussion

The test item had no effect on any of the parameters measured in the functional observations tests performed during Week 4.

4.7. Blood haematology analysis

Individual animal data are presented in a spreadsheet as an appendix to the report (**Appendix D** Haematology and Biochemistry) and in **Table 15** - Haematology Summary: Main Groups – Day 29 and **Table 16** - Haematology Summary: Recovery Groups – Day 43.

4.7.1. Main Groups – Day 29

4.7.1.1. Vehicle control (Gr 1)

One animal (R2414) has significantly lower WBC (and hence absolute numbers of various leukocytes from differential) than its group mates and is considered to be an outlier.

4.7.1.2. Treated (Gr 2 - 1 mg/kg) vs Vehicle control (Gr 1)

No differences between treated and control groups were found.

4.7.1.3. Treated (Gr 3 - 20 mg/kg) vs Vehicle control (Gr 1)

No differences between treated and control groups were found.

4.7.2. Recovery Groups – Day 43

4.7.2.1. Treated (Gr 5 - 20 mg/kg) vs Vehicle control (Gr 4)

No differences between treated and control groups were found.

4.7.3. Discussion

There was no obvious test item related effect. Any differences were small and interpreted to be not clinically relevant.

4.8. Blood biochemistry analysis

Individual animal data are presented in a spreadsheet as an appendix to the report (**Appendix D** Haematology and Biochemistry) and in **Table 17** – Biochemistry Summary: Main Groups – Day 29 and in **Table 18** – Biochemistry Summary: Main Groups – Day 43.

4.8.1. Main Groups – Day 29

4.8.1.1. Treated (Gr 2 - 1 mg/kg) vs Vehicle control (Gr 1)

There was no obvious test item related effect.

4.8.1.2. Treated (Gr 3 - 20 mg/kg) vs Vehicle control (Gr 1)

There was no obvious test item related effect.

4.8.2. Recovery groups – Day 43

4.8.2.1. Treated (Gr 5 - 20 mg/kg) vs Vehicle control (Gr 4)

No differences of any clinical significance were seen between treated and control groups.

4.8.3. Discussion

There was no treatment related changes in any of the treatment groups. Any differences were small and interpreted to be not clinically relevant.

4.9. Urinalysis

Individual animal data are presented in a spreadsheet as an appendix to the report (**Appendix C** Study Data Spreadsheets) and in **Table 19** – Urinalysis Summary: Main Groups – Day 29 and **in Table 20** – Urinalysis Summary: Recovery Groups – Day 43.

4.9.1. Main Groups – Day 29

4.9.1.1. Treated (Gr 2 - 1 mg/kg) vs Vehicle control (Gr 1)

All urine parameters appeared similar in the vehicle control and test item treated groups.

4.9.1.2. Treated (Gr 3 - 20 mg/kg) vs Vehicle control (Gr 1)

All urine parameters appeared similar in the vehicle control and test item treated groups.

4.9.2. Recovery Groups – Day 43

4.9.2.1. Treated (Gr 5 - 20 mg/kg) vs Vehicle control (Gr 4)

All urine parameters appeared similar in vehicle control and test item animals.

4.9.3. Discussion

The test item had no obvious effects on urine parameters analysed.

4.10. Gross necropsy

Individual animal data for gross necropsy and organ weights are presented in a spreadsheet as an appendix to the report (**Appendix C** Study Data Spreadsheets). Summarised data are presented in **Table 21**– Organ Weights Summary: Main Groups – Day 29, **Table 22**– Organ Weights Summary: Recovery Groups – Day 43, **Table 23** – Organ Weight (% Body Weight): Main Groups – Day 29, and **Table 24** – Organ Weight (% Body Weight): Recovery Groups – Day 43. Organ weights were adjusted for body weights measured on day of sacrifice i.e. after fasting, Day 29 for main study groups and Day 43 for recovery groups.

Gross necropsy data are presented in **Table 25** - Gross Necropsy Summary: Main Groups – Day 29 and **Table 26** - Gross Necropsy Summary: Recovery Groups – Day 43.

4.10.1. *Main Groups: sacrificed - Day 29*

4.10.1.1. *Vehicle control (Gr 1)*

Macroscopic changes: No gross abnormalities were observed in any organ.

4.10.1.2. *Treated (Gr 2 - 1 mg/kg) vs Vehicle control (Gr 1)*

Macroscopic changes: No gross abnormalities were observed in any organ.

Organ weights: The test item did not have any effect of organ weights when expressed as a percentage of body weight.

4.10.1.3. *Treated (Gr 3 - 20 mg/kg) vs Vehicle control (Gr 1)*

Macroscopic changes: No gross abnormalities were observed in any organ.

Organ weights: The test item did not have any effect of organ weights when expressed as a percentage of body weight.

4.10.2. *Recovery Groups – sacrificed - Day 43:*

4.10.2.1. *Vehicle control (Gr 4)*

Macroscopic changes: No gross abnormalities were observed in any organ.

4.10.2.2. *Treated (Gr 5 - 20 mg/kg) vs Vehicle control (Gr 4))*

Macroscopic changes: No gross abnormalities were observed in any organ.

Organ weights: The test item did not have any effect of organ weights when expressed as a percentage of body weight.

4.10.3. *Discussion*

The test item had no obvious effects on organ weight when expressed as a percentage of body weight.

4.11. Histopathology

Individual animal data for histopathology are presented in a spreadsheet as an appendix to the report (**Appendix E** Histopathology). Summarised data are presented in **Table 27** – Histopathology Summary: Main Groups. Histopathology examinations were performed on all animals in the vehicle control (Gr 1) and treated (Gr 3) 20 mg/kg dose animals in the main study. No other organs needed to be examined. Treatment related results only are reported below.

4.11.1. *Alimentary and associated tissues Gr 1 and Gr 3*

4.11.1.1. *Stomach*

No treatment-related differences were observed between the treated and vehicle control groups.

4.11.1.2. *Small intestine: ileum*

No treatment-related differences were observed between the treated and vehicle control groups.

4.11.1.3. *Large intestine: colon*

No treatment-related differences were observed between the treated and vehicle control groups.

4.11.1.4. *Liver*

No treatment-related differences were observed between the treated and vehicle control groups.

4.11.2. Cardiovascular and respiratory Gr 1 and Gr 3

4.11.2.1. Heart

No treatment-related differences were observed between the treated and vehicle control groups.

4.11.2.2. Trachea

No treatment-related differences were observed between the treated and vehicle control groups.

4.11.2.3. Lung

No treatment-related differences were observed between the treated and vehicle control groups.

4.11.3. Endocrine Gr 1 and Gr 3

4.11.3.1. Adrenal glands

No treatment-related differences were observed between the treated and vehicle control groups.

4.11.3.2. Thyroid gland

No treatment-related differences were observed between treated and vehicle control groups.

4.11.4. Genito-urinary Gr 1 and Gr 3

4.11.4.1. Kidney

No treatment-related differences were observed between treated and vehicle control groups.

4.11.4.2. Epididymis

No treatment-related differences were observed between treated and vehicle control groups.

4.11.4.3. Prostate

No treatment-related differences were observed between treated and vehicle control groups.

4.11.4.4. Seminal vesicle with coagulating glands

No treatment-related differences were observed between treated and vehicle control groups.

4.11.4.5. Urinary bladder

No treatment-related differences were observed between treated and vehicle control groups.

4.11.4.6. Testes

No treatment-related differences were observed between treated and vehicle control groups.

4.11.5. Lymphoid and haematopoietic tissues Gr 1 and Gr 3

4.11.5.1. Bone marrow

No treatment-related differences were observed between treated and vehicle control groups.

4.11.5.2. Spleen

No treatment-related differences were observed between treated and vehicle control groups.

4.11.5.3. Peyer's Patch

No treatment-related differences were observed between treated and vehicle control groups.

4.11.5.4. Thymus

No treatment-related differences were observed between treated and vehicle control groups.

4.11.5.5. Lymph node: mesenteric

No treatment-related differences were observed between treated and vehicle control groups.

4.11.6. Nervous and special tissues Gr 1 and Gr 3

4.11.6.1. Brain stem

No treatment-related differences were observed between treated and vehicle control groups.

4.11.6.2. Cerebrum

No treatment-related differences were observed between treated and vehicle control groups.

4.11.6.3. Cerebellum

No treatment-related differences were observed between treated and vehicle control groups.

4.11.6.4. Spinal cord

No treatment-related differences were observed between treated and vehicle control groups.

5.0. CONCLUSION

The objective of this study was to investigate the potential oral toxicity of the test item **C2** at two dose levels in the Specific Pathogen Free Sprague Dawley male rat.

Under the conditions of the study the test item, **c2, Batch: 1037619** administered at the two dose levels of 1 and 20 mg/kg for 28 days in the male Sprague Dawley rat, produced no toxic effects

Table 5 - Mean Body Weights: Main and Recovery Groups Combined – Week 1-4

Day	Mean ± SD (g)		
	**Groups 1 & 4** Vehicle control	**Group 2** 1 mg/kg TI/458	**Groups 3 & 5** 20 mg/kg TI/458
**Day 1**	258 ± 9.5	261 ± 14.0	257 ± 11.3
**Day 8**	316 ± 12.4	312 ± 15.3	312 ± 14.1
**Day 15**	362 ± 16.4	356 ± 22.3	354 ± 20.8
**Day 22**	398 ± 22.6	387 ± 24.9	392 ± 24.5
**Day 28**	423 ± 27.8	406 ± 31.0	414 ± 27.3
**Day 29***	401 ± 20.3	395 ± 28.0	395 ± 31.5

Note: SD, Standard deviation; *, Groups 1 - 3 weighed Day 29 (body weights after overnight fasting).

Table 6 - Mean Body Weights: Recovery Groups – Week 5 - 6

Day	Mean ± SD (g) (n=5)	
	**Group 4** Vehicle Control	**Group 5** 20 mg/kg TI/458
**Day 36**	463 ± 47.0	458 ± 12.0
**Day 42**	487 ± 55.8	480 ± 11.6
**Day 43***	468 ± 54.3	460 ± 12.3

Note: SD, Standard deviation; *, body weights after overnight fasting.

Table 7 - Percentage Body Weight Gain: Main and Recovery Groups Combined – Week 1-4

**Week**	**Mean ± SD (g)**		
	**Groups 1 & 4** Vehicle control	**Group 2** 1 mg/kg TI/458	**Groups 3 & 5** 20 mg/kg TI/458
**Week 1**	22.4 ± 2.50	19.6 ± 2.49* P=0.0141	21.4 ± 2.45
**Week 2**	14.7 ± 2.01	13.8 ± 2.51	13.5 ± 2.39
**Week 3**	9.8 ± 2.35	9.0 ± 1.96	10.9 ± 2.47
**Week 4**	6.2 ± 1.35	4.7 ± 3.0	5.5 ± 1.39
**Week 1-4**	63.8 ± 8.65	55.4 ± 8.84* P=0.0299	61.4 ± 9.23

Note: SD, standard deviation of the mean; Percentage body weight gain = (Wy – Wx) / Wx *100;
*, significantly different from control mean, P < 0.05.

Table 8 - Percentage Body Weight Gain: Recovery Groups – Week 5 - 6

**Week**	**Mean ± SD (g)** (n=5)	
	**Group 4** Vehicle Control	**Group 5** 20 mg/kg TI/458
**Week 5**	6.58 ± 0.935	7.82 ± 1.202
**Week 6**	5.08 ± 1.689	4.94 ± 0.627
**Week 1-6**	91.1 ± 16.51	86.7 ± 7.72

Note: SD, standard deviation of the mean; Percentage body weight gain = (Wy – Wx) / Wx *100;

Table 9 - Mean Feed Intake/Cage#/Week: Main and Recovery Groups Combined – Week 1 - 4

Week	Mean ± SD (g)		
	**Groups 1 & 4** Vehicle control	**Group 2** 1 mg/kg TI/458	**Groups 3 & 5** 20 mg/kg TI/458
**Week 1**	810 ± 25.5	801 ± 26.9	819 ± 55.4
**Week 2**	862 ± 39.4	839 ± 12.0	877 ± 45.1
**Week 3**	891 ± 44.3	845 ± 0	916 ± 35.2
**Week 4**	822 ± 51.4	775 ± 27.6	815 ± 42.0
**Week 1-4**	3385 ± 131.2	3259 ± 66.5	3427 ± 174.5

Note: #, 5 animals/cage; SD, Standard deviation of the mean

Table 10 - Mean Feed Intake/Cage#/Week: Recovery Groups – Week 5-6

Week	Mean ± SD (g)	
	**Group 4** Vehicle Control	**Group 5** 20 mg/kg TI/458
**Week 5**	1053	1013
**Week 6**	858	802
**Week 1-6**	5440	5180

Note: #, 5 animals/cage; SD, Standard deviation of the mean.

Table 11 - Summarised Clinical Observations: Main Group and Recovery Groups

Days	Group/DOSE<br>Animal Number				
	**Group 1**<br>Vehicle control	**Group 2**<br>1 mg/kg<br>TI/458	**Group 3**<br>20 mg/kg<br>TI/458	**Group 4**<br>Vehicle control	**Group 5**<br>20 mg/kg<br>TI/458
	**R2409-R2418**	**R2419-R2428**	**R2429-R2438**	**R2439-R2443**	**R2444-R2448**
**Day 1-7**	NCA	NCA	NCA	NCA	NCA
**Day 8-14**	NCA	NCA	NCA	NCA	NCA
**Day 15-21**	NCA<br>P - (R2410)	NCA	NCA	NCA	NCA
**Day 22-28**	NCA	NCA	NCA	NCA	NCA
**Day 29-35**	#	#	#	NCA	NCA
**Day 36-43**	#	#	#	NCA	NCA

Note: #, Sacrificed on Day 29; NCA, no clinical abnormalities; P, porphyrin around the right eye on Day 21-22.

Table 12 - Detailed Clinical Examination Criteria

**POSTURE** When first approached by the observer	
**Scale**	**Description**
1	Flattened, limbs may be spread out
2	Lying on side
3	Curled up, often asleep (normal)
4	Sitting but with head hung down
5	Sitting normally. Feet tucked in (normal)
6	Sitting or standing alert, watching and sniffing at observer (normal)
7	Rearing (normal)
**GAIT**	
1	Normal
2	Ataxia, excessive sway, rocks or lurches as rat proceeds forward
3	Body drags, stomach makes contact with surface, some body sway
4	Hind limbs splayed or dragging, body makes contact with surface
5	Front limbs dragging, unable to support weight
**TOTAL GAIT SCORE**	
1	Normal
2	Slightly impaired
3	Somewhat impaired
4	Totally impaired
**CLONIC CONVULSIONS**	
1	No clonic convulsions
2	"Chewing", clonus of the jaws
3	Mild clonic tremors of limbs (contractions followed by relaxations)
4	Repetitive clonic tremors of whole body
**TONIC CONVULSIONS**	
1	No tonic convulsions
2	Tonic, constant contraction or extension of hind limb muscles
3	Opisthotonos - head, body, and limbs rigidly arched backward
4	Emprosthotonos - head, body and limbs extended forward
5	Popcorn - Rat repeatedly pops in air
6	Asphyxial - bout of severe clonic - tonic convulsions resulting in difficult respiration, post-ictal depression, or death
**BITING**	
1	No biting
2	Biting of cages
3	Self-destructive biting (tail, paws, etc)
**REMOVING RAT FROM CAGE**	
1	Very easy (rat sits quietly, allows observer to pick him up)
2	Easy (vocalisations, without resistance to being picked up)
3	Moderately difficult (rat rears, often following observer's hand)
4	Freezes (with or without vocalisations)
5	Difficult (runs around cage, is hard to grab, with or without vocalisations)
6	Very difficult (tail and throat rattles with or without vocalisations, may attack hand)
**HANDLING RAT IN HAND**	
1	Very easy (rat is totally limp)
2	Easy (alert, limbs may be pulled up against body)
3	Moderately easy (vocalisations, without resistance to being handled)
4	Freezes (rigid in hand, with or without vocalisations)
5	Difficult (squirming, twisting, attempting to bite, with or without vocalisations)

Table 12 - Detailed Clinical Examination Criteria (cont'd)

**LACRIMATION**	
1	None
2	Slight
3	Severe
**FUR APPEARANCE**	
1	Normal
2	Slightly soiled
3	Very soiled, crusty
Piloerection (Y/N)	
**SALIVATION**	
1	None
2	Slight
3	Severe
**DEFECATION**	
Number of faecal boluses on paper after 3 minutes. A **D** will be recorded if diarrhoea is present.	
**URINATION**	
Number of pools of urine on the paper after 3 minutes. An **X** will be recorded if polyuria is present.	
**GROOMING**	
Number of grooming episodes during a 3-minute period.<br>**S**: Repetitive grooming;<br>**U**: Unbalanced grooming	
**PALPEBRAL CLOSURE**	
1	Eyelids wide open
2	Eyelids slightly drooping
3	Ptosis - Drooping eyelids approximately half-way
4	Eyelids completely shut
**PUPIL RESPONSE**	
1	Normal response
2	Response is slightly reduced
3	Response is markedly reduced
4	Response is absent
**EYES**	
Exophthalmos (Y/N)	
Crustiness around eyes (Y/N)	
**RESPIRATION**	
1	Normal
2	Apnoea
3	Hyperventilation
**BEHAVIOUR**	
Writhing (Y/N)	
Circling (Y/N)	
Vocalisations (Y/N)	
**REARING**	
Number of rears in 3 minutes.<br>Unsupported rears;<br>Supported rears	

From IPCS (1986). Principles and Methods for the assessment of Neurotoxicity associated with Exposure to Chemicals. Environmental Health Criteria Document No 60.

Table 13 - Summarised Detailed Clinical Examinations: Main and Recovery Groups*

CRITERIA	OBSERVATIONS (n=10. Grs 1-3; n=5 Grs 4-5)				
	**Group 1** Vehicle control	**Group 2** 1 mg/kg TI/458	**Group 3** 20 mg/kg TI/458	**Group 4** Vehicle control	**Group 5** 20 mg/kg TI/458
Posture	NA	NA	NA	NA	NA
Gait	NA	NA	NA	NA	NA
Total Gait Score	NA	NA	NA	NA	NA
Clonic Convulsions	NA	NA	NA	NA	NA
Tonic Convulsions	NA	NA	NA	NA	NA
Biting	NA	NA	NA	NA	NA
Removing Rat from Cage	NA	NA	NA	NA	NA
Handling Rat in Hand	NA	NA	NA	NA	NA
Lacrimation	NA	NA	NA	NA	NA
Fur Appearance	NA	NA	NA	NA	NA
Salivation	NA	NA	NA	NA	NA
Defecation	NA	NA	NA	NA	NA
Urination	NA	NA	NA	NA	NA
Grooming	NA	NA	NA	NA	NA
Palpebral Closure	NA	NA	NA	NA	NA
Pupil Response	NA	NA	NA	NA	NA
Eyes	NA	NA	NA	NA	NA
Respiration	NA	NA	NA	NA	NA
Behaviour	NA	NA	NA	NA	NA
Rearing	NA	NA	NA	NA	NA

Note: *, Detailed clinical observations performed pre-treatment and each week for weeks 1-4; NA, no detailed clinical abnormalities.

Table 14 - Summarised Functional Observations*: Main and Recovery Groups

Stimulus	**OBSERVATIONS** (n=10 Grs 1-3; n=5 Grs 4-5)				
	**Group 1** Vehicle control	**Group 2** 1 mg/kg TI/458	**Group 3** 20 mg/kg TI/458	**Group 4** Vehicle control	**Group 5** 20 mg/kg TI/458
Arousal	NA	NA	NA	NA	NA
Finger snap	NA	NA	NA	NA	NA
Approach	NA	NA	NA	NA	NA
Touch	NA	NA	NA	NA	NA
Tail pinch	NA	NA	NA	NA	NA
Extensor rotation	NA	NA	NA	NA	NA
Catalepsy	NA	NA	NA	NA	NA
Righting reflex	NA	NA	NA	NA	NA
Supine drop	NA	NA	NA	NA	NA
Grip strength (mean) Forelimb	475 ± 32.9	487 ± 33.0	458 ± 39.9	441 ± 25.2	456 ± 47.2
Grip strength (mean) Hind limb	60 ± 7.9	59 ± 3.2	57 ± 2.5	60 ± 5.3	61 ± 3.8

Note: *, Functional observations performed on week 4 of treatment; NA, no functional abnormalities

Table 15 - Haematology Summary: Main Groups - Day 29

Parameter	Mean ± SD (n=10)		
	**Group 1** Vehicle control	**Group 2** 1 mg/kg TI/458	**Group 3** 20 mg/kg TI/458
**RBC** (x 10^{12} / L)	8.02 ± 0.318	7.90 ± 0.146	8.05 ± 0.199
**Hb** (g / L)	158 ± 5.0	157 ± 4.7	159 ± 3.0
**Hct** (L/L)	0.455 ± 0.0124	0.455 ± 0.0117	0.452 ± 0.0110
**MCV~** (fL)	56.8 ± 1.63	57.6 ± 1.41	56.2 ± 1.69
**MCH ~** (pg)	19.8 ± 0.48	19.9 ± 0.53	19.7 ± 0.51
**MCHC~** (g / L)	348 ± 5.7	344 ± 5.1	351 ± 4.0
**PLT** (x 10^9 / L)	1174 ± 164.7	1184 ± 127.7	1191 ± 93.8
**MPV** (fL)	8.1± 0.25	8.2 ± 0.41	8.1 ± 0.32
**WBC** (x 10^9 / L)	6.45 ± 2.012	7.04 ± 1.420	6.48 ± 1.752
**Neut** (x 10^9 / L)	0.91 ± 0.369	1.29 ± 0.510	1.01 ± 0.350
**Lymph** (x 10^9 / L)	5.35 ± 1.692	5.54 ± 0.937	5.26 ± 1.394
**Mono** (x 10^9 / L)	0.13 ± 0.064	0.16 ± 0.050	0.15 ± 0.049
**Eos** (x 10^9 / L)	0.05 ± 0.029	0.06 ± 0.030	0.06 ± 0.027
**Baso** (x 10^9 / L)	0.001 ± 0.0032	0.00 ± 0.003	0.00 ± 0.00

Notes: RBC, red blood cell count; **Hb**, haemoglobin; **Hct**, haematocrit; **MCV**, mean corpuscular volume; **MCH**, mean corpuscular haemoglobin; **MCHC**, mean corpuscular haemoglobin concentration; **PLT**, platelet count; **WBC**, white blood cell count; **Neut,** neutrophil count; **Lymph**, lymphocyte count; **Mono**, monocyte count; **Eos,** eosinophil count; **Baso,** basophil count; **SD**, standard deviation; **~**, calculated value.

Table 16 - Haematology Summary: Recovery Groups - Day 43

Parameter	Mean ± SD (n=5)	
	**Group 4** Vehicle control	**Group 5** 20 mg/kg TI/458
**RBC** (x 10^{12} / L)	8.19 ± 0.125	8.12 ± 0.224
**Hb** (g / L)	155 ± 5.2	154 ± 1.8
**Hct** (L/L)	0.0442 ± 0.0182	0.436 ± 0.0096
**MCV~** (fL)	54.0 ± 1.84	53.7 ± 2.02
**MCH ~** (pg)	18.9 ± 0.50	18.9 ± 0.62
**MCHC~** (g / L)	351 ± 3.7	353 ± 4.2
**PLT** (x 10^{9} / L)	1216 ± 59.0	1182 ± 143.7
**MPV** (fL)	8.0 ± 0.28	7.9 ± 0.21
**WBC** (x 10^{9} / L)	4.35 ± 1.745	5.92 ± 1.184
**Neut** (x 10^{9} / L)	0.78 ± 0.166	1.13 ± 0.675
**Lymph** (x 10^{9} / L)	3.42 ± 1.536	4.63 ± 1.160
**Mono** (x 10^{9} / L)	0.11 ± 0.073	0.12 ± 0.054
**Eos** (x 10^{9} / L)	0.04 ± 0.020	0.05 ± 0.027
**Baso** (x 10^{9} / L)	0	0

Notes: RBC, red blood cell count; **Hb**, haemoglobin; **Hct**, haematocrit; **MCV**, mean corpuscular volume; **MCH**, mean corpuscular haemoglobin; **MCHC**, mean corpuscular haemoglobin concentration; **PLT**, platelet count; **WBC**, white blood cell count; **Neut,** neutrophil count; **Lymph**, lymphocyte count; **Mono**, monocyte count; **Eos,** eosinophil count; **Baso,** basophil count; **SD**, standard deviation; **~**, calculated value.

Table 17 - Biochemistry Summary: Main Groups - Day 29

Parameter	Mean ± SD (n = 10)		
	**Group 1** Vehicle control	**Group 2** 1 mg/kg TI/458	**Group 3** 20 mg/kg TI/458
**ALT** (IU/L)	69.8 ± 21.83	61.1 ± 15.53	64.7 ± 19.01
**Ca** (mmol/L)	2.545 ± 0.072	2.53 ± 0.043	2.53 ± 0.075
**CK** (IU/L)	169 ± 91.4	164 ± 47.4	140 ± 34.4
**Cl** (mmol/L)	101 ± 1.1	101 ± 1.2	102 ± 1.2
**Glucose** (mmol/L)	9.81 ± 0.863	9.27 ± 0.963	10.14 ± 0.902
**TP** (g/L)	61.6 ± 1.78	61.2 ± 2.70	60.7 ± 1.95
**Creat** (mmol/L)	0.041 ± 0.0027	0.042 ± 0.0022	0.039 ± 0.0023
**Phos** (mmol/L)	2.32 ± 0.162	2.29 ± 0.232	2.20 ± 0.137
**Urea** (mmol/L)	5.57 ± 0.915	5.44 ± 0.772	4.98 ± 0.316
**Trig** (mmol/L)	0.647 ± 0.3391	0.392 ± 0.1322	0.538 ± 0.2772
**AST** (IU/L)	209 ± 63.0	173 ± 69.4	163 ± 76.8
**TBil** (µmol/L)	1.89 ± 0.373	1.91 ± 0.563	2.27 ± 0.327
**ALP** (IU/L)	236 ± 57.0	219 ± 37.4	234 ± 35.7
**Chol** (mmol/L)	2.15 ± 0.419	2.07 ± 0.433	1.97 ± 0.348
**Glob~** (g/L)	26.5 ± 1.61	26.9 ± 1.79	25.5 ± 1.91
**Na** (mmol/L)	145 ± 1.1	145 ± 0.7	145 ± 0.8
**ALB** (g/L)	35.1 ± 1.33	34.4 ± 1.99	35.2 ± 1.02
**GGT** (IU/L)	0.1 ± 0.32	0.5 ± 0.71	0.5 ± 0.53
**K** (mmol/L)	3.83 ± 0.177	3.59 ± 0.137	3.66 ± 0.295
**Ca:P ratio~**	1.1 ± 0.06	1.1 ± 0.10	1.2 ± 0.07
**Na:K ratio~**	37.9 ± 1.78	40.5 ± 1.59	39.9 ± 3.16
**A:G ratio~**	1.33 ± 0.099	1.28 ± 0.110	1.38 ± 0.117

Notes: Na, Sodium; **K**, potassium; **Cl**, Chloride; **Na:K**, sodium/potassium ratio; **Creat**, creatinine; **Ca**, calcium, **Phos,** phosphate; **Ca:P**, calcium/phosphate ratio; **TP**, total protein; **ALB**, albumin; **Glob**, globulin; **A:G ratio,** albumin/globulin ratio; **TBil**, total bilirubin; **ALP**, alkaline phosphatase; **AST**, aspartate aminotransferase; **ALT**, alanine aminotransferase; **CK,** creatine kinase; **GGT**, gamma glutamyl transferase; **Chol**, cholesterol; **Trig**, triglycerides; ~ calculated value; **SD**, standard deviation.

Table 18 - Biochemistry Summary: Recovery Groups - Day 43

Parameter	**Mean ± SD** (n = 5)	
	**Group 4** Vehicle control	**Group 5** 20 mg/kg TI/458
**ALT** (IU/L)	82 ± 31.7	62 ± 20.2
**Ca** (mmol/L)	2.60 ± 0.062	2.59 ± 0.029
**CK** (IU/L)	156 ± 30.3	117 ± 36.0
**Cl** (mmol/L)	101 ± 1.3	102 ± 1.9
**Glucose** (mmol/L)	9.1 ± 0.72	9.2 ± 0.37
**TP** (g/L)	61 ± 0.9	62 ± 1.7
**Creat** (mmol/L)	0.046 ± 0.0027	0.044 ± 0.0018
**Phos** (mmol/L)	2.34 ± 0.089	2.15 ± 0.129
**Urea** (mmol/L)	5.3 ± 0.36	5.3 ± 0.58
**Trig** (mmol/L)	0.79 ± 0.446	0.49 ± 0.123
**AST** (IU/L)	225 ± 116.8	236 ± 106.7
**TBil** (μmol/L)	2.6 ± 0.52	2.1 ± 0.81
**ALP** (IU/L)	138 ± 23.6	144 ± 24.2
**Chol** (mmol/L)	2.34 ± 0.526	2.35 ± 0.286
**Glob~** (g/L)	29.3 ± 0.99	31.0 ± 1.26
**Na** (mmol/L)	144 ± 1.2	144 ± 0.4
**ALB** (g/L)	31.3 ± 0.30	31.4 ± 1.29
**GGT** (IU/L)	0.8 ± 0.45	0.8 ± 0.45
**K** (mmol/L)	4.1 ± 0.26	3.8 ± 0.19
**Ca:P ratio~**	1.1 ± 0.04	1.2 ± 0.06
**Na:K ratio~**	35.4 ± 2.12	37.9 ± 1.92
**A:G ratio~**	1.1 ± 0.04	1.0 ± 0.06

Notes: Na, Sodium; **K**, potassium; **Cl**, Chloride; **Na:K**, sodium/potassium ratio; **Creat**, creatinine; **Ca**, calcium, **Phos,** phosphate; **Ca:P**, calcium/phosphate ratio; **TP**, total protein; **ALB**, albumin; **Glob**, globulin; **A:G ratio,** albumin/globulin ratio; **TBil**, total bilirubin; **ALP**, alkaline phosphatase; **AST**, aspartate aminotransferase; **ALT**, alanine aminotransferase; **CK,** creatine kinase; **GGT**, gamma glutamyl transferase; **Chol**, cholesterol; **Trig**, triglycerides; ~ calculated value; **SD**, standard deviation.

Table 19 - Urinalysis Summary: Main Groups - Day 29

Parameter	Mean ± SD		
	**Groups 1** Vehicle control n = 7	**Group 2** 1 mg/kg TI/458 n = 8	**Group 3** 20 mg/kg TI/458 n = 8
**Glucose** (Negative, 1+, 2+, 3+, 4+)	Negative - 7/7	Negative - 8/8	Negative - 9/9
**Bilirubin** (Negative, 1+, 2+, 3+)	Negative - 7/7	Negative - 8/8	Negative - 9/9
**Ketone** (Negative, trace, small, moderate, large)	Negative - 2/7<br>Trace - 3/7<br>Small - 2/7	Negative - 2/2<br>Trace – 5/8<br>Small – 1/8	Negative - 1/9<br>Trace – 4/9<br>Small – 4/9
**Specific Gravity**	1.020 ± 0.0096	1.012 ± 0.0065	1.009 ± 0.0055
**Blood** (Negative, trace, small, moderate, large)	Negative - 7/7	Negative - 8/8	Negative - 9/9
**pH**	6.6 ± 0.56	7.1 ± 0.48	7.2 ± 0.26
**Protein** (Negative, Trace, 1+, 2+, 3+, 4+)	Trace - 1/7<br>1+ - 2/7<br>2+ - 4/7	Trace - 2/8<br>1+ - 4/8<br>2+ - 2/8	1+ - 7/9<br>2+ - 2/9
**Urobilinogen** (Normal, 2, 4, 8)	Normal – 7/7	Normal – 8/8	Normal – 9/9
**Nitrite** (Negative, positive)	Negative - 7/7	Negative - 8/8	Negative - 9/9
**Leucocytes** (Negative, 1+, 2+, 3+)	Negative - 7/7	Negative - 8/8	Negative - 9/9

Notes: * Not all animals had urine in their bladder or there was insufficient sample to perform all tests (NSQ, not sufficient quantity); **Blood,** +, small; ++, moderate; +++, large; **Protein,** +, 0.3 g/L; ++ 1g/L; +++ 3 g/L; ++++, > 20 g/L.

Table 20 - Urinalysis Summary: Recovery Groups - Day 43

Parameter	Mean ± SD (n = 5 unless otherwise stated)	
	**Group 4** Vehicle control	**Group 5** 20 mg/kg TI/458
**Glucose** (Negative, 1+, 2+, 3+, 4+)	Negative - 5/5	Negative - 5/5
**Bilirubin** (Negative, 1+, 2+, 3+)	Negative - 5/5	Negative - 5/5
**Ketone** (Negative, trace, small, moderate, large)	Negative - 3/5<br>Small – 1/5<br>Trace – 1/5	Negative - 2/5<br>Trace – 3/5
**Specific Gravity**	1.014 ± 0.0065	1.014 ± 0.0108
**Blood** (Negative, trace, small, moderate, large)	Negative - 1/5<br>Small – 4/5	Negative - 3/5<br>Small – 1/5<br>Trace – 1/5
**pH**	7.1 ± 0.42	7.2 ± 0.45
**Protein** (Negative, 1+, 2+, 3+, 4+)	2+ - 4/5<br>1+ - 1/5	Negative - 2/5<br>1+ – 2/5<br>2+ – 1/5
**Urobilinogen** (Normal, 2, 4, 8)	Normal – 5/5	Normal – 5/5
**Nitrite** (Negative, positive)	Negative - 5/5	Negative - 5/5
**Leucocytes** (Negative, 1+, 2+, 3+)	Negative - 5/5	Negative - 5/5

Notes: * Not all animals had urine in their bladder or there was insufficient sample to perform all tests (NSQ, not sufficient quantity); **Blood,** +, small; ++, moderate; +++, large; **Protein,** +, 0.3 g/L; ++ 1g/L; +++ 3 g/L; ++++, > 20 g/L.

Table 21 - Organ Weights Summary: Main Groups - Day 29*

Parameter	Mean ± SD (g) n=10		
	**Group 1** Vehicle control	**Group 2** 1 mg/kg TI/458	**Group 3** 20 mg/kg TI/458
**Liver**	13.8 ± 1.85	13.2 ± 2.52	13.6 ± 1.68
**Kidneys**	3.052 ± 0.2528	2.955 ± 0.4075	3.018 ± 0.1928
**Adrenals**	00469 ± 0.00630	0.0454 ± 0.00783	0.0473 ± 0.00889
**Testes**	3.137 ± 0.1820	3.085 ± 0.2880	3.093 ± 0.2424
**Spleen**	0.754 ± 0.1148	0.8031 ± 0.0637	0.769 ± 0.1354
**Brain**	2.013 ± 0.0396	2.046 ± 0.0642	2.027 ± 0.0479
**Heart**	1.278 ± 0.1020	1.211 ± 0.1348	1.322 ± 0.105
**Thymus**	0.487 ± 0.0900	0.462 ± 0.0991	0.437 ± 0.0673
**Epididymis**	1.169 ± 0.1204	1.139 ± 0.0887	1.112 ± 0.0844
**Prostate + seminal vesicles with coagulating glands**	2.558 ± 0.1949	2.454 ± 0.2462	2.653 ± 0.2345

Notes: SD, standard deviation; *, weights measured after overnight fast.

,

Table 22 - Organ Weights Summary: Recovery Groups – Day 43*

Parameter	Mean ± SD (g) n=5	
	**Group 4** Vehicle control	**Group 5** 20 mg/kg TI/458
**Liver**	15.5 ± 3.86	14.5 ± 1.69
**Kidneys**	3.02 ± 0.319	3.03 ± 0.049
**Adrenals**	0.0476 ± 0.00475	0.0443 ± 0.00610
**Testes**	3.15 ± 0.236	3.01 ± 0.338
**Spleen**	0.774 ± 0.0357	0.826 ± 0.0843
**Brain**	2.04 ± 0.071	2.06 ± 0.061
**Heart**	1.40 ± 0.167	1.36 ± 0.015
**Thymus**	0.442 ± 0.0657	0.447 ± 0.0807
**Epididymis**	1.317 ± 0.0688	1.251 ± 0.0957
**Prostate + seminal vesicles with coagulating glands**	3.031 ± 0.1295	3.084 ± 0.1779

Notes: SD, standard deviation; *, weights measured after overnight fast.

Table 23 - Organ Weights (% Body Weight): Main Groups –Day 29#

Parameter	Mean ± SD (%) (n=10)		
	**Group 1** Vehicle control	**Group 2** 1 mg/kg TI/458	**Group 3** 20 mg/kg TI/458
**Adrenals**	3.44 ± 0.351	3.32 ± 0.411	3.44 ± 0.192
**Liver**	0.76 ± 0.047	0.75 ± 0.066	0.77 ± 0.042
**Kidneys**	0.012 ± 0.0018	0.012 ± 0.0020	0.012 ± 0.0027
**Testes**	0.78 ± 0.057	0.78 ± 0.073	0.79 ± 0.094
**Spleen**	0.18 ± 0.047	0.20 ± 0.018	0.19 ± 0.031
**Brain**	0.50 ± 0.027	0.52 ± 0.036	0.52 ± 0.041
**Heart**	0.32 ± 0.018	0.31 ± 0.026	0.34 ± 0.025
**Thymus**	0.12 ± 0.021	0.12 ± 0.027	0.11 ± 0.016
**Epididymis**	0.29 ± 0.027	0.29 ± 0.016	0.28 ± 0.036
**Prostate + seminal vesicles with coagulating glands**	0.64 ± 0.062	0.62 ± 0.061	0.67 ± 0.069

Notes: SD, standard deviation; #, body weights measured after fasting

Table 24 - Organ Weight (% Body Weight): Recovery Groups – Day 43#

**Parameter**	**Mean ± SD (%)** (n=5)	
	**Group 4** Vehicle control	**Group 5** 20 mg/kg TI/458
**Liver**	3.28 ± 0.448	3.15 ± 0.305
**Kidneys**	0.647 ± 0.0298	0.659 ± 0.0254
**Adrenals**	0.0102 ± 0.00101	0.0097 ± 0.00158
**Testes**	0.677 ± 0.0651	0.656 ± 0.0884
**Spleen**	0.167 ± 0.0227	0.180 ± 0.0190
**Brain**	0.440 ± 0.0377	0.449 ± 0.0221
**Heart**	0.300 ± 0.0109	0.297 ± 0.0108
**Thymus**	0.095 ± 0.0118	0.097 ± 0.0191
**Epididymis**	0.284 ± 0.0300	0.272 ± 0.0263
**Prostate + seminal vesicles with coagulating glands**	0.656 ± 0.0950	0.670 ± 0.0314

Notes: SD, standard deviation; #, body weights measured after fasting

Table 25 - Gross Necropsy Summary: Main Groups – Day 29

Organs	**Observations** (n=10)		
	**Group 1** Vehicle control	**Group 2** 1 mg/kg TI/458	**Group 3** 20 mg/kg TI/458
**Adrenals**	NA	NA	NA
**Liver**	NA	NA	NA
**Kidneys**	NA	NA	NA
**Testes**	NA	NA	NA
**Spleen**	NA	NA	NA
**Brain**	NA	NA	NA
**Heart**	NA	NA	NA
**Thymus**	NA	NA	NA
**Epididymis**	NA	NA	NA
**Prostate + seminal vesicles with coagulating glands**	NA	NA	NA
**Stomach**	NA	NA	NA
**Large intestine**	NA	NA	NA
**Small intestine**	NA	NA	NA
**Lung**	NA	NA	NA
**Eye**	NA	NA	NA
**Lymph nodes**	NA	NA	NA
**Peripheral nerve (sciatic)**	NA	NA	NA
**Spinal cord**	NA	NA	NA
**Thyroid**	NA	NA	NA
**Trachea**	NA	NA	NA
**Urinary bladder**	NA	NA	NA
**Bone marrow**	NA	NA	NA

Note: NA, no gross abnormalities attributed to treatment

Table 26 - Gross Necropsy Summary: Recovery Groups – Day 43

**Organs**	**Observations** (n=5)	
	**Group 4** Vehicle control	**Group 5** 20 mg/kg TI/458
**Adrenals**	NA	NA
**Liver**	NA	NA
**Kidneys**	NA	NA
**Testes**	NA	NA
**Spleen**	NA	NA
**Brain**	NA	NA
**Heart**	NA	NA
**Thymus**	NA	
**Epididymis**	NA	NA
**Prostate + seminal vesicles with coagulating glands**	NA	NA
**Stomach**	NA	NA
**Large intestine**	NA	NA
**Small intestine**	NA	NA
**Lung**	NA	NA
**Eye**	NA	NA
**Lymph nodes**	NA	NA
**Peripheral nerve (sciatic)**	NA	NA
**Spinal cord**	NA	NA
**Thyroid**	NA	NA
**Trachea**	NA	NA
**Urinary bladder**	NA	NA
**Bone marrow**	NA	NA

Note: NA, no gross abnormalities attributed to treatment

Table 27 - Histopathology Summary – Main Groups and Recovery

System	Individual Organs	Main groups		Recovery groups	
		**Group 1** Vehicle control	**Group 3** TI/458	**Group 4** Vehicle control	**Group 5** TI/458
**Alimentary and associated tissues**	**Stomach**	NA	NA	-	-
	**Small Intestine**	NA	NA		
	**Large intestine**	NA	NA	-	-
	**Liver**	NA	NA	-	-
**Cardio-vascular and respiratory**	**Heart**	NA	NA	-	-
	**Trachea**	NA	NA	-	-
	**Lung**	NA	NA	-	-
**Endocrine**	**Adrenal glands**	NA	NA	-	-
	**Thyroid gland**	NA	NA	-	-
**Genito-urinary**	**Kidney**	NA	NA	-	-
	**Epididymis**	NA	NA	-	-
	**Seminal vesicles**	NA	NA	-	-
	**Urinary bladder**	NA	NA	-	-
	**Prostate**	NA	NA	-	-
	**Testes**				
**Lymphoid and Haemato-poietic**	**Bone marrow**	NA	NA	-	-
	**Peyer's patch**	NA	NA	-	-
	**Spleen**	NA	NA	-	-
	**Thymus**	NA	NA	-	-
	**Lymph node (mesenteric)**	NA	NA	-	-
**Nervous and special senses**	**Brain stem**	NA	NA	-	-
	**Cerebrum**	NA	NA	-	-
	**Cerebellum**	NA	NA	-	-
	**Spinal cord**	NA	NA	-	-

Notes: NA, no abnormalities

The bright light of
certainty

I C P Firefly Pty Ltd

ACN 071 626 358

PO Box 6198, Alexandria NSW 2015 Australia
TEL: 61 2 9310 3899 FAX: 61 2 9310 4889 EMAIL: info@icpfirefly.com.au WEBSITE: www.icpfirefly.com.au

28-DAY REPEATED DOSE ORAL TOXICITY STUDY (OECD 407)
OF
c2
IN
SPRAGUE DAWLEY RATS WITH A RECOVERY PERIOD

ICPQN1035.B

CONTENTS OF APPENDICES

Appendix A: **Certificate of Analysis**

Appendix B: **Test Item Preparations**

Appendix C: **Study Data Spreadsheets**

Appendix D: **Haematology and Biochemistry**

Appendix E: **Histopathology**

Appendix F: **Protocol/Study Plan**

I C P Firefly Pty Ltd

ACN 071 626 358

PO Box 6198, Alexandria NSW 2015 Australia
TEL: 61 2 9310 3899 FAX: 61 2 9310 4889 EMAIL: info@icpfirefly.com.au WEBSITE: www.icpfirefly.com.au

Appendix A: Certificate of Analysis

1-2

BACHEM

Certificate of Analysis

**Lot number**	**1037619**
**Product**	**Cyclo(-2-Nal-Leu-Ser-2-Nal-Arg) acetate salt**
**Product number**	4070954
**Molecular formula (net)**	$C_{41}H_{50}N_8O_6$
**Relative molecular mass**	750.9
**Date of Manufacture**	September 30, 2011
**Date of Release**	November 02, 2011
**Date of Retest**	September 2012
**Specifications**	QS-4070954A/02
**Storage Conditions**	< -15 °C

**Tests**	**Specifications**	**Results**
Appearance	white to off-white powder	off-white powder
Appearance of solution	clear, colorless solution in 90% acetic acid, (1 mg/mL)	complies
Identification (ESI-MS)	m = 750.4 ± 1.0 u (monoisotopic mass)	m = 750.4 u
Identification (amino acid analysis)	determine and report	Ser* 0.8 (1) Leu 1.0 (1)<br>Arg° 0.7 (1) Nal not detected<br>* Ser partially destroyed during hydrolysis<br>° Nal-Arg probably incomplete hydrolysis
Purity (HPLC)	≥ 92.0%	97.5%
Related substances (HPLC)	report each individual ≥ 0.10%	RRT area%<br>0.50 0.33<br>0.61 0.16<br>1.27 1.50<br>1.56 0.22<br>1.57 0.12<br>1.68 0.18
Assay (elemental analysis)	determine and report as is (N_{th} = 14.92%)	86.7%
Water content (Karl Fischer)	determine and report	3.8%
Acetic acid content (IC)	determine and report	6.7%
Trifluoroacetic acid content (IC)	≤ 0.1%	< 0.015%
Residual organic solvents (GC)	≤ 410 mg/kg acetonitrile<br>≤ 5000 mg/kg isopropanol<br>≤ 3000 mg/kg methanol	< 10 mg/kg (LOD)<br>< 41 mg/kg (LOQ)<br>< 43 mg/kg (LOQ)
Mass balance	95.0 - 105.0% [sum of assay (elemental analysis), water content, and acetic acid content]	97.2%

Bachem AG
Hauptstrasse 144
4416 Bubendorf
Switzerland
Tel +41 61 935 2333
Fax +41 61 935 2325

BACHEM

Lot number	1037619
Product	Cyclo(-2-Nal-Leu-Ser-2-Nal-Arg) acetate salt

Tests	Specifications	Results
Bacterial endotoxins	≤ 10 IU/mg	< 10.0 IU/mg
Microbial limit test (Ph. Eur. 2.6.12)		
Total aerobic microbial count (TAMC)	≤ 10^2 CFU/100 mg	< 1 CFU/100 mg
Total yeasts and moulds count (TYMC)	≤ 10^1 CFU/100 mg	< 1 CFU/100 mg

I hereby certify that the above information is authentic and accurate. This batch of product has been manufactured, including packaging and quality control at Bachem AG in Bubendorf / Switzerland in full compliance with the GMP requirements of the local Regulatory Authority and according to the ICH Q7 Guideline for „APIs for use in Clinical Trials". The batch processing, packaging and analysis records were reviewed and found to be in compliance with GMP.

Date: November 03, 2011
Bachem AG

Dieter Arn, Ph.D.
QA Release Manager

This shipment has been dispensed and labeled according to the above mentioned GMP requirements. The dispensing record was reviewed and found to be in compliance with GMP.

Date: Nov 07, 2011
Bachem AG month day, year

Weighing Record Number: GM 11717

Signature: M. Kraemer

Name: M. Kraemer
Quality Assurance

Bachem AG
Hauptstrasse 144
4416 Bubendorf
Switzerland
Tel +41 61 935 2333
Fax +41 61 935 2325

I C P Firefly Pty Ltd

ACN 071 626 358

PO Box 6198, Alexandria NSW 2015 Australia
TEL: 61 2 9310 3899 FAX: 61 2 9310 4889 EMAIL: info@icpfirefly.com.au WEBSITE: www.icpfirefly.com.au

Appendix B: Test Item Preparations 1-9

I C P Firefly Pty Ltd

ACN 071 626 358

NATA

CERTIFIED QUALITY MANAGEMENT SYSTEM

TEST ITEM PREPARATION

**1. Study number** ICPQN1035.B	**2. Date** 10/1/12 – 31/1/12.	
**3. Test item** (incl. TI no.) Carboxymethyl cellulose. (CMC)	**4. Test item description** White powder.	
**5. Concentration of test item**	1% w/v.	
**6. Vehicle**	distilled water.	
**7. Procedure:** Include description of materials used, preparation of label, weight/vol equivalence duration of mixing or shaking)	① Weigh 4 g CMC into a 500 ml glass Schott bottle. ② Add 400 ml distilled water and place on magnetic stirrer until dissolved (overnight if necessary).	**Operator** (Sign & Date) [signature] 10/1/12
**8. Equipment used** (measuring devices etc)	TE005 balance.	
**9. Procedure checked by:**	[signature] **Date:** 10/1/12	
**10. Experimental procedure performed by:**	[signature] 10/1/12 [signature] 17/1/12 [signature] 24/1/12 [signature] 31/1/12. **Date:**	
**11. Notes** (Include information on test item vials used here)		

LAB-096/f3
issue 16.08.2007
(i/i)

QA Review ……[signature]…….. **Date** …05 MAR 2012…..

I C P Firefly Pty Ltd

ACN 071 626 358

NATA

NCS INTERNATIONAL CERTIFIED QUALITY MANAGEMENT SYSTEM ISO9001

TEST ITEM PREPARATION

**1. Study number** ICPQN1035.B		**2. Date** 11/01/12
**3. Test item** (incl. TI no.) TI/458 – c2		**4. Test item description** White powder
**5. Concentration of test item**	HD – 0.2% w/w (20mg/kg)<br>LD – 0.01% w/w (1mg/kg).	
**6. Vehicle**	1% CMC	
**7. Procedure:** Include description of materials used, preparation of label, weight/vol equivalence duration of mixing or shaking)	① Weigh 1.5mg test item into a glass vial + add vehicle to a total weight of 15g.<br>② Mix thoroughly to achieve a homogenous suspension (0.01% w/w).<br>③ Weigh 56mg test item into a glass beaker + add CMC to a total of 28g.<br>④ Mix thoroughly to achieve a homogenous suspension (0.2% w/w).	**Operator** (Sign & Date)<br>[signature] 11/01/12
**8. Equipment used** (measuring devices etc)	TE005 balance	
**9. Procedure checked by:**	[signature] **Date:** 11/1/12	
**10. Experimental procedure performed by:**	[signature] 14/1/12 [signature] 17/1/12<br>[signature] **Date:** 11/1/12.	
**11. Notes** (Include information on test item vials used here)	0.01%<br>1ml weighs: 0.9897, 0.9926, 0.9910<br>x̄ = 0.9911g.<br>10g/kg ≅ 10.09mL/kg	0.2%<br>1ml weighs: 0.9782, 0.9755, 0.9791<br>x̄ = 0.9776g<br>10g/kg ≅ 10.23ml/kg

LAB-096/f3
issue 16.08.2007
(i/i)

QA Review [signature] **Date** 05 MAR 2012

I C P Firefly Pty Ltd

ACN 071 626 358

NATA

CERTIFIED QUALITY MANAGEMENT SYSTEM ISO9001

TEST ITEM PREPARATION

**1. Study number** ICPQN1035.B	**2. Date** 12/01/12	
**3. Test item** (incl. TI no.) TI/458 – c2	**4. Test item description** White powder	
**5. Concentration of test item**	High dose – 0.2% w/w (20 mg/kg)<br>Low dose – 0.01% w/w (1 mg/kg)	
**6. Vehicle**	1% carboxymethylcellulose	
**7. Procedure:** Include description of materials used, preparation of label, weight/vol equivalence duration of mixing or shaking)	1. Weigh 1.5 mg test item into a glass vial and add vehicle to a total weight of 15 g.<br><br>2. Mix thoroughly to achieve a homogenous suspension (0.01%).<br><br>3. Weigh 30 mg test item into a glass vial and add vehicle to a total weight of 15 g.<br><br>4. Mix thoroughly to achieve a homogenous suspension (0.2%).	**Operator** (Sign & Date)
**8. Equipment used** (measuring devices etc)	TE005 balance	
**9. Procedure checked by:**	[signature] **Date:** 12/1/12	
**10. Experimental procedure performed by:**	[signature] 15/01/12 [signature] 18/1/12<br>[signature] **Date:** 12/01/12	
**11. Notes** (Include information on test item vials used here)		

QA Review ……[signature]…….. Date ……05 MAR 2012……

I C P Firefly Pty Ltd

ACN 071 626 358

NATA

NCS INTERNATIONAL CERTIFIED QUALITY MANAGEMENT SYSTEM ISO9001

TEST ITEM PREPARATION

1. **Study number** ICPQN1035.B	2. **Date** 20/1/12	
3. **Test item** (incl. TI no.) TI/458 – c2	4. **Test item description** White powder	
5. **Concentration of test item**	High dose – 0.2% w/w (20 mg/kg)<br>Low dose – 0.01% w/w (1 mg/kg)	
6. **Vehicle**	1% carboxymethylcellulose	
7. **Procedure:** Include description of materials used, preparation of label, weight/vol equivalence duration of mixing or shaking)	1. Weigh 1.7 mg test item into a glass vial and add vehicle to a total weight of 17 g.<br>2. Mix thoroughly to achieve a homogenous suspension (0.01%).<br>3. Weigh 66 mg test item into a glass vial and add vehicle to a total weight of 33 g.<br>4. Mix thoroughly to achieve a homogenous suspension (0.2%).	**Operator** (Sign & Date)<br>[signature] 20/1/12
8. **Equipment used** (measuring devices etc)	TE005 balance.	
9. **Procedure checked by:**	ER **Date:** 19/1/12	
10. **Experimental procedure performed by:**	[signature] 23/1/12 [signature] **Date:** 20/01/12	
11. **Notes** (Include information on test item vials used here)		

QA Review ...[initials]........... **Date** .....05 MAR 2012

I C P Firefly Pty Ltd

ACN 071 626 358

NATA

NCS INTERNATIONAL CERTIFIED QUALITY MANAGEMENT SYSTEM ISO9001

TEST ITEM PREPARATION

**1. Study number** ICPQN1035.B	**2. Date** 21/1/12	
**3. Test item** (incl. TI no.) TI/458 – c2	**4. Test item description** White powder	
**5. Concentration of test item**	High dose – 0.2% w/w (20 mg/kg)<br>Low dose – 0.01% w/w (1 mg/kg)	
**6. Vehicle**	1% carboxymethylcellulose	
**7. Procedure:** Include description of materials used, preparation of label, weight/vol equivalence duration of mixing or shaking)	1. Weigh 1.7 mg test item into a glass vial and add vehicle to a total weight of 17 g.<br>2. Mix thoroughly to achieve a homogenous suspension (0.01%).<br>3. Weigh 34 mg test item into a glass vial and add vehicle to a total weight of 17 g.<br>4. Mix thoroughly to achieve a homogenous suspension (0.2%).	**Operator** (Sign & Date)<br>[signature] 21/1/12
**8. Equipment used** (measuring devices etc)	TE005 balance	
**9. Procedure checked by:**	[signature] **Date:** 19/1/12	
**10. Experimental procedure performed by:**	[signature] 24/1/12<br>[signature] **Date:** 21/1/12	
**11. Notes** (Include information on test item vials used here)		

LAB-096/f3
issue 16.08.2007
(i/i)

QA Review ....[signature]........ **Date** ....05 MAR 2012....

B 5/9

NATA

CERTIFIED QUALITY MANAGEMENT SYSTEM

TEST ITEM PREPARATION

**1. Study number** ICPQN1035.B	**2. Date** 26/01/12	
**3. Test item** (incl. TI no.) TI/458 – c2	**4. Test item description** White powder	
**5. Concentration of test item**	High dose – 0.2% w/w (20 mg/kg)<br>Low dose – 0.01% w/w (1 mg/kg)	
**6. Vehicle**	1% carboxymethylcellulose	
**7. Procedure:** Include description of materials used, preparation of label, weight/vol equivalence duration of mixing or shaking)	1. Weigh 1.9 mg test item into a glass vial and add vehicle to a total weight of 19 g.<br>2. Mix thoroughly to achieve a homogenous suspension (0.01%).<br>3. Weigh 76 mg test item into a glass vial and add vehicle to a total weight of 38 g.<br>4. Mix thoroughly to achieve a homogenous suspension (0.2%).	**Operator** (Sign & Date)
**8. Equipment used** (measuring devices etc)	TE005 balance.	
**9. Procedure checked by:**	[signature] **Date:** 25/1/12	
**10. Experimental procedure performed by:**	[signature] 29/1/12 [signature] **Date:** 26/1/12	
**11. Notes** (Include information on test item vials used here)		

QA Review [signature] Date 05 MAR 2012

I C P Firefly Pty Ltd

ACN 071 626 358

NATA

CERTIFIED QUALITY MANAGEMENT SYSTEM ISO9001

TEST ITEM PREPARATION

**1. Study number** ICPQN1035.B	**2. Date** 27/01/12	
**3. Test item** (incl. TI no.) TI/458 – c2	**4. Test item description** White powder	
**5. Concentration of test item**	High dose – 0.2% w/w (20 mg/kg)<br>Low dose – 0.01% w/w (1 mg/kg)	
**6. Vehicle**	1% carboxymethylcellulose	
**7. Procedure:** Include description of materials used, preparation of label, weight/vol equivalence duration of mixing or shaking)	1. Weigh 1.9 mg test item into a glass vial and add vehicle to a total weight of 19 g.<br>2. Mix thoroughly to achieve a homogenous suspension (0.01%).<br>3. Weigh 38 mg test item into a glass vial and add vehicle to a total weight of 19 g.<br>4. Mix thoroughly to achieve a homogenous suspension (0.2%).	**Operator** (Sign & Date)
**8. Equipment used** (measuring devices etc)		
**9. Procedure checked by:**	[signature] **Date:** 28/1/12	
**10. Experimental procedure performed by:**	[signature] 30/1/12 [signature] **Date:** 27/1/12	
**11. Notes** (Include information on test item vials used here)		

QA Review ....[signature]........ **Date** ...05 MAR 2012...

B 7/9

I C P Firefly Pty Ltd

ACN 071 626 358

NATA

CERTIFIED QUALITY MANAGEMENT SYSTEM

TEST ITEM PREPARATION

**1. Study number** ICPQN1035.B	**2. Date** 1/2/12	
**3. Test item** (incl. TI no.) TI/458 – c2	**4. Test item description** White powder	
**5. Concentration of test item**	High dose – 0.2% w/w (20 mg/kg)<br>Low dose – 0.01% w/w (1 mg/kg)	
**6. Vehicle**	1% carboxymethylcellulose	
**7. Procedure:** Include description of materials used, preparation of label, weight/vol equivalence duration of mixing or shaking)	1. Weigh 2.3 mg test item into a glass vial and add vehicle to a total weight of 23 g.<br>2. Mix thoroughly to achieve a homogenous suspension (0.01%).<br>3. Weigh 90 mg test item into a glass vial and add vehicle to a total weight of 45 g.<br>4. Mix thoroughly to achieve a homogenous suspension (0.2%).	**Operator** (Sign & Date)
**8. Equipment used** (measuring devices etc)	TE005 balance.	
**9. Procedure checked by:**	[illegible] **Date:** 1/2/12	
**10. Experimental procedure performed by:**	[illegible] 4/2/12<br>[illegible] 7/2/12<br>[illegible] **Date:** 1/2/12	
**11. Notes** (Include information on test item vials used here)		

LAB-096/f3
issue 16.08.2007
(i/i)

QA Review ......[illegible]...... **Date** ...05 MAR 2012...

B 8/9

I C P Firefly Pty Ltd

ACN 071 626 358

TEST ITEM PREPARATION

**1. Study number** ICPQN1035.B	**2. Date** 2/2/12	
**3. Test item** (incl. TI no.) TI/458 – c2	**4. Test item description** White powder	
**5. Concentration of test item**	High dose – 0.2% w/w (20 mg/kg)<br>Low dose – 0.01% w/w (1 mg/kg)	
**6. Vehicle**	1% carboxymethylcellulose	
**7. Procedure:** Include description of materials used, preparation of label, weight/vol equivalence duration of mixing or shaking)	1. Weigh 2.3 mg test item into a glass vial and add vehicle to a total weight of 23 g.<br>2. Mix thoroughly to achieve a homogenous suspension (0.01%).<br>3. Weigh 46 mg test item into a glass vial and add vehicle to a total weight of 23 g.<br>4. Mix thoroughly to achieve a homogenous suspension (0.2%).	**Operator** (Sign & Date)
**8. Equipment used** (measuring devices etc)	TE005 balance.	
**9. Procedure checked by:**	E2 Date: 1/2/12	
**10. Experimental procedure performed by:**	d5/2/12<br>d8/2/12<br>d Date: 2/2/12	
**11. Notes** (Include information on test item vials used here)		

QA Review [illegible] Date 05 MAR 2012

The bright light of
certainty

I C P Firefly Pty Ltd

ACN 071 626 358

PO Box 6198, Alexandria NSW 2015 Australia
TEL: 61 2 9310 3899 FAX: 61 2 9310 4889 EMAIL: info@icpfirefly.com.au WEBSITE: www.icpfirefly.com.au

Appendix C: Study Data Spreadsheets 1-45

ICPQN1035.B

LAB-028/f1 & LAB-028/F2 INDIVIDUAL CLINICAL OBSERVATIONS - DAILY	GROUP	1	1	1	1	1	1	1	1	1	1
	Treatment	Vehicle	Vehicle	Vehicle	Vehicle	Vehicle	Vehicle	Vehicle	Vehicle	Vehicle	Vehicle
	Dose (mg/kg)	-	-	-	-	-	-	-	-	-	-
Note: -, No abnormalities; P- porphyrin discharge around eye	Animal ID	R2409	R2410	R2411	R2412	R2413	R2414	R2415	R2416	R2417	R2418
	Sex	M	M	M	M	M	M	M	M	M	M
**DAY 1**											
general appearance		-	-	-	-	-	-	-	-	-	-
skin and fur		-	-	-	-	-	-	-	-	-	-
eyes/mucous membranes		-	-	-	-	-	-	-	-	-	-
respiratory system		-	-	-	-	-	-	-	-	-	-
somatomotor activity		-	-	-	-	-	-	-	-	-	-
behaviour pattern		-	-	-	-	-	-	-	-	-	-
tremors and convulsions		-	-	-	-	-	-	-	-	-	-
salivation		-	-	-	-	-	-	-	-	-	-
diarrhoea		-	-	-	-	-	-	-	-	-	-
lethargy		-	-	-	-	-	-	-	-	-	-
sleep or coma		-	-	-	-	-	-	-	-	-	-
other(specify)		-	-	-	-	-	-	-	-	-	-
**DAY 2**											
general appearance		-	-	-	-	-	-	-	-	-	-
skin and fur		-	-	-	-	-	-	-	-	-	-
eyes/mucous membranes		-	-	-	-	-	-	-	-	-	-
respiratory system		-	-	-	-	-	-	-	-	-	-
somatomotor activity		-	-	-	-	-	-	-	-	-	-
behaviour pattern		-	-	-	-	-	-	-	-	-	-
tremors and convulsions		-	-	-	-	-	-	-	-	-	-
salivation		-	-	-	-	-	-	-	-	-	-
diarrhoea		-	-	-	-	-	-	-	-	-	-
lethargy		-	-	-	-	-	-	-	-	-	-
sleep or coma		-	-	-	-	-	-	-	-	-	-
other(specify)		-	-	-	-	-	-	-	-	-	-
**DAY 3**											
general appearance		-	-	-	-	-	-	-	-	-	-
skin and fur		-	-	-	-	-	-	-	-	-	-
eyes/mucous membranes		-	-	-	-	-	-	-	-	-	-
respiratory system		-	-	-	-	-	-	-	-	-	-
somatomotor activity		-	-	-	-	-	-	-	-	-	-
behaviour pattern		-	-	-	-	-	-	-	-	-	-
tremors and convulsions		-	-	-	-	-	-	-	-	-	-
salivation		-	-	-	-	-	-	-	-	-	-
diarrhoea		-	-	-	-	-	-	-	-	-	-
lethargy		-	-	-	-	-	-	-	-	-	-
sleep or coma		-	-	-	-	-	-	-	-	-	-
other(specify)		-	-	-	-	-	-	-	-	-	-
**DAY 4**											
general appearance		-	-	-	-	-	-	-	-	-	-
skin and fur		-	-	-	-	-	-	-	-	-	-
eyes/mucous membranes		-	-	-	-	-	-	-	-	-	-
respiratory system		-	-	-	-	-	-	-	-	-	-
somatomotor activity		-	-	-	-	-	-	-	-	-	-
behaviour pattern		-	-	-	-	-	-	-	-	-	-
tremors and convulsions		-	-	-	-	-	-	-	-	-	-
salivation		-	-	-	-	-	-	-	-	-	-
diarrhoea		-	-	-	-	-	-	-	-	-	-
lethargy		-	-	-	-	-	-	-	-	-	-
sleep or coma		-	-	-	-	-	-	-	-	-	-
other(specify)		-	-	-	-	-	-	-	-	-	-
**DAY 5**											
general appearance		-	-	-	-	-	-	-	-	-	-
skin and fur		-	-	-	-	-	-	-	-	-	-
eyes/mucous membranes		-	-	-	-	-	-	-	-	-	-
respiratory system		-	-	-	-	-	-	-	-	-	-
somatomotor activity		-	-	-	-	-	-	-	-	-	-
behaviour pattern		-	-	-	-	-	-	-	-	-	-
tremors and convulsions		-	-	-	-	-	-	-	-	-	-
salivation		-	-	-	-	-	-	-	-	-	-
diarrhoea		-	-	-	-	-	-	-	-	-	-
lethargy		-	-	-	-	-	-	-	-	-	-
sleep or coma		-	-	-	-	-	-	-	-	-	-
other(specify)		-	-	-	-	-	-	-	-	-	-
**DAY 6**											
general appearance		-	-	-	-	-	-	-	-	-	-
skin and fur		-	-	-	-	-	-	-	-	-	-
eyes/mucous membranes		-	-	-	-	-	-	-	-	-	-
respiratory system		-	-	-	-	-	-	-	-	-	-
somatomotor activity		-	-	-	-	-	-	-	-	-	-
behaviour pattern		-	-	-	-	-	-	-	-	-	-
tremors and convulsions		-	-	-	-	-	-	-	-	-	-
salivation		-	-	-	-	-	-	-	-	-	-
diarrhoea		-	-	-	-	-	-	-	-	-	-
lethargy		-	-	-	-	-	-	-	-	-	-
sleep or coma		-	-	-	-	-	-	-	-	-	-
other(specify)		-	-	-	-	-	-	-	-	-	-

ENTERED BY: ER 10/2/12

CHECKED BY: 20/2/12

ICPQN1035.B

LAB-028/f1 & LAB-028/F2 INDIVIDUAL CLINICAL OBSERVATIONS - DAILY	GROUP	1	1	1	1	1	1	1	1	1	1
	Treatment	Vehicle	Vehicle	Vehicle	Vehicle	Vehicle	Vehicle	Vehicle	Vehicle	Vehicle	Vehicle
	Dose (mg/kg)	-	-	-	-	-	-	-	-	-	-
Note: -, No abnormalities;	Animal ID	R2409	R2410	R2411	R2412	R2413	R2414	R2415	R2416	R2417	R2418
P- porphyrin discharge around eye	Sex	M	M	M	M	M	M	M	M	M	M
**DAY 7**											
general appearance		-	-	-	-	-	-	-	-	-	-
skin and fur		-	-	-	-	-	-	-	-	-	-
eyes/mucous membranes		-	-	-	-	-	-	-	-	-	-
respiratory system		-	-	-	-	-	-	-	-	-	-
somatomotor activity		-	-	-	-	-	-	-	-	-	-
behaviour pattern		-	-	-	-	-	-	-	-	-	-
tremors and convulsions		-	-	-	-	-	-	-	-	-	-
salivation		-	-	-	-	-	-	-	-	-	-
diarrhoea		-	-	-	-	-	-	-	-	-	-
lethargy		-	-	-	-	-	-	-	-	-	-
sleep or coma		-	-	-	-	-	-	-	-	-	-
other(specify)		-	-	-	-	-	-	-	-	-	-
**DAY 8**											
general appearance		-	-	-	-	-	-	-	-	-	-
skin and fur		-	-	-	-	-	-	-	-	-	-
eyes/mucous membranes		-	-	-	-	-	-	-	-	-	-
respiratory system		-	-	-	-	-	-	-	-	-	-
somatomotor activity		-	-	-	-	-	-	-	-	-	-
behaviour pattern		-	-	-	-	-	-	-	-	-	-
tremors and convulsions		-	-	-	-	-	-	-	-	-	-
salivation		-	-	-	-	-	-	-	-	-	-
diarrhoea		-	-	-	-	-	-	-	-	-	-
lethargy		-	-	-	-	-	-	-	-	-	-
sleep or coma		-	-	-	-	-	-	-	-	-	-
other(specify)		-	-	-	-	-	-	-	-	-	-
**DAY 9**											
general appearance		-	-	-	-	-	-	-	-	-	-
skin and fur		-	-	-	-	-	-	-	-	-	-
eyes/mucous membranes		-	-	-	-	-	-	-	-	-	-
respiratory system		-	-	-	-	-	-	-	-	-	-
somatomotor activity		-	-	-	-	-	-	-	-	-	-
behaviour pattern		-	-	-	-	-	-	-	-	-	-
tremors and convulsions		-	-	-	-	-	-	-	-	-	-
salivation		-	-	-	-	-	-	-	-	-	-
diarrhoea		-	-	-	-	-	-	-	-	-	-
lethargy		-	-	-	-	-	-	-	-	-	-
sleep or coma		-	-	-	-	-	-	-	-	-	-
other(specify)		-	-	-	-	-	-	-	-	-	-
**DAY 10**											
general appearance		-	-	-	-	-	-	-	-	-	-
skin and fur		-	-	-	-	-	-	-	-	-	-
eyes/mucous membranes		-	-	-	-	-	-	-	-	-	-
respiratory system		-	-	-	-	-	-	-	-	-	-
somatomotor activity		-	-	-	-	-	-	-	-	-	-
behaviour pattern		-	-	-	-	-	-	-	-	-	-
tremors and convulsions		-	-	-	-	-	-	-	-	-	-
salivation		-	-	-	-	-	-	-	-	-	-
diarrhoea		-	-	-	-	-	-	-	-	-	-
lethargy		-	-	-	-	-	-	-	-	-	-
sleep or coma		-	-	-	-	-	-	-	-	-	-
other(specify)		-	-	-	-	-	-	-	-	-	-
**DAY 11**											
general appearance		-	-	-	-	-	-	-	-	-	-
skin and fur		-	-	-	-	-	-	-	-	-	-
eyes/mucous membranes		-	-	-	-	-	-	-	-	-	-
respiratory system		-	-	-	-	-	-	-	-	-	-
somatomotor activity		-	-	-	-	-	-	-	-	-	-
behaviour pattern		-	-	-	-	-	-	-	-	-	-
tremors and convulsions		-	-	-	-	-	-	-	-	-	-
salivation		-	-	-	-	-	-	-	-	-	-
diarrhoea		-	-	-	-	-	-	-	-	-	-
lethargy		-	-	-	-	-	-	-	-	-	-
sleep or coma		-	-	-	-	-	-	-	-	-	-
other(specify)		-	-	-	-	-	-	-	-	-	-
**DAY 12**											
general appearance		-	-	-	-	-	-	-	-	-	-
skin and fur		-	-	-	-	-	-	-	-	-	-
eyes/mucous membranes		-	-	-	-	-	-	-	-	-	-
respiratory system		-	-	-	-	-	-	-	-	-	-
somatomotor activity		-	-	-	-	-	-	-	-	-	-
behaviour pattern		-	-	-	-	-	-	-	-	-	-
tremors and convulsions		-	-	-	-	-	-	-	-	-	-
salivation		-	-	-	-	-	-	-	-	-	-
diarrhoea		-	-	-	-	-	-	-	-	-	-
lethargy		-	-	-	-	-	-	-	-	-	-
sleep or coma		-	-	-	-	-	-	-	-	-	-
other(specify)		-	-	-	-	-	-	-	-	-	-

ENTERED BY: [illegible] 10/2/12

CHECKED BY: [illegible] 20/2/12

ICPQN1035.B

**LAB-028/f1 & LAB-028/F2 INDIVIDUAL CLINICAL OBSERVATIONS - DAILY**	GROUP	1	1	1	1	1	1	1	1	1	1
	Treatment	Vehicle	Vehicle	Vehicle	Vehicle	Vehicle	Vehicle	Vehicle	Vehicle	Vehicle	Vehicle
	Dose (mg/kg)	-	-	-	-	-	-	-	-	-	-
**Note:** -, No abnormalities;	Animal ID	R2409	R2410	R2411	R2412	R2413	R2414	R2415	R2416	R2417	R2418
P- porphyrin discharge around eye	Sex	M	M	M	M	M	M	M	M	M	M
**DAY 13**											
general appearance		-	-	-	-	-	-	-	-	-	-
skin and fur		-	-	-	-	-	-	-	-	-	-
eyes/mucous membranes		-	-	-	-	-	-	-	-	-	-
respiratory system		-	-	-	-	-	-	-	-	-	-
somatomotor activity		-	-	-	-	-	-	-	-	-	-
behaviour pattern		-	-	-	-	-	-	-	-	-	-
tremors and convulsions		-	-	-	-	-	-	-	-	-	-
salivation		-	-	-	-	-	-	-	-	-	-
diarrhoea		-	-	-	-	-	-	-	-	-	-
lethargy		-	-	-	-	-	-	-	-	-	-
sleep or coma		-	-	-	-	-	-	-	-	-	-
other(specify)		-	-	-	-	-	-	-	-	-	-
**DAY 14**											
general appearance		-	-	-	-	-	-	-	-	-	-
skin and fur		-	-	-	-	-	-	-	-	-	-
eyes/mucous membranes		-	-	-	-	-	-	-	-	-	-
respiratory system		-	-	-	-	-	-	-	-	-	-
somatomotor activity		-	-	-	-	-	-	-	-	-	-
behaviour pattern		-	-	-	-	-	-	-	-	-	-
tremors and convulsions		-	-	-	-	-	-	-	-	-	-
salivation		-	-	-	-	-	-	-	-	-	-
diarrhoea		-	-	-	-	-	-	-	-	-	-
lethargy		-	-	-	-	-	-	-	-	-	-
sleep or coma		-	-	-	-	-	-	-	-	-	-
other(specify)		-	-	-	-	-	-	-	-	-	-
**DAY 15**											
general appearance		-	-	-	-	-	-	-	-	-	-
skin and fur		-	-	-	-	-	-	-	-	-	-
eyes/mucous membranes		-	-	-	-	-	-	-	-	-	-
respiratory system		-	-	-	-	-	-	-	-	-	-
somatomotor activity		-	-	-	-	-	-	-	-	-	-
behaviour pattern		-	-	-	-	-	-	-	-	-	-
tremors and convulsions		-	-	-	-	-	-	-	-	-	-
salivation		-	-	-	-	-	-	-	-	-	-
diarrhoea		-	-	-	-	-	-	-	-	-	-
lethargy		-	-	-	-	-	-	-	-	-	-
sleep or coma		-	-	-	-	-	-	-	-	-	-
other(specify)		-	-	-	-	-	-	-	-	-	-
**DAY 16**											
general appearance		-	-	-	-	-	-	-	-	-	-
skin and fur		-	-	-	-	-	-	-	-	-	-
eyes/mucous membranes		-	-	-	-	-	-	-	-	-	-
respiratory system		-	-	-	-	-	-	-	-	-	-
somatomotor activity		-	-	-	-	-	-	-	-	-	-
behaviour pattern		-	-	-	-	-	-	-	-	-	-
tremors and convulsions		-	-	-	-	-	-	-	-	-	-
salivation		-	-	-	-	-	-	-	-	-	-
diarrhoea		-	-	-	-	-	-	-	-	-	-
lethargy		-	-	-	-	-	-	-	-	-	-
sleep or coma		-	-	-	-	-	-	-	-	-	-
other(specify)		-	-	-	-	-	-	-	-	-	-
**DAY 17**											
general appearance		-	-	-	-	-	-	-	-	-	-
skin and fur		-	-	-	-	-	-	-	-	-	-
eyes/mucous membranes		-	-	-	-	-	-	-	-	-	-
respiratory system		-	-	-	-	-	-	-	-	-	-
somatomotor activity		-	-	-	-	-	-	-	-	-	-
behaviour pattern		-	-	-	-	-	-	-	-	-	-
tremors and convulsions		-	-	-	-	-	-	-	-	-	-
salivation		-	-	-	-	-	-	-	-	-	-
diarrhoea		-	-	-	-	-	-	-	-	-	-
lethargy		-	-	-	-	-	-	-	-	-	-
sleep or coma		-	-	-	-	-	-	-	-	-	-
other(specify)		-	-	-	-	-	-	-	-	-	-
**DAY 18**											
general appearance		-	-	-	-	-	-	-	-	-	-
skin and fur		-	-	-	-	-	-	-	-	-	-
eyes/mucous membranes		-	-	-	-	-	-	-	-	-	-
respiratory system		-	-	-	-	-	-	-	-	-	-
somatomotor activity		-	-	-	-	-	-	-	-	-	-
behaviour pattern		-	-	-	-	-	-	-	-	-	-
tremors and convulsions		-	-	-	-	-	-	-	-	-	-
salivation		-	-	-	-	-	-	-	-	-	-
diarrhoea		-	-	-	-	-	-	-	-	-	-
lethargy		-	-	-	-	-	-	-	-	-	-
sleep or coma		-	-	-	-	-	-	-	-	-	-
other(specify)		-	-	-	-	-	-	-	-	-	-

ENTERED BY: 82 10/2/12
CHECKED BY: d 20/2/12

ICPQN1035.B

LAB-028/f1 & LAB-028/F2 INDIVIDUAL CLINICAL OBSERVATIONS - DAILY	GROUP	1	1	1	1	1	1	1	1	1	1
	Treatment	Vehicle	Vehicle	Vehicle	Vehicle	Vehicle	Vehicle	Vehicle	Vehicle	Vehicle	Vehicle
	Dose (mg/kg)	-	-	-	-	-	-	-	-	-	-
**Note:** -, No abnormalities;	Animal ID	R2409	R2410	R2411	R2412	R2413	R2414	R2415	R2416	R2417	R2418
P- porphyrin discharge around eye	Sex	M	M	M	M	M	M	M	M	M	M
**DAY 19**											
general appearance		-	-	-	-	-	-	-	-	-	-
skin and fur		-	-	-	-	-	-	-	-	-	-
eyes/mucous membranes		-	-	-	-	-	-	-	-	-	-
respiratory system		-	-	-	-	-	-	-	-	-	-
somatomotor activity		-	-	-	-	-	-	-	-	-	-
behaviour pattern		-	-	-	-	-	-	-	-	-	-
tremors and convulsions		-	-	-	-	-	-	-	-	-	-
salivation		-	-	-	-	-	-	-	-	-	-
diarrhoea		-	-	-	-	-	-	-	-	-	-
lethargy		-	-	-	-	-	-	-	-	-	-
sleep or coma		-	-	-	-	-	-	-	-	-	-
other(specify)		-	-	-	-	-	-	-	-	-	-
**DAY 20**											
general appearance		-	-	-	-	-	-	-	-	-	-
skin and fur		-	-	-	-	-	-	-	-	-	-
eyes/mucous membranes		-	-	-	-	-	-	-	-	-	-
respiratory system		-	-	-	-	-	-	-	-	-	-
somatomotor activity		-	-	-	-	-	-	-	-	-	-
behaviour pattern		-	-	-	-	-	-	-	-	-	-
tremors and convulsions		-	-	-	-	-	-	-	-	-	-
salivation		-	-	-	-	-	-	-	-	-	-
diarrhoea		-	-	-	-	-	-	-	-	-	-
lethargy		-	-	-	-	-	-	-	-	-	-
sleep or coma		-	-	-	-	-	-	-	-	-	-
other(specify)		-	-	-	-	-	-	-	-	-	-
**DAY 21**											
general appearance		-	-	-	-	-	-	-	-	-	-
skin and fur		-	-	-	-	-	-	-	-	-	-
eyes/mucous membranes		-	P	-	-	-	-	-	-	-	-
respiratory system		-	-	-	-	-	-	-	-	-	-
somatomotor activity		-	-	-	-	-	-	-	-	-	-
behaviour pattern		-	-	-	-	-	-	-	-	-	-
tremors and convulsions		-	-	-	-	-	-	-	-	-	-
salivation		-	-	-	-	-	-	-	-	-	-
diarrhoea		-	-	-	-	-	-	-	-	-	-
lethargy		-	-	-	-	-	-	-	-	-	-
sleep or coma		-	-	-	-	-	-	-	-	-	-
other(specify)		-	-	-	-	-	-	-	-	-	-
**DAY 22**											
general appearance		-	-	-	-	-	-	-	-	-	-
skin and fur		-	-	-	-	-	-	-	-	-	-
eyes/mucous membranes		-	P	-	-	-	-	-	-	-	-
respiratory system		-	-	-	-	-	-	-	-	-	-
somatomotor activity		-	-	-	-	-	-	-	-	-	-
behaviour pattern		-	-	-	-	-	-	-	-	-	-
tremors and convulsions		-	-	-	-	-	-	-	-	-	-
salivation		-	-	-	-	-	-	-	-	-	-
diarrhoea		-	-	-	-	-	-	-	-	-	-
lethargy		-	-	-	-	-	-	-	-	-	-
sleep or coma		-	-	-	-	-	-	-	-	-	-
other(specify)		-	-	-	-	-	-	-	-	-	-
**DAY 23**											
general appearance		-	-	-	-	-	-	-	-	-	-
skin and fur		-	-	-	-	-	-	-	-	-	-
eyes/mucous membranes		-	-	-	-	-	-	-	-	-	-
respiratory system		-	-	-	-	-	-	-	-	-	-
somatomotor activity		-	-	-	-	-	-	-	-	-	-
behaviour pattern		-	-	-	-	-	-	-	-	-	-
tremors and convulsions		-	-	-	-	-	-	-	-	-	-
salivation		-	-	-	-	-	-	-	-	-	-
diarrhoea		-	-	-	-	-	-	-	-	-	-
lethargy		-	-	-	-	-	-	-	-	-	-
sleep or coma		-	-	-	-	-	-	-	-	-	-
other(specify)		-	-	-	-	-	-	-	-	-	-
**DAY 24**											
general appearance		-	-	-	-	-	-	-	-	-	-
skin and fur		-	-	-	-	-	-	-	-	-	-
eyes/mucous membranes		-	-	-	-	-	-	-	-	-	-
respiratory system		-	-	-	-	-	-	-	-	-	-
somatomotor activity		-	-	-	-	-	-	-	-	-	-
behaviour pattern		-	-	-	-	-	-	-	-	-	-
tremors and convulsions		-	-	-	-	-	-	-	-	-	-
salivation		-	-	-	-	-	-	-	-	-	-
diarrhoea		-	-	-	-	-	-	-	-	-	-
lethargy		-	-	-	-	-	-	-	-	-	-
sleep or coma		-	-	-	-	-	-	-	-	-	-
other(specify)		-	-	-	-	-	-	-	-	-	-

ENTERED BY: SQ 10/2/12

CHECKED BY: [illegible] 20/2/12

ICPQN1035.B

**LAB-028/f1 & LAB-028/F2 INDIVIDUAL CLINICAL OBSERVATIONS - DAILY**	GROUP	1	1	1	1	1	1	1	1	1	1
	Treatment	Vehicle	Vehicle	Vehicle	Vehicle	Vehicle	Vehicle	Vehicle	Vehicle	Vehicle	Vehicle
	Dose (mg/kg)	-	-	-	-	-	-	-	-	-	-
**Note:** -, No abnormalities;	Animal ID	R2409	R2410	R2411	R2412	R2413	R2414	R2415	R2416	R2417	R2418
P- porphyrin discharge around eye	Sex	M	M	M	M	M	M	M	M	M	M
**DAY 25**											
general appearance		-	-	-	-	-	-	-	-	-	-
skin and fur		-	-	-	-	-	-	-	-	-	-
eyes/mucous membranes		-	-	-	-	-	-	-	-	-	-
respiratory system		-	-	-	-	-	-	-	-	-	-
somatomotor activity		-	-	-	-	-	-	-	-	-	-
behaviour pattern		-	-	-	-	-	-	-	-	-	-
tremors and convulsions		-	-	-	-	-	-	-	-	-	-
salivation		-	-	-	-	-	-	-	-	-	-
diarrhoea		-	-	-	-	-	-	-	-	-	-
lethargy		-	-	-	-	-	-	-	-	-	-
sleep or coma		-	-	-	-	-	-	-	-	-	-
other(specify)		-	-	-	-	-	-	-	-	-	-
**DAY 26**											
general appearance		-	-	-	-	-	-	-	-	-	-
skin and fur		-	-	-	-	-	-	-	-	-	-
eyes/mucous membranes		-	-	-	-	-	-	-	-	-	-
respiratory system		-	-	-	-	-	-	-	-	-	-
somatomotor activity		-	-	-	-	-	-	-	-	-	-
behaviour pattern		-	-	-	-	-	-	-	-	-	-
tremors and convulsions		-	-	-	-	-	-	-	-	-	-
salivation		-	-	-	-	-	-	-	-	-	-
diarrhoea		-	-	-	-	-	-	-	-	-	-
lethargy		-	-	-	-	-	-	-	-	-	-
sleep or coma		-	-	-	-	-	-	-	-	-	-
other(specify)		-	-	-	-	-	-	-	-	-	-
**DAY 27**											
general appearance		-	-	-	-	-	-	-	-	-	-
skin and fur		-	-	-	-	-	-	-	-	-	-
eyes/mucous membranes		-	-	-	-	-	-	-	-	-	-
respiratory system		-	-	-	-	-	-	-	-	-	-
somatomotor activity		-	-	-	-	-	-	-	-	-	-
behaviour pattern		-	-	-	-	-	-	-	-	-	-
tremors and convulsions		-	-	-	-	-	-	-	-	-	-
salivation		-	-	-	-	-	-	-	-	-	-
diarrhoea		-	-	-	-	-	-	-	-	-	-
lethargy		-	-	-	-	-	-	-	-	-	-
sleep or coma		-	-	-	-	-	-	-	-	-	-
other(specify)		-	-	-	-	-	-	-	-	-	-
**DAY 28**											
general appearance		-	-	-	-	-	-	-	-	-	-
skin and fur		-	-	-	-	-	-	-	-	-	-
eyes/mucous membranes		-	-	-	-	-	-	-	-	-	-
respiratory system		-	-	-	-	-	-	-	-	-	-
somatomotor activity		-	-	-	-	-	-	-	-	-	-
behaviour pattern		-	-	-	-	-	-	-	-	-	-
tremors and convulsions		-	-	-	-	-	-	-	-	-	-
salivation		-	-	-	-	-	-	-	-	-	-
diarrhoea		-	-	-	-	-	-	-	-	-	-
lethargy		-	-	-	-	-	-	-	-	-	-
sleep or coma		-	-	-	-	-	-	-	-	-	-
other(specify)		-	-	-	-	-	-	-	-	-	-
**DAY 29**											
general appearance		-	-	-	-	-	-	-	-	-	-
skin and fur		-	-	-	-	-	-	-	-	-	-
eyes/mucous membranes		-	-	-	-	-	-	-	-	-	-
respiratory system		-	-	-	-	-	-	-	-	-	-
somatomotor activity		-	-	-	-	-	-	-	-	-	-
behaviour pattern		-	-	-	-	-	-	-	-	-	-
tremors and convulsions		-	-	-	-	-	-	-	-	-	-
salivation		-	-	-	-	-	-	-	-	-	-
diarrhoea		-	-	-	-	-	-	-	-	-	-
lethargy		-	-	-	-	-	-	-	-	-	-
sleep or coma		-	-	-	-	-	-	-	-	-	-
other(specify)		-	-	-	-	-	-	-	-	-	-

ENTERED BY: SQ 10/2/12

CHECKED BY: [illegible] 20/2/12

ICPQN1035.B

**LAB-028/f1 & LAB-028/F2 INDIVIDUAL CLINICAL OBSERVATIONS - DAILY**	GROUP	2	2	2	2	2	2	2	2	2	2
	Treatment	TI/458	TI/458	TI/458	TI/458	TI/458	TI/458	TI/458	TI/458	TI/458	TI/458
	Dose (mg/kg)	1	1	1	1	1	1	1	1	1	1
**Note:** -, No abnormalities;	Animal ID	R2419	R2420	R2421	R2422	R2423	R2424	R2425	R2426	R2427	R2428
P- porphyrin discharge around eye	Sex	M	M	M	M	M	M	M	M	M	M
**DAY 1**											
general appearance		-	-	-	-	-	-	-	-	-	-
skin and fur		-	-	-	-	-	-	-	-	-	-
eyes/mucous membranes		-	-	-	-	-	-	-	-	-	-
respiratory system		-	-	-	-	-	-	-	-	-	-
somatomotor activity		-	-	-	-	-	-	-	-	-	-
behaviour pattern		-	-	-	-	-	-	-	-	-	-
tremors and convulsions		-	-	-	-	-	-	-	-	-	-
salivation		-	-	-	-	-	-	-	-	-	-
diarrhoea		-	-	-	-	-	-	-	-	-	-
lethargy		-	-	-	-	-	-	-	-	-	-
sleep or coma		-	-	-	-	-	-	-	-	-	-
other(specify)		-	-	-	-	-	-	-	-	-	-
**DAY 2**											
general appearance		-	-	-	-	-	-	-	-	-	-
skin and fur		-	-	-	-	-	-	-	-	-	-
eyes/mucous membranes		-	-	-	-	-	-	-	-	-	-
respiratory system		-	-	-	-	-	-	-	-	-	-
somatomotor activity		-	-	-	-	-	-	-	-	-	-
behaviour pattern		-	-	-	-	-	-	-	-	-	-
tremors and convulsions		-	-	-	-	-	-	-	-	-	-
salivation		-	-	-	-	-	-	-	-	-	-
diarrhoea		-	-	-	-	-	-	-	-	-	-
lethargy		-	-	-	-	-	-	-	-	-	-
sleep or coma		-	-	-	-	-	-	-	-	-	-
other(specify)		-	-	-	-	-	-	-	-	-	-
**DAY 3**											
general appearance		-	-	-	-	-	-	-	-	-	-
skin and fur		-	-	-	-	-	-	-	-	-	-
eyes/mucous membranes		-	-	-	-	-	-	-	-	-	-
respiratory system		-	-	-	-	-	-	-	-	-	-
somatomotor activity		-	-	-	-	-	-	-	-	-	-
behaviour pattern		-	-	-	-	-	-	-	-	-	-
tremors and convulsions		-	-	-	-	-	-	-	-	-	-
salivation		-	-	-	-	-	-	-	-	-	-
diarrhoea		-	-	-	-	-	-	-	-	-	-
lethargy		-	-	-	-	-	-	-	-	-	-
sleep or coma		-	-	-	-	-	-	-	-	-	-
other(specify)		-	-	-	-	-	-	-	-	-	-
**DAY 4**											
general appearance		-	-	-	-	-	-	-	-	-	-
skin and fur		-	-	-	-	-	-	-	-	-	-
eyes/mucous membranes		-	-	-	-	-	-	-	-	-	-
respiratory system		-	-	-	-	-	-	-	-	-	-
somatomotor activity		-	-	-	-	-	-	-	-	-	-
behaviour pattern		-	-	-	-	-	-	-	-	-	-
tremors and convulsions		-	-	-	-	-	-	-	-	-	-
salivation		-	-	-	-	-	-	-	-	-	-
diarrhoea		-	-	-	-	-	-	-	-	-	-
lethargy		-	-	-	-	-	-	-	-	-	-
sleep or coma		-	-	-	-	-	-	-	-	-	-
other(specify)		-	-	-	-	-	-	-	-	-	-
**DAY 5**											
general appearance		-	-	-	-	-	-	-	-	-	-
skin and fur		-	-	-	-	-	-	-	-	-	-
eyes/mucous membranes		-	-	-	-	-	-	-	-	-	-
respiratory system		-	-	-	-	-	-	-	-	-	-
somatomotor activity		-	-	-	-	-	-	-	-	-	-
behaviour pattern		-	-	-	-	-	-	-	-	-	-
tremors and convulsions		-	-	-	-	-	-	-	-	-	-
salivation		-	-	-	-	-	-	-	-	-	-
diarrhoea		-	-	-	-	-	-	-	-	-	-
lethargy		-	-	-	-	-	-	-	-	-	-
sleep or coma		-	-	-	-	-	-	-	-	-	-
other(specify)		-	-	-	-	-	-	-	-	-	-
**DAY 6**											
general appearance		-	-	-	-	-	-	-	-	-	-
skin and fur		-	-	-	-	-	-	-	-	-	-
eyes/mucous membranes		-	-	-	-	-	-	-	-	-	-
respiratory system		-	-	-	-	-	-	-	-	-	-
somatomotor activity		-	-	-	-	-	-	-	-	-	-
behaviour pattern		-	-	-	-	-	-	-	-	-	-
tremors and convulsions		-	-	-	-	-	-	-	-	-	-
salivation		-	-	-	-	-	-	-	-	-	-
diarrhoea		-	-	-	-	-	-	-	-	-	-
lethargy		-	-	-	-	-	-	-	-	-	-
sleep or coma		-	-	-	-	-	-	-	-	-	-
other(specify)		-	-	-	-	-	-	-	-	-	-

ENTERED BY: SR 10/2/12
CHECKED BY: [illegible] 20/2/12

ICPQN1035.B

**LAB-028/f1 & LAB-028/F2 INDIVIDUAL CLINICAL OBSERVATIONS - DAILY**	GROUP	2	2	2	2	2	2	2	2	2	2
	Treatment	TI/458	TI/458	TI/458	TI/458	TI/458	TI/458	TI/458	TI/458	TI/458	TI/458
	Dose (mg/kg)	1	1	1	1	1	1	1	1	1	1
**Note:** -, No abnormalities;	Animal ID	R2419	R2420	R2421	R2422	R2423	R2424	R2425	R2426	R2427	R2428
P- porphyrin discharge around eye	Sex	M	M	M	M	M	M	M	M	M	M
**DAY 7**											
general appearance		-	-	-	-	-	-	-	-	-	-
skin and fur		-	-	-	-	-	-	-	-	-	-
eyes/mucous membranes		-	-	-	-	-	-	-	-	-	-
respiratory system		-	-	-	-	-	-	-	-	-	-
somatomotor activity		-	-	-	-	-	-	-	-	-	-
behaviour pattern		-	-	-	-	-	-	-	-	-	-
tremors and convulsions		-	-	-	-	-	-	-	-	-	-
salivation		-	-	-	-	-	-	-	-	-	-
diarrhoea		-	-	-	-	-	-	-	-	-	-
lethargy		-	-	-	-	-	-	-	-	-	-
sleep or coma		-	-	-	-	-	-	-	-	-	-
other(specify)		-	-	-	-	-	-	-	-	-	-
**DAY 8**											
general appearance		-	-	-	-	-	-	-	-	-	-
skin and fur		-	-	-	-	-	-	-	-	-	-
eyes/mucous membranes		-	-	-	-	-	-	-	-	-	-
respiratory system		-	-	-	-	-	-	-	-	-	-
somatomotor activity		-	-	-	-	-	-	-	-	-	-
behaviour pattern		-	-	-	-	-	-	-	-	-	-
tremors and convulsions		-	-	-	-	-	-	-	-	-	-
salivation		-	-	-	-	-	-	-	-	-	-
diarrhoea		-	-	-	-	-	-	-	-	-	-
lethargy		-	-	-	-	-	-	-	-	-	-
sleep or coma		-	-	-	-	-	-	-	-	-	-
other(specify)		-	-	-	-	-	-	-	-	-	-
**DAY 9**											
general appearance		-	-	-	-	-	-	-	-	-	-
skin and fur		-	-	-	-	-	-	-	-	-	-
eyes/mucous membranes		-	-	-	-	-	-	-	-	-	-
respiratory system		-	-	-	-	-	-	-	-	-	-
somatomotor activity		-	-	-	-	-	-	-	-	-	-
behaviour pattern		-	-	-	-	-	-	-	-	-	-
tremors and convulsions		-	-	-	-	-	-	-	-	-	-
salivation		-	-	-	-	-	-	-	-	-	-
diarrhoea		-	-	-	-	-	-	-	-	-	-
lethargy		-	-	-	-	-	-	-	-	-	-
sleep or coma		-	-	-	-	-	-	-	-	-	-
other(specify)		-	-	-	-	-	-	-	-	-	-
**DAY 10**											
general appearance		-	-	-	-	-	-	-	-	-	-
skin and fur		-	-	-	-	-	-	-	-	-	-
eyes/mucous membranes		-	-	-	-	-	-	-	-	-	-
respiratory system		-	-	-	-	-	-	-	-	-	-
somatomotor activity		-	-	-	-	-	-	-	-	-	-
behaviour pattern		-	-	-	-	-	-	-	-	-	-
tremors and convulsions		-	-	-	-	-	-	-	-	-	-
salivation		-	-	-	-	-	-	-	-	-	-
diarrhoea		-	-	-	-	-	-	-	-	-	-
lethargy		-	-	-	-	-	-	-	-	-	-
sleep or coma		-	-	-	-	-	-	-	-	-	-
other(specify)		-	-	-	-	-	-	-	-	-	-
**DAY 11**											
general appearance		-	-	-	-	-	-	-	-	-	-
skin and fur		-	-	-	-	-	-	-	-	-	-
eyes/mucous membranes		-	-	-	-	-	-	-	-	-	-
respiratory system		-	-	-	-	-	-	-	-	-	-
somatomotor activity		-	-	-	-	-	-	-	-	-	-
behaviour pattern		-	-	-	-	-	-	-	-	-	-
tremors and convulsions		-	-	-	-	-	-	-	-	-	-
salivation		-	-	-	-	-	-	-	-	-	-
diarrhoea		-	-	-	-	-	-	-	-	-	-
lethargy		-	-	-	-	-	-	-	-	-	-
sleep or coma		-	-	-	-	-	-	-	-	-	-
other(specify)		-	-	-	-	-	-	-	-	-	-
**DAY 12**											
general appearance		-	-	-	-	-	-	-	-	-	-
skin and fur		-	-	-	-	-	-	-	-	-	-
eyes/mucous membranes		-	-	-	-	-	-	-	-	-	-
respiratory system		-	-	-	-	-	-	-	-	-	-
somatomotor activity		-	-	-	-	-	-	-	-	-	-
behaviour pattern		-	-	-	-	-	-	-	-	-	-
tremors and convulsions		-	-	-	-	-	-	-	-	-	-
salivation		-	-	-	-	-	-	-	-	-	-
diarrhoea		-	-	-	-	-	-	-	-	-	-
lethargy		-	-	-	-	-	-	-	-	-	-
sleep or coma		-	-	-	-	-	-	-	-	-	-
other(specify)		-	-	-	-	-	-	-	-	-	-

ENTERED BY: 82 10/2/12

CHECKED BY: [illegible] 20/2/12

ICPQN1035.B

LAB-028/f1 & LAB-028/F2 INDIVIDUAL CLINICAL OBSERVATIONS - DAILY	GROUP	2	2	2	2	2	2	2	2	2	2
	Treatment	TI/458	TI/458	TI/458	TI/458	TI/458	TI/458	TI/458	TI/458	TI/458	TI/458
	Dose (mg/kg)	1	1	1	1	1	1	1	1	1	1
Note: -, No abnormalities;	Animal ID	R2419	R2420	R2421	R2422	R2423	R2424	R2425	R2426	R2427	R2428
P- porphyrin discharge around eye	Sex	M	M	M	M	M	M	M	M	M	M
**DAY 13**											
general appearance		-	-	-	-	-	-	-	-	-	-
skin and fur		-	-	-	-	-	-	-	-	-	-
eyes/mucous membranes		-	-	-	-	-	-	-	-	-	-
respiratory system		-	-	-	-	-	-	-	-	-	-
somatomotor activity		-	-	-	-	-	-	-	-	-	-
behaviour pattern		-	-	-	-	-	-	-	-	-	-
tremors and convulsions		-	-	-	-	-	-	-	-	-	-
salivation		-	-	-	-	-	-	-	-	-	-
diarrhoea		-	-	-	-	-	-	-	-	-	-
lethargy		-	-	-	-	-	-	-	-	-	-
sleep or coma		-	-	-	-	-	-	-	-	-	-
other(specify)		-	-	-	-	-	-	-	-	-	-
**DAY 14**											
general appearance		-	-	-	-	-	-	-	-	-	-
skin and fur		-	-	-	-	-	-	-	-	-	-
eyes/mucous membranes		-	-	-	-	-	-	-	-	-	-
respiratory system		-	-	-	-	-	-	-	-	-	-
somatomotor activity		-	-	-	-	-	-	-	-	-	-
behaviour pattern		-	-	-	-	-	-	-	-	-	-
tremors and convulsions		-	-	-	-	-	-	-	-	-	-
salivation		-	-	-	-	-	-	-	-	-	-
diarrhoea		-	-	-	-	-	-	-	-	-	-
lethargy		-	-	-	-	-	-	-	-	-	-
sleep or coma		-	-	-	-	-	-	-	-	-	-
other(specify)		-	-	-	-	-	-	-	-	-	-
**DAY 15**											
general appearance		-	-	-	-	-	-	-	-	-	-
skin and fur		-	-	-	-	-	-	-	-	-	-
eyes/mucous membranes		-	-	-	-	-	-	-	-	-	-
respiratory system		-	-	-	-	-	-	-	-	-	-
somatomotor activity		-	-	-	-	-	-	-	-	-	-
behaviour pattern		-	-	-	-	-	-	-	-	-	-
tremors and convulsions		-	-	-	-	-	-	-	-	-	-
salivation		-	-	-	-	-	-	-	-	-	-
diarrhoea		-	-	-	-	-	-	-	-	-	-
lethargy		-	-	-	-	-	-	-	-	-	-
sleep or coma		-	-	-	-	-	-	-	-	-	-
other(specify)		-	-	-	-	-	-	-	-	-	-
**DAY 16**											
general appearance		-	-	-	-	-	-	-	-	-	-
skin and fur		-	-	-	-	-	-	-	-	-	-
eyes/mucous membranes		-	-	-	-	-	-	-	-	-	-
respiratory system		-	-	-	-	-	-	-	-	-	-
somatomotor activity		-	-	-	-	-	-	-	-	-	-
behaviour pattern		-	-	-	-	-	-	-	-	-	-
tremors and convulsions		-	-	-	-	-	-	-	-	-	-
salivation		-	-	-	-	-	-	-	-	-	-
diarrhoea		-	-	-	-	-	-	-	-	-	-
lethargy		-	-	-	-	-	-	-	-	-	-
sleep or coma		-	-	-	-	-	-	-	-	-	-
other(specify)		-	-	-	-	-	-	-	-	-	-
**DAY 17**											
general appearance		-	-	-	-	-	-	-	-	-	-
skin and fur		-	-	-	-	-	-	-	-	-	-
eyes/mucous membranes		-	-	-	-	-	-	-	-	-	-
respiratory system		-	-	-	-	-	-	-	-	-	-
somatomotor activity		-	-	-	-	-	-	-	-	-	-
behaviour pattern		-	-	-	-	-	-	-	-	-	-
tremors and convulsions		-	-	-	-	-	-	-	-	-	-
salivation		-	-	-	-	-	-	-	-	-	-
diarrhoea		-	-	-	-	-	-	-	-	-	-
lethargy		-	-	-	-	-	-	-	-	-	-
sleep or coma		-	-	-	-	-	-	-	-	-	-
other(specify)		-	-	-	-	-	-	-	-	-	-
**DAY 18**											
general appearance		-	-	-	-	-	-	-	-	-	-
skin and fur		-	-	-	-	-	-	-	-	-	-
eyes/mucous membranes		-	-	-	-	-	-	-	-	-	-
respiratory system		-	-	-	-	-	-	-	-	-	-
somatomotor activity		-	-	-	-	-	-	-	-	-	-
behaviour pattern		-	-	-	-	-	-	-	-	-	-
tremors and convulsions		-	-	-	-	-	-	-	-	-	-
salivation		-	-	-	-	-	-	-	-	-	-
diarrhoea		-	-	-	-	-	-	-	-	-	-
lethargy		-	-	-	-	-	-	-	-	-	-
sleep or coma		-	-	-	-	-	-	-	-	-	-
other(specify)		-	-	-	-	-	-	-	-	-	-

ENTERED BY: [illegible] 19/2/12

CHECKED BY: [illegible] 20/2/12

ICPQN1035.B

**LAB-028/f1 & LAB-028/F2 INDIVIDUAL CLINICAL OBSERVATIONS - DAILY**	GROUP	2	2	2	2	2	2	2	2	2	2
	Treatment	TI/458	TI/458	TI/458	TI/458	TI/458	TI/458	TI/458	TI/458	TI/458	TI/458
	Dose (mg/kg)	1	1	1	1	1	1	1	1	1	1
**Note:** -, No abnormalities;	Animal ID	R2419	R2420	R2421	R2422	R2423	R2424	R2425	R2426	R2427	R2428
P- porphyrin discharge around eye	Sex	M	M	M	M	M	M	M	M	M	M
**DAY 19**											
general appearance		-	-	-	-	-	-	-	-	-	-
skin and fur		-	-	-	-	-	-	-	-	-	-
eyes/mucous membranes		-	-	-	-	-	-	-	-	-	-
respiratory system		-	-	-	-	-	-	-	-	-	-
somatomotor activity		-	-	-	-	-	-	-	-	-	-
behaviour pattern		-	-	-	-	-	-	-	-	-	-
tremors and convulsions		-	-	-	-	-	-	-	-	-	-
salivation		-	-	-	-	-	-	-	-	-	-
diarrhoea		-	-	-	-	-	-	-	-	-	-
lethargy		-	-	-	-	-	-	-	-	-	-
sleep or coma		-	-	-	-	-	-	-	-	-	-
other(specify)		-	-	-	-	-	-	-	-	-	-
**DAY 20**											
general appearance		-	-	-	-	-	-	-	-	-	-
skin and fur		-	-	-	-	-	-	-	-	-	-
eyes/mucous membranes		-	-	-	-	-	-	-	-	-	-
respiratory system		-	-	-	-	-	-	-	-	-	-
somatomotor activity		-	-	-	-	-	-	-	-	-	-
behaviour pattern		-	-	-	-	-	-	-	-	-	-
tremors and convulsions		-	-	-	-	-	-	-	-	-	-
salivation		-	-	-	-	-	-	-	-	-	-
diarrhoea		-	-	-	-	-	-	-	-	-	-
lethargy		-	-	-	-	-	-	-	-	-	-
sleep or coma		-	-	-	-	-	-	-	-	-	-
other(specify)		-	-	-	-	-	-	-	-	-	-
**DAY 21**											
general appearance		-	-	-	-	-	-	-	-	-	-
skin and fur		-	-	-	-	-	-	-	-	-	-
eyes/mucous membranes		-	-	-	-	-	-	-	-	-	-
respiratory system		-	-	-	-	-	-	-	-	-	-
somatomotor activity		-	-	-	-	-	-	-	-	-	-
behaviour pattern		-	-	-	-	-	-	-	-	-	-
tremors and convulsions		-	-	-	-	-	-	-	-	-	-
salivation		-	-	-	-	-	-	-	-	-	-
diarrhoea		-	-	-	-	-	-	-	-	-	-
lethargy		-	-	-	-	-	-	-	-	-	-
sleep or coma		-	-	-	-	-	-	-	-	-	-
other(specify)		-	-	-	-	-	-	-	-	-	-
**DAY 22**											
general appearance		-	-	-	-	-	-	-	-	-	-
skin and fur		-	-	-	-	-	-	-	-	-	-
eyes/mucous membranes		-	-	-	-	-	-	-	-	-	-
respiratory system		-	-	-	-	-	-	-	-	-	-
somatomotor activity		-	-	-	-	-	-	-	-	-	-
behaviour pattern		-	-	-	-	-	-	-	-	-	-
tremors and convulsions		-	-	-	-	-	-	-	-	-	-
salivation		-	-	-	-	-	-	-	-	-	-
diarrhoea		-	-	-	-	-	-	-	-	-	-
lethargy		-	-	-	-	-	-	-	-	-	-
sleep or coma		-	-	-	-	-	-	-	-	-	-
other(specify)		-	-	-	-	-	-	-	-	-	-
**DAY 23**											
general appearance		-	-	-	-	-	-	-	-	-	-
skin and fur		-	-	-	-	-	-	-	-	-	-
eyes/mucous membranes		-	-	-	-	-	-	-	-	-	-
respiratory system		-	-	-	-	-	-	-	-	-	-
somatomotor activity		-	-	-	-	-	-	-	-	-	-
behaviour pattern		-	-	-	-	-	-	-	-	-	-
tremors and convulsions		-	-	-	-	-	-	-	-	-	-
salivation		-	-	-	-	-	-	-	-	-	-
diarrhoea		-	-	-	-	-	-	-	-	-	-
lethargy		-	-	-	-	-	-	-	-	-	-
sleep or coma		-	-	-	-	-	-	-	-	-	-
other(specify)		-	-	-	-	-	-	-	-	-	-
**DAY 24**											
general appearance		-	-	-	-	-	-	-	-	-	-
skin and fur		-	-	-	-	-	-	-	-	-	-
eyes/mucous membranes		-	-	-	-	-	-	-	-	-	-
respiratory system		-	-	-	-	-	-	-	-	-	-
somatomotor activity		-	-	-	-	-	-	-	-	-	-
behaviour pattern		-	-	-	-	-	-	-	-	-	-
tremors and convulsions		-	-	-	-	-	-	-	-	-	-
salivation		-	-	-	-	-	-	-	-	-	-
diarrhoea		-	-	-	-	-	-	-	-	-	-
lethargy		-	-	-	-	-	-	-	-	-	-
sleep or coma		-	-	-	-	-	-	-	-	-	-
other(specify)		-	-	-	-	-	-	-	-	-	-

ENTERED BY: [illegible] 10/2/12

CHECKED BY: [illegible] 20/2/12

ICPQN1035.B

**LAB-028/f1 & LAB-028/F2 INDIVIDUAL CLINICAL OBSERVATIONS - DAILY**	GROUP	2	2	2	2	2	2	2	2	2	2
	Treatment	TI/458	TI/458	TI/458	TI/458	TI/458	TI/458	TI/458	TI/458	TI/458	TI/458
	Dose (mg/kg)	1	1	1	1	1	1	1	1	1	1
**Note:** -, No abnormalities;	Animal ID	R2419	R2420	R2421	R2422	R2423	R2424	R2425	R2426	R2427	R2428
P- porphyrin discharge around eye	Sex	M	M	M	M	M	M	M	M	M	M
**DAY 25**											
general appearance		-	-	-	-	-	-	-	-	-	-
skin and fur		-	-	-	-	-	-	-	-	-	-
eyes/mucous membranes		-	-	-	-	-	-	-	-	-	-
respiratory system		-	-	-	-	-	-	-	-	-	-
somatomotor activity		-	-	-	-	-	-	-	-	-	-
behaviour pattern		-	-	-	-	-	-	-	-	-	-
tremors and convulsions		-	-	-	-	-	-	-	-	-	-
salivation		-	-	-	-	-	-	-	-	-	-
diarrhoea		-	-	-	-	-	-	-	-	-	-
lethargy		-	-	-	-	-	-	-	-	-	-
sleep or coma		-	-	-	-	-	-	-	-	-	-
other(specify)		-	-	-	-	-	-	-	-	-	-
**DAY 26**											
general appearance		-	-	-	-	-	-	-	-	-	-
skin and fur		-	-	-	-	-	-	-	-	-	-
eyes/mucous membranes		-	-	-	-	-	-	-	-	-	-
respiratory system		-	-	-	-	-	-	-	-	-	-
somatomotor activity		-	-	-	-	-	-	-	-	-	-
behaviour pattern		-	-	-	-	-	-	-	-	-	-
tremors and convulsions		-	-	-	-	-	-	-	-	-	-
salivation		-	-	-	-	-	-	-	-	-	-
diarrhoea		-	-	-	-	-	-	-	-	-	-
lethargy		-	-	-	-	-	-	-	-	-	-
sleep or coma		-	-	-	-	-	-	-	-	-	-
other(specify)		-	-	-	-	-	-	-	-	-	-
**DAY 27**											
general appearance		-	-	-	-	-	-	-	-	-	-
skin and fur		-	-	-	-	-	-	-	-	-	-
eyes/mucous membranes		-	-	-	-	-	-	-	-	-	-
respiratory system		-	-	-	-	-	-	-	-	-	-
somatomotor activity		-	-	-	-	-	-	-	-	-	-
behaviour pattern		-	-	-	-	-	-	-	-	-	-
tremors and convulsions		-	-	-	-	-	-	-	-	-	-
salivation		-	-	-	-	-	-	-	-	-	-
diarrhoea		-	-	-	-	-	-	-	-	-	-
lethargy		-	-	-	-	-	-	-	-	-	-
sleep or coma		-	-	-	-	-	-	-	-	-	-
other(specify)		-	-	-	-	-	-	-	-	-	-
**DAY 28**											
general appearance		-	-	-	-	-	-	-	-	-	-
skin and fur		-	-	-	-	-	-	-	-	-	-
eyes/mucous membranes		-	-	-	-	-	-	-	-	-	-
respiratory system		-	-	-	-	-	-	-	-	-	-
somatomotor activity		-	-	-	-	-	-	-	-	-	-
behaviour pattern		-	-	-	-	-	-	-	-	-	-
tremors and convulsions		-	-	-	-	-	-	-	-	-	-
salivation		-	-	-	-	-	-	-	-	-	-
diarrhoea		-	-	-	-	-	-	-	-	-	-
lethargy		-	-	-	-	-	-	-	-	-	-
sleep or coma		-	-	-	-	-	-	-	-	-	-
other(specify)		-	-	-	-	-	-	-	-	-	-
**DAY 29**											
general appearance		-	-	-	-	-	-	-	-	-	-
skin and fur		-	-	-	-	-	-	-	-	-	-
eyes/mucous membranes		-	-	-	-	-	-	-	-	-	-
respiratory system		-	-	-	-	-	-	-	-	-	-
somatomotor activity		-	-	-	-	-	-	-	-	-	-
behaviour pattern		-	-	-	-	-	-	-	-	-	-
tremors and convulsions		-	-	-	-	-	-	-	-	-	-
salivation		-	-	-	-	-	-	-	-	-	-
diarrhoea		-	-	-	-	-	-	-	-	-	-
lethargy		-	-	-	-	-	-	-	-	-	-
sleep or coma		-	-	-	-	-	-	-	-	-	-
other(specify)		-	-	-	-	-	-	-	-	-	-

ENTERED BY: SQ 20/2/12
CHECKED BY: d 20/2/12

ICPQN1035.B

LAB-028/f1 & LAB-028/F2 INDIVIDUAL CLINICAL OBSERVATIONS - DAILY	GROUP	3	3	3	3	3	3	3	3	3	3
	Treatment	TI/458	TI/458	TI/458	TI/458	TI/458	TI/458	TI/458	TI/458	TI/458	TI/458
	Dose (mg/kg)	20	20	20	20	20	20	20	20	20	20
Note: -, No abnormalities;	Animal ID	R2429	R2430	R2431	R2432	R2433	R2434	R2435	R2436	R2437	R2438
P- porphyrin discharge around eye	Sex	M	M	M	M	M	M	M	M	M	M
**DAY 1**											
general appearance		-	-	-	-	-	-	-	-	-	-
skin and fur		-	-	-	-	-	-	-	-	-	-
eyes/mucous membranes		-	-	-	-	-	-	-	-	-	-
respiratory system		-	-	-	-	-	-	-	-	-	-
somatomotor activity		-	-	-	-	-	-	-	-	-	-
behaviour pattern		-	-	-	-	-	-	-	-	-	-
tremors and convulsions		-	-	-	-	-	-	-	-	-	-
salivation		-	-	-	-	-	-	-	-	-	-
diarrhoea		-	-	-	-	-	-	-	-	-	-
lethargy		-	-	-	-	-	-	-	-	-	-
sleep or coma		-	-	-	-	-	-	-	-	-	-
other(specify)		-	-	-	-	-	-	-	-	-	-
**DAY 2**											
general appearance		-	-	-	-	-	-	-	-	-	-
skin and fur		-	-	-	-	-	-	-	-	-	-
eyes/mucous membranes		-	-	-	-	-	-	-	-	-	-
respiratory system		-	-	-	-	-	-	-	-	-	-
somatomotor activity		-	-	-	-	-	-	-	-	-	-
behaviour pattern		-	-	-	-	-	-	-	-	-	-
tremors and convulsions		-	-	-	-	-	-	-	-	-	-
salivation		-	-	-	-	-	-	-	-	-	-
diarrhoea		-	-	-	-	-	-	-	-	-	-
lethargy		-	-	-	-	-	-	-	-	-	-
sleep or coma		-	-	-	-	-	-	-	-	-	-
other(specify)		-	-	-	-	-	-	-	-	-	-
**DAY 3**											
general appearance		-	-	-	-	-	-	-	-	-	-
skin and fur		-	-	-	-	-	-	-	-	-	-
eyes/mucous membranes		-	-	-	-	-	-	-	-	-	-
respiratory system		-	-	-	-	-	-	-	-	-	-
somatomotor activity		-	-	-	-	-	-	-	-	-	-
behaviour pattern		-	-	-	-	-	-	-	-	-	-
tremors and convulsions		-	-	-	-	-	-	-	-	-	-
salivation		-	-	-	-	-	-	-	-	-	-
diarrhoea		-	-	-	-	-	-	-	-	-	-
lethargy		-	-	-	-	-	-	-	-	-	-
sleep or coma		-	-	-	-	-	-	-	-	-	-
other(specify)		-	-	-	-	-	-	-	-	-	-
**DAY 4**											
general appearance		-	-	-	-	-	-	-	-	-	-
skin and fur		-	-	-	-	-	-	-	-	-	-
eyes/mucous membranes		-	-	-	-	-	-	-	-	-	-
respiratory system		-	-	-	-	-	-	-	-	-	-
somatomotor activity		-	-	-	-	-	-	-	-	-	-
behaviour pattern		-	-	-	-	-	-	-	-	-	-
tremors and convulsions		-	-	-	-	-	-	-	-	-	-
salivation		-	-	-	-	-	-	-	-	-	-
diarrhoea		-	-	-	-	-	-	-	-	-	-
lethargy		-	-	-	-	-	-	-	-	-	-
sleep or coma		-	-	-	-	-	-	-	-	-	-
other(specify)		-	-	-	-	-	-	-	-	-	-
**DAY 5**											
general appearance		-	-	-	-	-	-	-	-	-	-
skin and fur		-	-	-	-	-	-	-	-	-	-
eyes/mucous membranes		-	-	-	-	-	-	-	-	-	-
respiratory system		-	-	-	-	-	-	-	-	-	-
somatomotor activity		-	-	-	-	-	-	-	-	-	-
behaviour pattern		-	-	-	-	-	-	-	-	-	-
tremors and convulsions		-	-	-	-	-	-	-	-	-	-
salivation		-	-	-	-	-	-	-	-	-	-
diarrhoea		-	-	-	-	-	-	-	-	-	-
lethargy		-	-	-	-	-	-	-	-	-	-
sleep or coma		-	-	-	-	-	-	-	-	-	-
other(specify)		-	-	-	-	-	-	-	-	-	-
**DAY 6**											
general appearance		-	-	-	-	-	-	-	-	-	-
skin and fur		-	-	-	-	-	-	-	-	-	-
eyes/mucous membranes		-	-	-	-	-	-	-	-	-	-
respiratory system		-	-	-	-	-	-	-	-	-	-
somatomotor activity		-	-	-	-	-	-	-	-	-	-
behaviour pattern		-	-	-	-	-	-	-	-	-	-
tremors and convulsions		-	-	-	-	-	-	-	-	-	-
salivation		-	-	-	-	-	-	-	-	-	-
diarrhoea		-	-	-	-	-	-	-	-	-	-
lethargy		-	-	-	-	-	-	-	-	-	-
sleep or coma		-	-	-	-	-	-	-	-	-	-
other(specify)		-	-	-	-	-	-	-	-	-	-

ENTERED BY: ε2 10/2/12

CHECKED BY: d 20/2/12

ICPQN1035.B

**LAB-028/f1 & LAB-028/F2 INDIVIDUAL CLINICAL OBSERVATIONS - DAILY**	GROUP	3	3	3	3	3	3	3	3	3	3
	Treatment	TI/458	TI/458	TI/458	TI/458	TI/458	TI/458	TI/458	TI/458	TI/458	TI/458
	Dose (mg/kg)	20	20	20	20	20	20	20	20	20	20
**Note:** -, No abnormalities;	Animal ID	R2429	R2430	R2431	R2432	R2433	R2434	R2435	R2436	R2437	R2438
P- porphyrin discharge around eye	Sex	M	M	M	M	M	M	M	M	M	M
**DAY 7**											
general appearance		-	-	-	-	-	-	-	-	-	-
skin and fur		-	-	-	-	-	-	-	-	-	-
eyes/mucous membranes		-	-	-	-	-	-	-	-	-	-
respiratory system		-	-	-	-	-	-	-	-	-	-
somatomotor activity		-	-	-	-	-	-	-	-	-	-
behaviour pattern		-	-	-	-	-	-	-	-	-	-
tremors and convulsions		-	-	-	-	-	-	-	-	-	-
salivation		-	-	-	-	-	-	-	-	-	-
diarrhoea		-	-	-	-	-	-	-	-	-	-
lethargy		-	-	-	-	-	-	-	-	-	-
sleep or coma		-	-	-	-	-	-	-	-	-	-
other(specify)		-	-	-	-	-	-	-	-	-	-
**DAY 8**											
general appearance		-	-	-	-	-	-	-	-	-	-
skin and fur		-	-	-	-	-	-	-	-	-	-
eyes/mucous membranes		-	-	-	-	-	-	-	-	-	-
respiratory system		-	-	-	-	-	-	-	-	-	-
somatomotor activity		-	-	-	-	-	-	-	-	-	-
behaviour pattern		-	-	-	-	-	-	-	-	-	-
tremors and convulsions		-	-	-	-	-	-	-	-	-	-
salivation		-	-	-	-	-	-	-	-	-	-
diarrhoea		-	-	-	-	-	-	-	-	-	-
lethargy		-	-	-	-	-	-	-	-	-	-
sleep or coma		-	-	-	-	-	-	-	-	-	-
other(specify)		-	-	-	-	-	-	-	-	-	-
**DAY 9**											
general appearance		-	-	-	-	-	-	-	-	-	-
skin and fur		-	-	-	-	-	-	-	-	-	-
eyes/mucous membranes		-	-	-	-	-	-	-	-	-	-
respiratory system		-	-	-	-	-	-	-	-	-	-
somatomotor activity		-	-	-	-	-	-	-	-	-	-
behaviour pattern		-	-	-	-	-	-	-	-	-	-
tremors and convulsions		-	-	-	-	-	-	-	-	-	-
salivation		-	-	-	-	-	-	-	-	-	-
diarrhoea		-	-	-	-	-	-	-	-	-	-
lethargy		-	-	-	-	-	-	-	-	-	-
sleep or coma		-	-	-	-	-	-	-	-	-	-
other(specify)		-	-	-	-	-	-	-	-	-	-
**DAY 10**											
general appearance		-	-	-	-	-	-	-	-	-	-
skin and fur		-	-	-	-	-	-	-	-	-	-
eyes/mucous membranes		-	-	-	-	-	-	-	-	-	-
respiratory system		-	-	-	-	-	-	-	-	-	-
somatomotor activity		-	-	-	-	-	-	-	-	-	-
behaviour pattern		-	-	-	-	-	-	-	-	-	-
tremors and convulsions		-	-	-	-	-	-	-	-	-	-
salivation		-	-	-	-	-	-	-	-	-	-
diarrhoea		-	-	-	-	-	-	-	-	-	-
lethargy		-	-	-	-	-	-	-	-	-	-
sleep or coma		-	-	-	-	-	-	-	-	-	-
other(specify)		-	-	-	-	-	-	-	-	-	-
**DAY 11**											
general appearance		-	-	-	-	-	-	-	-	-	-
skin and fur		-	-	-	-	-	-	-	-	-	-
eyes/mucous membranes		-	-	-	-	-	-	-	-	-	-
respiratory system		-	-	-	-	-	-	-	-	-	-
somatomotor activity		-	-	-	-	-	-	-	-	-	-
behaviour pattern		-	-	-	-	-	-	-	-	-	-
tremors and convulsions		-	-	-	-	-	-	-	-	-	-
salivation		-	-	-	-	-	-	-	-	-	-
diarrhoea		-	-	-	-	-	-	-	-	-	-
lethargy		-	-	-	-	-	-	-	-	-	-
sleep or coma		-	-	-	-	-	-	-	-	-	-
other(specify)		-	-	-	-	-	-	-	-	-	-
**DAY 12**											
general appearance		-	-	-	-	-	-	-	-	-	-
skin and fur		-	-	-	-	-	-	-	-	-	-
eyes/mucous membranes		-	-	-	-	-	-	-	-	-	-
respiratory system		-	-	-	-	-	-	-	-	-	-
somatomotor activity		-	-	-	-	-	-	-	-	-	-
behaviour pattern		-	-	-	-	-	-	-	-	-	-
tremors and convulsions		-	-	-	-	-	-	-	-	-	-
salivation		-	-	-	-	-	-	-	-	-	-
diarrhoea		-	-	-	-	-	-	-	-	-	-
lethargy		-	-	-	-	-	-	-	-	-	-
sleep or coma		-	-	-	-	-	-	-	-	-	-
other(specify)		-	-	-	-	-	-	-	-	-	-

ENTERED BY: EA 19/2/12
CHECKED BY: [illegible] 20/2/12

ICPQN1035.B

**LAB-028/f1 & LAB-028/F2 INDIVIDUAL CLINICAL OBSERVATIONS - DAILY**	GROUP	3	3	3	3	3	3	3	3	3	3
	Treatment	TI/458	TI/458	TI/458	TI/458	TI/458	TI/458	TI/458	TI/458	TI/458	TI/458
	Dose (mg/kg)	20	20	20	20	20	20	20	20	20	20
**Note:** -, No abnormalities;	Animal ID	R2429	R2430	R2431	R2432	R2433	R2434	R2435	R2436	R2437	R2438
P- porphyrin discharge around eye	Sex	M	M	M	M	M	M	M	M	M	M
**DAY 13**											
general appearance		-	-	-	-	-	-	-	-	-	-
skin and fur		-	-	-	-	-	-	-	-	-	-
eyes/mucous membranes		-	-	-	-	-	-	-	-	-	-
respiratory system		-	-	-	-	-	-	-	-	-	-
somatomotor activity		-	-	-	-	-	-	-	-	-	-
behaviour pattern		-	-	-	-	-	-	-	-	-	-
tremors and convulsions		-	-	-	-	-	-	-	-	-	-
salivation		-	-	-	-	-	-	-	-	-	-
diarrhoea		-	-	-	-	-	-	-	-	-	-
lethargy		-	-	-	-	-	-	-	-	-	-
sleep or coma		-	-	-	-	-	-	-	-	-	-
other(specify)		-	-	-	-	-	-	-	-	-	-
**DAY 14**											
general appearance		-	-	-	-	-	-	-	-	-	-
skin and fur		-	-	-	-	-	-	-	-	-	-
eyes/mucous membranes		-	-	-	-	-	-	-	-	-	-
respiratory system		-	-	-	-	-	-	-	-	-	-
somatomotor activity		-	-	-	-	-	-	-	-	-	-
behaviour pattern		-	-	-	-	-	-	-	-	-	-
tremors and convulsions		-	-	-	-	-	-	-	-	-	-
salivation		-	-	-	-	-	-	-	-	-	-
diarrhoea		-	-	-	-	-	-	-	-	-	-
lethargy		-	-	-	-	-	-	-	-	-	-
sleep or coma		-	-	-	-	-	-	-	-	-	-
other(specify)		-	-	-	-	-	-	-	-	-	-
**DAY 15**											
general appearance		-	-	-	-	-	-	-	-	-	-
skin and fur		-	-	-	-	-	-	-	-	-	-
eyes/mucous membranes		-	-	-	-	-	-	-	-	-	-
respiratory system		-	-	-	-	-	-	-	-	-	-
somatomotor activity		-	-	-	-	-	-	-	-	-	-
behaviour pattern		-	-	-	-	-	-	-	-	-	-
tremors and convulsions		-	-	-	-	-	-	-	-	-	-
salivation		-	-	-	-	-	-	-	-	-	-
diarrhoea		-	-	-	-	-	-	-	-	-	-
lethargy		-	-	-	-	-	-	-	-	-	-
sleep or coma		-	-	-	-	-	-	-	-	-	-
other(specify)		-	-	-	-	-	-	-	-	-	-
**DAY 16**											
general appearance		-	-	-	-	-	-	-	-	-	-
skin and fur		-	-	-	-	-	-	-	-	-	-
eyes/mucous membranes		-	-	-	-	-	-	-	-	-	-
respiratory system		-	-	-	-	-	-	-	-	-	-
somatomotor activity		-	-	-	-	-	-	-	-	-	-
behaviour pattern		-	-	-	-	-	-	-	-	-	-
tremors and convulsions		-	-	-	-	-	-	-	-	-	-
salivation		-	-	-	-	-	-	-	-	-	-
diarrhoea		-	-	-	-	-	-	-	-	-	-
lethargy		-	-	-	-	-	-	-	-	-	-
sleep or coma		-	-	-	-	-	-	-	-	-	-
other(specify)		-	-	-	-	-	-	-	-	-	-
**DAY 17**											
general appearance		-	-	-	-	-	-	-	-	-	-
skin and fur		-	-	-	-	-	-	-	-	-	-
eyes/mucous membranes		-	-	-	-	-	-	-	-	-	-
respiratory system		-	-	-	-	-	-	-	-	-	-
somatomotor activity		-	-	-	-	-	-	-	-	-	-
behaviour pattern		-	-	-	-	-	-	-	-	-	-
tremors and convulsions		-	-	-	-	-	-	-	-	-	-
salivation		-	-	-	-	-	-	-	-	-	-
diarrhoea		-	-	-	-	-	-	-	-	-	-
lethargy		-	-	-	-	-	-	-	-	-	-
sleep or coma		-	-	-	-	-	-	-	-	-	-
other(specify)		-	-	-	-	-	-	-	-	-	-
**DAY 18**											
general appearance		-	-	-	-	-	-	-	-	-	-
skin and fur		-	-	-	-	-	-	-	-	-	-
eyes/mucous membranes		-	-	-	-	-	-	-	-	-	-
respiratory system		-	-	-	-	-	-	-	-	-	-
somatomotor activity		-	-	-	-	-	-	-	-	-	-
behaviour pattern		-	-	-	-	-	-	-	-	-	-
tremors and convulsions		-	-	-	-	-	-	-	-	-	-
salivation		-	-	-	-	-	-	-	-	-	-
diarrhoea		-	-	-	-	-	-	-	-	-	-
lethargy		-	-	-	-	-	-	-	-	-	-
sleep or coma		-	-	-	-	-	-	-	-	-	-
other(specify)		-	-	-	-	-	-	-	-	-	-

ENTERED BY: 82 19/2/12

CHECKED BY: [illegible] 20/2/12

ICPQN1035.B

**LAB-028/f1 & LAB-028/F2 INDIVIDUAL CLINICAL OBSERVATIONS - DAILY**	GROUP	3	3	3	3	3	3	3	3	3	3
	Treatment	TI/458	TI/458	TI/458	TI/458	TI/458	TI/458	TI/458	TI/458	TI/458	TI/458
	Dose (mg/kg)	20	20	20	20	20	20	20	20	20	20
**Note:** -, No abnormalities;	Animal ID	R2429	R2430	R2431	R2432	R2433	R2434	R2435	R2436	R2437	R2438
P- porphyrin discharge around eye	Sex	M	M	M	M	M	M	M	M	M	M
**DAY 19**											
general appearance		-	-	-	-	-	-	-	-	-	-
skin and fur		-	-	-	-	-	-	-	-	-	-
eyes/mucous membranes		-	-	-	-	-	-	-	-	-	-
respiratory system		-	-	-	-	-	-	-	-	-	-
somatomotor activity		-	-	-	-	-	-	-	-	-	-
behaviour pattern		-	-	-	-	-	-	-	-	-	-
tremors and convulsions		-	-	-	-	-	-	-	-	-	-
salivation		-	-	-	-	-	-	-	-	-	-
diarrhoea		-	-	-	-	-	-	-	-	-	-
lethargy		-	-	-	-	-	-	-	-	-	-
sleep or coma		-	-	-	-	-	-	-	-	-	-
other(specify)		-	-	-	-	-	-	-	-	-	-
**DAY 20**											
general appearance		-	-	-	-	-	-	-	-	-	-
skin and fur		-	-	-	-	-	-	-	-	-	-
eyes/mucous membranes		-	-	-	-	-	-	-	-	-	-
respiratory system		-	-	-	-	-	-	-	-	-	-
somatomotor activity		-	-	-	-	-	-	-	-	-	-
behaviour pattern		-	-	-	-	-	-	-	-	-	-
tremors and convulsions		-	-	-	-	-	-	-	-	-	-
salivation		-	-	-	-	-	-	-	-	-	-
diarrhoea		-	-	-	-	-	-	-	-	-	-
lethargy		-	-	-	-	-	-	-	-	-	-
sleep or coma		-	-	-	-	-	-	-	-	-	-
other(specify)		-	-	-	-	-	-	-	-	-	-
**DAY 21**											
general appearance		-	-	-	-	-	-	-	-	-	-
skin and fur		-	-	-	-	-	-	-	-	-	-
eyes/mucous membranes		-	-	-	-	-	-	-	-	-	-
respiratory system		-	-	-	-	-	-	-	-	-	-
somatomotor activity		-	-	-	-	-	-	-	-	-	-
behaviour pattern		-	-	-	-	-	-	-	-	-	-
tremors and convulsions		-	-	-	-	-	-	-	-	-	-
salivation		-	-	-	-	-	-	-	-	-	-
diarrhoea		-	-	-	-	-	-	-	-	-	-
lethargy		-	-	-	-	-	-	-	-	-	-
sleep or coma		-	-	-	-	-	-	-	-	-	-
other(specify)		-	-	-	-	-	-	-	-	-	-
**DAY 22**											
general appearance		-	-	-	-	-	-	-	-	-	-
skin and fur		-	-	-	-	-	-	-	-	-	-
eyes/mucous membranes		-	-	-	-	-	-	-	-	-	-
respiratory system		-	-	-	-	-	-	-	-	-	-
somatomotor activity		-	-	-	-	-	-	-	-	-	-
behaviour pattern		-	-	-	-	-	-	-	-	-	-
tremors and convulsions		-	-	-	-	-	-	-	-	-	-
salivation		-	-	-	-	-	-	-	-	-	-
diarrhoea		-	-	-	-	-	-	-	-	-	-
lethargy		-	-	-	-	-	-	-	-	-	-
sleep or coma		-	-	-	-	-	-	-	-	-	-
other(specify)		-	-	-	-	-	-	-	-	-	-
**DAY 23**											
general appearance		-	-	-	-	-	-	-	-	-	-
skin and fur		-	-	-	-	-	-	-	-	-	-
eyes/mucous membranes		-	-	-	-	-	-	-	-	-	-
respiratory system		-	-	-	-	-	-	-	-	-	-
somatomotor activity		-	-	-	-	-	-	-	-	-	-
behaviour pattern		-	-	-	-	-	-	-	-	-	-
tremors and convulsions		-	-	-	-	-	-	-	-	-	-
salivation		-	-	-	-	-	-	-	-	-	-
diarrhoea		-	-	-	-	-	-	-	-	-	-
lethargy		-	-	-	-	-	-	-	-	-	-
sleep or coma		-	-	-	-	-	-	-	-	-	-
other(specify)		-	-	-	-	-	-	-	-	-	-
**DAY 24**											
general appearance		-	-	-	-	-	-	-	-	-	-
skin and fur		-	-	-	-	-	-	-	-	-	-
eyes/mucous membranes		-	-	-	-	-	-	-	-	-	-
respiratory system		-	-	-	-	-	-	-	-	-	-
somatomotor activity		-	-	-	-	-	-	-	-	-	-
behaviour pattern		-	-	-	-	-	-	-	-	-	-
tremors and convulsions		-	-	-	-	-	-	-	-	-	-
salivation		-	-	-	-	-	-	-	-	-	-
diarrhoea		-	-	-	-	-	-	-	-	-	-
lethargy		-	-	-	-	-	-	-	-	-	-
sleep or coma		-	-	-	-	-	-	-	-	-	-
other(specify)		-	-	-	-	-	-	-	-	-	-

ENTERED BY: [illegible] 10/2/12

CHECKED BY: [illegible] 20/2/12

ICPQN1035.B

**LAB-028/f1 & LAB-028/F2 INDIVIDUAL CLINICAL OBSERVATIONS - DAILY**	GROUP	3	3	3	3	3	3	3	3	3	3
	Treatment	TI/458	TI/458	TI/458	TI/458	TI/458	TI/458	TI/458	TI/458	TI/458	TI/458
	Dose (mg/kg)	20	20	20	20	20	20	20	20	20	20
**Note:** -, No abnormalities;	Animal ID	R2429	R2430	R2431	R2432	R2433	R2434	R2435	R2436	R2437	R2438
P- porphyrin discharge around eye	Sex	M	M	M	M	M	M	M	M	M	M
**DAY 25**											
general appearance		-	-	-	-	-	-	-	-	-	-
skin and fur		-	-	-	-	-	-	-	-	-	-
eyes/mucous membranes		-	-	-	-	-	-	-	-	-	-
respiratory system		-	-	-	-	-	-	-	-	-	-
somatomotor activity		-	-	-	-	-	-	-	-	-	-
behaviour pattern		-	-	-	-	-	-	-	-	-	-
tremors and convulsions		-	-	-	-	-	-	-	-	-	-
salivation		-	-	-	-	-	-	-	-	-	-
diarrhoea		-	-	-	-	-	-	-	-	-	-
lethargy		-	-	-	-	-	-	-	-	-	-
sleep or coma		-	-	-	-	-	-	-	-	-	-
other(specify)		-	-	-	-	-	-	-	-	-	-
**DAY 26**											
general appearance		-	-	-	-	-	-	-	-	-	-
skin and fur		-	-	-	-	-	-	-	-	-	-
eyes/mucous membranes		-	-	-	-	-	-	-	-	-	-
respiratory system		-	-	-	-	-	-	-	-	-	-
somatomotor activity		-	-	-	-	-	-	-	-	-	-
behaviour pattern		-	-	-	-	-	-	-	-	-	-
tremors and convulsions		-	-	-	-	-	-	-	-	-	-
salivation		-	-	-	-	-	-	-	-	-	-
diarrhoea		-	-	-	-	-	-	-	-	-	-
lethargy		-	-	-	-	-	-	-	-	-	-
sleep or coma		-	-	-	-	-	-	-	-	-	-
other(specify)		-	-	-	-	-	-	-	-	-	-
**DAY 27**											
general appearance		-	-	-	-	-	-	-	-	-	-
skin and fur		-	-	-	-	-	-	-	-	-	-
eyes/mucous membranes		-	-	-	-	-	-	-	-	-	-
respiratory system		-	-	-	-	-	-	-	-	-	-
somatomotor activity		-	-	-	-	-	-	-	-	-	-
behaviour pattern		-	-	-	-	-	-	-	-	-	-
tremors and convulsions		-	-	-	-	-	-	-	-	-	-
salivation		-	-	-	-	-	-	-	-	-	-
diarrhoea		-	-	-	-	-	-	-	-	-	-
lethargy		-	-	-	-	-	-	-	-	-	-
sleep or coma		-	-	-	-	-	-	-	-	-	-
other(specify)		-	-	-	-	-	-	-	-	-	-
**DAY 28**											
general appearance		-	-	-	-	-	-	-	-	-	-
skin and fur		-	-	-	-	-	-	-	-	-	-
eyes/mucous membranes		-	-	-	-	-	-	-	-	-	-
respiratory system		-	-	-	-	-	-	-	-	-	-
somatomotor activity		-	-	-	-	-	-	-	-	-	-
behaviour pattern		-	-	-	-	-	-	-	-	-	-
tremors and convulsions		-	-	-	-	-	-	-	-	-	-
salivation		-	-	-	-	-	-	-	-	-	-
diarrhoea		-	-	-	-	-	-	-	-	-	-
lethargy		-	-	-	-	-	-	-	-	-	-
sleep or coma		-	-	-	-	-	-	-	-	-	-
other(specify)		-	-	-	-	-	-	-	-	-	-
**DAY 29**											
general appearance		-	-	-	-	-	-	-	-	-	-
skin and fur		-	-	-	-	-	-	-	-	-	-
eyes/mucous membranes		-	-	-	-	-	-	-	-	-	-
respiratory system		-	-	-	-	-	-	-	-	-	-
somatomotor activity		-	-	-	-	-	-	-	-	-	-
behaviour pattern		-	-	-	-	-	-	-	-	-	-
tremors and convulsions		-	-	-	-	-	-	-	-	-	-
salivation		-	-	-	-	-	-	-	-	-	-
diarrhoea		-	-	-	-	-	-	-	-	-	-
lethargy		-	-	-	-	-	-	-	-	-	-
sleep or coma		-	-	-	-	-	-	-	-	-	-
other(specify)		-	-	-	-	-	-	-	-	-	-

ENTERED BY: [illegible] 10/2/12

CHECKED BY: [illegible] 20/2/12

ICPQN1035.B

**LAB-028/f1 & LAB-028/F2 INDIVIDUAL CLINICAL OBSERVATIONS - DAILY**											
	GROUP	4	4	4	4	4	5	5	5	5	5
	Treatment	Vehicle	Vehicle	Vehicle	Vehicle	Vehicle	TI/458	TI/458	TI/458	TI/458	TI/458
	Dose (mg/kg)						20	20	20	20	20
Note: -, No abnormalities;	Animal ID	R2439	R2440	R2441	R2442	R2443	R2444	R2445	R2446	R2447	R2448
P- porphyrin discharge around eye	Sex	M	M	M	M	M	M	M	M	M	F
**DAY 1**											
general appearance		-	-	-	-	-	-	-	-	-	-
skin and fur		-	-	-	-	-	-	-	-	-	-
eyes/mucous membranes		-	-	-	-	-	-	-	-	-	-
respiratory system		-	-	-	-	-	-	-	-	-	-
somatomotor activity		-	-	-	-	-	-	-	-	-	-
behaviour pattern		-	-	-	-	-	-	-	-	-	-
tremors and convulsions		-	-	-	-	-	-	-	-	-	-
salivation		-	-	-	-	-	-	-	-	-	-
diarrhoea		-	-	-	-	-	-	-	-	-	-
lethargy		-	-	-	-	-	-	-	-	-	-
sleep or coma		-	-	-	-	-	-	-	-	-	-
other(specify)		-	-	-	-	-	-	-	-	-	-
**DAY 2**											
general appearance		-	-	-	-	-	-	-	-	-	-
skin and fur		-	-	-	-	-	-	-	-	-	-
eyes/mucous membranes		-	-	-	-	-	-	-	-	-	-
respiratory system		-	-	-	-	-	-	-	-	-	-
somatomotor activity		-	-	-	-	-	-	-	-	-	-
behaviour pattern		-	-	-	-	-	-	-	-	-	-
tremors and convulsions		-	-	-	-	-	-	-	-	-	-
salivation		-	-	-	-	-	-	-	-	-	-
diarrhoea		-	-	-	-	-	-	-	-	-	-
lethargy		-	-	-	-	-	-	-	-	-	-
sleep or coma		-	-	-	-	-	-	-	-	-	-
other(specify)		-	-	-	-	-	-	-	-	-	-
**DAY 3**											
general appearance		-	-	-	-	-	-	-	-	-	-
skin and fur		-	-	-	-	-	-	-	-	-	-
eyes/mucous membranes		-	-	-	-	-	-	-	-	-	-
respiratory system		-	-	-	-	-	-	-	-	-	-
somatomotor activity		-	-	-	-	-	-	-	-	-	-
behaviour pattern		-	-	-	-	-	-	-	-	-	-
tremors and convulsions		-	-	-	-	-	-	-	-	-	-
salivation		-	-	-	-	-	-	-	-	-	-
diarrhoea		-	-	-	-	-	-	-	-	-	-
lethargy		-	-	-	-	-	-	-	-	-	-
sleep or coma		-	-	-	-	-	-	-	-	-	-
other(specify)		-	-	-	-	-	-	-	-	-	-
**DAY 4**											
general appearance		-	-	-	-	-	-	-	-	-	-
skin and fur		-	-	-	-	-	-	-	-	-	-
eyes/mucous membranes		-	-	-	-	-	-	-	-	-	-
respiratory system		-	-	-	-	-	-	-	-	-	-
somatomotor activity		-	-	-	-	-	-	-	-	-	-
behaviour pattern		-	-	-	-	-	-	-	-	-	-
tremors and convulsions		-	-	-	-	-	-	-	-	-	-
salivation		-	-	-	-	-	-	-	-	-	-
diarrhoea		-	-	-	-	-	-	-	-	-	-
lethargy		-	-	-	-	-	-	-	-	-	-
sleep or coma		-	-	-	-	-	-	-	-	-	-
other(specify)		-	-	-	-	-	-	-	-	-	-
**DAY 5**											
general appearance		-	-	-	-	-	-	-	-	-	-
skin and fur		-	-	-	-	-	-	-	-	-	-
eyes/mucous membranes		-	-	-	-	-	-	-	-	-	-
respiratory system		-	-	-	-	-	-	-	-	-	-
somatomotor activity		-	-	-	-	-	-	-	-	-	-
behaviour pattern		-	-	-	-	-	-	-	-	-	-
tremors and convulsions		-	-	-	-	-	-	-	-	-	-
salivation		-	-	-	-	-	-	-	-	-	-
diarrhoea		-	-	-	-	-	-	-	-	-	-
lethargy		-	-	-	-	-	-	-	-	-	-
sleep or coma		-	-	-	-	-	-	-	-	-	-
other(specify)		-	-	-	-	-	-	-	-	-	-
**DAY 6**											
general appearance		-	-	-	-	-	-	-	-	-	-
skin and fur		-	-	-	-	-	-	-	-	-	-
eyes/mucous membranes		-	-	-	-	-	-	-	-	-	-
respiratory system		-	-	-	-	-	-	-	-	-	-
somatomotor activity		-	-	-	-	-	-	-	-	-	-
behaviour pattern		-	-	-	-	-	-	-	-	-	-
tremors and convulsions		-	-	-	-	-	-	-	-	-	-
salivation		-	-	-	-	-	-	-	-	-	-
diarrhoea		-	-	-	-	-	-	-	-	-	-
lethargy		-	-	-	-	-	-	-	-	-	-
sleep or coma		-	-	-	-	-	-	-	-	-	-
other(specify)		-	-	-	-	-	-	-	-	-	-

ENTERED BY: E2 10/2/12

CHECKED BY: S 20/2/12

ICPQN1035.B

**LAB-028/f1 & LAB-028/F2 INDIVIDUAL CLINICAL OBSERVATIONS - DAILY**	GROUP	4	4	4	4	4	5	5	5	5	5
	Treatment	Vehicle	Vehicle	Vehicle	Vehicle	Vehicle	TI/458	TI/458	TI/458	TI/458	TI/458
	Dose (mg/kg)						20	20	20	20	20
**Note:** -, No abnormalities;	Animal ID	R2439	R2440	R2441	R2442	R2443	R2444	R2445	R2446	R2447	R2448
P- porphyrin discharge around eye	Sex	M	M	M	M	M	M	M	M	M	F
**DAY 7**											
general appearance		-	-	-	-	-	-	-	-	-	-
skin and fur		-	-	-	-	-	-	-	-	-	-
eyes/mucous membranes		-	-	-	-	-	-	-	-	-	-
respiratory system		-	-	-	-	-	-	-	-	-	-
somatomotor activity		-	-	-	-	-	-	-	-	-	-
behaviour pattern		-	-	-	-	-	-	-	-	-	-
tremors and convulsions		-	-	-	-	-	-	-	-	-	-
salivation		-	-	-	-	-	-	-	-	-	-
diarrhoea		-	-	-	-	-	-	-	-	-	-
lethargy		-	-	-	-	-	-	-	-	-	-
sleep or coma		-	-	-	-	-	-	-	-	-	-
other(specify)		-	-	-	-	-	-	-	-	-	-
**DAY 8**											
general appearance		-	-	-	-	-	-	-	-	-	-
skin and fur		-	-	-	-	-	-	-	-	-	-
eyes/mucous membranes		-	-	-	-	-	-	-	-	-	-
respiratory system		-	-	-	-	-	-	-	-	-	-
somatomotor activity		-	-	-	-	-	-	-	-	-	-
behaviour pattern		-	-	-	-	-	-	-	-	-	-
tremors and convulsions		-	-	-	-	-	-	-	-	-	-
salivation		-	-	-	-	-	-	-	-	-	-
diarrhoea		-	-	-	-	-	-	-	-	-	-
lethargy		-	-	-	-	-	-	-	-	-	-
sleep or coma		-	-	-	-	-	-	-	-	-	-
other(specify)		-	-	-	-	-	-	-	-	-	-
**DAY 9**											
general appearance		-	-	-	-	-	-	-	-	-	-
skin and fur		-	-	-	-	-	-	-	-	-	-
eyes/mucous membranes		-	-	-	-	-	-	-	-	-	-
respiratory system		-	-	-	-	-	-	-	-	-	-
somatomotor activity		-	-	-	-	-	-	-	-	-	-
behaviour pattern		-	-	-	-	-	-	-	-	-	-
tremors and convulsions		-	-	-	-	-	-	-	-	-	-
salivation		-	-	-	-	-	-	-	-	-	-
diarrhoea		-	-	-	-	-	-	-	-	-	-
lethargy		-	-	-	-	-	-	-	-	-	-
sleep or coma		-	-	-	-	-	-	-	-	-	-
other(specify)		-	-	-	-	-	-	-	-	-	-
**DAY 10**											
general appearance		-	-	-	-	-	-	-	-	-	-
skin and fur		-	-	-	-	-	-	-	-	-	-
eyes/mucous membranes		-	-	-	-	-	-	-	-	-	-
respiratory system		-	-	-	-	-	-	-	-	-	-
somatomotor activity		-	-	-	-	-	-	-	-	-	-
behaviour pattern		-	-	-	-	-	-	-	-	-	-
tremors and convulsions		-	-	-	-	-	-	-	-	-	-
salivation		-	-	-	-	-	-	-	-	-	-
diarrhoea		-	-	-	-	-	-	-	-	-	-
lethargy		-	-	-	-	-	-	-	-	-	-
sleep or coma		-	-	-	-	-	-	-	-	-	-
other(specify)		-	-	-	-	-	-	-	-	-	-
**DAY 11**											
general appearance		-	-	-	-	-	-	-	-	-	-
skin and fur		-	-	-	-	-	-	-	-	-	-
eyes/mucous membranes		-	-	-	-	-	-	-	-	-	-
respiratory system		-	-	-	-	-	-	-	-	-	-
somatomotor activity		-	-	-	-	-	-	-	-	-	-
behaviour pattern		-	-	-	-	-	-	-	-	-	-
tremors and convulsions		-	-	-	-	-	-	-	-	-	-
salivation		-	-	-	-	-	-	-	-	-	-
diarrhoea		-	-	-	-	-	-	-	-	-	-
lethargy		-	-	-	-	-	-	-	-	-	-
sleep or coma		-	-	-	-	-	-	-	-	-	-
other(specify)		-	-	-	-	-	-	-	-	-	-
**DAY 12**											
general appearance		-	-	-	-	-	-	-	-	-	-
skin and fur		-	-	-	-	-	-	-	-	-	-
eyes/mucous membranes		-	-	-	-	-	-	-	-	-	-
respiratory system		-	-	-	-	-	-	-	-	-	-
somatomotor activity		-	-	-	-	-	-	-	-	-	-
behaviour pattern		-	-	-	-	-	-	-	-	-	-
tremors and convulsions		-	-	-	-	-	-	-	-	-	-
salivation		-	-	-	-	-	-	-	-	-	-
diarrhoea		-	-	-	-	-	-	-	-	-	-
lethargy		-	-	-	-	-	-	-	-	-	-
sleep or coma		-	-	-	-	-	-	-	-	-	-
other(specify)		-	-	-	-	-	-	-	-	-	-

ENTERED BY: 82 10/2/12

CHECKED BY: [illegible] 20/2/12

ICPQN1035.B

**LAB-028/f1 & LAB-028/F2 INDIVIDUAL CLINICAL OBSERVATIONS - DAILY**	GROUP	4	4	4	4	4	5	5	5	5	5
	Treatment	Vehicle	Vehicle	Vehicle	Vehicle	Vehicle	TI/458	TI/458	TI/458	TI/458	TI/458
	Dose (mg/kg)						20	20	20	20	20
**Note:** -. No abnormalities;	Animal ID	R2439	R2440	R2441	R2442	R2443	R2444	R2445	R2446	R2447	R2448
P- porphyrin discharge around eye	Sex	M	M	M	M	M	M	M	M	M	F
**DAY 13**											
general appearance		-	-	-	-	-	-	-	-	-	-
skin and fur		-	-	-	-	-	-	-	-	-	-
eyes/mucous membranes		-	-	-	-	-	-	-	-	-	-
respiratory system		-	-	-	-	-	-	-	-	-	-
somatomotor activity		-	-	-	-	-	-	-	-	-	-
behaviour pattern		-	-	-	-	-	-	-	-	-	-
tremors and convulsions		-	-	-	-	-	-	-	-	-	-
salivation		-	-	-	-	-	-	-	-	-	-
diarrhoea		-	-	-	-	-	-	-	-	-	-
lethargy		-	-	-	-	-	-	-	-	-	-
sleep or coma		-	-	-	-	-	-	-	-	-	-
other(specify)		-	-	-	-	-	-	-	-	-	-
**DAY 14**											
general appearance		-	-	-	-	-	-	-	-	-	-
skin and fur		-	-	-	-	-	-	-	-	-	-
eyes/mucous membranes		-	-	-	-	-	-	-	-	-	-
respiratory system		-	-	-	-	-	-	-	-	-	-
somatomotor activity		-	-	-	-	-	-	-	-	-	-
behaviour pattern		-	-	-	-	-	-	-	-	-	-
tremors and convulsions		-	-	-	-	-	-	-	-	-	-
salivation		-	-	-	-	-	-	-	-	-	-
diarrhoea		-	-	-	-	-	-	-	-	-	-
lethargy		-	-	-	-	-	-	-	-	-	-
sleep or coma		-	-	-	-	-	-	-	-	-	-
other(specify)		-	-	-	-	-	-	-	-	-	-
**DAY 15**											
general appearance		-	-	-	-	-	-	-	-	-	-
skin and fur		-	-	-	-	-	-	-	-	-	-
eyes/mucous membranes		-	-	-	-	-	-	-	-	-	-
respiratory system		-	-	-	-	-	-	-	-	-	-
somatomotor activity		-	-	-	-	-	-	-	-	-	-
behaviour pattern		-	-	-	-	-	-	-	-	-	-
tremors and convulsions		-	-	-	-	-	-	-	-	-	-
salivation		-	-	-	-	-	-	-	-	-	-
diarrhoea		-	-	-	-	-	-	-	-	-	-
lethargy		-	-	-	-	-	-	-	-	-	-
sleep or coma		-	-	-	-	-	-	-	-	-	-
other(specify)		-	-	-	-	-	-	-	-	-	-
**DAY 16**											
general appearance		-	-	-	-	-	-	-	-	-	-
skin and fur		-	-	-	-	-	-	-	-	-	-
eyes/mucous membranes		-	-	-	-	-	-	-	-	-	-
respiratory system		-	-	-	-	-	-	-	-	-	-
somatomotor activity		-	-	-	-	-	-	-	-	-	-
behaviour pattern		-	-	-	-	-	-	-	-	-	-
tremors and convulsions		-	-	-	-	-	-	-	-	-	-
salivation		-	-	-	-	-	-	-	-	-	-
diarrhoea		-	-	-	-	-	-	-	-	-	-
lethargy		-	-	-	-	-	-	-	-	-	-
sleep or coma		-	-	-	-	-	-	-	-	-	-
other(specify)		-	-	-	-	-	-	-	-	-	-
**DAY 17**											
general appearance		-	-	-	-	-	-	-	-	-	-
skin and fur		-	-	-	-	-	-	-	-	-	-
eyes/mucous membranes		-	-	-	-	-	-	-	-	-	-
respiratory system		-	-	-	-	-	-	-	-	-	-
somatomotor activity		-	-	-	-	-	-	-	-	-	-
behaviour pattern		-	-	-	-	-	-	-	-	-	-
tremors and convulsions		-	-	-	-	-	-	-	-	-	-
salivation		-	-	-	-	-	-	-	-	-	-
diarrhoea		-	-	-	-	-	-	-	-	-	-
lethargy		-	-	-	-	-	-	-	-	-	-
sleep or coma		-	-	-	-	-	-	-	-	-	-
other(specify)		-	-	-	-	-	-	-	-	-	-
**DAY 18**											
general appearance		-	-	-	-	-	-	-	-	-	-
skin and fur		-	-	-	-	-	-	-	-	-	-
eyes/mucous membranes		-	-	-	-	-	-	-	-	-	-
respiratory system		-	-	-	-	-	-	-	-	-	-
somatomotor activity		-	-	-	-	-	-	-	-	-	-
behaviour pattern		-	-	-	-	-	-	-	-	-	-
tremors and convulsions		-	-	-	-	-	-	-	-	-	-
salivation		-	-	-	-	-	-	-	-	-	-
diarrhoea		-	-	-	-	-	-	-	-	-	-
lethargy		-	-	-	-	-	-	-	-	-	-
sleep or coma		-	-	-	-	-	-	-	-	-	-
other(specify)		-	-	-	-	-	-	-	-	-	-

ENTERED BY: [illegible] 20/2/12

CHECKED BY: [illegible] 20/2/12

ICPQN1035.B

**LAB-028/f1 & LAB-028/F2 INDIVIDUAL CLINICAL OBSERVATIONS - DAILY**	GROUP	4	4	4	4	4	5	5	5	5	5
	Treatment	Vehicle	Vehicle	Vehicle	Vehicle	Vehicle	TI/458	TI/458	TI/458	TI/458	TI/458
	Dose (mg/kg)						20	20	20	20	20
**Note:** -, No abnormalities;	Animal ID	R2439	R2440	R2441	R2442	R2443	R2444	R2445	R2446	R2447	R2448
P- porphyrin discharge around eye	Sex	M	M	M	M	M	M	M	M	M	F
**DAY 19**											
general appearance		-	-	-	-	-	-	-	-	-	-
skin and fur		-	-	-	-	-	-	-	-	-	-
eyes/mucous membranes		-	-	-	-	-	-	-	-	-	-
respiratory system		-	-	-	-	-	-	-	-	-	-
somatomotor activity		-	-	-	-	-	-	-	-	-	-
behaviour pattern		-	-	-	-	-	-	-	-	-	-
tremors and convulsions		-	-	-	-	-	-	-	-	-	-
salivation		-	-	-	-	-	-	-	-	-	-
diarrhoea		-	-	-	-	-	-	-	-	-	-
lethargy		-	-	-	-	-	-	-	-	-	-
sleep or coma		-	-	-	-	-	-	-	-	-	-
other(specify)		-	-	-	-	-	-	-	-	-	-
**DAY 20**											
general appearance		-	-	-	-	-	-	-	-	-	-
skin and fur		-	-	-	-	-	-	-	-	-	-
eyes/mucous membranes		-	-	-	-	-	-	-	-	-	-
respiratory system		-	-	-	-	-	-	-	-	-	-
somatomotor activity		-	-	-	-	-	-	-	-	-	-
behaviour pattern		-	-	-	-	-	-	-	-	-	-
tremors and convulsions		-	-	-	-	-	-	-	-	-	-
salivation		-	-	-	-	-	-	-	-	-	-
diarrhoea		-	-	-	-	-	-	-	-	-	-
lethargy		-	-	-	-	-	-	-	-	-	-
sleep or coma		-	-	-	-	-	-	-	-	-	-
other(specify)		-	-	-	-	-	-	-	-	-	-
**DAY 21**											
general appearance		-	-	-	-	-	-	-	-	-	-
skin and fur		-	-	-	-	-	-	-	-	-	-
eyes/mucous membranes		-	-	-	-	-	-	-	-	-	-
respiratory system		-	-	-	-	-	-	-	-	-	-
somatomotor activity		-	-	-	-	-	-	-	-	-	-
behaviour pattern		-	-	-	-	-	-	-	-	-	-
tremors and convulsions		-	-	-	-	-	-	-	-	-	-
salivation		-	-	-	-	-	-	-	-	-	-
diarrhoea		-	-	-	-	-	-	-	-	-	-
lethargy		-	-	-	-	-	-	-	-	-	-
sleep or coma		-	-	-	-	-	-	-	-	-	-
other(specify)		-	-	-	-	-	-	-	-	-	-
**DAY 22**											
general appearance		-	-	-	-	-	-	-	-	-	-
skin and fur		-	-	-	-	-	-	-	-	-	-
eyes/mucous membranes		-	-	-	-	-	-	-	-	-	-
respiratory system		-	-	-	-	-	-	-	-	-	-
somatomotor activity		-	-	-	-	-	-	-	-	-	-
behaviour pattern		-	-	-	-	-	-	-	-	-	-
tremors and convulsions		-	-	-	-	-	-	-	-	-	-
salivation		-	-	-	-	-	-	-	-	-	-
diarrhoea		-	-	-	-	-	-	-	-	-	-
lethargy		-	-	-	-	-	-	-	-	-	-
sleep or coma		-	-	-	-	-	-	-	-	-	-
other(specify)		-	-	-	-	-	-	-	-	-	-
**DAY 23**											
general appearance		-	-	-	-	-	-	-	-	-	-
skin and fur		-	-	-	-	-	-	-	-	-	-
eyes/mucous membranes		-	-	-	-	-	-	-	-	-	-
respiratory system		-	-	-	-	-	-	-	-	-	-
somatomotor activity		-	-	-	-	-	-	-	-	-	-
behaviour pattern		-	-	-	-	-	-	-	-	-	-
tremors and convulsions		-	-	-	-	-	-	-	-	-	-
salivation		-	-	-	-	-	-	-	-	-	-
diarrhoea		-	-	-	-	-	-	-	-	-	-
lethargy		-	-	-	-	-	-	-	-	-	-
sleep or coma		-	-	-	-	-	-	-	-	-	-
other(specify)		-	-	-	-	-	-	-	-	-	-
**DAY 24**											
general appearance		-	-	-	-	-	-	-	-	-	-
skin and fur		-	-	-	-	-	-	-	-	-	-
eyes/mucous membranes		-	-	-	-	-	-	-	-	-	-
respiratory system		-	-	-	-	-	-	-	-	-	-
somatomotor activity		-	-	-	-	-	-	-	-	-	-
behaviour pattern		-	-	-	-	-	-	-	-	-	-
tremors and convulsions		-	-	-	-	-	-	-	-	-	-
salivation		-	-	-	-	-	-	-	-	-	-
diarrhoea		-	-	-	-	-	-	-	-	-	-
lethargy		-	-	-	-	-	-	-	-	-	-
sleep or coma		-	-	-	-	-	-	-	-	-	-
other(specify)		-	-	-	-	-	-	-	-	-	-

ENTERED BY: BZ 19/2/12
CHECKED BY: [illegible] 20/2/12

ICPQN1035.B

**LAB-028/f1 & LAB-028/F2 INDIVIDUAL CLINICAL OBSERVATIONS - DAILY**	GROUP	4	4	4	4	4	5	5	5	5	5
	Treatment	Vehicle	Vehicle	Vehicle	Vehicle	Vehicle	TI/458	TI/458	TI/458	TI/458	TI/458
	Dose (mg/kg)						20	20	20	20	20
**Note:** -, No abnormalities;	Animal ID	R2439	R2440	R2441	R2442	R2443	R2444	R2445	R2446	R2447	R2448
P- porphyrin discharge around eye	Sex	M	M	M	M	M	M	M	M	M	F
**DAY 25**											
general appearance		-	-	-	-	-	-	-	-	-	-
skin and fur		-	-	-	-	-	-	-	-	-	-
eyes/mucous membranes		-	-	-	-	-	-	-	-	-	-
respiratory system		-	-	-	-	-	-	-	-	-	-
somatomotor activity		-	-	-	-	-	-	-	-	-	-
behaviour pattern		-	-	-	-	-	-	-	-	-	-
tremors and convulsions		-	-	-	-	-	-	-	-	-	-
salivation		-	-	-	-	-	-	-	-	-	-
diarrhoea		-	-	-	-	-	-	-	-	-	-
lethargy		-	-	-	-	-	-	-	-	-	-
sleep or coma		-	-	-	-	-	-	-	-	-	-
other(specify)		-	-	-	-	-	-	-	-	-	-
**DAY 26**											
general appearance		-	-	-	-	-	-	-	-	-	-
skin and fur		-	-	-	-	-	-	-	-	-	-
eyes/mucous membranes		-	-	-	-	-	-	-	-	-	-
respiratory system		-	-	-	-	-	-	-	-	-	-
somatomotor activity		-	-	-	-	-	-	-	-	-	-
behaviour pattern		-	-	-	-	-	-	-	-	-	-
tremors and convulsions		-	-	-	-	-	-	-	-	-	-
salivation		-	-	-	-	-	-	-	-	-	-
diarrhoea		-	-	-	-	-	-	-	-	-	-
lethargy		-	-	-	-	-	-	-	-	-	-
sleep or coma		-	-	-	-	-	-	-	-	-	-
other(specify)		-	-	-	-	-	-	-	-	-	-
**DAY 27**											
general appearance		-	-	-	-	-	-	-	-	-	-
skin and fur		-	-	-	-	-	-	-	-	-	-
eyes/mucous membranes		-	-	-	-	-	-	-	-	-	-
respiratory system		-	-	-	-	-	-	-	-	-	-
somatomotor activity		-	-	-	-	-	-	-	-	-	-
behaviour pattern		-	-	-	-	-	-	-	-	-	-
tremors and convulsions		-	-	-	-	-	-	-	-	-	-
salivation		-	-	-	-	-	-	-	-	-	-
diarrhoea		-	-	-	-	-	-	-	-	-	-
lethargy		-	-	-	-	-	-	-	-	-	-
sleep or coma		-	-	-	-	-	-	-	-	-	-
other(specify)		-	-	-	-	-	-	-	-	-	-
**DAY 28**											
general appearance		-	-	-	-	-	-	-	-	-	-
skin and fur		-	-	-	-	-	-	-	-	-	-
eyes/mucous membranes		-	-	-	-	-	-	-	-	-	-
respiratory system		-	-	-	-	-	-	-	-	-	-
somatomotor activity		-	-	-	-	-	-	-	-	-	-
behaviour pattern		-	-	-	-	-	-	-	-	-	-
tremors and convulsions		-	-	-	-	-	-	-	-	-	-
salivation		-	-	-	-	-	-	-	-	-	-
diarrhoea		-	-	-	-	-	-	-	-	-	-
lethargy		-	-	-	-	-	-	-	-	-	-
sleep or coma		-	-	-	-	-	-	-	-	-	-
other(specify)		-	-	-	-	-	-	-	-	-	-
**DAY 29**											
general appearance		-	-	-	-	-	-	-	-	-	-
skin and fur		-	-	-	-	-	-	-	-	-	-
eyes/mucous membranes		-	-	-	-	-	-	-	-	-	-
respiratory system		-	-	-	-	-	-	-	-	-	-
somatomotor activity		-	-	-	-	-	-	-	-	-	-
behaviour pattern		-	-	-	-	-	-	-	-	-	-
tremors and convulsions		-	-	-	-	-	-	-	-	-	-
salivation		-	-	-	-	-	-	-	-	-	-
diarrhoea		-	-	-	-	-	-	-	-	-	-
lethargy		-	-	-	-	-	-	-	-	-	-
sleep or coma		-	-	-	-	-	-	-	-	-	-
other(specify)		-	-	-	-	-	-	-	-	-	-

ENTERED BY: SZ 10/2/12
CHECKED BY: [illegible] 20/2/12

BODY WEIGHT (g) / % WEIGHT GAIN
MAIN RECOVERY COMBINED

ICPQN1035.B

LAB-027/f2

Animals	Sex	Dose (mg/kg)	Body Weight (g) on Receipt	Dose Volume D1-7 (mL)	D8-14 (mL)	D15-21 (mL)	D22-27 (mL)	D28 (mL)	Weight (g) D1	D8	D15	D22	D28
**GROUPS 1 & 4**													
R2409	M	Vehicle	204	2.6	3.2	3.7	4.1	4.4	256	314	366	402	430
R2410	M	Vehicle	202	2.6	3.3	3.7	4.2	4.3	256	321	364	406	425
R2411	M	Vehicle	205	2.7	3.3	3.8	4.2	4.5	265	319	371	410	438
R2412	M	Vehicle	203	2.7	3.2	3.5	3.9	4.1	262	309	344	377	397
R2413	M	Vehicle	190	2.5	3.1	3.5	3.9	4.1	248	303	343	383	400
R2414	M	Vehicle	200	2.7	3.2	3.7	4.0	4.3	263	315	362	395	418
R2415	M	Vehicle	202	2.9	3.5	4.0	4.2	4.5	279	342	387	415	442
R2416	M	Vehicle	201	2.7	3.3	3.8	4.1	4.4	260	321	368	402	426
R2417	M	Vehicle	197	2.7	3.3	3.7	3.9	4.2	262	320	361	386	408
R2418	M	Vehicle	189	2.5	3.1	3.6	3.8	3.9	246	299	350	367	386
R2439	M	Vehicle	202	2.6	3.3	3.9	4.4	4.8	257	324	382	430	472
R2440	M	Vehicle	207	2.6	3.1	3.6	4.0	4.3	256	307	353	390	419
R2441	M	Vehicle	203	2.6	3.2	3.7	4.1	4.4	256	310	360	399	427
R2442	M	Vehicle	192	2.4	3.0	3.4	3.7	3.9	238	297	332	359	377
R2443	M	Vehicle	212	2.7	3.4	4.0	4.6	4.9	265	335	390	445	476
Mean M			201						258	316	362	398	423
STDEV			6.3						9.5	12.4	16.4	22.6	27.8
**GROUP 2**													
R2419	M	1	191	2.4	2.9	3.2	3.6	3.7	239	287	322	352	367
R2420	M	1	196	2.5	3.0	3.4	3.8	3.9	248	301	340	372	391
R2421	M	1	203	2.6	3.2	3.7	4.2	4.3	254	315	367	414	427
R2422	M	1	210	2.7	3.2	3.7	4.2	4.4	269	316	370	412	432
R2423	M	1	200	2.6	3.1	3.5	3.9	4.0	255	305	350	382	399
R2424	M	1	211	2.8	3.4	3.9	4.2	4.7	276	337	391	419	461
R2425	M	1	209	2.7	3.2	3.7	3.9	3.9	269	321	363	390	382
R2426	M	1	202	2.7	3.2	3.5	3.7	3.9	271	313	342	364	385
R2427	M	1	192	2.5	3.0	3.3	3.6	3.8	249	297	330	360	378
R2428	M	1	207	2.8	3.3	3.8	4.1	4.4	282	331	380	409	436
Mean M			202						261	312	356	387	406
STDEV			7.3						14.0	15.3	22.3	24.9	31.0
t-test													
**GROUPS 3 & 5**													
R2429	M	20	200	2.5	2.9	3.1	3.4	3.5	242	280	300	335	346
R2430	M	20	197	2.5	3.0	3.4	3.8	4.1	240	293	331	374	400
R2431	M	20	195	2.5	3.0	3.4	3.8	4.0	240	296	334	372	391
R2432	M	20	203	2.6	3.1	3.5	3.7	3.9	252	301	340	366	386
R2433	M	20	200	2.6	3.2	3.7	4.2	4.4	254	312	361	412	429
R2434	M	20	195	2.7	3.2	3.7	4.1	4.3	263	317	360	403	422
R2435	M	20	201	2.7	3.2	3.7	4.1	4.4	260	311	358	400	427
R2436	M	20	209	2.8	3.3	3.7	3.9	4.1	271	325	361	381	397
R2437	M	20	199	2.7	3.4	3.9	4.5	4.8	266	330	382	438	465
R2438	M	20	200	2.8	3.3	3.8	4.1	4.3	274	326	369	402	425
R2444	M	20	200	2.6	3.2	3.6	4.0	4.3	250	311	356	390	418
R2445	M	20	211	2.7	3.3	3.9	4.3	4.4	265	324	380	416	433
R2446	M	20	213	2.8	3.3	3.8	4.1	4.3	269	325	368	403	420
R2447	M	20	199	2.5	3.2	3.6	4.1	4.5	249	309	356	403	435
R2448	M	20	200	2.6	3.2	3.6	4.0	4.3	254	314	352	390	416
Mean M			201						257	312	354	392	414
STDEV			5.4						11.3	14.1	20.8	24.5	27.3
t-test													

ENTERED BY: ER 10/2/12

CHECKED BY: JS 29/2/12

BODY WEIGHT (g) / % WEIGHT GAIN
MAIN RECOVERY COMBINED

ICPQN103.

LAB-027/f2

Animals	Sex	%Wt gain D1-8	D8-15	D15-22	D22-28	D1-28
**GROUPS 1 & 4**						
R2409	M	22.7	16.6	9.8	7.0	68.0
R2410	M	25.4	13.4	11.5	4.7	66.0
R2411	M	20.4	16.3	10.5	6.8	65.3
R2412	M	17.9	11.3	9.6	5.3	51.5
R2413	M	22.2	13.2	11.7	4.4	61.3
R2414	M	19.8	14.9	9.1	5.8	58.9
R2415	M	22.6	13.2	7.2	6.5	58.4
R2416	M	23.5	14.6	9.2	6.0	63.8
R2417	M	22.1	12.8	6.9	5.7	55.7
R2418	M	20.6	17.1	4.9	5.2	55.6
R2439	M	26.1	17.9	12.6	9.8	83.7
R2440	M	19.9	15.0	10.5	7.4	63.7
R2441	M	21.1	16.1	10.8	7.0	66.8
R2442	M	24.8	11.8	8.1	5.0	58.4
R2443	M	26.4	16.4	14.1	7.0	79.6
Mean M		22.4	14.7	9.8	6.2	63.8
STDEV		2.50	2.01	2.35	1.35	8.65
**GROUP 2**						
R2419	M	20.1	12.2	9.3	4.3	53.6
R2420	M	21.4	13.0	9.4	5.1	57.7
R2421	M	24.0	16.5	12.8	3.1	68.1
R2422	M	17.5	17.1	11.4	4.9	60.6
R2423	M	19.6	14.8	9.1	4.5	56.5
R2424	M	22.1	16.0	7.2	10.0	67.0
R2425	M	19.3	13.1	7.4	-2.1	42.0
R2426	M	15.5	9.3	6.4	5.8	42.1
R2427	M	19.3	11.1	9.1	5.0	51.8
R2428	M	17.4	14.8	7.6	6.6	54.6
Mean M		19.6	13.8	9.0	4.7	55.4
STDEV		2.49	2.51	1.96	3.0	8.84
t-test		***0.0141***	0.3424	0.3687	0.1602	***0.0299***
**GROUPS 3 & 5**						
R2429	M	15.7	7.1	11.7	3.3	43.0
R2430	M	22.1	13.0	13.0	7.0	66.7
R2431	M	23.3	12.8	11.4	5.1	62.9
R2432	M	19.4	13.0	7.6	5.5	53.2
R2433	M	22.8	15.7	14.1	4.1	68.9
R2434	M	20.5	13.6	11.9	4.7	60.5
R2435	M	19.6	15.1	11.7	6.8	64.2
R2436	M	19.9	11.1	5.5	4.2	46.5
R2437	M	24.1	15.8	14.7	6.2	74.8
R2438	M	19.0	13.2	8.9	5.7	55.1
R2444	M	24.4	14.5	9.6	7.2	67.2
R2445	M	22.3	17.3	9.5	4.1	63.4
R2446	M	20.8	13.2	9.5	4.2	56.1
R2447	M	24.1	15.2	13.2	7.9	74.7
R2448	M	23.6	12.1	10.8	6.7	63.8
Mean M		21.4	13.5	10.9	5.5	61.4
STDEV		2.45	2.39	2.47	1.39	9.23
t-test		0.3231	0.1491	0.2218	0.1543	0.4702

ENTERED BY: ER 10/2/12
CHECKED BY: [illegible] 29/2/12

BODY WEIGHT (g) / % WEIGHT GAIN
RECOVERY GROUPS

ICPQN1035.B

LAB-027/f2 Animals	Sex	Dose (mg/kg)	Weight (g) D1	D28	D36	D42	%Wt gain D28-36	D36-42	D1-42
**GROUP 4**									
R2439	M	Vehicle	257	472	509	548	7.8	7.7	113.2
R2440	M	Vehicle	256	419	442	458	5.5	3.6	78.9
R2441	M	Vehicle	256	427	456	474	6.8	3.9	85.2
R2442	M	Vehicle	238	377	399	416	5.8	4.3	74.8
R2443	M	Vehicle	265	476	509	539	6.9	5.9	103.4
Mean M			254	434	463	487	6.58	5.08	91.1
STDEV			9.9	41.0	47.0	55.8	0.935	1.689	16.51
**GROUP 5**									
R2444	M	20	250	418	449	472	7.4	5.1	88.8
R2445	M	20	265	433	466	484	7.6	3.9	82.6
R2446	M	20	269	420	460	483	9.5	5.0	79.6
R2447	M	20	249	435	471	496	8.3	5.3	99.2
R2448	M	20	254	416	442	466	6.3	5.4	83.5
Mean M			257	424	458	480	7.82	4.94	86.7
STDEV			9.1	8.9	12.0	11.6	1.202	0.627	7.72
t-test							0.1084	0.8760	0.6128

ENTERED BY: ℛ 28/2/12

CHECKED BY: ✓ 28/2/12

FEED INTAKE
MAIN RECOVERY GROUPS COMBINED

ICPQN1035.B

FEED INTAKE
LAB-024/f2

Animal No.	Dose (µg/kg)	Sex	Weight feed(g) start week 1	Weight feed(g) added week 1	Weight feed(g) end week 1	Feed intake Week 1	Weight feed(g) start week 2	Weight feed(g) added week 1	Weight feed(g) end week 2	Feed intake Week 2
**Groups 1 & 4**										
R2409-R2413	Vehicle	M	934		146	788	927		97	830
R2414-R2418	Vehicle	M	899		61	838	960		110	850
R2439-R2443	Vehicle	M	893		89	804	925		19	906
Mean/ 5 animal						810				862
STDEV						25.5				39.4
**Group 2**										
R2419-R2423	1	M	908		126	782	923		93	830
R2424-R2428	1	M	862		42	820	965		118	847
Mean/5 animal						801				839
STDEV						26.9				12.0
t-test						0.7416				0.4172
**Groups 3 & 5**										
R2429-R2433	20	M	904		136	768	927		88	839
R2434-R2438	20	M	893		15	878	935		8	927
R2444-R2448	20	M	902		90	812	928		62	866
Mean/5 animal						819				877
STDEV						55.4				45.1
t-test						0.8091				0.6807

ENTERED BY: SZ 10/2/12
CHECKED BY: [illegible] 10/2/12

FEED INTAKE
MAIN RECOVERY GROUPS COMBINED

ICPQN1035.B

FEED INTAKE
LAB-024/f2

Animal No.	Dose ($\mu g/kg$)	Sex	Weight feed(g) start week 3	Weight feed(g) added week 3	Weight feed(g) end week 3	Feed intake Week 3	Weight feed(g) start week 4	Weight feed(g) added week 4	Weight feed(g) end week 4	Feed intake Week 4	Feed Intake Week 1-4
**Groups 1 & 4**											
R2409-R2413	Vehicle	M	943		66	877	941		164	777	3272
R2414-R2418	Vehicle	M	944		88	856	940		129	811	3355
R2439-R2443	Vehicle	M	965		24	941	939		61	878	3529
Mean/ 5 animal						891				822	3385
STDEV						44.3				51.4	131.2
**Group 2**											
R2419-R2423	1	M	946		101	845	938		183	755	3212
R2424-R2428	1	M	998		153	845	931		137	794	3306
Mean/5 animal						845				775	3259
STDEV						0.00				27.6	66.5
t-test						0.2116				0.2737	0.2525
**Groups 3 & 5**											
R2429-R2433	20	M	948		57	891	941		147	794	3292
R2434-R2438	20	M	998		42	956	940		77	863	3624
R2444-R2448	20	M	969		69	900	938		151	787	3365
Mean/5 animal						916				815	3427
STDEV						35.2				42.0	174.5
t-test						0.4996				0.8579	0.7587

ENTERED BY: ꝺQ 10/2/12

CHECKED BY: [illegible] 10/2/12

FEED INTAKE
RECOVERY GROUPS

ICPQN1035.B

FEED INTAKE
LAB-024/f2

Animal No.	Dose (µg/kg)	Sex	Feed intake Week 1-4	Weight feed(g) start week 5	Weight feed(g) added week 5	Weight feed(g) end week 5	Feed intake Week 5	Weight feed(g) start week 6	Weight feed(g) added week 6	Weight feed(g) end week 6	Feed intake Week 6	Feed Intake Week 5-6	Feed Intake Week 1-6
**Group 4**													
R2439 -R2443	Vehicle	M	3529	1003	164	114	1053	1257	0	399	858	1911	5440
Mean/ 5 animal							1053				858	1911	5440
STDEV													
**Group 5**													
R2444- R2448	20	M	3365	980	183	150	1013	1171	0	369	802	1815	5180
Mean/5 animal							1013				802	1815	5180
STDEV													
t-test													

ENTERED BY: [illegible] 23/2/12

CHECKED BY: [illegible] 23/2/12

C 26/45

LAB-029/f1	GROUP	1	1	1	1	1	1	1	1	1	1	2	2	2	2	2	2	2	2	2	2
Detailed Clinical Observations	Time / Treatment	Vehicle	Vehicle	Vehicle	Vehicle	Vehicle	Vehicle	Vehicle	Vehicle	Vehicle	Vehicle	TI/458	TI/458	TI/458	TI/458	TI/458	TI/458	TI/458	TI/458	TI/458	TI/458
	Dose (mg/kg)	-	-	-	-	-	-	-	-	-	-	1	1	1	1	1	1	1	1	1	1
	Animal ID	R2409	R2410	R2411	R2412	R2413	R2414	R2415	R2416	R2417	R2418	R2419	R2420	R2421	R2422	R2423	R2424	R2425	R2426	R2427	R2428
Note: -, No abnormalities;	Sex	M	M	M	M	M	M	M	M	M	M	M	M	M	M	M	M	M	M	M	M
**POSTURE**	**PRE**	6	5	6	5	6	7	5	5	7	5	7	6	6	5	6	7	6	5	6	6
**GAIT**		1	1	1	1	1	1	1	1	1	1	1	1	1	1	1	1	1	1	1	1
**TOTAL GAIT SCORE**		1	1	1	1	1	1	1	1	1	1	1	1	1	1	1	1	1	1	1	1
**CLONIC CONVULSIONS**		1	1	1	1	1	1	1	1	1	1	1	1	1	1	1	1	1	1	1	1
**TONIC CONVULSIONS**		1	1	1	1	1	1	1	1	1	1	1	1	1	1	1	1	1	1	1	1
**BITING**		1	1	1	1	1	1	1	1	1	1	1	1	1	1	1	1	1	1	1	1
**REMOVING RAT FROM CAGE**		2	2	2	2	2	2	2	2	2	2	2	2	2	2	2	2	2	2	2	2
**HANDLING RAT IN HAND**		2	2	2	2	2	2	2	2	2	2	2	2	2	2	2	2	2	2	2	2
**LACRIMATION**		1	1	1	1	1	1	1	1	1	1	1	1	1	1	1	1	1	1	1	1
**FUR APPEARANCE:** Piloerection (Y/N)		1 N	1 N	1 N	1 N	1 N	1 N	1 N	1 N	1 N	1 N	1 N	1 N	1 N	1 N	1 N	1 N	1 N	1 N	1 N	1 N
**SALIVATION**		1	1	1	1	1	1	1	1	1	1	1	1	1	1	1	1	1	1	1	1
**DEFAECATION**		4	3	3	5	4	5	3	4	6	3	3	4	4	3	2	3	4	3	4	6
**URINATION**		1	2	2	1	2	3	1	2	2	2	2	1	1	2	3	1	3	2	2	2
**GROOMING:** Repetitive grooming		2	3	2	4	2	1	2	2	1	3	3	2	2	1	3	1	2	2	2	2
**GROOMING:** Unbalanced grooming		0	0	0	0	0	0	0	0	0	0	0	0	0	0	0	0	0	0	0	0
**PALPEBRAL CLOSURE**		1	1	1	1	1	1	1	1	1	1	1	1	1	1	1	1	1	1	1	1
**PUPIL RESPONSE**		1	1	1	1	1	1	1	1	1	1	1	1	1	1	1	1	1	1	1	1
**EYES:** Exopthalmus (Y/N)		N	N	N	N	N	N	N	N	N	N	N	N	N	N	N	N	N	N	N	N
**EYES:** Crustiness around eyes(Y/N)		N	N	N	N	N	N	N	N	N	N	N	N	N	N	N	N	N	N	N	N
**RESPIRATION**		1	1	1	1	1	1	1	1	1	1	1	1	1	1	1	1	1	1	1	1
**BEHAVIOUR:** Writhing (Y/N)		N	N	N	N	N	N	N	N	N	N	N	N	N	N	N	N	N	N	N	N
**BEHAVIOUR:** Circling (Y/N)		N	N	N	N	N	N	N	N	N	N	N	N	N	N	N	N	N	N	N	N
**BEHAVIOUR:** Vocalisations (Y/N)		N	N	N	N	N	N	N	N	N	N	N	N	N	N	N	N	N	N	N	N
**REARING:** Unsupported rears		0	1	0	0	0	0	0	0	1	0	0	1	0	0	0	0	0	0	1	0
**REARING:**Supported rears		13	17	9	16	12	18	14	12	8	16	11	16	12	13	14	8	12	19	14	23
**POSTURE**	**WEEK 1**	7	7	6	6	7	6	7	7	7	7	6	6	7	7	6	7	6	5	7	5
**GAIT**		1	1	1	1	1	1	1	1	1	1	1	1	1	1	1	1	1	1	1	1
**TOTAL GAIT SCORE**		1	1	1	1	1	1	1	1	1	1	1	1	1	1	1	1	1	1	1	1
**CLONIC CONVULSIONS**		1	1	1	1	1	1	1	1	1	1	1	1	1	1	1	1	1	1	1	1
**TONIC CONVULSIONS**		1	1	1	1	1	1	1	1	1	1	1	1	1	1	1	1	1	1	1	1
**BITING**		1	1	1	1	1	1	1	1	1	1	1	1	1	1	1	1	1	1	1	1
**REMOVING RAT FROM CAGE**		2	2	2	2	2	2	2	2	2	2	2	2	2	2	2	2	2	2	2	2
**HANDLING RAT IN HAND**		2	2	2	2	2	2	2	2	2	2	2	2	2	2	2	2	2	2	2	2
**LACRIMATION**		1	1	1	1	1	1	1	1	1	1	1	1	1	1	1	1	1	1	1	1
**FUR APPEARANCE:** Piloerection (Y/N)		1 N	1 N	1 N	1 N	1 N	1 N	1 N	1 N	1 N	1 N	1 N	1 N	1 N	1 N	1 N	1 N	1 N	1 N	1 N	1 N
**SALIVATION**		1	1	1	1	1	1	1	1	1	1	1	1	1	1	1	1	1	1	1	1
**DEFAECATION**		3	5	5	2	2	5	0	2	3	1	4	6	5	6	5	5	4	5	7	0
**URINATION**		5	1	0	0	1	1	0	2	2	0	0	1	1	0	2	1	0	2	3	0
**GROOMING :** Repetitive grooming		1	0	0	1	1	1	0	1	0	2	1	2	1	2	2	3	0	1	1	0
**GROOMING:** Unbalanced grooming		0	0	0	0	0	0	0	0	0	0	0	0	0	0	0	0	0	0	0	0
**PALPEBRAL CLOSURE**		1	1	1	1	1	1	1	1	1	1	1	1	1	1	1	1	1	1	1	1
**PUPIL RESPONSE**		1	1	1	1	1	1	1	1	1	1	1	1	1	1	1	1	1	1	1	1
**EYES:** Exopthalmus (Y/N)		N	N	N	N	N	N	N	N	N	N	N	N	N	N	N	N	N	N	N	N
**EYES:** Crustiness around eyes(Y/N)		N	N	N	N	N	N	N	N	N	N	N	N	N	N	N	N	N	N	N	N
**RESPIRATION**		1	1	1	1	1	1	1	1	1	1	1	1	1	1	1	1	1	1	1	1
**BEHAVIOUR:** Writhing (Y/N)		N	N	N	N	N	N	N	N	N	N	N	N	N	N	N	N	N	N	N	N
**BEHAVIOUR:** Circling (Y/N)		N	N	N	N	N	N	N	N	N	N	N	N	N	N	N	N	N	N	N	N
**BEHAVIOUR:** Vocalisations (Y/N)		N	N	N	N	N	N	N	N	N	N	N	N	N	N	N	N	N	N	N	N
**REARING:** Unsupported rears		0	0	0	0	0	0	2	0	0	1	0	0	0	0	0	0	0	0	0	1
**REARING:** Supported rears		8	9	10	8	10	14	16	9	6	7	8	7	9	8	5	6	18	11	11	12

Entered by: SR 14/2/12

Checked by: d 20/2/12

C 27/45

ICPQN1035.B

LAB-029/f1	GROUP	1	1	1	1	1	1	1	1	1	1	2	2	2	2	2	2	2	2	2	2
Detailed Clinical Observations	Time Treatment	Vehicle	Vehicle	Vehicle	Vehicle	Vehicle	Vehicle	Vehicle	Vehicle	Vehicle	Vehicle	TI/458	TI/458	TI/458	TI/458	TI/458	TI/458	TI/458	TI/458	TI/458	TI/458
	Dose (mg/kg)	-	-	-	-	-	-	-	-	-	-	1	1	1	1	1	1	1	1	1	1
	Animal ID	R2409	R2410	R2411	R2412	R2413	R2414	R2415	R2416	R2417	R2418	R2419	R2420	R2421	R2422	R2423	R2424	R2425	R2426	R2427	R2428
Note: -, No abnormalities;	Sex	M	M	M	M	M	M	M	M	M	M	M	M	M	M	M	M	M	M	M	M
**POSTURE**	**WEEK 2**	6	7	7	6	7	7	7	7	7	6	7	7	5	7	7	6	7	6	7	7
**GAIT**		1	1	1	1	1	1	1	1	1	1	1	1	1	1	1	1	1	1	1	1
**TOTAL GAIT SCORE**		1	1	1	1	1	1	1	1	1	1	1	1	1	1	1	1	1	1	1	1
**CLONIC CONVULSIONS**		1	1	1	1	1	1	1	1	1	1	1	1	1	1	1	1	1	1	1	1
**TONIC CONVULSIONS**		1	1	1	1	1	1	1	1	1	1	1	1	1	1	1	1	1	1	1	1
**BITING**		1	1	1	1	1	1	1	1	1	1	1	1	1	1	1	1	1	1	1	1
**REMOVING RAT FROM CAGE**		1	2	2	1	1	1	2	2	2	2	1	2	1	2	2	1	2	2	2	2
**HANDLING RAT IN HAND**		2	2	2	2	2	2	2	2	2	2	2	2	2	2	2	2	2	2	2	2
**LACRIMATION**		1	1	1	1	1	1	1	1	1	1	1	1	1	1	1	1	1	1	1	1
**FUR APPEARANCE:** Piloerection (Y/N)		1 N	1 N	1 N	1 N	1 N	1 N	1 N	1 N	1 N	1 N	1 N	1 N	1 N	1 N	1 N	1 N	1 N	1 N	1 N	1 N
**SALIVATION**		1	1	1	1	1	1	1	1	1	1	1	1	1	1	1	1	1	1	1	1
**DEFAECATION**		3	2	1	3	1	3	1	2	2	2	3	3	3	4	3	3	2	3	3	2
**URINATION**		2	1	2	1	1	2	1	3	3	1	1	1	2	2	3	1	2	3	4	1
**GROOMING :** Repetitive grooming		1	1	1	2	2	2	1	2	1	2	2	3	1	2	3	2	2	1	2	1
**GROOMING:** Unbalanced grooming		0	0	0	0	0	0	0	0	0	0	0	0	0	0	0	0	0	0	0	0
**PALPEBRAL CLOSURE**		1	1	1	1	1	1	1	1	1	1	1	1	1	1	1	1	1	1	1	1
**PUPIL RESPONSE**		1	1	1	1	1	1	1	1	1	1	1	1	1	1	1	1	1	1	1	1
**EYES:** Exopthalmus (Y/N)		N	N	N	N	N	N	N	N	N	N	N	N	N	N	N	N	N	N	N	N
**EYES:** Crustiness around eyes(Y/N)		N	N	N	N	N	N	N	N	N	N	N	N	N	N	N	N	N	N	N	N
**RESPIRATION**		1	1	1	1	1	1	1	1	1	1	1	1	1	1	1	1	1	1	1	1
**BEHAVIOUR:** Writhing (Y/N)		N	N	N	N	N	N	N	N	N	N	N	N	N	N	N	N	N	N	N	N
**BEHAVIOUR:** Circling (Y/N)		N	N	N	N	N	N	N	N	N	N	N	N	N	N	N	N	N	N	N	N
**BEHAVIOUR:** Vocalisations (Y/N)		N	N	N	N	N	N	N	N	N	N	N	N	N	N	N	N	N	N	N	N
**REARING:** Unsupported rears		0	0	0	0	0	0	1	0	0	0	0	0	0	0	0	0	0	0	0	0
**REARING:** Supported rears		11	8	12	11	9	12	14	8	9	11	9	12	8	11	8	9	14	14	9	17
**POSTURE**	**WEEK 3**	6	6	6	7	5	5	6	6	6	6	6	7	6	6	6	6	6	6	6	6
**GAIT**		1	1	1	1	1	1	1	1	1	1	1	1	1	1	1	1	1	1	1	1
**TOTAL GAIT SCORE**		1	1	1	1	1	1	1	1	1	1	1	1	1	1	1	1	1	1	1	1
**CLONIC CONVULSIONS**		1	1	1	1	1	1	1	1	1	1	1	1	1	1	1	1	1	1	1	1
**TONIC CONVULSIONS**		1	1	1	1	1	1	1	1	1	1	1	1	1	1	1	1	1	1	1	1
**BITING**		1	1	1	1	1	1	1	1	1	1	1	1	1	1	1	1	1	1	1	1
**REMOVING RAT FROM CAGE**		1	1	1	2	1	1	1	2	1	2	1	1	1	1	1	1	1	1	1	1
**HANDLING RAT IN HAND**		2	2	2	2	2	2	2	2	2	2	2	2	2	2	2	2	2	2	2	2
**LACRIMATION**		1	1	1	1	1	1	1	1	1	1	1	1	1	1	1	1	1	1	1	1
**FUR APPEARANCE:** Piloerection (Y/N)		1 N	1 N	1 N	1 N	1 N	1 N	1 N	1 N	1 N	1 N	1 N	1 N	1 N	1 N	1 N	1 N	1 N	1 N	1 N	1 N
**SALIVATION**		1	1	1	1	1	1	1	1	1	1	1	1	1	1	1	1	1	1	1	1
**DEFAECATION**		2	3	1	4	3	3	1	3	2	3	4	2	0	1	1	3	3	0	2	1
**URINATION**		1	2	1	2	2	2	2	0	2	2	2	1	0	3	0	0	2	0	0	0
**GROOMING :** Repetitive grooming		2	2	1	2	3	2	2	2	2	2	2	2	2	1	2	1	2	2	2	2
**GROOMING:** Unbalanced grooming		0	0	0	0	0	0	0	0	0	0	0	0	0	0	0	0	0	0	0	0
**PALPEBRAL CLOSURE**		1	1	1	1	1	1	1	1	1	1	1	1	1	1	1	1	1	1	1	1
**PUPIL RESPONSE**		1	1	1	1	1	1	1	1	1	1	1	1	1	1	1	1	1	1	1	1
**EYES:** Exopthalmus (Y/N)		N	N	N	N	N	N	N	N	N	N	N	N	N	N	N	N	N	N	N	N
**EYES:** Crustiness around eyes(Y/N)		N	N	N	N	N	N	N	N	N	N	N	N	N	N	N	N	N	N	N	N
**RESPIRATION**		1	1	1	1	1	1	1	1	1	1	1	1	1	1	1	1	1	1	1	1
**BEHAVIOUR:** Writhing (Y/N)		N	N	N	N	N	N	N	N	N	N	N	N	N	N	N	N	N	N	N	N
**BEHAVIOUR:** Circling (Y/N)		N	N	N	N	N	N	N	N	N	N	N	N	N	N	N	N	N	N	N	N
**BEHAVIOUR:** Vocalisations (Y/N)		N	N	N	N	N	N	N	N	N	N	N	N	N	N	N	N	N	N	N	N
**REARING:** Unsupported rears		0	0	0	0	0	0	1	0	0	0	0	0	0	0	0	0	0	0	0	0
**REARING:** Supported rears		9	12	14	10	13	16	13	14	12	14	12	9	10	12	12	4	16	12	11	15

Entered by: 82 14/2/12

Checked by: [illegible] 20/2/12

C 28/45

ICPQN1035.B

LAB-029/f1	GROUP	1	1	1	1	1	1	1	1	1	1	2	2	2	2	2	2	2	2	2	2
**Detailed Clinical Observations**	**Time** Treatment Dose (mg/kg)	Vehicle -	Vehicle -	Vehicle -	Vehicle -	Vehicle -	Vehicle -	Vehicle -	Vehicle -	Vehicle -	Vehicle -	TI/458 1	TI/458 1	TI/458 1	TI/458 1	TI/458 1	TI/458 1	TI/458 1	TI/458 1	TI/458 1	TI/458 1
	Animal ID	R2409	R2410	R2411	R2412	R2413	R2414	R2415	R2416	R2417	R2418	R2419	R2420	R2421	R2422	R2423	R2424	R2425	R2426	R2427	R2428
**Note:** -, No abnormalities;	Sex	M	M	M	M	M	M	M	M	M	M	M	M	M	M	M	M	M	M	M	M
**POSTURE**	**WEEK 4**	7	6	7	6	6	6	7	6	5	6	5	6	7	7	7	5	7	7	6	6
**GAIT**		1	1	1	1	1	1	1	1	1	1	1	1	1	1	1	1	1	1	1	1
**TOTAL GAIT SCORE**		1	1	1	1	1	1	1	1	1	1	1	1	1	1	1	1	1	1	1	1
**CLONIC CONVULSIONS**		1	1	1	1	1	1	1	1	1	1	1	1	1	1	1	1	1	1	1	1
**TONIC CONVULSIONS**		1	1	1	1	1	1	1	1	1	1	1	1	1	1	1	1	1	1	1	1
**BITING**		1	1	1	1	1	1	1	1	1	1	1	1	1	1	1	1	1	1	1	1
**REMOVING RAT FROM CAGE**		1	1	1	1	1	1	1	1	1	1	1	1	1	1	1	1	1	1	1	1
**HANDLING RAT IN HAND**		2	2	2	2	2	2	2	2	2	2	2	2	2	2	2	2	2	2	2	2
**LACRIMATION**		1	1	1	1	1	1	1	1	1	1	1	1	1	1	1	1	1	1	1	1
**FUR APPEARANCE:** Piloerection (Y/N)		1 N	1 N	1 N	1 N	1 N	1 N	1 N	1 N	1 N	1 N	1 N	1 N	1 N	1 N	1 N	1 N	1 N	1 N	1 N	1 N
**SALIVATION**		1	1	1	1	1	1	1	1	1	1	1	1	1	1	1	1	1	1	1	1
**DEFAECATION**		3	0	3	2	0	0	4	0	1	1	1	0	4	1	2	1	2	0	0	4
**URINATION**		1	0	2	0	2	2	2	0	1	0	1	0	1	1	1	2	0	1	0	2
**GROOMING :** Repetitive grooming		2	3	2	2	2	3	2	2	3	2	2	3	2	1	1	2	2	3	2	3
**GROOMING:** Unbalanced grooming		0	0	0	0	0	0	0	0	0	0	0	0	0	0	0	0	0	0	0	0
**PALPEBRAL CLOSURE**		1	1	1	1	1	1	1	1	1	1	1	1	1	1	1	1	1	1	1	1
**PUPIL RESPONSE**		1	1	1	1	1	1	1	1	1	1	1	1	1	1	1	1	1	1	1	1
**EYES:** Exopthalmus (Y/N)		N	N	N	N	N	N	N	N	N	N	N	N	N	N	N	N	N	N	N	N
**EYES:** Crustiness around eyes(Y/N)		N	N	N	N	N	N	N	N	N	N	N	N	N	N	N	N	N	N	N	N
**RESPIRATION**		1	1	1	1	1	1	1	1	1	1	1	1	1	1	1	1	1	1	1	1
**BEHAVIOUR:** Writhing (Y/N)		N	N	N	N	N	N	N	N	N	N	N	N	N	N	N	N	N	N	N	N
**BEHAVIOUR:** Circling (Y/N)		N	N	N	N	N	N	N	N	N	N	N	N	N	N	N	N	N	N	N	N
**BEHAVIOUR:** Vocalisations (Y/N)		N	N	N	N	N	N	N	N	N	N	N	N	N	N	N	N	N	N	N	N
**REARING:** Unsupported rears		0	1	1	0	0	0	2	0	0	2	0	0	0	0	0	0	0	1	0	0
**REARING:** Supported rears		14	10	12	8	11	11	9	13	11	10	10	13	13	9	9	11	14	9	12	13

Entered by: BR 14/2/12

Checked by: DS 20/2/12

C 29/45

ICPQN1035.B

LAB-029/f1		GROUP	3	3	3	3	3	3	3	3	3	3	4	4	4	4	4	5	5	5	5	5
Detailed Clinical Observations	Time	Treatment Dose (mg/kg)	TI/458 20	TI/458 20	TI/458 20	TI/458 20	TI/458 20	TI/458 20	TI/458 20	TI/458 20	TI/458 20	TI/458 20	Vehicle	Vehicle	Vehicle	Vehicle	Vehicle	TI/458 20	TI/458 20	TI/458 20	TI/458 20	TI/458 20
		Animal ID	R2429	R2430	R2431	R2432	R2433	R2434	R2435	R2436	R2437	R2438	R2439	R2440	R2441	R2442	R2443	R2444	R2445	R2446	R2447	R2448
Note: -, No abnormalities;		Sex	M	M	M	M	M	M	M	M	M	M	M	M	M	M	M	M	M	M	M	M
POSTURE	PRE		6	6	7	5	6	5	6	6	6	6	5	5	6	6	5	6	6	5	7	6
GAIT			1	1	1	1	1	1	1	1	1	1	1	1	1	1	1	1	1	1	1	1
TOTAL GAIT SCORE			1	1	1	1	1	1	1	1	1	1	1	1	1	1	1	1	1	1	1	1
CLONIC CONVULSIONS			1	1	1	1	1	1	1	1	1	1	1	1	1	1	1	1	1	1	1	1
TONIC CONVULSIONS			1	1	1	1	1	1	1	1	1	1	1	1	1	1	1	1	1	1	1	1
BITING			1	1	1	1	1	1	1	1	1	1	1	1	1	1	1	1	1	1	1	1
REMOVING RAT FROM CAGE			2	2	2	2	2	2	2	2	2	2	2	2	2	2	2	2	2	2	2	2
HANDLING RAT IN HAND			2	2	2	2	2	2	2	2	2	2	2	2	2	2	2	2	2	2	2	2
LACRIMATION			1	1	1	1	1	1	1	1	1	1	1	1	1	1	1	1	1	1	1	1
FUR APPEARANCE: Piloerection (Y/N)			1 N	1 N	1 N	1 N	1 N	1 N	1 N	1 N	1 N	1 N	1 N	1 N	1 N	1 N	1 N	1 N	1 N	1 N	1 N	1 N
SALIVATION			1	1	1	1	1	1	1	1	1	1	1	1	1	1	1	1	1	1	1	1
DEFAECATION			5	3	5	3	6	3	6	5	3	4	2	3	1	4	4	3	2	4	2	3
URINATION			1	2	2	1	2	2	2	1	1	1	2	1	0	1	0	2	1	1	1	0
GROOMING: Repetitive grooming			1	2	2	1	1	0	3	1	2	3	2	2	1	1	1	2	1	2	1	1
GROOMING: Unbalanced grooming			0	0	0	0	0	0	0	0	1	0	0	0	0	0	0	0	0	0	0	0
PALPEBRAL CLOSURE			1	1	1	1	1	1	1	1	1	1	1	1	1	1	1	1	1	1	1	1
PUPIL RESPONSE			1	1	1	1	1	1	1	1	1	1	1	1	1	1	1	1	1	1	1	1
EYES: Exopthalmus (Y/N)			N	N	N	N	N	N	N	N	N	N	N	N	N	N	N	N	N	N	N	N
EYES: Crustiness around eyes(Y/N)			N	N	N	N	N	N	N	N	N	N	N	N	N	N	N	N	N	N	N	N
RESPIRATION			1	1	1	1	1	1	1	1	1	1	1	1	1	1	1	1	1	1	1	1
BEHAVIOUR: Writhing (Y/N)			N	N	N	N	N	N	N	N	N	N	N	N	N	N	N	N	N	N	N	N
BEHAVIOUR: Circling (Y/N)			N	N	N	N	N	N	N	N	N	N	N	N	N	N	N	N	N	N	N	N
BEHAVIOUR: Vocalisations (Y/N)			N	N	N	N	N	N	N	N	N	N	N	N	N	N	N	N	N	N	N	N
REARING: Unsupported rears			0	0	1	0	0	0	2	0	0	0	2	0	0	0	1	0	0	0	0	0
REARING:Supported rears			22	17	16	16	12	10	18	12	13	16	12	15	8	12	16	19	6	14	18	12
POSTURE	WEEK 1		6	7	7	6	6	6	7	6	6	7	6	7	6	7	6	6	7	7	7	6
GAIT			1	1	1	1	1	1	1	1	1	1	1	1	1	1	1	1	1	1	1	1
TOTAL GAIT SCORE			1	1	1	1	1	1	1	1	1	1	1	1	1	1	1	1	1	1	1	1
CLONIC CONVULSIONS			1	1	1	1	1	1	1	1	1	1	1	1	1	1	1	1	1	1	1	1
TONIC CONVULSIONS			1	1	1	1	1	1	1	1	1	1	1	1	1	1	1	1	1	1	1	1
BITING			1	1	1	1	1	1	1	1	1	1	1	1	1	1	1	1	1	1	1	1
REMOVING RAT FROM CAGE			2	2	2	2	2	2	2	2	2	2	2	2	2	2	2	2	2	2	2	2
HANDLING RAT IN HAND			2	2	2	2	2	2	2	2	2	2	2	2	2	2	2	2	2	2	2	2
LACRIMATION			1	1	1	1	1	1	1	1	1	1	1	1	1	1	1	1	1	1	1	1
FUR APPEARANCE: Piloerection (Y/N)			1 N	1 N	1 N	1 N	1 N	1 N	1 N	1 N	1 N	1 N	1 N	1 N	1 N	1 N	1 N	1 N	1 N	1 N	1 N	1 N
SALIVATION			1	1	1	1	1	1	1	1	1	1	1	1	1	1	1	1	1	1	1	1
DEFAECATION			6	6	5	7	5	8	2	0	5	0	4	1	5	0	6	5	6	3	6	1
URINATION			5	0	0	2	1	2	1	1	1	0	1	1	1	0	1	0	0	3	3	2
GROOMING : Repetitive grooming			3	1	2	1	0	2	0	0	0	2	4	1	2	0	1	1	1	1	3	2
GROOMING: Unbalanced grooming			0	0	0	0	0	0	0	0	0	0	0	0	0	0	0	0	0	0	0	0
PALPEBRAL CLOSURE			1	1	1	1	1	1	1	1	1	1	1	1	1	1	1	1	1	1	1	1
PUPIL RESPONSE			1	1	1	1	1	1	1	1	1	1	1	1	1	1	1	1	1	1	1	1
EYES: Exopthalmus (Y/N)			N	N	N	N	N	N	N	N	N	N	N	N	N	N	N	N	N	N	N	N
EYES: Crustiness around eyes(Y/N)			N	N	N	N	N	N	N	N	N	N	N	N	N	N	N	N	N	N	N	N
RESPIRATION			1	1	1	1	1	1	1	1	1	1	1	1	1	1	1	1	1	1	1	1
BEHAVIOUR: Writhing (Y/N)			N	N	N	N	N	N	N	N	N	N	N	N	N	N	N	N	N	N	N	N
BEHAVIOUR: Circling (Y/N)			N	N	N	N	N	N	N	N	N	N	N	N	N	N	N	N	N	N	N	N
BEHAVIOUR: Vocalisations (Y/N)			N	N	N	N	N	N	N	N	N	N	N	N	N	N	N	N	N	N	N	N
REARING: Unsupported rears			0	0	1	0	0	0	0	0	0	0	2	0	0	0	1	2	0	0	0	0
REARING: Supported rears			6	10	9	13	8	8	17	9	8	9	9	11	12	11	18	15	4	11	17	10

Entered by: 82 14/2/12

Checked by: [illegible] 20/2/12

C 30/45

LAB-029/f1 — Detailed Clinical Observations (Note: -, No abnormalities;)																				
GROUP	3	3	3	3	3	3	3	3	3	3	4	4	4	4	4	5	5	5	5	5
Time / Treatment Dose (mg/kg)	TI/458 20	TI/458 20	TI/458 20	TI/458 20	TI/458 20	TI/458 20	TI/458 20	TI/458 20	TI/458 20	TI/458 20	Vehicle	Vehicle	Vehicle	Vehicle	Vehicle	TI/458 20	TI/458 20	TI/458 20	TI/458 20	TI/458 20
Animal ID	R2429	R2430	R2431	R2432	R2433	R2434	R2435	R2436	R2437	R2438	R2439	R2440	R2441	R2442	R2443	R2444	R2445	R2446	R2447	R2448
Sex	M	M	M	M	M	M	M	M	M	M	M	M	M	M	M	M	M	M	M	M
**WEEK 2**																				
**POSTURE**	5	7	6	7	7	7	6	7	6	7	7	6	7	7	7	7	6	6	6	7
**GAIT**	1	1	1	1	1	1	1	1	1	1	1	1	1	1	1	1	1	1	1	1
**TOTAL GAIT SCORE**	1	1	1	1	1	1	1	1	1	1	1	1	1	1	1	1	1	1	1	1
**CLONIC CONVULSIONS**	1	1	1	1	1	1	1	1	1	1	1	1	1	1	1	1	1	1	1	1
**TONIC CONVULSIONS**	1	1	1	1	1	1	1	1	1	1	1	1	1	1	1	1	1	1	1	1
**BITING**	1	1	1	1	1	1	1	1	1	1	1	1	1	1	1	1	1	1	1	1
**REMOVING RAT FROM CAGE**	1	2	2	2	2	1	2	2	2	2	2	2	2	2	2	2	1	2	2	2
**HANDLING RAT IN HAND**	2	2	2	2	2	2	2	2	2	2	2	2	2	2	2	2	2	2	2	2
**LACRIMATION**	1	1	1	1	1	1	1	1	1	1	1	1	1	1	1	1	1	1	1	1
**FUR APPEARANCE:** Piloerection (Y/N)	1 N	1 N	1 N	1 N	1 N	1 N	1 N	1 N	1 N	1 N	1 N	1 N	1 N	1 N	1 N	1 N	1 N	1 N	1 N	1 N
**SALIVATION**	1	1	1	1	1	1	1	1	1	1	1	1	1	1	1	1	1	1	1	1
**DEFAECATION**	3	2	4	4	3	4	3	2	4	2	3	1	3	0	3	3	5	2	3	1
**URINATION**	1	1	2	3	2	2	1	1	1	2	2	1	2	0	2	2	2	2	1	2
**GROOMING :** Repetitive grooming	1	2	1	2	1	1	1	1	0	2	3	2	2	1	1	2	1	2	2	1
**GROOMING:** Unbalanced grooming	0	0	0	0	0	0	0	0	0	0	0	0	0	0	0	0	0	0	0	0
**PALPEBRAL CLOSURE**	1	1	1	1	1	1	1	1	1	1	1	1	1	1	1	1	1	1	1	1
**PUPIL RESPONSE**	1	1	1	1	1	1	1	1	1	1	1	1	1	1	1	1	1	1	1	1
**EYES:** Exopthalmus (Y/N)	N	N	N	N	N	N	N	N	N	N	N	N	N	N	N	N	N	N	N	N
**EYES:** Crustiness around eyes(Y/N)	N	N	N	N	N	N	N	N	N	N	N	N	N	N	N	N	N	N	N	N
**RESPIRATION**	1	1	1	1	1	1	1	1	1	1	1	1	1	1	1	1	1	1	1	1
**BEHAVIOUR:** Writhing (Y/N)	N	N	N	N	N	N	N	N	N	N	N	N	N	N	N	N	N	N	N	N
**BEHAVIOUR:** Circling (Y/N)	N	N	N	N	N	N	N	N	N	N	N	N	N	N	N	N	N	N	N	N
**BEHAVIOUR:** Vocalisations (Y/N)	N	N	N	N	N	N	N	N	N	N	N	N	N	N	N	N	N	N	N	N
**REARING:** Unsupported rears	0	0	0	0	0	0	0	0	0	0	1	0	0	0	0	1	0	0	0	0
**REARING:** Supported rears	5	9	11	9	9	7	14	13	7	8	8	12	14	8	15	11	7	13	12	7
**WEEK 3**																				
**POSTURE**	7	6	5	6	6	6	6	5	7	6	6	6	6	5	6	6	6	6	6	5
**GAIT**	1	1	1	1	1	1	1	1	1	1	1	1	1	1	1	1	1	1	1	1
**TOTAL GAIT SCORE**	1	1	1	1	1	1	1	1	1	1	1	1	1	1	1	1	1	1	1	1
**CLONIC CONVULSIONS**	1	1	1	1	1	1	1	1	1	1	1	1	1	1	1	1	1	1	1	1
**TONIC CONVULSIONS**	1	1	1	1	1	1	1	1	1	1	1	1	1	1	1	1	1	1	1	1
**BITING**	1	1	1	1	1	1	1	1	1	1	1	1	1	1	1	1	1	1	1	1
**REMOVING RAT FROM CAGE**	1	1	1	1	1	1	1	1	1	1	1	1	1	1	1	1	1	1	1	1
**HANDLING RAT IN HAND**	2	2	2	2	2	2	2	2	2	2	2	2	2	2	2	2	2	2	2	2
**LACRIMATION**	1	1	1	1	1	1	1	1	1	1	1	1	1	1	1	1	1	1	1	1
**FUR APPEARANCE:** Piloerection (Y/N)	1 N	1 N	1 N	1 N	1 N	1 N	1 N	1 N	1 N	1 N	1 N	1 N	1 N	1 N	1 N	1 N	1 N	1 N	1 N	1 N
**SALIVATION**	1	1	1	1	1	1	1	1	1	1	1	1	1	1	1	1	1	1	1	1
**DEFAECATION**	2	2	3	3	0	3	0	1	2	0	1	1	0	0	2	0	3	1	0	4
**URINATION**	1	2	0	0	0	2	1	1	2	0	0	3	1	0	0	1	2	0	1	1
**GROOMING :** Repetitive grooming	2	3	2	3	3	1	2	2	1	2	3	2	3	2	2	2	2	1	2	2
**GROOMING:** Unbalanced grooming	0	0	0	0	0	0	0	0	0	0	0	0	0	0	0	0	0	0	0	0
**PALPEBRAL CLOSURE**	1	1	1	1	1	1	1	1	1	1	1	1	1	1	1	1	1	1	1	1
**PUPIL RESPONSE**	1	1	1	1	1	1	1	1	1	1	1	1	1	1	1	1	1	1	1	1
**EYES:** Exopthalmus (Y/N)	N	N	N	N	N	N	N	N	N	N	N	N	N	N	N	N	N	N	N	N
**EYES:** Crustiness around eyes(Y/N)	N	N	N	N	N	N	N	N	N	N	N	N	N	N	N	N	N	N	N	N
**RESPIRATION**	1	1	1	1	1	1	1	1	1	1	1	1	1	1	1	1	1	1	1	1
**BEHAVIOUR:** Writhing (Y/N)	N	N	N	N	N	N	N	N	N	N	N	N	N	N	N	N	N	N	N	N
**BEHAVIOUR:** Circling (Y/N)	N	N	N	N	N	N	N	N	N	N	N	N	N	N	N	N	N	N	N	N
**BEHAVIOUR:** Vocalisations (Y/N)	N	N	N	N	N	N	N	N	N	N	N	N	N	N	N	N	N	N	N	N
**REARING:** Unsupported rears	0	0	1	0	0	0	0	0	1	0	2	0	0	0	1	0	0	0	0	0
**REARING:** Supported rears	8	11	8	14	10	12	11	10	9	7	11	10	10	9	12	9	9	8	10	9

Entered by: [illegible] 14/2/12

Checked by: [illegible] 20/2/12

C 31/45

ICPQN1035.B

LAB-029/f1		GROUP	3	3	3	3	3	3	3	3	3	3	4	4	4	4	4	5	5	5	5	5
**Detailed Clinical Observations**	**Time**	Treatment Dose (mg/kg)	TI/458 20	TI/458 20	TI/458 20	TI/458 20	TI/458 20	TI/458 20	TI/458 20	TI/458 20	TI/458 20	TI/458 20	Vehicle	Vehicle	Vehicle	Vehicle	Vehicle	TI/458 20	TI/458 20	TI/458 20	TI/458 20	TI/458 20
		Animal ID	R2429	R2430	R2431	R2432	R2433	R2434	R2435	R2436	R2437	R2438	R2439	R2440	R2441	R2442	R2443	R2444	R2445	R2446	R2447	R2448
**Note:** -, No abnormalities;		Sex	M	M	M	M	M	M	M	M	M	M	M	M	M	M	M	M	M	M	M	M
**POSTURE**	**WEEK 4**		6	6	6	6	6	6	7	6	5	6	6	6	7	6	7	6	6	7	7	6
**GAIT**			1	1	1	1	1	1	1	1	1	1	1	1	1	1	1	1	1	1	1	1
**TOTAL GAIT SCORE**			1	1	1	1	1	1	1	1	1	1	1	1	1	1	1	1	1	1	1	1
**CLONIC CONVULSIONS**			1	1	1	1	1	1	1	1	1	1	1	1	1	1	1	1	1	1	1	1
**TONIC CONVULSIONS**			1	1	1	1	1	1	1	1	1	1	1	1	1	1	1	1	1	1	1	1
**BITING**			1	1	1	1	1	1	1	1	1	1	1	1	1	1	1	1	1	1	1	1
**REMOVING RAT FROM CAGE**			1	1	1	1	1	1	1	1	1	1	1	1	1	1	1	1	1	1	1	1
**HANDLING RAT IN HAND**			2	2	2	2	2	2	2	2	2	2	2	2	2	2	2	2	2	2	2	2
**LACRIMATION**			1	1	1	1	1	1	1	1	1	1	1	1	1	1	1	1	1	1	1	1
**FUR APPEARANCE:** Piloerection (Y/N)			1 N	1 N	1 N	1 N	1 N	1 N	1 N	1 N	1 N	1 N	1 N	1 N	1 N	1 N	1 N	1 N	1 N	1 N	1 N	1 N
**SALIVATION**			1	1	1	1	1	1	1	1	1	1	1	1	1	1	1	1	1	1	1	1
**DEFAECATION**			3	0	2	2	2	3	2	1	0	3	4	4	2	0	3	4	2	0	2	3
**URINATION**			0	0	3	0	3	2	0	1	2	0	2	2	0	0	4	2	0	2	0	2
**GROOMING :** Repetitive grooming			2	2	1	2	2	2	1	3	2	2	3	2	2	3	3	3	3	2	1	2
**GROOMING:** Unbalanced grooming			0	0	0	0	0	0	0	0	0	0	0	0	0	0	0	0	0	0	0	0
**PALPEBRAL CLOSURE**			1	1	1	1	1	1	1	1	1	1	1	1	1	1	1	1	1	1	1	1
**PUPIL RESPONSE**			1	1	1	1	1	1	1	1	1	1	1	1	1	1	1	1	1	1	1	1
**EYES:** Exopthalmus (Y/N)			N	N	N	N	N	N	N	N	N	N	N	N	N	N	N	N	N	N	N	N
**EYES:** Crustiness around eyes(Y/N)			N	N	N	N	N	N	N	N	N	N	N	N	N	N	N	N	N	N	N	N
**RESPIRATION**			1	1	1	1	1	1	1	1	1	1	1	1	1	1	1	1	1	1	1	1
**BEHAVIOUR:** Writhing (Y/N)			N	N	N	N	N	N	N	N	N	N	N	N	N	N	N	N	N	N	N	N
**BEHAVIOUR:** Circling (Y/N)			N	N	N	N	N	N	N	N	N	N	N	N	N	N	N	N	N	N	N	N
**BEHAVIOUR:** Vocalisations (Y/N)			N	N	N	N	N	N	N	N	N	N	N	N	N	N	N	N	N	N	N	N
**REARING:** Unsupported rears			0	0	0	0	0	1	0	0	0	0	3	0	0	0	2	2	0	0	0	0
**REARING:** Supported rears			12	8	14	9	11	9	15	11	12	11	12	9	9	7	13	14	8	12	12	10

C 32/45

Entered by: [signature] 14/2/12

Checked by: [signature] 20/2/12

ICPQN1035.B

FUNCTIONAL OBSERVATIONS

FUNCTIONAL OBSERVATIONS WEEK LAB-030/f1

GROUP	1	1	1	1	1	1	1	1	1	1
Treatment	Vehicle	Vehicle	Vehicle	Vehicle	Vehicle	Vehicle	Vehicle	Vehicle	Vehicle	Vehicle
Dose (mg/kg)	-	-	-	-	-	-	-	-	-	-
Animal ID	R2409	R2410	R2411	R2412	R2413	R2414	R2415	R2416	R2417	R2418
**AROUSAL**	4	4	4	4	4	4	4	4	4	4
**FINGER SNAP**	2	2	2	2	2	2	2	2	2	2
**APPROACH RESPONSE**	2	2	2	2	2	2	2	1	2	2
**TOUCH RESPONSE**	2	3	1	2	3	1	2	3	1	2
**TAIL PINCH**	3	5	3	3	3	3	5	3	3	3
**EXTENSOR THRUST**	1	1	1	1	1	1	1	1	1	1
**LIMB ROTATION**	1	1	1	1	1	1	1	1	1	1
**CATALEPSY**	0	0	0	0	0	0	0	0	0	0
**RIGHTING REFLEX**	0	0	0	0	0	0	0	0	0	0
**DROP METHOD**	0	0	0	0	0	0	0	0	0	0
**GRIP STRENGTH TEST**										
**Forelimb**	420	400	420	380	420	520	440	460	370	440
**Forelimb**	480	440	540	460	540	390	570	530	520	580
**Forelimb**	410	510	500	500	510	400	490	590	490	520
**Hindlimb**	80	70	50	50	60	50	60	80	50	70
**Hindlimb**	100	60	60	50	50	60	50	60	60	60
**Hindlimb**	60	50	60	80	50	50	70	50	50	60
**Mean M Forelimb/animal**	437	450	487	447	490	437	500	527	460	513
**Mean M Forelimb/group**	475									
**STDEV**	32.9									
**t-test**										
**Mean M Hindlimb/animal**	80	60	57	60	53	53	60	63	53	63
**Mean M Hindimb/group**	60									
**STDEV**	7.9									
**t-test**										

GROUP	2	2	2	2	2	2	2	2	2	2
Treatment	TI/458	TI/458	TI/458	TI/458	TI/458	TI/458	TI/458	TI/458	TI/458	TI/458
Dose (mg/kg)	1	1	1	1	1	1	1	1	1	1
Animal ID	R2419	R2420	R2421	R2422	R2423	R2424	R2425	R2426	R2427	R2428
**AROUSAL**	4	4	4	4	4	4	4	4	4	4
**FINGER SNAP**	2	2	2	2	2	2	2	2	2	2
**APPROACH RESPONSE**	2	1	2	2	2	2	1	2	2	2
**TOUCH RESPONSE**	2	1	2	2	3	2	2	3	2	2
**TAIL PINCH**	3	3	5	3	3	3	5	3	3	3
**EXTENSOR THRUST**	1	1	1	1	1	1	1	1	1	1
**LIMB ROTATION**	1	1	1	1	1	1	1	1	1	1
**CATALEPSY**	0	0	0	0	0	0	0	0	0	0
**RIGHTING REFLEX**	0	0	0	0	0	0	0	0	0	0
**DROP METHOD**	0	0	0	0	0	0	0	0	0	0
**GRIP STRENGTH TEST**										
**Forelimb**	410	470	620	480	580	450	510	450	460	380
**Forelimb**	550	560	480	520	450	590	470	530	510	520
**Forelimb**	600	510	520	390	530	380	370	440	400	470
**Hindlimb**	60	80	50	60	60	50	50	60	50	60
**Hindlimb**	50	60	70	50	50	60	60	70	60	50
**Hindlimb**	50	50	60	60	60	60	70	60	70	60
**Mean M Forelimb/animal**	520	513	540	463	520	473	450	473	457	457
**Mean M Forelimb/group**	487									
**STDEV**	33.0									
**t-test**	0.4260									
**Mean M Hindlimb/animal**	53	63	60	57	57	57	60	63	60	57
**Mean M Hindimb/group**	59									
**STDEV**	3.2									
**t-test**	0.5495									

C 33/45

Enter by: ER 14/2/12

Checked by: [illegible] 14/2/12

ICPQN1035.B

FUNCTIONAL OBSERVATIONS

FUNCTIONAL OBSERVATIONS WEEK LAB-030/f1	3 TI/458 20 R2429	3 TI/458 20 R2430	3 TI/458 20 R2431	3 TI/458 20 R2432	3 TI/458 20 R2433	3 TI/458 20 R2434	3 TI/458 20 R2435	3 TI/458 20 R2436	3 TI/458 20 R2437	3 TI/458 20 R2438
**AROUSAL**	4	4	4	4	4	4	4	4	4	4
**FINGER SNAP**	2	2	2	2	2	2	2	2	2	2
**APPROACH RESPONSE**	2	2	2	2	2	2	2	2	1	2
**TOUCH RESPONSE**	2	2	3	1	2	2	3	2	2	2
**TAIL PINCH**	3	5	3	3	3	5	3	3	3	3
**EXTENSOR THRUST**	1	1	1	1	1	1	1	1	1	1
**LIMB ROTATION**	1	1	1	1	1	1	1	1	1	1
**CATALEPSY**	0	0	0	0	0	0	0	0	0	0
**RIGHTING REFLEX**	0	0	0	0	0	0	0	0	0	0
**DROP METHOD**	0	0	0	0	0	0	0	0	0	0
**GRIP STRENGTH TEST**										
**Forelimb**	380	430	410	390	470	330	410	520	390	420
**Forelimb**	450	450	480	450	550	520	470	380	530	490
**Forelimb**	470	390	500	430	620	430	490	410	580	510
**Hindlimb**	60	50	50	50	60	60	60	70	60	50
**Hindlimb**	50	60	60	60	50	70	50	60	60	60
**Hindlimb**	50	70	60	60	60	50	50	50	50	60
**Mean M Forelimb/animal**	433	423	463	423	547	427	457	437	500	473
**Mean M Forelimb/group**	458									
**STDEV**	39.9									
**t-test**	0.3315									
**Mean M Hindlimb/animal**	53	60	57	57	57	60	53	60	57	57
**Mean M Hindimb/group**	57									
**STDEV**	2.5									
**t-test**	0.2310									

FUNCTIONAL OBSERVATIONS WEEK LAB-030/f1	4 Vehicle R2439	4 Vehicle R2440	4 Vehicle R2441	4 Vehicle R2442	4 Vehicle R2443	5 TI/458 20 R2444	5 TI/458 20 R2445	5 TI/458 20 R2446	5 TI/458 20 R2447	5 TI/458 20 R2448
**AROUSAL**	4	4	4	4	4	4	4	4	4	4
**FINGER SNAP**	2	2	2	2	2	2	2	2	2	2
**APPROACH RESPONSE**	2	2	1	2	2	1	2	2	2	2
**TOUCH RESPONSE**	2	3	2	2	2	2	3	2	2	2
**TAIL PINCH**	3	3	3	5	3	3	3	3	3	3
**EXTENSOR THRUST**	1	1	1	1	1	1	1	1	1	1
**LIMB ROTATION**	1	1	1	1	1	1	1	1	1	1
**CATALEPSY**	0	0	0	0	0	0	0	0	0	0
**RIGHTING REFLEX**	0	0	0	0	0	0	0	0	0	0
**DROP METHOD**	0	0	0	0	0	0	0	0	0	0
**GRIP STRENGTH TEST**										
**Forelimb**	460	410	530	400	380	370	510	380	430	600
**Forelimb**	420	480	420	480	490	450	430	430	380	510
**Forelimb**	510	510	370	340	410	490	450	420	500	490
**Hindlimb**	100	50	80	50	60	60	60	70	70	60
**Hindlimb**	50	50	50	70	60	60	70	60	60	70
**Hindlimb**	50	60	60	60	50	50	70	50	60	50
**Mean M Forelimb/animal**	463	467	440	407	427	437	463	410	437	533
**Mean M Forelimb/group**	441					456				
**STDEV**	25.2					47.2				
**t-test**	0.0500					0.4574				
**Mean M Hindlimb/animal**	67	53	63	60	57	57	67	60	63	60
**Mean M Hindimb/group**	60					61				
**STDEV**	5.3					3.8				
**t-test**	0.9245					0.7465				

C 34/45

Enter by: ℓℓ 14/2/12

Checked by: d 14/2/12

HAEMATOLOGY

ICPQN1035.B

Animals	Dose (mg/kg)	Units Sex	Submission #	WBC x10⁹/L	RBC x10¹²/L	Hb g/L	Hct L/L	MCV fL	MCH pg	MCHC g/L	PLT x10⁹/L	MPV fL	Neut x10⁹/L	Lymph x10⁹/L	Mono x10⁹/L	Eos x10⁹/L	Baso x10⁹/L	Neut %	Lymph %	Mono %	Eos %	Baso %
**GROUP 1**																						
R2409	Vehicle	M	4324031	8.65	8.07	157	0.454	56.3	19.5	346	1217	8.3	1.16	7.30	0.11	0.08	0.00	13.4	84.4	1.3	0.9	0.0
R2410	Vehicle	M	4324032	7.18	8.10	157	0.455	56.2	19.4	345	1240	8.2	0.87	6.17	0.12	0.02	0.00	12.1	85.9	1.7	0.3	0.0
R2411	Vehicle	M	4324033	5.45	8.06	156	0.446	55.3	19.4	350	1353	8.0	0.69	4.55	0.16	0.05	0.00	12.7	83.5	2.9	0.9	0.0
R2412	Vehicle	M	4324034	7.46	8.34	159	0.458	54.9	19.1	347	1289	7.9	0.85	6.22	0.28	0.11	0.00	11.3	83.4	3.8	1.5	0.0
R2413	Vehicle	M	4324035	6.04	7.95	164	0.474	59.6	20.6	346	981	8.3	0.64	5.27	0.09	0.04	0.00	10.5	87.3	1.5	0.7	0.0
R2414	Vehicle	M	4326995	1.41	7.80	155	0.456	58.5	19.9	340	868	8.2	0.15	1.21	0.03	0.01	0.01	10.7	85.8	2.1	0.7	0.7
R2415	Vehicle	M	4326996	6.53	7.60	153	0.437	57.5	20.1	350	1270	7.9	1.49	4.85	0.13	0.06	0.00	22.8	74.3	2.0	0.9	0.0
R2416	Vehicle	M	4326997	7.70	7.54	152	0.441	58.5	20.2	345	1055	8.0	1.22	6.32	0.12	0.04	0.00	15.8	82.1	1.6	0.5	0.0
R2417	Vehicle	M	4326998	7.81	8.20	165	0.456	55.6	20.1	362	1109	8.3	1.06	6.57	0.15	0.03	0.00	13.6	84.1	1.9	0.4	0.0
R2418	Vehicle	M	4326999	6.22	8.57	166	0.475	55.4	19.4	349	1359	7.5	0.99	5.08	0.10	0.05	0.00	15.9	81.7	1.6	0.8	0.0
Mean M				6.45	8.02	158	0.455	56.8	19.8	348	1174	8.1	0.91	5.35	0.13	0.05	0.001	13.9	83.3	2.04	0.76	0.070
STDEV				2.012	0.318	5.0	0.0124	1.63	0.48	5.7	164.7	0.25	0.369	1.692	0.064	0.029	0.0032	3.66	3.59	0.760	0.337	0.2214
**GROUP 2**																						
R2419	1	M	4324036	6.45	7.93	165	0.476	60.0	20.8	347	1080	8.5	1.07	5.18	0.17	0.03	0.00	16.6	80.3	2.6	0.5	0.0
R2420	1	M	4324037	6.59	8.10	157	0.454	56.0	19.4	346	1202	8.3	0.74	5.69	0.12	0.04	0.00	11.3	86.3	1.8	0.6	0.0
R2421	1	M	4324038	7.79	8.03	153	0.452	56.3	19.1	338	1279	8.1	1.38	6.18	0.18	0.05	0.00	17.8	79.3	2.3	0.6	0.0
R2422	1	M	4324039	8.14	7.72	151	0.451	58.4	19.6	335	1373	7.8	1.55	6.30	0.24	0.04	0.01	19.1	77.4	2.9	0.5	0.1
R2423	1	M	4324040	5.43	7.87	155	0.457	58.1	19.7	339	1019	9.1	0.81	4.46	0.11	0.05	0.00	15.0	82.1	2.0	0.9	0.0
R2424	1	M	4326983	5.58	7.92	157	0.448	56.6	19.8	350	1358	7.9	0.84	4.60	0.12	0.02	0.00	15.0	82.4	2.2	0.4	0.0
R2425	1	M	4326984	5.07	7.73	151	0.435	56.3	19.5	347	1080	7.8	0.87	4.06	0.09	0.05	0.00	17.1	80.1	1.8	1.0	0.0
R2426	1	M	4326985	7.58	7.68	156	0.450	58.6	20.3	347	1061	8.2	1.72	5.68	0.12	0.06	0.00	22.7	74.9	1.6	0.8	0.0
R2427	1	M	4326986	8.74	7.97	163	0.472	59.2	20.5	345	1256	8.0	1.61	6.86	0.18	0.09	0.00	18.4	78.5	2.1	1.0	0.0
R2428	1	M	4326987	9.05	8.03	159	0.456	56.8	19.8	349	1136	7.8	2.30	6.41	0.22	0.12	0.00	25.5	70.8	2.4	1.3	0.0
Mean M				7.04	7.90	157	0.455	57.6	19.9	344	1184	8.2	1.29	5.54	0.16	0.06	0.00	17.9	79.2	2.2	0.76	0.01
STDEV				1.420	0.146	4.7	0.0117	1.41	0.53	5.1	127.7	0.41	0.510	0.937	0.050	0.030	0.003	4.02	4.27	0.40	0.288	0.032
**GROUP 3**																						
R2429	20	M	4324041	4.79	8.43	164	0.467	55.4	19.5	351	1007	8.2	0.65	3.98	0.14	0.02	0.00	13.6	83.1	2.9	0.4	0.0
R2430	20	M	4324042	6.72	8.15	156	0.445	54.6	19.1	351	1327	8.2	1.11	5.36	0.19	0.06	0.00	16.5	79.8	2.8	0.9	0.0
R2431	20	M	4324043	6.06	7.86	163	0.467	59.4	20.7	349	1179	8.9	0.98	4.89	0.13	0.06	0.00	16.2	80.7	2.1	1.0	0.0
R2432	20	M	4326993	5.43	8.25	158	0.457	55.4	19.2	346	1308	8.2	0.68	4.61	0.11	0.03	0.00	12.5	84.9	2.0	0.6	0.0
R2433	20	M	4326994	8.52	7.95	161	0.463	58.2	20.3	348	1270	8.2	1.62	6.63	0.21	0.06	0.00	19.0	77.8	2.5	0.7	0.0
R2434	20	M	4326988	4.26	7.95	158	0.444	55.8	19.9	356	1124	8.1	0.68	3.44	0.09	0.05	0.00	15.9	80.8	2.1	1.2	0.0
R2435	20	M	4326989	8.32	7.73	155	0.446	57.7	20.1	348	1166	8.0	1.52	6.56	0.16	0.08	0.00	18.3	78.8	1.9	1.0	0.0
R2436	20	M	4326990	5.81	8.03	156	0.434	54.0	19.4	359	1152	8.1	0.77	4.86	0.12	0.06	0.00	13.3	83.6	2.1	1.0	0.0
R2437	20	M	4326991	9.52	8.10	158	0.452	55.8	19.5	350	1182	7.7	1.19	7.96	0.25	0.12	0.00	12.5	83.6	2.6	1.3	0.0
R2438	20	M	4326992	5.35	8.01	158	0.448	55.9	19.7	353	1197	7.8	0.86	4.28	0.14	0.07	0.00	16.1	80.0	2.6	1.3	0.0
Mean M				6.48	8.05	159	0.452	56.2	19.7	351	1191	8.1	1.01	5.26	0.15	0.06	0.00	15.4	81.3	2.4	0.9	0.0
STDEV				1.752	0.199	3.0	0.0110	1.69	0.51	4.0	93.8	0.32	0.350	1.394	0.049	0.027	0.000	2.32	2.35	0.36	0.30	0.00

C 35/45

ENTERED BY: BR 13/2/12

CHECKED BY: A. 14/2/12

HAEMATOLOGY

ICPQN1035.B

Animals	Dose (mg/kg)	Units Sex	Submission #	WBC x10⁹/L	RBC x10¹²/L	Hb g/L	Hct L/L	MCV fL	MCH pg	MCHC g/L	PLT x10⁹/L	MPV fL	Neut x10⁹/L	Lymph x10⁹/L	Mono x10⁹/L	Eos x10⁹/L	Baso x10⁹/L	Neut %	Lymph %	Mono %	Eos %	Baso %
GROUP 4																						
R2439	Vehicle	M	4326973	4.97	8.01	151	0.426	53.2	18.9	354	1157	7.9	0.66	4.18	0.10	0.03	0.00	13.3	84.1	2.0	0.6	0.0
R2440	Vehicle	M	4326974	6.71	8.26	158	0.456	55.2	19.1	346	1177	8.4	1.04	5.43	0.18	0.06	0.00	15.5	80.9	2.7	0.9	0.0
R2441	Vehicle	M	4326975	4.81	8.27	163	0.467	56.5	19.7	349	1207	8.2	0.84	3.72	0.19	0.06	0.00	17.5	77.3	4.0	1.2	0.0
R2442	Vehicle	M	4326976	2.75	8.30	153	0.431	51.9	18.4	355	1229	7.9	0.71	1.97	0.05	0.02	0.00	25.9	71.6	1.8	0.7	0.0
R2443	Vehicle	M	4326977	2.49	8.11	151	0.431	53.1	18.6	350	1309	7.7	0.64	1.80	0.03	0.02	0.00	25.7	72.3	1.2	0.8	0.0
Mean M				4.35	8.19	155	0.442	54.0	18.9	351	1216	8.0	0.78	3.42	0.11	0.04	0.00	19.6	77.2	2.3	0.8	0.0
STDEV				1.745	0.125	5.2	0.0182	1.84	0.50	3.7	59.0	0.28	0.166	1.536	0.073	0.020	0.000	5.87	5.40	1.07	0.23	0.00
GROUP 5																						
R2444	20	M	4326978	6.21	8.45	154	0.436	51.6	18.2	353	1152	8.2	0.75	5.30	0.13	0.03	0.00	12.1	85.3	2.1	0.5	0.0
R2445	20	M	4326979	6.93	8.17	154	0.439	53.7	18.8	351	1281	7.7	0.73	6.02	0.16	0.02	0.00	10.5	86.9	2.3	0.3	0.0
R2446	20	M	4326980	6.68	7.84	156	0.446	56.9	19.9	350	1326	7.9	2.32	4.08	0.19	0.09	0.00	34.8	61.1	2.8	1.3	0.0
R2447	20	M	4326981	3.94	8.02	151	0.420	52.4	18.8	360	1193	8.1	0.84	2.99	0.07	0.04	0.00	21.3	75.9	1.8	1.0	0.0
R2448	20	M	4326982	5.86	8.13	153	0.437	53.8	18.8	350	956	7.8	1.00	4.74	0.07	0.05	0.00	17.0	80.9	1.2	0.9	0.0
Mean M				5.92	8.12	154	0.436	53.7	18.9	353	1182	7.9	1.13	4.63	0.12	0.05	0.00	19.1	78.0	2.0	0.8	0.0
STDEV				1.184	0.224	1.8	0.0096	2.02	0.62	4.2	143.7	0.21	0.675	1.160	0.054	0.027	0.000	9.73	10.38	0.59	0.40	0.00

C 36/45

ENTERED BY: ZR 22/2/12
CHECKED BY: [illegible] 22/2/12

BIOCHEMISTRY

ICPQN1035.B

Animals	Dose (mg/kg)	Units Sex	Submission #	ALT IU/L	Ca^{2+} mmol/L	CK IU/L	Cl^- mmol/L	Glu mmol/L	TP g/L	Cre mmol/L	Phos mmol/L	Urea mmol/L	Trig mmol/L	AST IU/L	Tbil umol/L	ALP IU/L	Chol mmol/L	Glob g/L	Na^+ mmol/L	ALB g/L	Gamma GT IU/L	K^+ mmol/L	Ca:P	Na:K	A:G
**GROUP 1**																									
R2409	Vehicle	M	4324031	64	2.52	189	100	8.2	61	0.041	2.22	4.9	0.20	233	1.1	167	2.51	25.5	143	35.5	0	3.8	1.1	37.6	1.39
R2410	Vehicle	M	4324032	80	2.62	137	101	9.6	60	0.038	2.30	4.9	0.76	232	2.0	257	2.12	25.9	145	34.1	0	3.8	1.1	38.2	1.32
R2411	Vehicle	M	4324033	65	2.52	117	102	8.6	61	0.039	2.27	4.2	0.56	167	1.8	295	2.08	27.1	146	33.9	0	3.6	1.1	40.6	1.25
R2412	Vehicle	M	4324034	30	2.55	91	100	10.4	62	0.039	2.23	5.1	0.47	66	2.1	186	2.26	25.6	143	36.4	1	3.8	1.1	37.6	1.42
R2413	Vehicle	M	4324035	71	2.54	150	103	9.6	62	0.042	2.37	6.1	0.31	229	1.9	155	2.15	27.1	145	34.9	0	3.9	1.1	37.2	1.29
R2414	Vehicle	M	4326995	117	2.57	412	100	10.8	63	0.046	2.62	7.1	1.32	252	1.8	286	1.42	26.1	145	36.9	0	3.8	1.0	38.2	1.41
R2415	Vehicle	M	4326996	71	2.43	163	100	9.9	60	0.042	2.30	5.4	0.48	301	2.0	256	2.01	25.1	146	34.9	0	3.6	1.1	40.6	1.39
R2416	Vehicle	M	4326997	57	2.43	118	102	10.3	59	0.039	2.01	5.8	1.02	191	1.7	265	2.01	25.9	145	33.1	0	3.8	1.2	38.2	1.28
R2417	Vehicle	M	4326998	63	2.63	121	101	10.8	63	0.039	2.42	5.3	0.83	181	2.6	308	1.92	26.0	144	37.0	0	4.0	1.1	36.0	1.42
R2418	Vehicle	M	4326999	80	2.62	195	100	9.9	65	0.045	2.45	6.9	0.52	233	1.9	185	3.05	30.7	146	34.3	0	4.2	1.1	34.8	1.12
Mean M				69.8	2.54	169	101	9.81	61.6	0.041	2.32	5.57	0.647	209	1.89	236	2.15	26.5	145	35.1	0.1	3.83	1.1	37.9	1.33
STDEV				21.83	0.072	91.4	1.1	0.863	1.78	0.0027	0.162	0.915	0.3391	63.0	0.373	57.0	0.419	1.61	1.1	1.33	0.32	0.177	0.06	1.78	0.099
**GROUP 2**																									
R2419	1	M	4324036	82	2.53	160	102	10.1	60	0.044	2.15	5.0	0.55	207	2.5	285	1.47	24.4	145	35.6	0	3.8	1.2	38.2	1.46
R2420	1	M	4324037	43	2.54	214	100	9.8	62	0.044	2.21	5.4	0.31	73	2.4	178	2.15	26.9	145	35.1	0	3.5	1.1	41.4	1.30
R2421	1	M	4324038	49	2.45	150	101	9.0	61	0.042	2.19	5.2	0.33	203	0.9	208	2.31	27.2	146	33.8	1	3.7	1.1	39.5	1.24
R2422	1	M	4324039	48	2.52	163	99	8.1	62	0.046	2.72	6.4	0.23	134	2.3	207	2.11	26.1	144	35.9	1	3.6	0.9	40.0	1.38
R2423	1	M	4324040	57	2.55	102	101	8.4	62	0.041	2.54	5.7	0.41	202	1.6	172	1.80	28.5	145	33.5	0	3.6	1.0	40.3	1.18
R2424	1	M	4326983	65	2.59	152	102	9.0	63	0.043	2.16	5.8	0.65	262	1.2	252	2.15	27.0	145	36.0	0	3.7	1.2	39.2	1.33
R2425	1	M	4326984	47	2.47	149	102	9.0	54	0.038	2.15	4.7	0.23	89	2.5	266	1.44	24.3	145	29.7	2	3.3	1.1	43.9	1.22
R2426	1	M	4326985	70	2.50	268	103	8.2	63	0.041	1.96	4.2	0.38	224	2.0	193	1.93	27.9	146	35.1	0	3.5	1.3	41.7	1.26
R2427	1	M	4326986	89	2.55	171	102	10.0	62	0.042	2.32	5.2	0.39	243	1.6	218	2.50	26.0	146	36.0	0	3.6	1.1	40.6	1.38
R2428	1	M	4326987	61	2.57	115	101	11.1	63	0.042	2.52	6.8	0.44	93	2.1	210	2.83	30.2	146	32.8	1	3.6	1.0	40.6	1.09
Mean M				61.1	2.53	164	101	9.27	61.2	0.042	2.29	5.44	0.392	173	1.91	219	2.07	26.9	145	34.4	0.5	3.59	1.1	40.5	1.28
STDEV				15.53	0.043	47.4	1.2	0.963	2.70	0.0022	0.232	0.772	0.1322	69.4	0.563	37.4	0.433	1.79	0.7	1.99	0.71	0.137	0.10	1.59	0.110
**GROUP 3**																									
R2429	20	M	4324041	86	2.53	214	102	10.6	61	0.044	2.51	5.4	0.25	205	2.4	228	1.47	26.7	144	34.3	0	4.3	1.0	33.5	1.28
R2430	20	M	4324042	49	2.37	127	103	9.80	59	0.041	2.15	4.9	0.24	156	2.6	231	2.29	24.1	146	34.9	0	3.5	1.1	41.7	1.45
R2431	20	M	4324043	80	2.54	190	101	10.7	63	0.039	2.23	5.7	0.49	233	1.6	294	1.80	26.7	144	36.3	0	3.7	1.1	38.9	1.36
R2432	20	M	4326993	56	2.48	140	102	9.0	58	0.038	2.21	4.7	0.29	190	2.0	262	1.90	23.3	145	34.7	1	3.7	1.1	39.2	1.49
R2433	20	M	4326994	43	2.61	107	100	11.9	63	0.037	2.21	4.5	0.75	80	2.4	226	1.70	26.4	145	36.6	1	3.4	1.2	42.6	1.39
R2434	20	M	4326988	103	2.53	124	101	10.2	59	0.041	2.16	4.7	0.69	325	1.9	191	1.84	22.9	146	36.1	0	3.6	1.2	40.6	1.58
R2435	20	M	4326989	53	2.48	119	103	10.3	59	0.039	1.99	5.0	0.96	87	2.5	254	1.73	24.5	146	34.5	0	3.3	1.2	44.2	1.41
R2436	20	M	4326990	59	2.52	138	102	10.4	60	0.039	2.23	4.5	0.41	141	2.3	181	2.01	24.7	146	35.3	1	3.8	1.1	38.4	1.43
R2437	20	M	4326991	65	2.63	122	104	9.8	63	0.036	2.22	5.9	0.94	95	2.5	208	2.47	27.4	145	35.6	1	3.9	1.2	37.2	1.30
R2438	20	M	4326992	53	2.59	121	102	8.7	62	0.039	2.05	4.5	0.36	122	2.5	269	2.52	28.7	145	33.3	1	3.4	1.3	42.6	1.16
Mean M				64.7	2.53	140	102	10.14	60.7	0.039	2.20	4.98	0.538	163	2.27	234	1.97	25.5	145	35.2	0.5	3.66	1.2	39.9	1.38
STDEV				19.01	0.075	34.4	1.2	0.902	1.95	0.0023	0.137	0.516	0.2772	76.8	0.327	35.7	0.348	1.91	0.8	1.02	0.53	0.295	0.07	3.16	0.117

ENTERED BY: EQ 14/2/12

CHECKED BY: [illegible] 14/2/12

C 37/45

ICPQN1035.B

Animals	Dose (mg/kg)	Units Sex	Submission #	ALT IU/L	Ca^{2+} mmol/L	CK IU/L	Cl^- mmol/L	Glu mmol/L	TP g/L	Cre mmol/L	Phos mmol/L	Urea mmol/L	Trig mmol/L	AST IU/L	Tbil umol/L	ALP IU/L	Chol mmol/L	Glob g/L	Na^+ mmol/L	ALB g/L	Gamma GT IU/L	K^+ mmol/L	Ca:P	Na:K	A:G
GROUP 4																									
R2439	Vehicle	M	4326973	105	2.69	138	100	9.9	62	0.044	2.43	5.2	1.26	335	2.7	142	2.87	30.7	142	31.3	1.0	3.9	1.1	36.4	1.02
R2440	Vehicle	M	4326974	93	2.57	200	100	8.4	60	0.047	2.43	5.0	0.52	270	2.2	123	2.78	29.1	144	30.9	0.0	3.8	1.1	37.9	1.06
R2441	Vehicle	M	4326975	38	2.55	128	102	9.6	60	0.050	2.22	4.9	0.61	74	2.5	123	1.69	28.5	145	31.5	1.0	4.0	1.1	36.3	1.11
R2442	Vehicle	M	4326976	114	2.55	140	103	8.3	61	0.046	2.31	5.8	0.29	316	2.1	178	1.90	30.0	144	31.0	1.0	4.4	1.1	32.7	1.03
R2443	Vehicle	M	4326977	62	2.64	175	102	9.3	60	0.043	2.32	5.4	1.26	128	3.4	125	2.47	28.4	145	31.6	1.0	4.3	1.1	33.7	1.11
Mean M				82	2.60	156	101	9.1	61	0.046	2.34	5.3	0.79	225	2.6	138	2.34	29.3	144	31.3	0.8	4.1	1.1	35.4	1.1
STDEV				31.7	0.062	30.3	1.3	0.72	0.9	0.0027	0.089	0.36	0.446	116.8	0.52	23.6	0.526	0.99	1.2	0.30	0.45	0.26	0.04	2.12	0.04
GROUP 5																									
R2444	20	M	4326978	62	2.59	102	101	8.7	61	0.044	2.30	5.9	0.38	249	2.7	173	2.30	29.4	143	31.6	1.0	4.0	1.1	35.8	1.07
R2445	20	M	4326979	51	2.63	181	100	9.1	62	0.042	2.26	4.6	0.66	193	2.4	152	2.21	31.1	144	30.9	1.0	3.7	1.2	38.9	0.99
R2446	20	M	4326980	40	2.61	97	102	9.4	61	0.044	2.13	5.1	0.48	110	2.3	129	2.85	31.5	144	29.5	1.0	3.7	1.2	38.9	0.94
R2447	20	M	4326981	94	2.57	109	102	9.2	65	0.047	2.07	5.0	0.56	402	2.5	111	2.25	32.7	144	32.3	0.0	4.0	1.2	36.0	0.99
R2448	20	M	4326982	63	2.56	97	105	9.7	63	0.044	1.99	5.9	0.37	228	0.7	155	2.14	30.2	144	32.8	1.0	3.6	1.3	40.0	1.09
Mean M				62	2.59	117	102	9.2	62	0.044	2.15	5.3	0.49	236	2.1	144	2.35	31.0	144	31.4	0.8	3.8	1.2	37.9	1.0
STDEV				20.2	0.029	36.0	1.9	0.37	1.7	0.0018	0.129	0.58	0.123	106.7	0.81	24.2	0.286	1.26	0.4	1.29	0.45	0.19	0.06	1.92	0.06

C 38/45

ENTERED BY: 8R 23/2/12

CHECKED BY: [illegible] 28/2/12

URINALYSIS

ICPQN1035.B

Animals	Dose (mg/kg)	Sex	Volume (mL)	Glucose	Bilirubin	Ketones	Specific Gravity	Blood	pH	Protein	Urobilinogen	Nitrite	Leucocytes
**Note:** Neg, Negative; Norm, Normal; -, No specimen collected from animal; nsq, Not sufficient quantity													
**GROUP 1**													
R2409	Vehicle	M	0	-	-	-	-	-	-	-	-	-	-
R2410	Vehicle	M	0.2	Neg	Neg	Small	1.030	Neg	7.0	2+	Norm	Neg	Neg
R2411	Vehicle	M	0.4	Neg	Neg	Small	1.025	Neg	6.0	2+	Norm	Neg	Neg
R2412	Vehicle	M	1	Neg	Neg	Trace	1.020	Neg	6.0	Trace	Norm	Neg	Neg
R2413	Vehicle	M	0.2	Neg	Neg	Neg	1.020	Neg	6.5	1+	Norm	Neg	Neg
R2414	Vehicle	M	0.2	Neg	Neg	Neg	1.030	Neg	7.5	2+	Norm	Neg	Neg
R2415	Vehicle	M	0	-	-	-	-	-	-	-	-	-	-
R2416	Vehicle	M	0.4	Neg	Neg	Trace	1.005	Neg	6.5	1+	Norm	Neg	Neg
R2417	Vehicle	M	0.15	Neg	Neg	Trace	1.010	Neg	7.0	2+	Norm	Neg	Neg
R2418	Vehicle	M	0	-	-	-	-	-	-	-	-	-	-
Mean M							1.020		6.6				
STDEV							0.0096		0.56				
**GROUP 2**													
R2419	1	M	0.3	Neg	Neg	Neg	1.005	Neg	8.0	2+	Norm	Neg	Neg
R2420	1	M	0.2	Neg	Neg	Neg	1.005	Neg	7.0	1+	Norm	Neg	Neg
R2421	1	M	0.2	Neg	Neg	Trace	1.010	Neg	7.0	1+	Norm	Neg	Neg
R2422	1	M	0.5	Neg	Neg	Trace	1.015	Neg	7.0	1+	Norm	Neg	Neg
R2423	1	M	0.4	Neg	Neg	Trace	1.020	Neg	6.5	1+	Norm	Neg	Neg
R2424	1	M	0.15	Neg	Neg	Small	1.020	Neg	7.0	2+	Norm	Neg	Neg
R2425	1	M	1.2	Neg	Neg	Trace	1.005	Neg	7.5	Trace	Norm	Neg	Neg
R2426	1	M	0	-	-	-	-	-	-	-	-	-	-
R2427	1	M	0	-	-	-	-	-	-	-	-	-	-
R2428	1	M	0.45	Neg	Neg	Trace	1.015	Neg	7.0	Trace	Norm	Neg	Neg
Mean M							1.012		7.1				
STDEV							0.0065		0.44				
**GROUP 3**													
R2429	20	M	0	-	-	-	-	-	-	-	-	-	-
R2430	20	M	0.2	Neg	Neg	Small	1.020	Neg	7.0	1+	Norm	Neg	Neg
R2431	20	M	0.2	Neg	Neg	Trace	1.015	Neg	7.0	1+	Norm	Neg	Neg
R2432	20	M	1	Neg	Neg	Small	1.005	Neg	7.5	1+	Norm	Neg	Neg
R2433	20	M	0.5	Neg	Neg	Small	1.010	Neg	7.5	1+	Norm	Neg	Neg
R2434	20	M	0.2	Neg	Neg	Trace	1.010	Neg	7.0	1+	Norm	Neg	Neg
R2435	20	M	0.8	Neg	Neg	Neg	1.005	Neg	7.5	2+	Norm	Neg	Neg
R2436	20	M	0.3	Neg	Neg	Trace	1.005	Neg	7.0	1+	Norm	Neg	Neg
R2437	20	M	1	Neg	Neg	Trace	1.005	Neg	7.0	1+	Norm	Neg	Neg
R2438	20	M	0.45	Neg	Neg	Small	1.005	Neg	7.5	2+	Norm	Neg	Neg
Mean M							1.009		7.2				
STDEV							0.0055		0.26				

ENTERED BY ER 23/2/12

CHECKED BY: [illegible] 23/2/12

C 39/45

URINALYSIS

ICPQN1035.B

Animals	Dose (mg/kg)	Sex	Volume (mL)	Glucose	Bilirubin	Ketones	Specific Gravity	Blood	pH	Protein	Urobilinogen	Nitrite	Leucocytes
**GROUP 4**													
R2439	Vehicle	M	0.4	neg	neg	trace	1.020	small	6.5	2+	norm	neg	neg
R2440	Vehicle	M	0.25	neg	neg	neg	1.005	small	7.5	2+	norm	neg	neg
R2441	Vehicle	M	0.1	neg	neg	neg	1.010	small	7.5	1+	norm	neg	neg
R2442	Vehicle	M	0.45	neg	neg	neg	1.015	small	7.0	2+	norm	neg	neg
R2443	Vehicle	M	1	neg	neg	small	1.020	neg	7.0	2+	norm	neg	neg
Mean M							1.014		7.1				
STDEV							0.0065		0.42				
**GROUP 5**													
R2444	20	M	0.2	neg	neg	neg	1.030	neg	6.5	2+	norm	neg	neg
R2445	20	M	0.2	neg	neg	trace	1.005	small	7.5	1+	norm	neg	neg
R2446	20	M	0.9	neg	neg	neg	1.005	neg	7.5	neg	norm	neg	neg
R2447	20	M	0.05	neg	neg	trace	1.020	neg	7.5	neg	norm	neg	neg
R2448	20	M	0.5	neg	neg	trace	1.010	trace	7.0	1+	norm	neg	neg
Mean M							1.014		7.2				
STDEV							0.0108		0.45				

Note:
Neg, Negative
Norm, Normal
-, No specimen collected from animal
nsq, Not sufficient quantity

ENTERED BY ER 23/2/12

CHECKED BY: [illegible] 23/2/12

C 40/45

ORGAN WEIGHT/ ORGAN WEIGHT AS % BODY WEIGHT
MAIN GROUPS

ICPQN1035.B

LAB-027/f2

Animals	Dose (mg/kg)	Sex	Body weight Day 29	Liver	% Liver	Kidneys	%Kidneys	Adrenals	%Adrenals	Testes	%Testes	Spleen	%Spleen	Brain	%Brain	Heart	%Heart
**GROUP 1**																	
R2409	Vehicle	M	404	15.08	3.733	3.2093	0.7944	0.04549	0.01126	3.1854	0.7885	0.6328	0.1566	2.0534	0.5083	1.2948	0.3205
R2410	Vehicle	M	405	12.75	3.148	3.2524	0.8031	0.04573	0.01129	3.2378	0.7995	0.8602	0.2124	2.0360	0.5027	1.2014	0.2966
R2411	Vehicle	M	421	13.73	3.261	3.3692	0.8003	0.05112	0.01214	3.3741	0.8014	0.6806	0.1617	2.0610	0.4895	1.2226	0.2904
R2412	Vehicle	M	374	11.00	2.941	2.6982	0.7214	0.04409	0.01179	2.8343	0.7578	0.7478	0.1999	1.9430	0.5195	1.2070	0.3227
R2413	Vehicle	M	376	11.97	3.184	2.9125	0.7746	0.05694	0.01514	3.0543	0.8123	0.9363	0.2490	2.0449	0.5439	1.2670	0.3370
R2414	Vehicle	M	413	15.43	3.736	2.7477	0.6653	0.04803	0.01163	3.2183	0.7792	0.7560	0.1831	2.0095	0.4866	1.3101	0.3172
R2415	Vehicle	M	424	15.62	3.684	3.0494	0.7192	0.04284	0.01010	2.8834	0.6800	0.6837	0.1613	1.9963	0.4708	1.4633	0.3451
R2416	Vehicle	M	421	16.72	3.971	3.3240	0.7895	0.03944	0.00937	3.0116	0.7153	0.9252	0.2198	1.9553	0.4644	1.4309	0.3399
R2417	Vehicle	M	399	12.29	3.080	3.1878	0.7989	0.05654	0.01417	3.2690	0.8193	0.6907	0.1731	2.0219	0.5067	1.2462	0.3123
R2418	Vehicle	M	372	13.47	3.621	2.7688	0.7443	0.03911	0.01051	3.3007	0.8873	0.6285	0.1690	2.0091	0.5401	1.1381	0.3059
Mean M			401	13.8	3.44	3.052	0.76	0.0469	0.012	3.137	0.78	0.754	0.19	2.013	0.50	1.278	0.32
STDEV			20.3	1.85	0.351	0.2528	0.047	0.00630	0.0018	0.1820	0.057	0.1148	0.031	0.0396	0.027	0.1020	0.018
**GROUP 2**																	
R2419	1	M	356	10.16	2.854	2.5664	0.7209	0.05123	0.01439	3.2492	0.9127	0.7342	0.2062	1.9379	0.5444	1.2113	0.3403
R2420	1	M	379	11.70	3.087	2.5898	0.6833	0.03784	0.00998	2.8877	0.7619	0.8426	0.2223	2.0066	0.5294	1.0291	0.2715
R2421	1	M	411	13.81	3.360	3.3367	0.8118	0.04546	0.01106	3.2971	0.8022	0.7989	0.1944	2.0387	0.4960	1.2575	0.3060
R2422	1	M	419	13.94	3.327	2.6459	0.6315	0.05772	0.01378	2.6910	0.6422	0.7883	0.1881	2.0847	0.4975	1.4220	0.3394
R2423	1	M	386	12.10	3.135	2.6835	0.6952	0.04616	0.01196	2.9930	0.7754	0.7008	0.1816	1.9565	0.5069	1.0904	0.2825
R2424	1	M	435	18.22	4.189	3.6842	0.8469	0.03643	0.00837	3.4692	0.7975	0.7943	0.1826	2.1059	0.4841	1.4357	0.3300
R2425	1	M	405	13.33	3.291	3.1660	0.7817	0.05278	0.01303	3.3823	0.8351	0.7878	0.1945	2.0797	0.5135	1.1857	0.2928
R2426	1	M	368	10.71	2.910	2.7289	0.7415	0.03255	0.00885	2.6275	0.7140	0.8474	0.2303	2.1369	0.5807	1.2102	0.3289
R2427	1	M	362	11.74	3.243	2.7258	0.7530	0.04551	0.01257	3.0165	0.8333	0.8019	0.2215	2.0792	0.5744	1.0784	0.2979
R2428	1	M	424	16.34	3.854	3.4188	0.8063	0.04829	0.01139	3.2336	0.7626	0.9343	0.2204	2.0329	0.4795	1.1879	0.2802
Mean M			395	13.2	3.32	2.955	0.75	0.0454	0.012	3.085	0.78	0.8031	0.20	2.046	0.52	1.211	0.31
STDEV			28.0	2.52	0.411	0.4075	0.066	0.00783	0.0020	0.2880	0.073	0.0637	0.018	0.0642	0.036	0.1348	0.026
t-test					0.525		0.596		0.813		0.990		0.187		0.235		0.254

C 41/45

ENTERED BY: ER 10/2/12

CHECKED BY: [illegible] 10/2/12

ORGAN WEIGHT/ ORGAN WEIGHT AS % BODY WEIGHT
MAIN GROUPS

ICPQN1035.B

LAB-027/f2

Animals	Dose (mg/kg)	Sex	Body weight Day 29	Liver	% Liver	Kidneys	%Kidneys	Adrenals	%Adrenals	Testes	%Testes	Spleen	%Spleen	Brain	%Brain	Heart	%Heart
**GROUP 3**																	
R2429	20	M	336	11.23	3.342	2.6984	0.8031	0.06018	0.01791	3.2264	0.9602	0.7005	0.2085	2.0290	0.6039	1.2573	0.3742
R2430	20	M	386	12.50	3.238	3.0175	0.7817	0.04963	0.01286	2.5076	0.6496	0.8286	0.2147	2.0478	0.5305	1.2394	0.3211
R2431	20	M	376	12.74	3.388	3.0951	0.8232	0.03974	0.01057	3.2773	0.8716	0.7290	0.1939	1.9710	0.5242	1.2244	0.3256
R2432	20	M	368	11.41	3.101	2.9686	0.8067	0.04027	0.01094	3.3258	0.9038	0.7868	0.2138	2.0538	0.5581	1.3657	0.3711
R2433	20	M	410	14.60	3.561	3.2612	0.7954	0.03262	0.00796	3.0203	0.7367	1.0341	0.2522	2.0028	0.4885	1.4402	0.3513
R2434	20	M	414	14.05	3.394	2.8779	0.6951	0.04738	0.01144	3.1427	0.7591	0.6973	0.1684	2.0260	0.4894	1.4426	0.3485
R2435	20	M	410	14.22	3.468	3.0046	0.7328	0.05872	0.01432	3.1328	0.7641	0.6666	0.1626	1.9659	0.4795	1.2224	0.2981
R2436	20	M	389	13.75	3.535	2.8626	0.7359	0.04084	0.01050	2.9094	0.7479	0.6249	0.1606	1.9786	0.5086	1.2339	0.3172
R2437	20	M	452	16.50	3.650	3.3656	0.7446	0.05060	0.01119	3.2856	0.7269	0.9615	0.2127	2.1072	0.4662	1.4953	0.3308
R2438	20	M	409	15.31	3.743	3.0308	0.7410	0.05325	0.01302	3.0977	0.7574	0.6639	0.1623	2.0839	0.5095	1.2991	0.3176
Mean M			395	13.6	3.44	3.018	0.77	0.0473	0.012	3.093	0.79	0.769	0.19	2.027	0.52	1.322	0.34
STDEV			31.5	1.68	0.192	0.1928	0.042	0.00889	0.0027	0.2424	0.094	0.1354	0.031	0.0479	0.041	0.105	0.025
t-test					0.962		0.809		0.749		0.918		0.410		0.428		0.104

C 42/45

ENTERED BY: [initials] 10/2/12

CHECKED BY: [initials] 10/2/12

ORGAN WEIGHT/ ORGAN WEIGHT AS % BODY WEIGHT
MAIN GROUPS

ICPQN1035.B

LAB-027/f2

Animals	Dose (mg/kg)	Sex	Thymus	%Thymus	Epididymis	%Epididymis	Prostate + seminal vesicles	%Prostate + seminal vesicles
**GROUP 1**								
R2409	Vehicle	M	0.4039	0.1000	1.3598	0.3366	2.7850	0.6894
R2410	Vehicle	M	0.6863	0.1695	1.2081	0.2983	2.2850	0.5642
R2411	Vehicle	M	0.4214	0.1001	1.2867	0.3056	2.5960	0.6166
R2412	Vehicle	M	0.4638	0.1240	1.1141	0.2979	2.4160	0.6460
R2413	Vehicle	M	0.4144	0.1102	1.0060	0.2676	2.5130	0.6684
R2414	Vehicle	M	0.5010	0.1213	0.9904	0.2398	2.4563	0.5947
R2415	Vehicle	M	0.5184	0.1223	1.1474	0.2706	2.4384	0.5751
R2416	Vehicle	M	0.5316	0.1263	1.2902	0.3065	2.4645	0.5854
R2417	Vehicle	M	0.5447	0.1365	1.1232	0.2815	2.9353	0.7357
R2418	Vehicle	M	0.3889	0.1045	1.1634	0.3127	2.6867	0.7222
Mean M			0.487	0.12	1.169	0.29	2.558	0.64
STDEV			0.0900	0.021	0.1204	0.027	0.1949	0.062
**GROUP 2**								
R2419	1	M	0.5725	0.1608	1.1294	0.3172	2.0784	0.5838
R2420	1	M	0.4994	0.1318	1.0006	0.2640	2.7431	0.7238
R2421	1	M	0.4409	0.1073	1.1047	0.2688	2.7269	0.6635
R2422	1	M	0.4694	0.1120	1.2225	0.2918	2.2701	0.5418
R2423	1	M	0.4339	0.1124	1.1302	0.2928	2.6746	0.6929
R2424	1	M	0.4099	0.0942	1.2162	0.2796	2.7014	0.6210
R2425	1	M	0.6764	0.1670	1.1994	0.2961	2.4950	0.6160
R2426	1	M	0.3874	0.1053	1.0405	0.2827	2.1937	0.5961
R2427	1	M	0.3577	0.0988	1.0694	0.2954	2.3665	0.6537
R2428	1	M	0.3710	0.0875	1.2780	0.3014	2.2940	0.5410
Mean M			0.462	0.12	1.139	0.29	2.454	0.62
STDEV			0.0991	0.027	0.0888	0.016	0.2462	0.061
t-test				0.733		0.790		0.558

C 43/45

ENTERED BY: BR 10/2/12

CHECKED BY: [illegible] 10/2/12

ORGAN WEIGHT/ ORGAN WEIGHT AS % BODY WEIGHT
MAIN GROUPS

ICPQN1035.B

LAB-027/f2

Animals	Dose (mg/kg)	Sex	Thymus	%Thymus	Epididymis	%Epididymis	Prostate + seminal vesicles	%Prostate + seminal vesicles
**GROUP 3**								
R2429	20	M	0.3844	0.1144	1.2355	0.3677	2.7552	0.8200
R2430	20	M	0.3566	0.0924	1.0587	0.2743	2.8293	0.7330
R2431	20	M	0.5432	0.1445	1.0816	0.2877	2.6423	0.7027
R2432	20	M	0.3484	0.0947	1.1868	0.3225	2.3112	0.6280
R2433	20	M	0.5032	0.1227	1.0361	0.2527	2.6419	0.6444
R2434	20	M	0.4605	0.1112	1.0904	0.2634	2.6337	0.6362
R2435	20	M	0.4160	0.1015	1.0600	0.2585	2.7036	0.6594
R2436	20	M	0.3890	0.1000	0.9954	0.2559	2.2033	0.5664
R2437	20	M	0.4711	0.1042	1.2398	0.2743	2.9544	0.6536
R2438	20	M	0.4964	0.1214	1.1383	0.2783	2.8542	0.6978
Mean M			0.437	0.11	1.112	0.28	2.653	0.67
STDEV			0.0673	0.016	0.0844	0.036	0.2345	0.069
t-test				0.210		0.574		0.257

C 44/45

ENTERED BY: ß2 10/2/12

CHECKED BY: d 10/2/12

ORGAN WEIGHT/ ORGAN WEIGHT AS % BODY WEIGHT
RECOVERY GROUPS

ICPQN1035.B

LAB-027/f2

Animals	Dose (mg/kg)	Sex	Body weight Day 43	Liver	% Liver	Kidneys	%Kidneys	Adrenals	%Adrenals	Testes	%Testes	Spleen	%Spleen	Brain	%Brain	Heart	%Heart	Thymus	%Thymus	Epididymis	%Epididymis	Prostate + seminal vesicles	%Prostate + seminal vesicles
**GROUP 4**																							
R2439	Vehicle	M	526	19.91	3.785	3.4340	0.6529	0.04897	0.00931	3.1220	0.5935	0.7490	0.1424	2.1029	0.3998	1.5627	0.2971	0.4888	0.0929	1.3174	0.2505	3.0963	0.5887
R2440	Vehicle	M	439	13.33	3.036	2.8734	0.6545	0.05105	0.01163	2.8410	0.6472	0.7648	0.1742	1.9788	0.4508	1.2577	0.2865	0.5046	0.1149	1.2824	0.2921	2.9941	0.6820
R2441	Vehicle	M	457	13.16	2.880	3.1164	0.6819	0.04226	0.00925	3.2544	0.7121	0.8355	0.1828	2.0281	0.4438	1.3443	0.2942	0.4179	0.0914	1.2400	0.2713	2.8860	0.6315
R2442	Vehicle	M	399	11.73	2.940	2.5767	0.6458	0.04309	0.01080	3.0529	0.7651	0.7678	0.1924	1.9739	0.4947	1.2516	0.3137	0.3406	0.0854	1.3186	0.3305	3.2187	0.8067
R2443	Vehicle	M	520	19.46	3.742	3.1183	0.5997	0.05287	0.01017	3.4751	0.6683	0.7504	0.1443	2.1292	0.4095	1.6002	0.3077	0.4585	0.0882	1.4257	0.2742	2.9591	0.5691
Mean M			468	15.5	3.28	3.02	0.647	0.0476	0.0102	3.15	0.677	0.774	0.167	2.04	0.440	1.40	0.300	0.442	0.095	1.317	0.284	3.031	0.656
STDEV			54.3	3.86	0.448	0.319	0.0298	0.00475	0.00101	0.236	0.0651	0.0357	0.0227	0.071	0.0377	0.167	0.0109	0.0657	0.0118	0.0688	0.0300	0.1295	0.0950
**GROUP 5**																							
R2444	20	M	448	12.50	2.790	3.0620	0.6835	0.04930	0.01100	3.5136	0.7843	0.8235	0.1838	2.0398	0.4553	1.3778	0.3075	0.5674	0.1267	1.3814	0.3083	3.1592	0.7052
R2445	20	M	461	13.61	2.952	3.0005	0.6509	0.04265	0.00925	3.0996	0.6724	0.8371	0.1816	2.0313	0.4406	1.3614	0.2953	0.3405	0.0739	1.2989	0.2818	2.9248	0.6344
R2446	20	M	468	14.82	3.167	3.0730	0.6566	0.04147	0.00886	2.6660	0.5697	0.9494	0.2029	2.1013	0.4490	1.3702	0.2928	0.4316	0.0922	1.1290	0.2412	3.1102	0.6646
R2447	20	M	476	17.04	3.580	2.9564	0.6211	0.03654	0.00768	2.7360	0.5748	0.7139	0.1500	1.9953	0.4192	1.3400	0.2815	0.4486	0.0942	1.2019	0.2525	3.3283	0.6992
R2448	20	M	448	14.72	3.286	3.0508	0.6810	0.05158	0.01151	3.0392	0.6784	0.8036	0.1794	2.1496	0.4798	1.3741	0.3067	0.4472	0.0998	1.2444	0.2778	2.8962	0.6465
Mean M			460	14.5	3.15	3.03	0.659	0.0443	0.0097	3.01	0.656	0.826	0.180	2.06	0.449	1.36	0.297	0.447	0.097	1.251	0.272	3.084	0.670
STDEV			12.3	1.69	0.305	0.049	0.0254	0.00610	0.00158	0.338	0.0884	0.0843	0.0190	0.061	0.0221	0.015	0.0108	0.0807	0.0191	0.0957	0.0263	0.1779	0.0314
t-test					0.6309		0.5252		0.5199		0.6762		0.3815		0.6568		0.6671		0.7890		0.5419		0.7610

C 45/45

ENTERED BY: [illegible] 23/2/12

CHECKED BY: [illegible] 23/2/12

I C P Firefly Pty Ltd

ACN 071 626 358

PO Box 6198, Alexandria NSW 2015 Australia
TEL: 61 2 9310 3899 FAX: 61 2 9310 4889 EMAIL: info@icpfirefly.com.au WEBSITE: www.icpfirefly.com.au

Appendix D: Haematology & Biochemistry 1-2

IDEXX LABORATORIES
IDEXX LABORATORIES Pty. Ltd.
ACN: 063 154 352 ABN: 31 063 154 352

ICP Firefly- Study Number QN 1035.B – Sprague Dawley rats

TESTS:
Haematology and Biochemistry results from 3 groups of 10-12 week old male rats. Group 1 consisted of 10 animals (R2409-R2418), Group 2 consisted of 10 animals (R2419-R2428) and Group 3 consisted of 10 animals (R2429-R2438). Group 2 received the test item at 1 mg/kg and Group 3 received the test item at 20 mg/kg. Group 1 (control group) received vehicle.

The results of Group 2 and Group 3 were compared with the results of Group 1 for general significant clinical differences.

RESULTS:
Haematology
One animal in the control group has significantly lower WBC (and hence absolute numbers of various leukocytes from the differential) than its group mates and is considered to be an outlier. There is no obvious test item related effect.

Any differences are small and interpreted to be not clinically relevant.

Biochemistry
There is no obvious test item related effect.

Any differences are small and interpreted to be not clinically relevant.

CONCLUSIONS:
There are no treatment related changes in any of the treatment groups.

Any other differences are small and interpreted to be not clinically relevant.

[signature]

RICHARD MILLER BVSc MSc PhD MACVSc Dip ACVP
Specialist Veterinary Pathologist
16 February 2012

LABORATORIES
IDEXX LABORATORIES Pty. Ltd.
ACN: 063 154 352 ABN: 31 063 154 352

ICP Firefly- Study Number QN 1035.B Recovery – Sprague Dawley rats

TESTS:
Haematology and Biochemistry results from 2 groups of 10-12 week old male rats. Group 4 consisted of 5 animals (R2439-R2443) and Group 5 consisted of 5 animals (R2444-R2448). Group 5 received the test item at 20 mg/kg. Group 4 (control group) received vehicle. These animals are cohorts of a previous study report (QN 1035.B) but were allowed to recover from testing for 2 weeks with no additional treatment.

The results of Group 5 were compared with the results of Group 4 for general significant clinical differences.

RESULTS:
Haematology
There is no obvious test item related effect.

Any differences are small and interpreted to be not clinically relevant.

Biochemistry
There is no obvious test item related effect.

Any differences are small and interpreted to be not clinically relevant.

CONCLUSIONS:
There are no treatment related changes.

Any other differences are small and interpreted to be not clinically relevant.

Richard Miller

RICHARD MILLER BVSc MSc PhD MACVSc Dip ACVP
Specialist Veterinary Pathologist
27 February 2012

The bright light of
certainty

I C P Firefly Pty Ltd

ACN 071 626 358

PO Box 6198, Alexandria NSW 2015 Australia
TEL: 61 2 9310 3899 FAX: 61 2 9310 4889 EMAIL: info@icpfirefly.com.au WEBSITE: www.icpfirefly.com.au

Appendix E: Histopathology 1-25

Repeated Dose 28-Day Oral Toxicity Main Study (OECD 407) of c2 in Sprague Dawley Rats with a Recovery Period

Project No: K12/002
ICP Firefly Study No: ICPQN1035.B

7th March 2012

Final Histopathology Phase Report

E 1/25

COPY AUTHENTICATION

Project No: K12/002

Study No: ICPQN1035.B

Study Title: Repeated Dose 28-Day Oral Toxicity Main Study (OECD 407) of c2 in Sprague Dawley Rats with a Recovery Period

This is a certified copy of the Final Histopathology Phase Report from Project No: K12/002. The original final histopathology phase report and supporting documentation and records will be returned to the Test Facility at ICP Firefly Pty Ltd, PO Box 6198, Alexandria, NSW 2015, Australia , within four weeks of receiving confirmation that the Final Histopathology Phase Report has been received.

Signed: KRIsaacs

K R Isaacs MA, VetMB, MRCVS, FRCPath

Date: 7th March 2012

E 2/25

HISTOPATHOLOGY PHASE REPORT

Text and Tables I – II

AUTHENTICATION 3

QUALITY ASSURANCE INSPECTION 4

SUMMARY 5

INTRODUCTION 5

MATERIALS 5

TISSUES EXAMINED 6

METHODS 6

RESULTS 7

TABLES

Table I	Histopathology Incidence Table – Terminal Kill
Table II	Individual Animal Reports

Authentication

I, the undersigned, as assigned Principal Investigator (Histopathology Phase), hereby declare that the following histopathology phase report constitutes a true and faithful account of the procedures adopted and the results obtained in the performance of the histopathology phase of this study.

This establishment is a member of the United Kingdom Department of Health Good Laboratory Practice Compliance Programme and this study was conducted according to the principles of GLP.

The procedures adopted in the conduct of the histopathology phase of this study are intended to conform to the guidelines of the OECD Principles of Good Laboratory Practice, revised 1997, issued Jan 1998, ENV/MC/CHEM(98)17 and the United Kingdom Good Laboratory Practice Regulations Statutory Instrument 1999 No. 3106 as amended by the Good Laboratory Practice (Codification Amendment Etc) Regulations 2004 Statutory Instrument No. 994.

Signed KRIsaacs

K R Isaacs M.A., Vet.M.B., M.R.C.V.S., F.R.C.Path

Date 7th March 2012

Prepared by
K R Isaacs M.A., Vet.M.B., M.R.C.V.S., F.R.C.Path
14 Rossett Park Road
Harrogate
North Yorkshire HG2 9NP
England

E 4/25

Quality Assurance Inspection

As far as can reasonably be established, I confirm that the methods, procedures and observations are accurately and completely described, and the reported results in this histopathology phase report accurately and completely reflect the raw data.

The dates on which the study was inspected and the findings reported to Management were as follows:

Study Based Inspections	Date Inspected	Date Reported to Principal Investigator/ Management	Date Reported to Study Director, Test Facility Management and Lead QA
Project Initiation	28th February 2012	28th February 2012	6th March 2012
Examination of slides & data recording	29th February 2012	29th February 2012	6th March 2012
1st Draft Report & Tables	6th March 2012	6th March 2012	6th March 2012
Inventory out of slides	7th March 2012	7th March 2012	7th March 2012
Final Report & Tables	7th March 2012	7th March 2012	7th March 2012

The following quarterly process-based inspections were conducted during the quarter that the histopathology phase of the study was conducted.

Process Based Inspections	Date Inspected	Date Reported to Principal Investigator/ Management	Date Reported to Study Director, Test Facility Management and Lead QA
Inventory in of slides	20th February 2012	20th February 2012	6th March 2012
Examination of slides & data recording	29th February 2012	29th February 2012	6th March 2012
Inventory out of slides	7th March 2012	7th March 2012	7th March 2012

An independent facility-based inspection is conducted annually at this test site. The last inspection was on 29th June 2011.

Signature: H M Isaacs
Hazel M Isaacs, Quality Assurance Inspector

Date: 7th March 2012

E 5/25

SUMMARY

Sections from a 28-day repeated dose oral toxicity study of c2 at doses of 0 (Vehicle), 1 or 20 mg/kg, once every 3 days, were submitted for histopathological evaluation. Further groups of 5 animals were dosed at doses of 0 or 20 mg/kg, once every 3 days, for 28 days and allowed to recover following withdrawal of treatment for 14 days.

TERMINAL KILL

There was no evidence of an effect of treatment in the sections submitted.

INTRODUCTION

Sections from a 28-day repeated dose oral toxicity study of c2 at doses of 0 (Vehicle), 1 or 20 mg/kg, once every 3 days, were submitted for histopathological evaluation. Further groups of 5 animals were dosed at doses of 0 or 20 mg/kg, once every 3 days, for 28 days and allowed to recover following withdrawal of treatment for 14 days.

The study details were as follows:

Group	Dosage level: c2 (mg/kg)	Animal Numbers
1	0 (Vehicle) – Main test	R2409 – R2418
2	1 – Main test	R2419 – R2428
3	20 – Main test	R2429 – R2438
4	0 (Vehicle) - Recovery	R2439 – R2443
5	20 - Recovery	R2444 – R2448

MATERIALS

The following documentation was provided by ICP Firefly Pty Ltd, PO Box 6198, Alexandria, NSW 2015, Australia:

- ***Experimental Protocol & Study Plan***
- ***Summarised results***

Histological sections were delivered to the premises of K R Isaacs on 28th February 2012 where the slide numbers were checked against the ICP Firefly histopathology slide summary forms.

E 6/25

TISSUES EXAMINED

The following tissues were examined from all animals in groups 1 and 3:

Tissue	No. of H&E Sections	Tissue	No. of H&E Sections
Adrenal gland	1	Seminal vesicles & coagulating gland	1
Brain	3	Small intestine: ileum (including Peyer's patches)	2
Bone marrow section	1	Spinal cord	4
Epididymis	1	Spleen	1
Heart	3	Stomach	2
Kidney	1	Testis	1
Large intestine: colon	2	Thymus	1
Liver	1	Thyroid gland	1
Lymph node: mesenteric	1	Trachea	1
Lung	1	Urinary bladder	2
Prostate gland	1	Gross lesions	1 or more

METHODS

Data

From the information supplied by the sponsor a file, exclusive to Project No: K12/002 was created on *Roelee 3.1 (Build 38),* a validated computer program.

All tissue sections were examined by light microscopy and the findings recorded directly into the computer system.

Tables were generated from the data in Project file K12/002 and used by the pathologist, in the assessment of the histopathological findings associated with the administration of the test compound.

Archive

The original final histopathology phase report and supporting documentation and records will be returned to the premises of ICP Firefly Pty Ltd, PO Box 6198, Alexandria, NSW 2015, Australia for archiving within four weeks of the issue of the Final Report.

RESULTS

The individual animal findings are presented in Table II and summarised in Table I (Histopathology Incidence).

TERMINAL KILL

There was no evidence of an effect of treatment in the sections submitted.

Incidental findings

A variety of spontaneous changes was noted in control and treated animals with no indication of an effect of treatment and the spectrum of these findings is consistent with changes encountered in rats of this age kept under laboratory conditions.

TABLE I
HISTOPATHOLOGY INCIDENCE TABLE

E 9/25

Table I
Histopathology Incidence Table

Dosage level c2 (mg/kg)	Main 0	Main 1	Main 20	Rec 0	Rec 20
number of animals	10	10	10	5	5
**ADRENAL GLANDS**					
number examined	10	-	10	-	-
**Vacuolation, zona fasciculata, diffuse**					
- grade 1 of 5 (minimal)	4	-	6	-	-
**BONE MARROW SECTION**					
number examined	10	-	10	-	-
**BRAIN**					
number examined	10	-	10	-	-
**EPIDIDYMIDES**					
number examined	10	-	10	-	-
**HEART**					
number examined	10	-	10	-	-
**Inflammation, mononuclear cell, myocardial, focal**					
- grade 1 of 5 (minimal)	2	-	1	-	-

Table I
Histopathology Incidence Table

Dosage level c2 (mg/kg)	Main 0	Main 1	Main 20	Rec 0	Rec 20
number of animals	10	10	10	5	5
**KIDNEYS**					
number examined	10	-	10	-	-
**Basophilic (regenerative) tubules, focal**					
- grade 1 of 5 (minimal)	5	-	3	-	-
**Hyaline droplets, proximal tubules**					
- grade 1 of 5 (minimal)	0	-	1	-	-
**Scar, cortex/medulla, focal**					
- grade 1 of 5 (minimal)	0	-	1	-	-
**LARGE INTESTINE: COLON**					
number examined	10	-	10	-	-
**LIVER**					
number examined	10	-	10	-	-
**Inflammation, mononuclear cell, focal**					
- grade 1 of 5 (minimal)	1	-	1	-	-
**Lipidosis, tension**					
- grade 1 of 5 (minimal)	0	-	1	-	-

Table I
Histopathology Incidence Table

Dosage level c2 (mg/kg)	Main 0	Main 1	Main 20	Rec 0	Rec 20
number of animals	10	10	10	5	5
**LUNG**					
number examined	10	-	10	-	-
**Alveolar macrophages, foamy, focal**					
- grade 1 of 5 (minimal)	1	-	0	-	-
**Inflammation, mononuclear cell, perivascular**					
- grade 1 of 5 (minimal)	2	-	0	-	-
- grade 2 of 5 (slight)	1	-	0	-	-
Total	3	-	0	-	-
**Inflammation, mononuclear cell, interstitial,**					
- grade 1 of 5 (minimal)	1	-	0	-	-
- grade 2 of 5 (slight)	1	-	0	-	-
Total	2	-	0	-	-
**Haemorrhage, intra-alveolar (agonal)**					
- grade 1 of 5 (minimal)	2	-	0	-	-
**LYMPH NODE: MESENTERIC**					
number examined	10	-	10	-	-
**Increased size/lymphocyte density, paracortex**					
- grade 1 of 5 (minimal)	0	-	1	-	-
**PEYER'S PATCHES (MALT)**					
number examined	10	-	10	-	-
**Mineralisation, focal**					
- grade 1 of 5 (minimal)	1	-	1	-	-

Table I
Histopathology Incidence Table

	Main	Main	Main	Rec	Rec
**Dosage level c2 (mg/kg)**	**0**	**1**	**20**	**0**	**20**
number of animals	10	10	10	5	5
**PROSTATE GLAND**					
number examined	10	-	10	-	-
**Inflammation, mononuclear cell, focal**					
- grade 1 of 5 (minimal)	0	-	5	-	-
- grade 2 of 5 (slight)	2	-	0	-	-
Total	2	-	5	-	-
**Atrophy, focal**					
- grade 2 of 5 (slight)	1	-	0	-	-
**SEMINAL VESICLE/COAGULATING G.**					
number examined	10	-	10	-	-
**Artefactual (barbiturate lysis) lesion**					
present	0	-	1	-	-
**SMALL INTESTINE: ILEUM**					
number examined	10	-	10	-	-
**SPINAL CORD**					
number examined	10	-	10	-	-
**SPLEEN**					
number examined	10	-	10	-	-
**STOMACH: GLANDULAR**					
number examined	10	-	10	-	-

Table I
Histopathology Incidence Table

	Main	Main	Main	Rec	Rec
**Dosage level c2 (mg/kg)**	**0**	**1**	**20**	**0**	**20**
number of animals	10	10	10	5	5
**STOMACH: NON-GLANDULAR**					
number examined	10	-	10	-	-
**TESTES**					
number examined	10	-	10	-	-
**THYMUS**					
number examined	10	-	10	-	-
**Congestion/haemorrhage (agonal)**					
- grade 1 of 5 (minimal)	4	-	1	-	-
**THYROID GLAND**					
number examined	10	-	10	-	-
**Heterotopic thymic tissue**					
present - unilateral	2	-	1	-	-
**TRACHEA**					
number examined	10	-	10	-	-
**URINARY BLADDER**					
number examined	10	-	10	-	-
**Mineralisation, adventitia, focal**					
- grade 1 of 5 (minimal)	1	-	0	-	-
**Artefactual (barbiturate lysis) lesion**					
present	1	-	3	-	-

Reported by Kevin Isaacs on 07-MAR-12 09:02:11

TABLE II
INDIVIDUAL ANIMAL REPORTS

E 15/25

Table II
Individual Animal Reports

Vehicle Main test

Animal	Tissue	Observation
R2409	ADRENAL GLANDS	Vacuolation, zona fasciculata, diffuse - grade 1 of 5 (minimal)
	KIDNEYS	Basophilic (regenerative) tubules, focal - grade 1 of 5 (minimal)
	LUNG	Inflammation, mononuclear cell, perivascular - grade 1 of 5 (minimal)
	THYROID GLAND	Heterotopic thymic tissue - unilateral
		Number of Sections less than protocol for URINARY BLADDER (1).
		The following tissues were not remarkable: BONE MARROW SECTION, BRAIN, EPIDIDYMIDES, HEART, LARGE INTESTINE: COLON, LIVER, LYMPH NODE: MESENTERIC, PEYER'S PATCHES (MALT), PROSTATE GLAND, SEMINAL VESICLE/COAGULATING G., SMALL INTESTINE: ILEUM, SPINAL CORD, SPLEEN, STOMACH: GLANDULAR, STOMACH: NON-GLANDULAR, TESTES, THYMUS, TRACHEA, URINARY BLADDER.
R2410	HEART	No atria or valves in sections
	PROSTATE GLAND	Inflammation, mononuclear cell, focal - grade 2 of 5 (slight)
	TESTES	Incomplete section
		The following tissues were not remarkable: ADRENAL GLANDS, BONE MARROW SECTION, BRAIN, EPIDIDYMIDES, KIDNEYS, LARGE INTESTINE: COLON, LIVER, LUNG, LYMPH NODE: MESENTERIC, PEYER'S PATCHES (MALT), SEMINAL VESICLE/COAGULATING G., SMALL INTESTINE: ILEUM, SPINAL CORD, SPLEEN, STOMACH: GLANDULAR, STOMACH: NON-GLANDULAR, THYMUS, THYROID GLAND, TRACHEA, URINARY BLADDER.

Reported by Kevin Isaacs on 07-MAR-12 09:02:26

KIsaacs

Table II
Individual Animal Reports

Vehicle Main test

Animal	Tissue	Observation
R2411	ADRENAL GLANDS	Vacuolation, zona fasciculata, diffuse - grade 1 of 5 (minimal)
	HEART	Inflammation, mononuclear cell, myocardial, focal - grade 1 of 5 (minimal)
	PROSTATE GLAND	Inflammation, mononuclear cell, focal - grade 2 of 5 (slight)<br>Atrophy, focal - grade 2 of 5 (slight)
	STOMACH: GLANDULAR	No pyloric region in sections
	THYMUS	Congestion/haemorrhage (agonal) - grade 1 of 5 (minimal)
		Number of Sections less than protocol for URINARY BLADDER (1).
		The following tissues were not remarkable: BONE MARROW SECTION, BRAIN, EPIDIDYMIDES, KIDNEYS, LARGE INTESTINE: COLON, LIVER, LUNG, LYMPH NODE: MESENTERIC, PEYER'S PATCHES (MALT), SEMINAL VESICLE/COAGULATING G., SMALL INTESTINE: ILEUM, SPINAL CORD, SPLEEN, STOMACH: NON-GLANDULAR, TESTES, THYROID GLAND, TRACHEA, URINARY BLADDER.
R2412	ADRENAL GLANDS	Vacuolation, zona fasciculata, diffuse - grade 1 of 5 (minimal)
	EPIDIDYMIDES	Incomplete section
	LIVER	Inflammation, mononuclear cell, focal - grade 1 of 5 (minimal)
	URINARY BLADDER	Mineralisation, adventitia, focal - grade 1 of 5 (minimal)
		The following tissues were not remarkable: BONE MARROW SECTION, BRAIN, HEART, KIDNEYS, LARGE INTESTINE: COLON, LUNG, LYMPH NODE: MESENTERIC, PEYER'S PATCHES (MALT), PROSTATE GLAND, SEMINAL VESICLE/COAGULATING G., SMALL INTESTINE: ILEUM, SPINAL CORD, SPLEEN, STOMACH: GLANDULAR, STOMACH: NON-GLANDULAR, TESTES, THYMUS, THYROID GLAND, TRACHEA.

Reported by Kevin Isaacs on 07-MAR-12 09:02:26

K Isaacs

Table II
Individual Animal Reports

Vehicle Main test

Animal	Tissue	Observation
R2413	TESTES	Incomplete section
	THYMUS	Congestion/haemorrhage (agonal) - grade 1 of 5 (minimal)
		The following tissues were not remarkable: ADRENAL GLANDS, BONE MARROW SECTION, BRAIN, EPIDIDYMIDES, HEART, KIDNEYS, LARGE INTESTINE: COLON, LIVER, LUNG, LYMPH NODE: MESENTERIC, PEYER'S PATCHES (MALT), PROSTATE GLAND, SEMINAL VESICLE/COAGULATING G., SMALL INTESTINE: ILEUM, SPINAL CORD, SPLEEN, STOMACH: GLANDULAR, STOMACH: NON-GLANDULAR, THYROID GLAND, TRACHEA, URINARY BLADDER.
R2414	ADRENAL GLANDS	Vacuolation, zona fasciculata, diffuse - grade 1 of 5 (minimal)
	TESTES	Incomplete section
	THYMUS	Congestion/haemorrhage (agonal) - grade 1 of 5 (minimal)
	THYROID GLAND	Heterotopic thymic tissue - unilateral
		The following tissues were not remarkable: BONE MARROW SECTION, BRAIN, EPIDIDYMIDES, HEART, KIDNEYS, LARGE INTESTINE: COLON, LIVER, LUNG, LYMPH NODE: MESENTERIC, PEYER'S PATCHES (MALT), PROSTATE GLAND, SEMINAL VESICLE/COAGULATING G., SMALL INTESTINE: ILEUM, SPINAL CORD, SPLEEN, STOMACH: GLANDULAR, STOMACH: NON-GLANDULAR, TRACHEA, URINARY BLADDER.

Reported by Kevin Isaacs on 07-MAR-12 09:02:26

K Isaacs

Table II
Individual Animal Reports

Vehicle Main test

Animal	Tissue	Observation
R2415	KIDNEYS	Basophilic (regenerative) tubules, focal - grade 1 of 5 (minimal)
	LUNG	Alveolar macrophages, foamy, focal - grade 1 of 5 (minimal)
	PEYER'S PATCHES (MALT)	Mineralisation, focal - grade 1 of 5 (minimal)
	THYMUS	Congestion/haemorrhage (agonal) - grade 1 of 5 (minimal)
		The following tissues were not remarkable: ADRENAL GLANDS, BONE MARROW SECTION, BRAIN, EPIDIDYMIDES, HEART, LARGE INTESTINE: COLON, LIVER, LYMPH NODE: MESENTERIC, PROSTATE GLAND, SEMINAL VESICLE/COAGULATING G., SMALL INTESTINE: ILEUM, SPINAL CORD, SPLEEN, STOMACH: GLANDULAR, STOMACH: NON-GLANDULAR, TESTES, THYROID GLAND, TRACHEA, URINARY BLADDER.
R2416	HEART	Inflammation, mononuclear cell, myocardial, focal - grade 1 of 5 (minimal)
	KIDNEYS	Basophilic (regenerative) tubules, focal - grade 1 of 5 (minimal)
	LUNG	Inflammation, mononuclear cell, perivascular - grade 2 of 5 (slight)<br>Inflammation, mononuclear cell, interstitial, - grade 2 of 5 (slight)
	URINARY BLADDER	Artefactual (barbiturate lysis) lesion
		The following tissues were not remarkable: ADRENAL GLANDS, BONE MARROW SECTION, BRAIN, EPIDIDYMIDES, LARGE INTESTINE: COLON, LIVER, LYMPH NODE: MESENTERIC, PEYER'S PATCHES (MALT), PROSTATE GLAND, SEMINAL VESICLE/COAGULATING G., SMALL INTESTINE: ILEUM, SPINAL CORD, SPLEEN, STOMACH: GLANDULAR, STOMACH: NON-GLANDULAR, TESTES, THYMUS, THYROID GLAND, TRACHEA.

Reported by Kevin Isaacs on 07-MAR-12 09:02:26

K Isaacs

Table II
Individual Animal Reports

Vehicle Main test

Animal	Tissue	Observation
R2417	KIDNEYS	Basophilic (regenerative) tubules, focal - grade 1 of 5 (minimal)
	LUNG	Inflammation, mononuclear cell, perivascular - grade 1 of 5 (minimal)<br>Inflammation, mononuclear cell, interstitial, - grade 1 of 5 (minimal)<br>Haemorrhage, intra-alveolar (agonal) - grade 1 of 5 (minimal)
		The following tissues were not remarkable: ADRENAL GLANDS, BONE MARROW SECTION, BRAIN, EPIDIDYMIDES, HEART, LARGE INTESTINE: COLON, LIVER, LYMPH NODE: MESENTERIC, PEYER'S PATCHES (MALT), PROSTATE GLAND, SEMINAL VESICLE/COAGULATING G., SMALL INTESTINE: ILEUM, SPINAL CORD, SPLEEN, STOMACH: GLANDULAR, STOMACH: NON-GLANDULAR, TESTES, THYMUS, THYROID GLAND, TRACHEA, URINARY BLADDER.
R2418	HEART	No atria in sections
	KIDNEYS	Basophilic (regenerative) tubules, focal - grade 1 of 5 (minimal)
	LUNG	Haemorrhage, intra-alveolar (agonal) - grade 1 of 5 (minimal)
		The following tissues were not remarkable: ADRENAL GLANDS, BONE MARROW SECTION, BRAIN, EPIDIDYMIDES, LARGE INTESTINE: COLON, LIVER, LYMPH NODE: MESENTERIC, PEYER'S PATCHES (MALT), PROSTATE GLAND, SEMINAL VESICLE/COAGULATING G., SMALL INTESTINE: ILEUM, SPINAL CORD, SPLEEN, STOMACH: GLANDULAR, STOMACH: NON-GLANDULAR, TESTES, THYMUS, THYROID GLAND, TRACHEA, URINARY BLADDER.

Reported by Kevin Isaacs on 07-MAR-12 09:02:26

K Isaacs

Table II
Individual Animal Reports

c2 20 mg/kg Main Test

Animal	Tissue	Observation
R2429	HEART	Inflammation, mononuclear cell, myocardial, focal - grade 1 of 5 (minimal)
	PROSTATE GLAND	Inflammation, mononuclear cell, focal - grade 1 of 5 (minimal)
	STOMACH: GLANDULAR	No pyloric region in sections
		Number of Sections less than protocol for SMALL INTESTINE: ILEUM (1), STOMACH: GLANDULAR (1).
		The following tissues were not remarkable: ADRENAL GLANDS, BONE MARROW SECTION, BRAIN, EPIDIDYMIDES, KIDNEYS, LARGE INTESTINE: COLON, LIVER, LUNG, LYMPH NODE: MESENTERIC, PEYER'S PATCHES (MALT), SEMINAL VESICLE/COAGULATING G., SMALL INTESTINE: ILEUM, SPINAL CORD, SPLEEN, STOMACH: NON-GLANDULAR, TESTES, THYMUS, THYROID GLAND, TRACHEA, URINARY BLADDER.
R2430	ADRENAL GLANDS	Vacuolation, zona fasciculata, diffuse - grade 1 of 5 (minimal)
	KIDNEYS	Basophilic (regenerative) tubules, focal - grade 1 of 5 (minimal)
	LYMPH NODE: MESENTERIC	Increased size/lymphocyte density, paracortex - grade 1 of 5 (minimal)
	SPINAL CORD	Incomplete section
		The following tissues were not remarkable: BONE MARROW SECTION, BRAIN, EPIDIDYMIDES, HEART, LARGE INTESTINE: COLON, LIVER, LUNG, PEYER'S PATCHES (MALT), PROSTATE GLAND, SEMINAL VESICLE/COAGULATING G., SMALL INTESTINE: ILEUM, SPLEEN, STOMACH: GLANDULAR, STOMACH: NON-GLANDULAR, TESTES, THYMUS, THYROID GLAND, TRACHEA, URINARY BLADDER.

Reported by Kevin Isaacs on 07-MAR-12 09:02:26

KIsaacs

Table II
Individual Animal Reports

c2 20 mg/kg Main Test

Animal	Tissue	Observation
R2431	PROSTATE GLAND	Inflammation, mononuclear cell, focal - grade 1 of 5 (minimal)
	STOMACH: GLANDULAR	No pyloric region in sections
		The following tissues were not remarkable: ADRENAL GLANDS, BONE MARROW SECTION, BRAIN, EPIDIDYMIDES, HEART, KIDNEYS, LARGE INTESTINE: COLON, LIVER, LUNG, LYMPH NODE: MESENTERIC, PEYER'S PATCHES (MALT), SEMINAL VESICLE/COAGULATING G., SMALL INTESTINE: ILEUM, SPINAL CORD, SPLEEN, STOMACH: NON-GLANDULAR, TESTES, THYMUS, THYROID GLAND, TRACHEA, URINARY BLADDER.
R2432	HEART	No atria in sections
	KIDNEYS	Basophilic (regenerative) tubules, focal - grade 1 of 5 (minimal)
	LIVER	Inflammation, mononuclear cell, focal - grade 1 of 5 (minimal)
	URINARY BLADDER	Artefactual (barbiturate lysis) lesion
		The following tissues were not remarkable: ADRENAL GLANDS, BONE MARROW SECTION, BRAIN, EPIDIDYMIDES, LARGE INTESTINE: COLON, LUNG, LYMPH NODE: MESENTERIC, PEYER'S PATCHES (MALT), PROSTATE GLAND, SEMINAL VESICLE/COAGULATING G., SMALL INTESTINE: ILEUM, SPINAL CORD, SPLEEN, STOMACH: GLANDULAR, STOMACH: NON-GLANDULAR, TESTES, THYMUS, THYROID GLAND, TRACHEA.
R2433	ADRENAL GLANDS	Vacuolation, zona fasciculata, diffuse - grade 1 of 5 (minimal)
	PEYER'S PATCHES (MALT)	Mineralisation, focal - grade 1 of 5 (minimal)
		The following tissues were not remarkable: BONE MARROW SECTION, BRAIN, EPIDIDYMIDES, HEART, KIDNEYS, LARGE INTESTINE: COLON, LIVER, LUNG, LYMPH NODE: MESENTERIC, PROSTATE GLAND, SEMINAL VESICLE/COAGULATING G., SMALL INTESTINE: ILEUM, SPINAL CORD, SPLEEN, STOMACH: GLANDULAR, STOMACH: NON-GLANDULAR, TESTES, THYMUS, THYROID GLAND, TRACHEA, URINARY BLADDER.

Reported by Kevin Isaacs on 07-MAR-12 09:02:26

KIsaacs

Table II
Individual Animal Reports

c2 20 mg/kg Main Test

Animal	Tissue	Observation
R2434	ADRENAL GLANDS	Vacuolation, zona fasciculata, diffuse - grade 1 of 5 (minimal)
	THYMUS	Congestion/haemorrhage (agonal) - grade 1 of 5 (minimal)
		Number of Sections less than protocol for BRAIN (2).
		The following tissues were not remarkable: BONE MARROW SECTION, BRAIN, EPIDIDYMIDES, HEART, KIDNEYS, LARGE INTESTINE: COLON, LIVER, LUNG, LYMPH NODE: MESENTERIC, PEYER'S PATCHES (MALT), PROSTATE GLAND, SEMINAL VESICLE/COAGULATING G., SMALL INTESTINE: ILEUM, SPINAL CORD, SPLEEN, STOMACH: GLANDULAR, STOMACH: NON-GLANDULAR, TESTES, THYROID GLAND, TRACHEA, URINARY BLADDER.
R2435	ADRENAL GLANDS	Vacuolation, zona fasciculata, diffuse - grade 1 of 5 (minimal)
	HEART	No atria in sections
	LIVER	Lipidosis, tension - grade 1 of 5 (minimal)
	PROSTATE GLAND	Inflammation, mononuclear cell, focal - grade 1 of 5 (minimal)
	THYROID GLAND	Heterotopic thymic tissue - unilateral
	URINARY BLADDER	Artefactual (barbiturate lysis) lesion
		The following tissues were not remarkable: BONE MARROW SECTION, BRAIN, EPIDIDYMIDES, KIDNEYS, LARGE INTESTINE: COLON, LUNG, LYMPH NODE: MESENTERIC, PEYER'S PATCHES (MALT), SEMINAL VESICLE/COAGULATING G., SMALL INTESTINE: ILEUM, SPINAL CORD, SPLEEN, STOMACH: GLANDULAR, STOMACH: NON-GLANDULAR, TESTES, THYMUS, TRACHEA.

Reported by Kevin Isaacs on 07-MAR-12 09:02:26

KIsaacs

Table II
Individual Animal Reports

c2 20 mg/kg Main Test

Animal	Tissue	Observation
R2436	ADRENAL GLANDS	Vacuolation, zona fasciculata, diffuse - grade 1 of 5 (minimal)
	KIDNEYS	Basophilic (regenerative) tubules, focal - grade 1 of 5 (minimal)<br>Scar, cortex/medulla, focal - grade 1 of 5 (minimal)
	PROSTATE GLAND	Inflammation, mononuclear cell, focal - grade 1 of 5 (minimal)
		The following tissues were not remarkable: BONE MARROW SECTION, BRAIN, EPIDIDYMIDES, HEART, LARGE INTESTINE: COLON, LIVER, LUNG, LYMPH NODE: MESENTERIC, PEYER'S PATCHES (MALT), SEMINAL VESICLE/COAGULATING G., SMALL INTESTINE: ILEUM, SPINAL CORD, SPLEEN, STOMACH: GLANDULAR, STOMACH: NON-GLANDULAR, TESTES, THYMUS, THYROID GLAND, TRACHEA, URINARY BLADDER.
R2437	ADRENAL GLANDS	Vacuolation, zona fasciculata, diffuse - grade 1 of 5 (minimal)
	HEART	No atria in sections
	KIDNEYS	Hyaline droplets, proximal tubules - grade 1 of 5 (minimal)
	PROSTATE GLAND	Inflammation, mononuclear cell, focal - grade 1 of 5 (minimal)
	SEMINAL VESICLE/COAGULATING G.	Artefactual (barbiturate lysis) lesion
	URINARY BLADDER	Artefactual (barbiturate lysis) lesion
		The following tissues were not remarkable: BONE MARROW SECTION, BRAIN, EPIDIDYMIDES, LARGE INTESTINE: COLON, LIVER, LUNG, LYMPH NODE: MESENTERIC, PEYER'S PATCHES (MALT), SMALL INTESTINE: ILEUM, SPINAL CORD, SPLEEN, STOMACH: GLANDULAR, STOMACH: NON-GLANDULAR, TESTES, THYMUS, THYROID GLAND, TRACHEA.

Reported by Kevin Isaacs on 07-MAR-12 09:02:26

K Isaacs

Table II
Individual Animal Reports

c2 20 mg/kg Main Test

Animal	Tissue	Observation
R2438		Not remarkable<br><br>The following tissues were not remarkable: ADRENAL GLANDS, BONE MARROW SECTION, BRAIN, EPIDIDYMIDES, HEART, KIDNEYS, LARGE INTESTINE: COLON, LIVER, LUNG, LYMPH NODE: MESENTERIC, PEYER'S PATCHES (MALT), PROSTATE GLAND, SEMINAL VESICLE/COAGULATING G., SMALL INTESTINE: ILEUM, SPINAL CORD, SPLEEN, STOMACH: GLANDULAR, STOMACH: NON-GLANDULAR, TESTES, THYMUS, THYROID GLAND, TRACHEA, URINARY BLADDER.

Reported by Kevin Isaacs on 07-MAR-12 09:02:26

KIsaacs

E 25/25.

I C P Firefly Pty Ltd

ACN 071 626 358

PO Box 6198, Alexandria NSW 2015 Australia
TEL: 61 2 9310 3899 FAX: 61 2 9310 4889 EMAIL: info@icpfirefly.com.au WEBSITE: www.icpfirefly.com.au

Appendix F: Protocol/Study Plan 1-29

The bright light of
certainty

I C P Firefly Pty Ltd

ACN 071 626 358

PO Box 6198, Alexandria NSW 2015 Australia
TEL: 61 2 9310 3899 FAX: 61 2 9310 4889 EMAIL: info@icpfirefly.com.au WEBSITE: www.icpfirefly.com.au

EXPERIMENTAL PROTOCOL AND STUDY PLAN

ICPQN1035.B

REPEATED DOSE 28-DAY ORAL TOXICITY MAIN STUDY (OECD 407) OF c2 IN SPRAGUE DAWLEY RATS WITH A RECOVERY PERIOD

ST GEORGE CLINICAL SCHOOL

TABLE OF CONTENTS

Page No

1.0. IDENTIFICATION OF THE STUDY, TEST ITEM AND REFERENCE ITEM **4**

1.1. Study title 4

1.2. Nature and Purpose of the Study 4

1.3. Identification of test item by code or name 4

1.4. Reference item to be used 4

1.5. Vehicle 4

1.6. GLP Compliance 4

2.0. INFORMATION CONCERNING THE SPONSOR AND THE TEST FACILITY **4**

2.1. Sponsor 4

2.1.1. Name *4*

2.1.2. Address *4*

2.1.3. Sponsor contact details *4*

2.2. Test Facility 4

2.3. Study Director 5

2.4. Lead Quality Assurance 5

2.5. Test Site 1 5

2.5.1. Phase of study *5*

2.6. Test Site 2 5

2.6.1. Principal Investigator *5*

2.6.2. Test Site QA *5*

2.6.3. Phase of study *5*

3.0. DATES **5**

3.1. Start date 5

3.2. Completion date 5

4.0. TEST METHODS **5**

4.1. Procedures/methods 5

4.2. Ethics approval number 5

5.0. ISSUES **6**

5.1. Principle of the test method 6

5.2. Justification for selection of the test system 6

5.3. Characterisation of the biological test system 6

5.3.1. Species *6*

5.3.2. Strain *6*

5.3.3. Substrain *6*

5.3.4. Source of Supply *6*

5.3.5. Number of Animals *6*

5.3.6. Bodyweight range *6*

5.3.7. Sex *6*

5.3.8. Age *6*

5.3.9. Housing and feeding conditions *6*

TABLE OF CONTENTS

		Page No
	5.3.10. Animal preparation	*7*
	5.3.11. Test item preparation and stability	*7*
5.4.	Method of administration	7
	5.4.1. Dose	*7*
	5.4.2. Concentration, volume	*7*
	5.4.3. Route of administration	*7*
	5.4.4. Justification for method of administration	*7*
	5.4.5. Frequency of administration	*7*
	5.4.6. Duration of administration/application	*7*
	5.4.7. Group allocation	*7*
5.5.	Body Weights	7
5.6.	Feed consumption	7
5.7.	Clinical observations	8
5.8.	Detailed clinical observations	8
5.9.	Functional observations	8
5.10.	Urine analysis	8
5.11.	Blood analysis	8
	5.11.1. Haematology	*8*
	5.11.2. Biochemistry	*8*
5.12.	Gross pathology	9
5.13.	Histopathology	10
5.14.	Statistical methods	10
5.15.	Reporting	11
5.16.	Forms required	11
5.17.	Personnel involved in the study	12
**6.0.**	**WORK SCHEDULE**	**13**
	6.1. Deviations	13
**7.0.**	**RECORDS**	**28**

1.0. IDENTIFICATION OF THE STUDY, TEST ITEM AND REFERENCE ITEM

1.1. Study title

Repeated dose 28-day oral toxicity main study (OECD 407) of c2 in Sprague Dawley rats with a recovery period.

1.2. Nature and Purpose of the Study

The aim of this study is to determine the toxic effects of the test item following repeated oral administration every 3 days for 28 days, at two dose levels. A control group is treated with the vehicle only. Clinical examinations are made daily throughout the study. The animals are weighed weekly and the main groups sacrificed after 28 days, 24 hours after the last dose. Recovery groups for the high dose and vehicle control are retained for a further 14 days to determine any reversibility of potential toxic effects.

1.3. Identification of test item by code or name

TI/458 c2

1.4. Reference item to be used

Not applicable.

1.5. Vehicle

1% Carboxymethylcellulose.

1.6. GLP Compliance

The study will be conducted in compliance with the OECD principles of Good Laboratory Practice (GLP).

2.0. INFORMATION CONCERNING THE SPONSOR AND THE TEST FACILITY

2.1. Sponsor

2.1.1. Name

St George Clinical School

2.1.2. Address

Clinical Sciences (WR Pitney) Building
Short Street
St George Hospital
Kogarah NSW 2217
AUSTRALIA.

2.1.3. Sponsor contact details

Dr Kieran Scott
kieran.scott@unsw.edu.au
Phone: 0419 143 647
Fax: (02) 8347 1756

2.2. Test Facility

ICP Firefly Pty Ltd
129 Queen Street
Beaconsfield NSW 2015
AUSTRALIA.

2.3. Study Director

Fiona Brook
129 Queen Street
Beaconsfield NSW 2015
AUSTRALIA
Phone: +61 2 9310 3899
Fax: +61 2 9310 4889
Email: Fiona@icpfirefly.com.au

2.4. Lead Quality Assurance

Edmund Ho
Phone: (02) 9310 3899
Fax: (02) 9310 4889
Email: Edmund@icpfirefly.com.au

2.5. Test Site 1

IDEXX Laboratories
The Metro Centre
Unit 20, 38-46 South Street
Rydalmere NSW 2116
AUSTRALIA.

2.5.1. Phase of study

Blood analysis, submission of raw data for inclusion in the final report.

2.6. Test Site 2

14 Rossett Park Road
Harrogate
North Yorkshire HG2 9NP
UNITED KINGDOM

2.6.1. Principal Investigator

Dr Kevin Isaacs.

2.6.2. Test Site QA

Mrs Hazel Isaacs.

2.6.3. Phase of study

Histopathology slide examination and GLP compliant report for inclusion in the final report.

3.0. DATES

3.1. Start date

to be determined

3.2. Completion date

to be determined

4.0. TEST METHODS

4.1. Procedures/methods

ICP Firefly Experimental protocol ICPQN1035

4.2. Ethics approval number

ICPE134

5.0. ISSUES

5.1. Principle of the test method

The test item is administered once every three days for 28 days (10 doses), by oral gavage to three groups of rats at two dose levels. Two control groups are administered with the vehicle only. The main groups (high dose, low dose and vehicle control) are sacrificed on Day 29. The additional two recovery groups (high dose and vehicle control) are observed for a further 14 days without treatment to investigate reversibility of any toxicity observed and persistence or delayed occurrence of toxicity. The recovery animals (high dose and vehicle control) are sacrificed on Day 43.

Animals are weighed weekly and observed daily for clinical effects. Food consumption is determined weekly by sex/group. Detailed clinical examinations (weekly) are performed on all animals. Functional observations are performed in the last week of treatment. Blood samples and urine samples are analysed on Day 29 for the main groups and Day 43 for the recovery groups.

A gross necropsy is performed on all animals and the liver, kidneys, adrenals, testes, spleen, thymus, heart, epididymis, prostate + seminal vesicles with coagulating glands as whole and brain are weighed wet following dissection. All tissues are transferred to 10% formalin. Histopathology is performed on all animals from the high dose and vehicle control of the main study on the following organs: adrenal glands, bone marrow, brain (cerebrum, cerebellum, brain stem), epididymis, heart, kidney, large intestine: colon, liver, lung, lymph nodes, prostate, seminal vesicles with coagulating glands, small intestine: ileum (including Peyer's patches), spinal cord, spleen, stomach, thymus, thyroid gland, trachea, testes and urinary bladder.

5.2. Justification for selection of the test system

The rat is used since it is widely accepted and recognised by international guidelines as an appropriate experimental model for toxicity testing.

5.3. Characterisation of the biological test system

5.3.1. Species
Specific pathogen free (SPF) outbred albino rat.

5.3.2. Strain
Sprague Dawley (SD).

5.3.3. Substrain
Not applicable.

5.3.4. Source of Supply
Animal Resources Centre (ARC), WA.

5.3.5. Number of Animals
40 animals.

5.3.6. Bodyweight range
200 - 220 grams.

5.3.7. Sex
Male

5.3.8. Age
Young adult.

5.3.9. Housing and feeding conditions
Animals are group housed (up to 5 per cage) according to SOP FAC-001. The animals are fed on a diet of mouse & rat pellets and provided tap water *ad libitum.*

Lighting is artificial and set to a 12 hour light/12 hour dark cycle. The temperature of the holding rooms is maintained at 22°C ± 3°C and the relative humidity (RH) between 30 - 70%.

5.3.10. Animal preparation

Healthy young animals are assigned to the treatment groups without bias. Animals are allowed to acclimatise to the laboratory conditions for at least 5 days prior to commencement of treatment.

5.3.11. Test item preparation and stability

The test item is prepared at the required concentration prior to administration.
The test item will be handled and prepared according to the instructions of the Sponsor.

5.4. Method of administration

5.4.1. Dose

Two doses (Low and High) will be used for the test item (see Table 1). The control group will be given an equivalent volume of the vehicle.

5.4.2. Concentration, volume

Concentrations will be determined when the doses are selected. A maximum volume of 10 mL/kg will be used.

5.4.3. Route of administration

Oral.

5.4.4. Justification for method of administration

The oral route of administration is used since this is the intended clinical route.

5.4.5. Frequency of administration

The test item is administered once every 3 days for 28 days in a single dose to each animal (total 10 doses).

5.4.6. Duration of administration/application

The test item is administered in a single dose by oral gavage.

5.4.7. Group allocation

Table 1 - Animal allocation

Group No	Number of animals/sex	Unique animal number	Dose (mg/kg)	Concentration	Days of treatment	Days of observation
**Main Groups**						
1	10 males	R2409 – R2418	Vehicle	-	28	28
2	10 males	R2419 – R2428	Low<br>1 mg/kg	0.01% w/w	28	28
3	10 males	R2429 – R2438	High<br>20 mg/kg	0.2% w/w	28	28
**Recovery Groups**						
4	5 males	R2439 – R2443	Vehicle	-	28	42
5	5 males	R2444 – R2448	High<br>20 mg/kg	0.2% w/w	28	42

5.5. Body Weights

Body weights will be determined weekly and at sacrifice (Days 1, 8, 15, 22, 28 for all Groups, Day 29 for Groups 1-3, Days 36, 42 and 43 for Groups 4-5).

5.6. Feed consumption

Feed consumption (grams/animal/week) will be determined weekly.

5.7. Clinical observations

General clinical examinations are made once daily following dosing. Changes in skin, fur and eyes, and mucous membranes, and also respiratory, circulatory, autonomic and central nervous systems, and somatomotor activity and behaviour patterns are monitored and recorded. Particular attention is paid to the observation of tremors, convulsions, salivation, diarrhoea, lethargy, sleep and coma. The time of death is recorded as accurately as possible (if death occurs). During the observation period animals suffering severe pain or distress will be euthanased. This includes animals showing severe motor depression, severe respiratory distress, hypothermia, convulsions and coma.

5.8. Detailed clinical observations

Detailed clinical examinations are performed at pre-treatment, then weekly thereafter in all animals. The following parameters are assessed: posture, gait, clonic convulsion, tonic convulsion, biting, removing rat from cage, handling rat in hand, lacrimation, fur appearance, salivation, defecations, urination, grooming, palpebral closure, pupil response, eye, respiration, behaviour, and rearing.

5.9. Functional observations

A functional observation battery is performed in the fourth week of the study to assess sensory reactivity to stimuli of different types (auditory, visual and proprioceptive); grip strength and motor activity on all animals.

5.10. Urine analysis

Urinalysis is performed on all animals upon sacrifice on Day 29 for the main groups and on Day 43 for the recovery groups. The following parameters are measured: glucose, bilirubin, ketone, specific gravity, blood, pH, protein, urobilinogen, nitrite and leucocytes.

5.11. Blood analysis

Upon sacrifice (Day 29 for the main groups and Day 43 for the recovery groups), approximately 4 mL of blood is withdrawn from all animals under anaesthesia by cardiac puncture for haematology and biochemistry analyses.

5.11.1. Haematology

The following parameters are measured: White blood cells (WBC), Lymphocytes (Lym), Monocytes (Mon), Neutrophil (Neut), Eosinophil (Eos), Basophil (Baso), Red blood cell (RBC), Mean corpuscular volume (MCV), Haematocrit (Hct), Mean corpuscular haemoglobin (MCH), Mean corpuscular haemoglobin concentration (MCHC), Haemoglobin (Hb), Platelets (PLT), Mean platelet volume (MPV).

5.11.2. Biochemistry

The following parameters are measured: Glucose (Glu), Sodium (Na), Potassium (K), Na:K ratio, Chloride (Cl), Calcium (Ca), Phosphate (Phos), Ca:P ratio, Urea, Creatinine (Cre), Total protein, (TP), Albumin (ALB), Globulin (Glob), Albumin/Globulin ratio (A:G), total bilirubin (Tbil), Alanine aminotransferase (ALT), Aspartate aminotransferase (AST), Alkaline phosphatase (ALP), Creatine Kinase (CK), Gamma-glutamyl-transaminase (GGT), Triglyceride (Trig), Cholesterol (Chol).

5.12. Gross pathology

At the completion of the experiment, all animals are subjected to a full gross necropsy, which includes careful examination of the external surface of the body, all orifices, and the cranial, thoracic and abdominal cavities and their contents. The liver, kidneys, adrenals, testes, spleen, thymus, heart, epididymis, prostate + seminal vesicles with coagulating glands as whole and brain are weighed wet.

The following organs/tissues are transferred to 10% formalin:

Group	Tissues examined for gross pathology
All groups	all gross lesions,<br>adrenal glands<br>bone marrow,<br>brain<br>epididymis,<br>eye*,<br>heart,<br>kidney,<br>large intestine: colon,<br>liver,<br>lung,<br>lymph nodes,<br>peripheral nerve (sciatic),<br>prostate + seminal vesicles,<br>small intestine: ileum with Peyer's patches,<br>spinal cord,<br>spleen,<br>stomach,<br>thymus,<br>thyroid gland,<br>trachea,<br>testes**,<br>urinary bladder.

*, The eyes are placed in Davidson fixative (DF) for 48 hours, and then transferred to 10% formalin.

**, The testes are place in modified DF for 48 hours and then transferred to 10% formalin.

All carcasses are retained in 10% formalin.

5.13. Histopathology

The following organs are processed for histopathology (see table below) for Groups 1 and 3, and all other tissues and the carcasses are kept in formalin for up to 6 months following sacrifice. The organs from Groups 2, 4 and 5 are retained for possible further investigation.

Group	Tissues for histopathology
Groups 1 and 3	Gross lesions,<br>adrenal glands<br>bone marrow,<br>brain (cerebrum, cerebellum, brain stem),<br>epididymis,<br>heart,<br>kidney,<br>large intestine: colon,<br>liver,<br>lung,<br>lymph nodes,<br>prostate,<br>seminal vesicles with coagulating glands,<br>small intestine: ileum (including Peyer's patches),<br>spinal cord,<br>spleen,<br>stomach,<br>thymus,<br>thyroid gland,<br>trachea,<br>testes,<br>urinary bladder.

5.14. Statistical methods

The following data are presented in Excel spreadsheets:

- Body weights
- Clinical observations (daily)
- Food consumption
- Detailed observation
- Functional observation
- Gross necropsy (observations)
- Organ weights
- Haematology/biochemistry/urinalysis

The following analyses are conducted:

- Body weights (percentage weight gain, unpaired T-test vs Vehicle control)
- Food consumption - (comparative analysis)
- Organ weights (unpaired T-test vs Vehicle control)

5.15. Reporting

The study is presented as a report, which will include the experimental procedures and conditions, the results and analysis of data with summary and conclusions and all relevant references.

Quality Assurance shall conduct inspections to determine whether the study is conducted in accordance with the OECD Principles of Good Laboratory Practice.

The report includes the following:

STUDY INFORMATION AND COMPLIANCE STATEMENTS
TEST ITEM
VEHICLE
TEST ANIMALS
TEST CONDITIONS
METHODS
RESULTS
CONCLUSIONS

5.16. Forms required

Form reference	Title
LAB-096/f1	Test item use
LAB-096/f2	Test item information
LAB-096/f3	Test item preparation
LAB-027/f1	Body weight on receipt
LAB-021/f1	Acclimatisation period clinical observations
LAB-027/f2	Bodyweights
LAB-028/f2	Individual clinical observations - daily
LAB-038/f1	Gross pathology
LAB-024/f2	Feed intake
LAB-029/f1	Detailed clinical examination
LAB-031/f1	Dose record
LAB-030/f1	Functional observation battery
LAB-090/f1	Urinalysis
ADM-010/f1	Chain of custody
ADM-013/f1	IDEXX GLP checklist
QD62	Cassette register
LAB-147/f1	Histopathology slide cutting
LAB-156/f1	Tissue processor run sheet
LAB-158/f2	Slide staining log
ADM-010/f4	Histopathology slides summary

5.17. Personnel involved in the study

Name	Job Title	Qualification
**Dr E. Rozinova**	Research Manager	**PhD**
**Ms F. Brook**	Senior Research Officer/ Study Director	**BSc**
**Dr R. Miller**	Specialist Veterinary Pathologist	**BVSc, MSc, PhD, DACVP, MACVSc**
**Dr K. Isaacs**	Consultant in Toxicological Pathology/ Principal Investigator	**MA, VetMB, MRCVS, FRCPath**
**Dr. Terry Rothwell**	Specialist Veterinary Pathologist	**BVSc, PhD, DVSc, MACVS**
**Mr E. Ho**	QA Manager	**BAppSc, MBus**
**Dr I. Meyer-Carrive**	Managing Director	**BSc, MSc, PhD**

6.0. WORK SCHEDULE

It is the responsibility of the nominated Study Director to ensure ICP Firefly SOPs and Protocols are adhered to. Any deviations and amendments shall be documented.

6.1. Deviations

Date	Deviation	Impact	Study Director/ Principal investigator
16/2/12	Histopathology processing: Initial sections from block N°s 10083, 10120, 10126, 10140 and 10210 were not considered to be of good quality and re-cuts were made. New slide numbers allocated were 10083-1, 10120-1, 10126-1, 10140-1, and 10210-1, respectively. Cassette N°s 10185 + 10257 did not process satisfactorily. New fixed tissue was prepared: cassette N°s 10325 + 10326, respectively. #10326 did not include brain stem section, as there was no remaining tissue.	No adverse impact.	[signature]
17/2/12	Histopathology processing: Initial sections from block N°s 10266, 10279, 10280, 10294, 10308, 10321 and 10322 were not considered to be of good quality and re-cuts were made. New slide N°s allocated were 10266-1, 10279-1, 10280-1, 10294-1, 10308-1, 10321-1, and 10322-1, respectively. Cassette N° 10132 – Thyroid tissue missing after embedding. Slide No 10327 prepared from new fixed tissue		[signature]

EXPERIMENTAL DATES

Day		Date	Dec. 2011.	Site	Beaconsfield
**Procedure:**<br>**Study preparations**					**Operator**
1. Prepare raw data forms as detailed in Section 5.16.<br>2. Allocate animal housing and prepare cages and cage cards					
**Study Director**					[signature] 20/12/11

Day		Date	20/12/11	Site	Beaconsfield
**Procedure:**<br>**Animal acquisition and preparations**					**Operator**
1. Order animals from ARC (Order No. 2988).<br>2. Ensure that the following are available prior to commencement of the test:<br>• Test item and vehicle<br>• Feed (DOM: 02/11/11 Exp: 02/05/12)<br>• Calibrated balances<br>• Fixative (10% formalin)<br>• Davidson's fixative and modified Davidson's fixative					
**Study Director**					[signature] 20/12/11.

Day		Date	06/1/12	Site	Beaconsfield
**Procedure:**<br>**Receipt of animals / acclimatisation period**					**Operator**
1. Animals arrive. Animal Holding Room......1..........					✓
2. Verify that animal specifications conform to the ordered requirements as per SOP LAB-021.					✓
3. Number and weigh the animals according to SOP LAB-027 and record on form LAB-027/f1.					✓
4. House animals in groups of up to 5 per cage according to SOP FAC-001 and Table 1.					✓
5. Assign the unique identification numbers to each animal and mark the tail with different coloured permanent marker pens for each group as per SOP LAB-022.					✓
6. Label cages with Study Director's name, date, identification of animal, study number, treatment group and Animal Ethics approval number.					✓
7. Allow the animals to acclimatise to the study environment.					✓
8. Observe animals daily according to SOP LAB-028 and record observations on form LAB-021/f1.					[initials] 16/1/12
**Study Director**					[signature] 6/1/12

Day		Date	10/1/12	Site	Beaconsfield

Procedure: Pre-treatment detailed clinical observations	Operator
1. Perform a detailed clinical examination on all animals according to SOP LAB-029 and record on form LAB-029/f1.	✓ [illegible] 10/1/12
Study Director	10/01/12

Day	1	Date	11/1/12 – 12/1/12	Site	Beaconsfield

Procedure: Body weights, dosing, observation, feed intake	Operator
1. Prepare the test item at the required concentration and document on form LAB-096/f3	✓
2. Weigh all animals according to SOP LAB-027, calculate the individual dose for each animal and record on form LAB-027/f2.	✓ [illegible]
3. Place a fresh known amount of feed in the hoppers of each cage and record on form LAB-024/f2	✓ [illegible]
4. Dose the animals orally according to SOP LAB-032 and Table 1 and record the time of administration on form LAB-031/f1.	11/01/12
5. Observe each animal carefully for signs of toxicity according to SOP LAB-028 and record observations on form LAB-028/f2.	[illegible] 11/1/12
Study Director	11/1/12

[illegible] 12/1/12 12/1/12

Day	2-3	Date	12/1/12 – 14/1/12	Site	Beaconsfield

Procedure: Observation	Operator
1. Observe each animal carefully for signs of toxicity according to SOP LAB-028 and record observations on form LAB-028/f2.	✓
2. Weigh any feed added and record on form LAB-024/f2.	✓ 12/1/12
Study Director	12/1/12

13/1/12 14/1/12

Day	4	Date	14/1/12 + 15/1/12	Site	Beaconsfield

Procedure: Dosing and observation	Operator
1. Prepare the test item at the required concentration and document on form LAB-096/f3	✓
2. Dose the animals orally according to SOP LAB-032 and Table 1 and record the time of administration on form LAB-031/f1.	✓
3. Observe each animal carefully for signs of toxicity according to SOP LAB-028 and record observations on form LAB-028/f2.	✓
4. Weigh any feed added and record on form LAB-024/f2.	✓ 14/1/12
Study Director	14/1/12

15/1/12

Day	5	Date	16/1/12	Site	Beaconsfield

Procedure: Detailed clinical observation and observation	Operator
1. Perform a detailed clinical examination on all animals according to SOP LAB-029 and record on form LAB-029/f1.	EQ 16/1/12
2. Observe each animal carefully for signs of toxicity according to SOP LAB-028 and record observations on form LAB-028/f2.	✓ [illegible]
3. Weigh any feed added and record on form LAB-024/f2.	
**Study Director**	[illegible] 16/1/12

Day	6	Date	16/1/12 + 17/1/12	Site	Beaconsfield

Procedure: Observation	Operator
1. Observe each animal carefully for signs of toxicity according to SOP LAB-028 and record observations on form LAB-028/f2.	✓ [illegible] 16/1/12
2. Weigh any feed added and record on form LAB-024/f2.	—
**Study Director**	[illegible] 16/1/12

[illegible] 17/1/12

Day	7	Date	17/1/12 + 18/1/12	Site	Beaconsfield

Procedure: Dosing and observation	Operator
1. Prepare the test item at the required concentration and document on form LAB-096/f3	✓ [illegible] 17/1/12
2. Dose the animals orally according to SOP LAB-032 and Table 1 and record the time of administration on form LAB-031/f1.	✓
3. Observe each animal carefully for signs of toxicity according to SOP LAB-028 and record observations on form LAB-028/f2.	✓
4. Weigh any feed added and record on form LAB-024/f2.	– [illegible] 17/1/12
**Study Director**	[illegible] 17/1/12

[illegible] 18/1/12

Day	8	Date	18/1/12 + 19/1/12	Site	Beaconsfield

Procedure: Bodyweights, observation and feed intake	Operator
1. Weigh all animals according to SOP LAB-027, calculate the individual dose for each animal and record on form LAB-027/f2.	✓
2. Weigh remaining feed in hoppers and record on form LAB-024/f2.	✓
3. Place a fresh known amount of feed in the hoppers of each cage and record on form LAB-024/f2	✓
4. Observe each animal carefully for signs of toxicity according to SOP LAB-028 and record observations on form LAB-028/f2.	✓ 18/1/12
**Study Director**	18/1/12

19/1/12

Day	9	Date	19/1/12 + 20/1/12	Site	Beaconsfield

Procedure: Observation	Operator
1. Observe each animal carefully for signs of toxicity according to SOP LAB-028 and record observations on form LAB-028/f2.	✓
2. Weigh any feed added and record on form LAB-024/f2.	✓ 19/1/12
**Study Director**	19/1/12

Day	10	Date	20/1/12 + 21/1/12	Site	Beaconsfield

Procedure: Dosing and observation	Operator
1. Prepare the test item at the required concentration and document on form LAB-096/f3	✓
2. Dose the animals orally according to SOP LAB-032 and Table 1 and record the time of administration on form LAB-031/f1.	✓
3. Observe each animal carefully for signs of toxicity according to SOP LAB-028 and record observations on form LAB-028/f2.	✓ 20/1/12
4. Weigh any feed added and record on form LAB-024/f2.	
**Study Director**	20/1/12

21/1/12

Day	11	Date	21/1/12 + 22/1/12	Site	Beaconsfield

Procedure: Detailed clinical observation and observation	Operator
1. Perform a detailed clinical examination on all animals according to SOP LAB-029 and record on form LAB-029/f1. – 23/1/12	✓ 23/1/12
2. Observe each animal carefully for signs of toxicity according to SOP LAB-028 and record observations on form LAB-028/f2.	✓
3. Weigh any feed added and record on form LAB-024/f2.	– 21/1/12
**Study Director**	21/1/12

22/1/12

Day	12	Date	22/1/12 + 23/1/12	Site	Beaconsfield

Procedure: Observation	Operator
1. Observe each animal carefully for signs of toxicity according to SOP LAB-028 and record observations on form LAB-028/f2.	✓
2. Weigh any feed added and record on form LAB-024/f2.	– 22/1/12
**Study Director**	22/1/12

23/1/12

Day	13	Date	23/1/12 + 24/1/12	Site	Beaconsfield

Procedure: Dosing and observation	Operator
1. Prepare the test item at the required concentration and document on form LAB-096/f3	✓
2. Dose the animals orally according to SOP LAB-032 and Table 1 and record the time of administration on form LAB-031/f1.	✓
3. Observe each animal carefully for signs of toxicity according to SOP LAB-028 and record observations on form LAB-028/f2.	✓
4. Weigh any feed added and record on form LAB-024/f2.	– 23/1/12
**Study Director**	23/1/12

24/1/12

Day	14	Date	24/1/12 + 25/1/12	Site	Beaconsfield

Procedure: Observation	Operator
1. Observe each animal carefully for signs of toxicity according to SOP LAB-028 and record observations on form LAB-028/f2.	✓
2. Weigh any feed added and record on form LAB-024/f2.	– 24/1/12
**Study Director**	24/1/12

25/1/12

Day	15	Date	25/1/12 + 26/1/12	Site	Beaconsfield

Procedure:<br>Bodyweights, observation and feed intake	Operator
1. Weigh all animals according to SOP LAB-027, calculate the individual dose for each animal and record on form LAB-027/f2.	✓
2. Weigh remaining feed in hoppers and record on form LAB-024/f2.	✓
3. Place a fresh known amount of feed in the hoppers of each cage and record on form LAB-024/f2	✓
4. Observe each animal carefully for signs of toxicity according to SOP LAB-028 and record observations on form LAB-028/f2.	✓ 25/1/12
**Study Director**	25/1/12

Day	16	Date	26/1/12 + 27/1/12	Site	Beaconsfield

Procedure:<br>Dosing and observation	Operator
1. Prepare the test item at the required concentration and document on form LAB-096/f3	✓
2. Dose the animals orally according to SOP LAB-032 and Table 1 and record the time of administration on form LAB-031/f1.	✓
3. Observe each animal carefully for signs of toxicity according to SOP LAB-028 and record observations on form LAB-028/f2.	✓
4. Weigh any feed added and record on form LAB-024/f2.	26/1/12
**Study Director**	26/1/12

27/1/12

Day	17	Date	27/1/12 + 28/1/12	Site	Beaconsfield

Procedure:<br>Observation	Operator
1. Observe each animal carefully for signs of toxicity according to SOP LAB-028 and record observations on form LAB-028/f2.	✓
2. Weigh any feed added and record on form LAB-024/f2.	27/1/12
**Study Director**	27/1/12

28/1/12

Day	18	Date	28/1/12 + 29/1/12	Site	Beaconsfield

Procedure:<br>Detailed clinical observation and observation	Operator
1. Perform a detailed clinical examination on all animals according to SOP LAB-029 and record on form LAB-029/f1. 30/01/12	✓ 30/1/12
2. Observe each animal carefully for signs of toxicity according to SOP LAB-028 and record observations on form LAB-028/f2.	✓ 28/1/12
**Study Director**	28/1/12

29/1/12

Day	19	Date	29/1/12 + 30/1/12	Site	Beaconsfield
**Procedure:**<br>**Dosing and observation**					**Operator**
1. Prepare the test item at the required concentration and document on form LAB-096/f3					✓
2. Dose the animals orally according to SOP LAB-032 and Table 1 and record the time of administration on form LAB-031/f1.					✓
3. Observe each animal carefully for signs of toxicity according to SOP LAB-028 and record observations on form LAB-028/f2.					✓ [illegible] 29/1/12
4. Weigh any feed added and record on form LAB-024/f2.					–
**Study Director**					[illegible] 29/1/12

[illegible] 30/1/12

Day	20-21	Date	30/1/12 - 31/1/12 + 31/1/12 - 1/2/12	Site	Beaconsfield
**Procedure:**<br>**Observation**					**Operator**
1. Observe each animal carefully for signs of toxicity according to SOP LAB-028 and record observations on form LAB-028/f2.					✓
2. Weigh any feed added and record on form LAB-024/f2.					– [illegible] 30/1/12
**Study Director**					[illegible] 30/1/12

[illegible] 31/1/12

Day	22	Date	1/2/12 + 2/2/12	Site	Beaconsfield
**Procedure:**<br>**Bodyweights, dosing, observation and feed intake**					**Operator**
1. Prepare the test item at the required concentration and document on form LAB-096/f3					✓
2. Weigh all animals according to SOP LAB-027, calculate the individual dose for each animal and record on form LAB-027/f2.					✓
3. Weigh remaining feed in hoppers and record on form LAB-024/f2.					✓
4. Place a fresh known amount of feed in the hoppers of each cage and record on form LAB-024/f2					✓
5. Dose the animals orally according to SOP LAB-032 and Table 1 and record the time of administration on form LAB-031/f1.					✓
6. Observe each animal carefully for signs of toxicity according to SOP LAB-028 and record observations on form LAB-028/f2.					✓ [illegible] 1/2/12
**Study Director**					[illegible] 1/2/12

[illegible] 2/2/12

Day	23-24	Date	2/2/12 - 3/2/12 + 3/2/12 - 4/2/12	Site	Beaconsfield
**Procedure:** **Observation**					**Operator**
1. Observe each animal carefully for signs of toxicity according to SOP LAB-028 and record observations on form LAB-028/f2. 2. Weigh any feed added and record on form LAB-024/f2.					✓ [illegible] 2/2/12
**Study Director**					[illegible] 2/2/12

[illegible] 3/2/12

Day	25	Date	4/2/12 + 5/2/12	Site	Beaconsfield
**Procedure:** **Dosing and observation**					**Operator**
1. Prepare the test item at the required concentration and document on form LAB-096/f3 2. Dose the animals orally according to SOP LAB-032 and Table 1 and record the time of administration on form LAB-031/f1. 3. Observe each animal carefully for signs of toxicity according to SOP LAB-028 and record observations on form LAB-028/f2. 4. Weigh any feed added and record on form LAB-024/f2.					✓ ✓ ✓ [illegible] 4/2/12
**Study Director**					[illegible] 4/2/12

[illegible] 5/2/12

[illegible] 6/2/12

Day	26	Date	~~5/2/12 +~~ 6/2/12	Site	Beaconsfield
**Procedure:** **Detailed clinical observation, functional observation and observation**					**Operator**
1. Perform a detailed clinical examination on all animals according to SOP LAB-029 and record on form LAB-029/f1. 2. Perform a functional observation battery on all animals according to SOP LAB-030 and record on form LAB030/f1. 3. Observe each animal carefully for signs of toxicity according to SOP LAB-028 and record observations on form LAB-028/f2. 4. Weigh any feed added and record on form LAB-024/f2.					✓ ✓ ✓ [illegible] 6/2/12
**Study Director**					[illegible] 6/2/12

Day	27	Date	6/2/12 + 7/2/12	Site	Beaconsfield
**Procedure:** **Observation**					**Operator**
1. Observe each animal carefully for signs of toxicity according to SOP LAB-028 and record observations on form LAB-028/f2. 2. Weigh any feed added and record on form LAB-024/f2.					✓ [illegible] 6/2/12
**Study Director**					[illegible] 6/2/12

[illegible] 7/2/12

Day	28	Date	7/2/12 + 8/2/12	Site	Beaconsfield

Procedure: Bodyweights, dosing, feed intake and observation. Feed removal (Groups 1, 2 and 3)	Operator
1. Weigh all animals according to SOP LAB-027, calculate the individual dose for each animal and record on form LAB-027/f2.	✓ [initials]
2. Weigh remaining feed in hoppers and record on form LAB-024/f2.	✓
3. Place a fresh known amount of feed in the hoppers of Groups 4 and 5 and record on form LAB-024/f2	✓
4. Dose the animals orally according to SOP LAB-032 and Table 1 and record the time of administration on form LAB-031/f1.	✓ [initials]
5. Observe each animal carefully for signs of toxicity according to SOP LAB-028 and record observations on form LAB-028/f2.	✓
6. Discard feed from Groups 1, 2 and 3 (between 4 to 5 pm).	✓
Study Director	[initials] 7/2/12

[initials] 8/2/12

Day	29	Date	8/2/12 + 9/2/12	Site	Beaconsfield

Procedure: Bodyweights and sacrifice main groups (Groups 1, 2 and 3). Observations recovery groups (Groups 4 & 5)	Operator
1. Collect urine from each animal in Groups 1, 2 and 3 and conduct urinalysis according to SOP LAB-090 and record results on form LAB-090/f1.	✓
2. Weigh all animals in Groups 1-3 according to SOP LAB-027 and record on form LAB-027/f2.	✓
3. Anaesthetise animals in Groups 1, 2 and 3 with Lethabarb by intraperitoneal (ip) injection according to SOP LAB-037.	✓
4. Withdraw approximately 4 mL of blood by cardiac puncture according to SOP LAB-035.	✓
5. Place blood into EDTA and lithium heparin tubes and mix gently.	✓
6. Examine macroscopically the external surfaces, cranial, abdominal and thoracic cavities and their contents and record observations on form LAB-038/f1.	✓
7. Dissect out and weigh the liver, kidneys, adrenals, testes, spleen, thymus, heart, thymus, epididymis, prostate + seminal vesicles with coagulating glands as whole and brain and record the wet weight on form LAB-038/f1. Place organs in 10% formalin.	✓ [initials] 8/2/12
8. Dissect out and place in formalin all gross lesions, bone marrow, eye, large intestine: colon, lung, lymph nodes, peripheral nerve (sciatic), small intestine: ileum with Peyer's patches, spinal cord, stomach, thyroid gland, trachea, urinary bladder.	✓
9. Place testes in modified Davidson's fixative for 48 hrs according to SOP LAB-038..	✓
10. Dissect out the eyes and place in Davidson's fixative according to SOP LAB-038..	✓
11. Place all tissue/or organs showing any abnormalities in 10% formalin for possible histopathology.	✓
12. Place all carcasses in 10% buffered formalin.	✓ [initials] 8/2/12
13. Observe each animal in Groups 4 & 5 for signs of toxicity according to SOP LAB-028 and record observations on form LAB-028/f2.	✓
Study Director	[initials] 8/2/12

[initials] 9/2/12

Day		Date	9/2/12 +	Site	Beaconsfield

Procedure: Transfer blood samples to IDEXX laboratories (Test Site 1)	Operator
1. Complete the chain of custody form ADM-010/f1.	✓
2. Pack all blood samples according to SOP ADM-010.	✓
3. Transfer the blood samples according to SOP ADM-013 to Test Site 1, for haematology and biochemistry analysis and complete form ADM-013/f1.	✓ [illegible] 9/2/12
**Study Director**	[illegible] 9/2/12

DAY		DATE	10/2/12 – 21/2/12	SITE	Beaconsfield

Procedure: Trimming tissues, histological processing, embedding, slide preparation Groups 1 and 3	OPERATOR
1. When the tissues are fixed (at least 48 hours), trim tissues (in the table below) and place in cassettes for processing according to SOP LAB-065.	✓
2. Place cassettes in the tissue processing machine according to SOP LAB -156.	✓ [illegible] 12/2/12
3. Embed tissues, prepare slides according to SOP LAB -157 and SOP LAB-147.	✓ [illegible]
**Study Director** [illegible] 13/2/12 [illegible] 14/2/12 [illegible] 15/2/12 [illegible] 16/2/12 21/2/12	[illegible] 10/2/12

Group	Tissues for histopathology
Groups 1 and 3	Gross lesions,<br>adrenal glands<br>bone marrow,<br>brain (cerebrum, cerebellum, brain stem),<br>epididymis,<br>heart,<br>kidney,<br>large intestine: colon,<br>liver,<br>lung,<br>lymph nodes,<br>prostate,<br>seminal vesicles with coagulating glands,<br>small intestine: ileum (including Peyer's patches),<br>spinal cord,<br>spleen,<br>stomach,<br>thymus,<br>thyroid gland,<br>trachea,<br>testes,<br>urinary bladder.

Date			15/2/12 – 22/2/12	Site	Beaconsfield
**Procedure:**<br>**Stain tissue sections**					**Operator**
1. Stain the tissue sections with Haematoxylin and Eosin according to SOP LAB - 158.					[illegible] 20/2/12
**Study Director**					[illegible] 22/2/12

Day	30	Date	9/2/12	Site	Beaconsfield
**Procedure:**<br>**Observation (recovery groups)**					**Operator**
1. Observe each animal carefully for signs of toxicity according to SOP LAB-028 and record observations on form LAB-028/f2.<br>2. Weigh any feed added and record on form LAB-024/f2.					✓<br>✓<br>[illegible] 9/2/12
**Study Director**					[illegible] 9/2/12

Day	31	Date	10/2/12	Site	Beaconsfield
**Procedure:**<br>**Observation (recovery groups) and transfer of organs to 10% formalin**					**Operator**
1. Observe each animal carefully for signs of toxicity according to SOP LAB-028 and record observations on form LAB-028/f2.<br>2. Transfer eyes and testes to 10% formalin.<br>3. Weigh any feed added and record on form LAB-024/f2.					✓<br>✓<br>✓ [illegible] 10/2/12
**Study Director**					[illegible] 10/2/12

Day	32-35	Date	11/2/12 – 14/2/12	Site	Beaconsfield
**Procedure:**<br>**Observation (recovery groups)**					**Operator**
1. Observe each animal carefully for signs of toxicity according to SOP LAB-028 and record observations on form LAB-028/f2.<br>2. Weigh any feed added and record on form LAB-024/f2.					✓<br>✓ [illegible] 11/2/12
**Study Director**					[illegible] 11/2/12

[illegible] 12/2/12 [illegible] 13/2/12 [illegible] 14/2/12

Day	36	Date	15/2/12	Site	Beaconsfield
**Procedure:** Bodyweights, observation, feed intake (recovery groups)					**Operator**
1. Weigh all animals according to SOP LAB-027 and record on form LAB-027/f2.					✓
2. Weigh remaining feed in hoppers and record on form LAB-024/f2. Place a fresh known amount of feed in the hoppers of each cage and record on form LAB-024/f2.					✓
3. Observe each animal carefully for signs of toxicity according to SOP LAB-028 and record observations on form LAB-028/f2.					✓ 15/2/12
**Study Director**					15/2/12

Day	37-41	Date	16/2/12 – 20/2/12	Site	Beaconsfield
**Procedure:** Observation (recovery groups)					**Operator**
1. Observe each animal carefully for signs of toxicity according to SOP LAB-028 and record observations on form LAB-028/f2.					✓
2. Weigh any feed added and record on form LAB-024/f2.					✓ 16/2/12
**Study Director**					16/2/12

17/2/12 18/2/12 19/2/12 20/2/12

Day	42	Date	21/2/12	Site	Beaconsfield
**Procedure:** Bodyweights, observations, feed removal (recovery groups)					**Operator**
1. Observe each animal carefully for signs of toxicity according to SOP LAB-028 and record observations on form LAB-028/f2.					✓
2. Weigh all remaining animals according to SOP LAB-27 and record on form LAB-027/f2.					✓
3. Weigh remaining feed in hoppers and record on form LAB-024/f2					✓ 21/2/12
4. Discard feed (around 4-5 pm).					
**Study Director**					21/2/12

Day	43	Date	22/2/12	Site	Beaconsfield

**Procedure:** **Sacrifice recovery groups (Groups 4 & 5)**	**Operator**
1. Collect urine from each animal in Groups 1, 2 and 3 and conduct urinalysis according to SOP LAB-090 and record results on form LAB-090/f1.	✓
2. Weigh all animals in Groups 1, 2 and 3 according to SOP LAB-027 and record on form LAB-027/f2.	✓
3. Anaesthetise animals in Groups 1, 2 and 3 with Lethabarb by intraperitoneal (ip) injection according to SOP LAB-037.	✓ [illegible]
4. Withdraw approximately 4 mL of blood by cardiac puncture according to SOP LAB-035.	✓ [illegible]
5. Place blood into EDTA and lithium heparin tubes and mix gently.	
6. Examine macroscopically the external surfaces, cranial, abdominal and thoracic cavities and their contents according to SOP LAB-038 and record observations on form LAB-038/f1.	✓
7. Dissect out and weigh the liver, kidneys, adrenals, testes, spleen, thymus, heart, thymus, epididymis, prostate + seminal vesicles with coagulating glands as whole and brain and record the wet weight on form LAB-038/f1. Place organs in 10% formalin.	✓
8. Dissect out and place in formalin all gross lesions, bone marrow, eye, large intestine: colon, lung, lymph nodes, peripheral nerve (sciatic), small intestine: ileum with Peyer's patches, spinal cord, stomach, thyroid gland, trachea, urinary bladder.	✓
9. Place testes in modified Davidson's fixative for 48 hrs according to SOP LAB-038..	✓
10. Dissect out the eyes and place in Davidson's fixative for 48 hrs according to SOP LAB-038..	✓
11. Place all tissue/or organs showing any abnormalities in 10% formalin for possible histopathology.	✓ [illegible] 22/2/12
12. Place all carcasses in 10% buffered formalin.	✓
**Study Director**	[illegible] 22/2/12

Day		Date	22/2/12	Site	Beaconsfield

**Procedure:** **Transfer blood samples to IDEXX laboratories (Test Site 1)**	**Operator**
1. Complete the chain of custody form ADM-010/f1.	✓
2. Pack all blood samples according to SOP ADM-010.	✓
**3.** Transfer the blood samples according to SOP ADM-013 to Test Site 1, for haematology and biochemistry analysis and complete form ADM-013/f1.	✓ [illegible] 22/2/12
**Study Director**	[illegible] 22/2/12

Day		Date		Site	Beaconsfield
**Procedure:** **Trimming tissues, histological processing, embedding, slide preparation and staining Groups 4 and 5 IF APPLICABLE**					**Operator**
If microscopic toxicity is found in groups 1 and 3 further processing may be required.<br>1. Trim and place in cassettes for processing according to SOP LAB-065.<br>2. Place cassettes in the tissue processing machine SOP LAB -157.<br>3. Embed tissues, prepare slides, and stain with H&E SOP LAB 147, 157 and 158.<br>4. Stain the tissue sections with Haematoxylin and Eosin according to SOP LAB - 158.					
**Study Director** Not Required					12/03/12

Date			22/2/12.	Site	
**Procedure:** **Transfer slides to histopathologist (Test Site 2)**					**Operator**
1. Transfer slides to histopathologist (Test Site 2) according to SOP ADM-010, with a copy of the study plan and forms ADM-010/f1 and ADM-010/f4 and chain of custody.					[illegible]
**Study Director**					22/2/12.

Date			29/2/12 – 6/3/12.	Site	Beaconsfield
**Procedure:** **Evaluation of slides (Test Site 2)**					**Operator**
1. Receive slides from ICP Firefly (Test Facility).<br>2. Evaluation of slides by histopathologist and record on the computer.<br>3. Report results and send to ICP Firefly (Test Facility).					See Appendix E.
**Study Director**					12/3/12.

Date			12/03/12.	Site	
**Procedure:** **Receipt of slides and report from histopathologist (Test Site 2)**					**Operator**
1. Receive slides and report from histopathologist.					[illegible]
**Study Director**					12/03/12.

Day		Date	6/1/12 – 9/3/12.	Site	Beaconsfield
**Procedure:** **Statistical analysis**					**Operator**
1. Enter raw data into spreadsheets.<br>2. Analyse data.					✓<br>✓
**Study Director**					12/3/12

Day		Date	14/03/12	Site	Beaconsfield
**Procedure:**<br>**Final report writing**					**Operator**
1. Write report.<br>2. Send report to Sponsor					✓ d 14/03/12
**Study Director**					d 14/03/12

7.0. RECORDS

The following records and histopathology slides will be retained in ICP Firefly archives for a period of 6 years:

- Study plan
- Raw data
- Copy of Final report
- Internal audit reports
- Records and reports of maintenance and calibration of equipment

Day		Date	14/03/12	Site	Beaconsfield
**Procedure:**<br>**Final report writing**					**Operator**
1. Write report.<br>2. Send report to Sponsor					[illegible] 14/03/12<br>[illegible] 14/03/12
**Study Director**					[illegible] 14/03/12

7.0. RECORDS

The following records and histopathology slides will be retained in ICP Firefly archives for a period of 6 years:

- Study plan
- Raw data
- Copy of Final report
- Internal audit reports
- Records and reports of maintenance and calibration of equipment

The nominated Study Director has fully read the study plan and by their signature subscribed hereto, accepts the terms thereof.

ICP Firefly Pty Ltd
Test facility

Signature

NAME: Fiona Brook

POSITION: Study Director

DATE: 29 APRIL 2011

The nominated signatory of the Sponsor and Contractor have fully read and agree to the procedures specified in the study plan, and by their signature subscribed hereto, accept the terms thereof.

St George Clinical School
(the Sponsor)

Signature

NAME: **Prof. Paul De Souza**

POSITION: MEDICAL ONCOLOGIST

DATE: 30 APRIL 2011

ICP Firefly Pty Ltd
(the Contractor)

Signature

NAME: Dr I. Meyer-Carrive

POSITION: Managing Director

DATE: 29 April 2011

ICP Firefly Pty Ltd
(the contractor)

Signature

NAME: Edmund Ho

POSITION: QA Manager

DATE: 29 APRIL 2011

The nominated Study Director has fully read the study plan and by their signature subscribed hereto, accepts the terms thereof.

ICP Firefly Pty Ltd
Test facility

Signature

NAME: Fiona Brook

POSITION: Study Director

DATE: 29 APRIL 2011

The nominated signatory of the Sponsor and Contractor have fully read and agree to the procedures specified in the study plan, and by their signature subscribed hereto, accept the terms thereof.

St George Clinical School
(the Sponsor)

Signature

NAME: **Prof. Paul De Souza**

POSITION:

DATE:

ICP Firefly Pty Ltd
(the Contractor)

Signature

NAME: Dr I. Meyer-Carrive

POSITION: Managing Director

DATE: 29 April 2011

ICP Firefly Pty Ltd
(the contractor)

Signature

NAME: Edmund Ho

POSITION: QA Manager

DATE: 29 APRIL 2011

Kevin Isaacs
Consultant in Toxicological Pathology

22 December 2011

Fiona Brook
Senior Research Officer
ICP Firefly Pty Ltd
PO Box 6198
Alexandria
NSW 2015
Australia

Dear Fiona

Please find one signed copy of the PI agreement for Study No: ICPQN1035.B.

If you have any queries, please do not hesitate to contact us.

Best Regards

Yours sincerely

[signature]

Kevin Isaacs MA, VetMB, MRCVS, FRCPath
Consultant in Toxicological Pathology

14 ROSSETT PARK ROAD • HARROGATE • NORTH YORKSHIRE • HG2 9NP
Telephone: 01423-870045 • Fax: 01423-870045 • Email: KICITP@aol.com

Partners: K.R. & H.M. Isaacs • VAT No.: 613 4226 75

The bright light of

certainty

I C P Firefly Pty Ltd

ACN 071 626 358

PO Box 6198, Alexandria NSW 2015 Australia
TEL: 61 2 9310 3899 FAX: 61 2 9310 4889 EMAIL: info@icpfirefly.com.au WEBSITE: www.icpfirefly.com.au

PRINCIPAL INVESTIGATOR AGREEMENT

Test Facility Study Number: ICPQN1035.B

Test Site Study Number: K12/002

1. Principal Investigator

Dr Kevin Isaacs

14 Rossett Park Road

Harrogate

North Yorkshire HG2 9NP

UNITED KINGDOM

Phone/Fax: 01423 870045

Email: kicitp@aol.com

2. Delegated Phase of the Study

Histopathology slide examination, interpretation and reporting.

3. Responsibilities

The Principal Investigator shall

- Ensure the study is performed according to the approved Study Plan and any amendments in accordance with current GLP legislation of the country in which the phase of the study will be performed and the OECD Principles of GLP as most recently amended.
- Document and report any deviations from the Study Plan or SOPs

Test Site Management shall

- Ensure the Principal Investigator has adequate qualifications, training and experience to perform the delegated phase.

Test Site Quality Assurance shall

- Inspect the delegated phase of the study for compliance with GLP and the Study Plan.
- Inspect the report for compliance with GLP.

4. APPROVAL

Test Facility Management has nominated the Principal Investigator named in Clause 1 of this Agreement to perform the Delegated Phase of the Study described in Clause 2.

ICP Firefly Pty Ltd
Test Facility

Signature

NAME: Dr I. Meyer-Carrive

POSITION: Managing Director

DATE: 21 December 2011

The nominated Principal Investigator and Test Site Management have read the Agreement and by their signature subscribed hereto, accept the terms thereof.

Kevin Isaacs
Consultant in Toxicological Pathology
Test site / Principal Investigator

Signature

NAME: Kevin Isaacs

MA, VetMB, MRCVS, FRCPath

POSITION: Principal Investigator

DATE: 21.12. 11

Kevin Isaacs
Consultant in Toxicological Pathology
Test site / Management

Signature

NAME: Kevin Isaacs

MA, VetMB, MRCVS, FRCPath

POSITION: MANAGER

DATE: 21.12.11
